# Supplementary material for: Emergency Medicine Obstetrics and Gynecology: A Case-Based Curriculum for Residents
Source: MedEdPORTAL. 2023 Aug 11;19:11330. doi: 10.15766/mep_2374-8265.11330 (PMC10415535; doi:10.15766/mep_2374-8265.11330)
Supplement: Supplementary file 1 — Ectopic Pregnancy and Emergencies in the First 20 Weeks.pptxPregnancy Emergencies After 20 Weeks.pptxDelivery Emergencies.pptxPelvic Pain in the Nonpregnant Patient.pptxVaginitis, Cervicitis, and PID.pptxAbnormal Uterine Bleeding.pptxLabor and Perimortem C-Section.pptxSession Review Questions.docxPrecurriculum Survey.docxPostcurriculum Survey.docx [file mep_2374-8265.11330-s001.zip › H. Session Review Questions.docx]

**Ectopic Pregnancy and Emergencies in the first 20 weeks**

1. When should you first expect to see cardiac activity on transvaginal ultrasound?
   1. 4 weeks
   2. 5 weeks
   3. **6 weeks**
   4. 7 weeks
2. Which antibiotic is the first-line treatment for UTI in a patient who is 8 weeks pregnant?
   1. Nitrofurantoin
   2. Trimethoprim/sulfamethoxazole
   3. Clindamycin
   4. **Cephalexin**
3. What dose of Rhogam will you give to your Rh- patient who is 6 weeks pregnant with an ectopic pregnancy.
   1. **50**
   2. 150
   3. 200
   4. 350
4. Which of the following is an ABSOLUTE contraindication to methotrexate for treatment of an ectopic pregnancy?
   1. Beta HCG > 5,000 mIU/mL
   2. Fetal cardiac activity
   3. Good follow up plan
   4. **Platelet count of 10,000**

**Pregnancy Emergencies after 20 weeks Gestation**

1. Which best correlates with the severity of bleeding in a placental abruption?
   1. **Fibrinogen**
   2. Hemoglobin level
   3. Visualized bleeding
   4. Kelihauer-Betke result
2. What is the systolic blood pressure that defines severe pre-eclampsia?
   1. 140
   2. **160mmHg**
   3. 150 mmHg
   4. 180 mmHg
3. Cut-off gestational age for empiric steroids in premature labor?
   1. 30 weeks
   2. 32 weeks
   3. **34 weeks**
   4. 36 weeks
4. What should you avoid using in the treatment of chronic hypertension in pregnancy?
   1. Labetalol
   2. Hydralazine
   3. Amlodipine
   4. Lisinopril (correct)
5. What is the initial dose of magnesium in eclampsia?
   1. 2mg over 3 minutes
   2. **4 mg over 20 minutes**
   3. 4 mg over 60 minutes
   4. 8 mg over 60 minutes

**Delivery Emergencies**

1. What is an important side effect to consider when giving an oxytocin bolus for postpartum hemorrhage?
   1. Hallucinations
   2. Uterine tachy-systole
   3. Hypernatremia
   4. **Hypotension**
2. What is the ideal patient positioning when there is cord prolapse?
   1. Supine with knees to chest in trendelenburg
   2. **Prone with hips in the air in trendelenburg**
   3. Extreme lithotomy
   4. Prone with hips in the air in reverse trendelenburg
3. What is the most common cause of postpartum hemorrhage?
   1. Retained tissue
   2. Trauma
   3. **Uterine atony**
   4. Uterine inversion
4. Patient just delivered and you notice a fleshy mass in the vagina concerning for uterine inversion. What should you do?
   1. Administer oxytocin
   2. Deliver the placenta if it’s still adhered to the uterus
   3. **Cup the fundus of the uterus and push in an anterior and superiorly direction**
   4. Cup the fundus and push in a posterior and superior direction.
5. A patient is delivering their baby and you notice the head retracts when she is not pushing. What should you try next?
   1. **Perform suprapubic pressure and place the patient in extreme lithotomy position.**
   2. Prone with hips in the air in Trendelenburg
   3. Tell patient to stop pushing
   4. Apply more downward traction to baby’s head

**Labor/Delivery and Perimortem C-Section**

1. What is the minimum gestational age for which a perimortem C-section would be recommended?
   1. 18 weeks
   2. **20 weeks**
   3. 28 weeks
   4. 32 weeks
2. Ideal time for perimortem c-section to occur:
   1. Within 1 hour of cardiac arrest
   2. Within 15 minutes of cardiac arrest
   3. Within 30 minutes of cardiac arrest
   4. **Within 5 minutes of cardiac arrest**
3. Incisional approach to perimortem c-section
   1. **Vertical abdominal incision, vertical uterine incision**
   2. Vertical abdominal incision, horizontal uterine incision
   3. Horizontal abdominal incision, vertical uterine incision
   4. Horizontal abdominal incision, horizontal uterine incision
4. What are the first four cardinal movements of labor in correct order?
   1. Engagement, Descent, Extension, Internal Rotation
   2. **Engagement, Descent, Flexion, Internal Rotation**
   3. Descent, Engagement, Flexion, Internal Rotation
   4. Flexion, Internal Rotation, Descent, Engagement

**Abdominal pain in a non-pregnant female**

1. Which of the following provides a definitive diagnosis of ovarian torsion?
   1. Transvaginal ultrasound
   2. CT abdomen/pelvis
   3. MR pelvis
   4. **Laparoscopy**
2. A 31-year-old female presents with cyclic pelvic pain, worsening over the last few months. Associated with dysmenorrhea, dyspareunia. Pelvic exam notable for palpable adnexal mass on left side. Negative urine pregnancy test. What is the most likely diagnosis?
   1. Ruptured ovarian cyst
   2. Ovarian torsion
   3. Ovarian hyperstimulation syndrome
   4. **Endometriosis**
3. What is the most common location for ectopic endometrial tissue in endometriosis?
   1. Pleural cavity
   2. **Ovary**
   3. Ureter
   4. Bladder

**Vaginitis, Cervicitis, and PID**

1. Which of the following would be appropriate for a patient with vaginal discharge, fever, and lower abdominal pain?
   1. **Ceftriaxone, doxycycline and metronidazole**
   2. Ceftriaxone and azithromycin
   3. Metronidazole
   4. Vancomycin and Zosyn
2. Which of the following is true regarding BV?
   1. It is a sexually transmitted infection
   2. Partners with penises should be treated
   3. Vaginal pH will be acidic
   4. **It is due to an overgrowth of gardnerella**
3. Which of the following does not require partner treatment?
   1. Trichomonas
   2. Chlamydia
   3. **Yeast infection**
   4. Gonorrhea
4. Which of the following is an indication for hospitalization for PID?
   1. Adnexal tenderness
   2. Post-coital bleeding
   3. **Pregnancy**
   4. Previous PID
5. What is an appropriate length of time to leave a Word Catheter in a bartholin abscess I&D?
   1. 7 days
   2. 10 days
   3. 2 weeks
   4. **4 weeks**

**Abnormal Uterine Bleeding**

1. What is the correct dose of TXA for AUB?
   1. **10mg/kg**
   2. 0.01mg/kg
   3. 0.1mg/kg
   4. 1mg/kg
2. What is the normal thickness of the endometrium in post-menopausal females?
   1. <20mm
   2. **<5mm**
   3. <15mm
   4. <10mm
3. What is the correct dose of IV conjugated estrogen?
   1. 25g
   2. 2.5mg
   3. **25mg**
   4. 250mg
4. Which of these is a contraindication to estrogen use in females?
   1. Sexual activity
   2. NSAID use
   3. **Hypertension**
   4. Thyroid disease
