# Supplementary material for: Emergency Medicine Obstetrics and Gynecology: A Case-Based Curriculum for Residents
Source: MedEdPORTAL. 2023 Aug 11;19:11330. doi: 10.15766/mep_2374-8265.11330 (PMC10415535; doi:10.15766/mep_2374-8265.11330)
Supplement: Supplementary file 1 — Ectopic Pregnancy and Emergencies in the First 20 Weeks.pptxPregnancy Emergencies After 20 Weeks.pptxDelivery Emergencies.pptxPelvic Pain in the Nonpregnant Patient.pptxVaginitis, Cervicitis, and PID.pptxAbnormal Uterine Bleeding.pptxLabor and Perimortem C-Section.pptxSession Review Questions.docxPrecurriculum Survey.docxPostcurriculum Survey.docx [file mep_2374-8265.11330-s001.zip › A. Ectopic Pregnancy and Emergencies in the First 20 Weeks.pptx]

## Slide 1
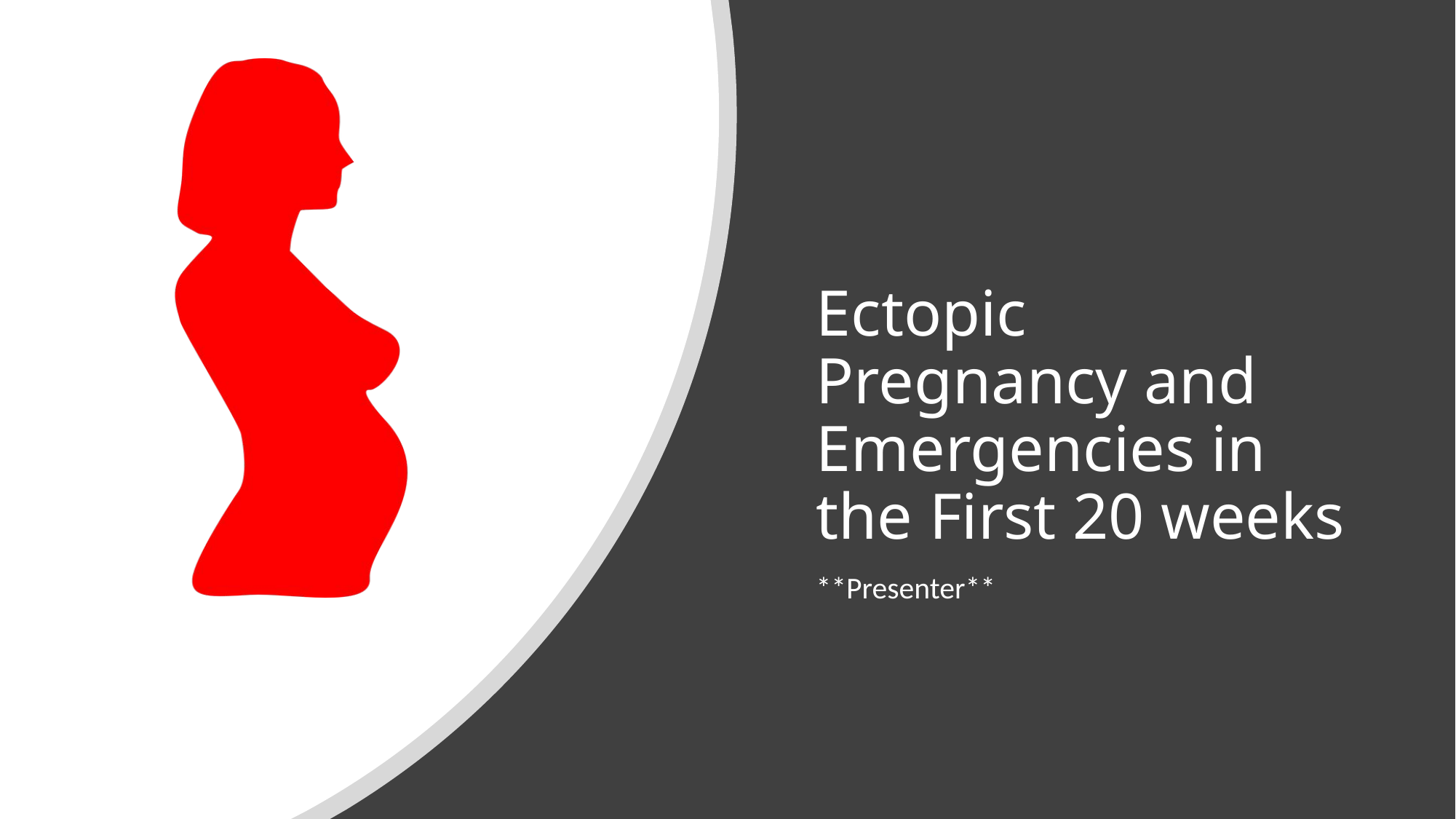

# Ectopic Pregnancy and Emergencies in the First 20 weeks
**Presenter**

## Slide 2
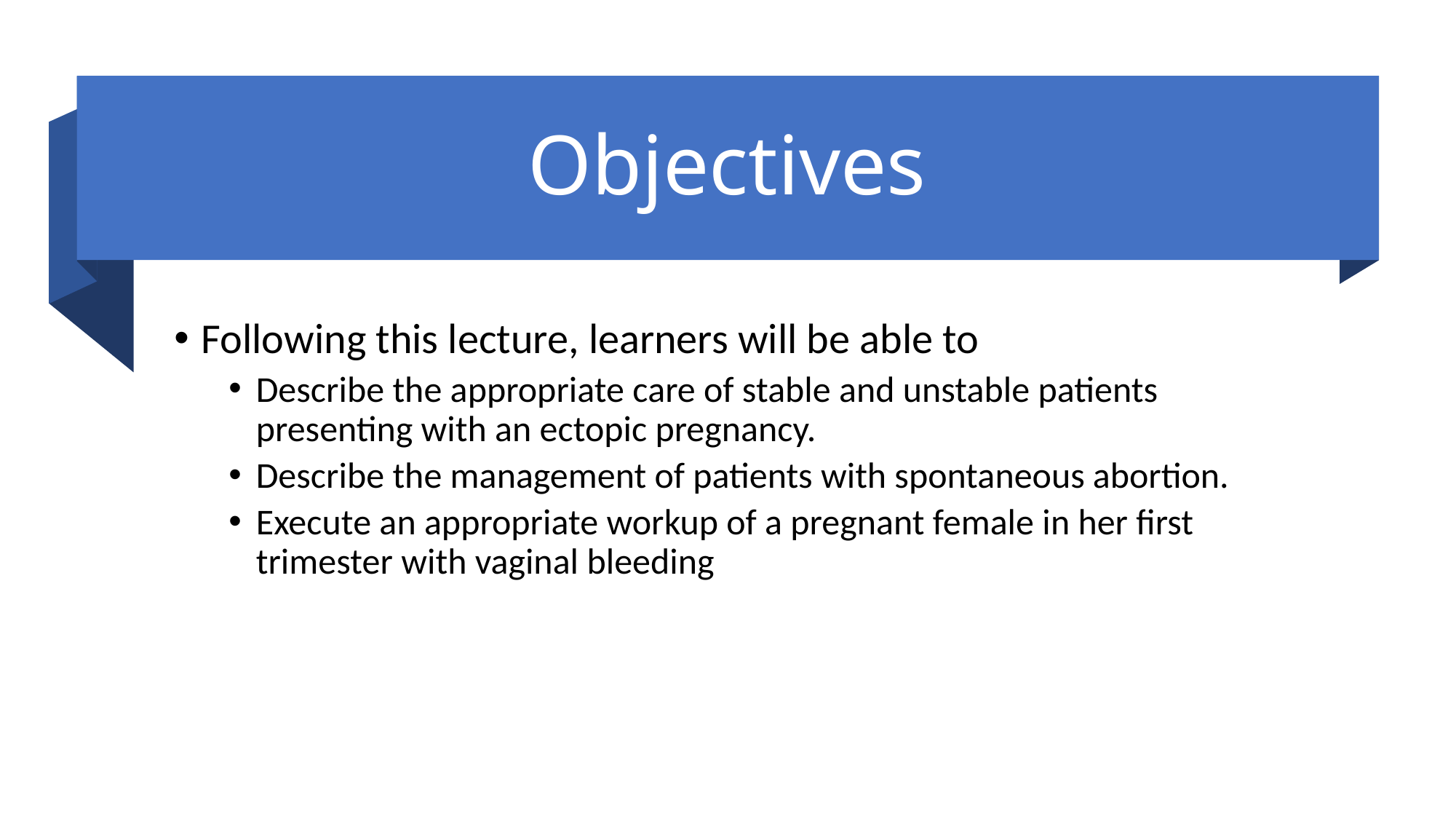

# Objectives
Following this lecture, learners will be able to
Describe the appropriate care of stable and unstable patients presenting with an ectopic pregnancy.
Describe the management of patients with spontaneous abortion.
Execute an appropriate workup of a pregnant female in her first trimester with vaginal bleeding

## Slide 3
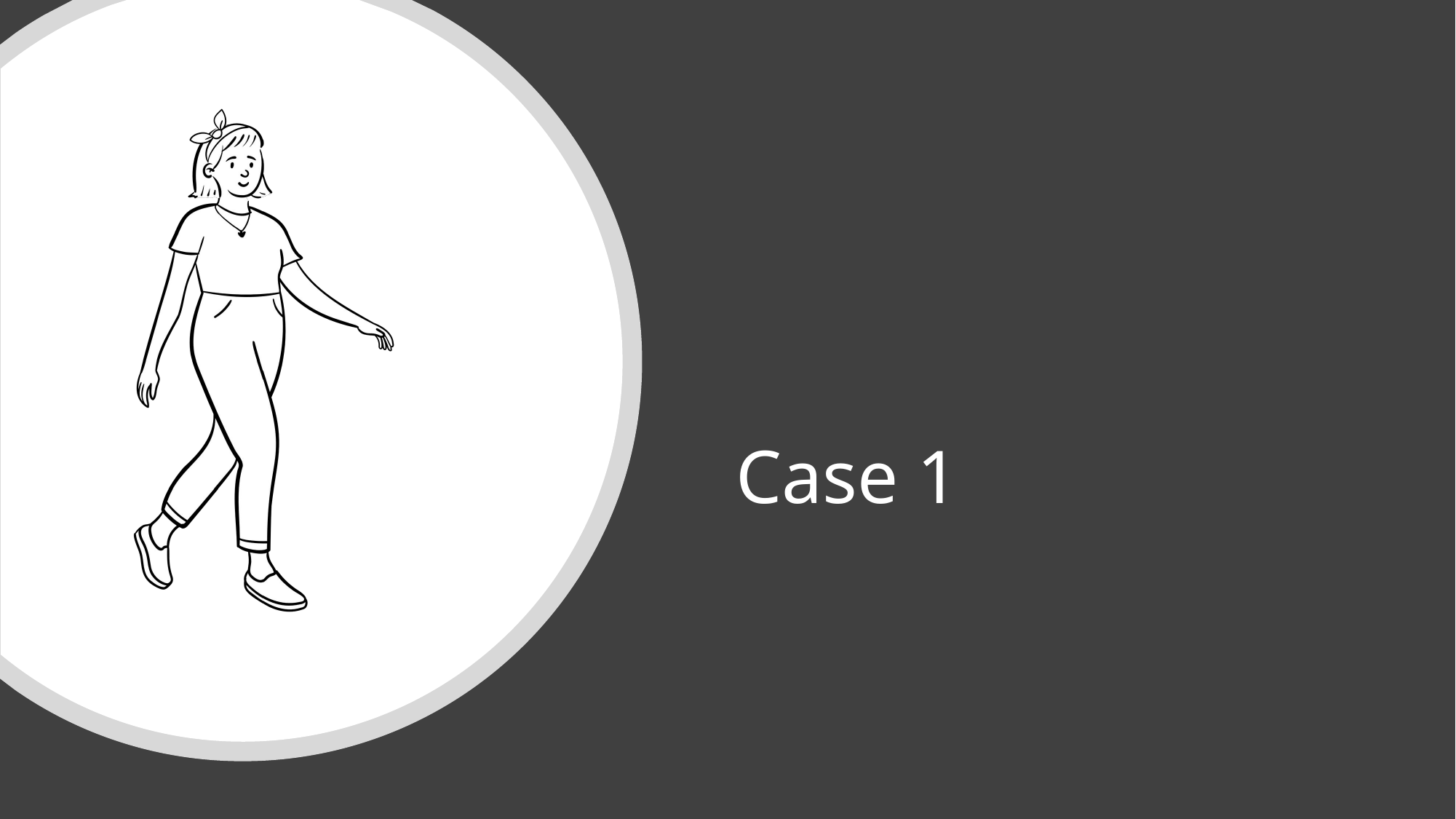

# Case 1

## Slide 4
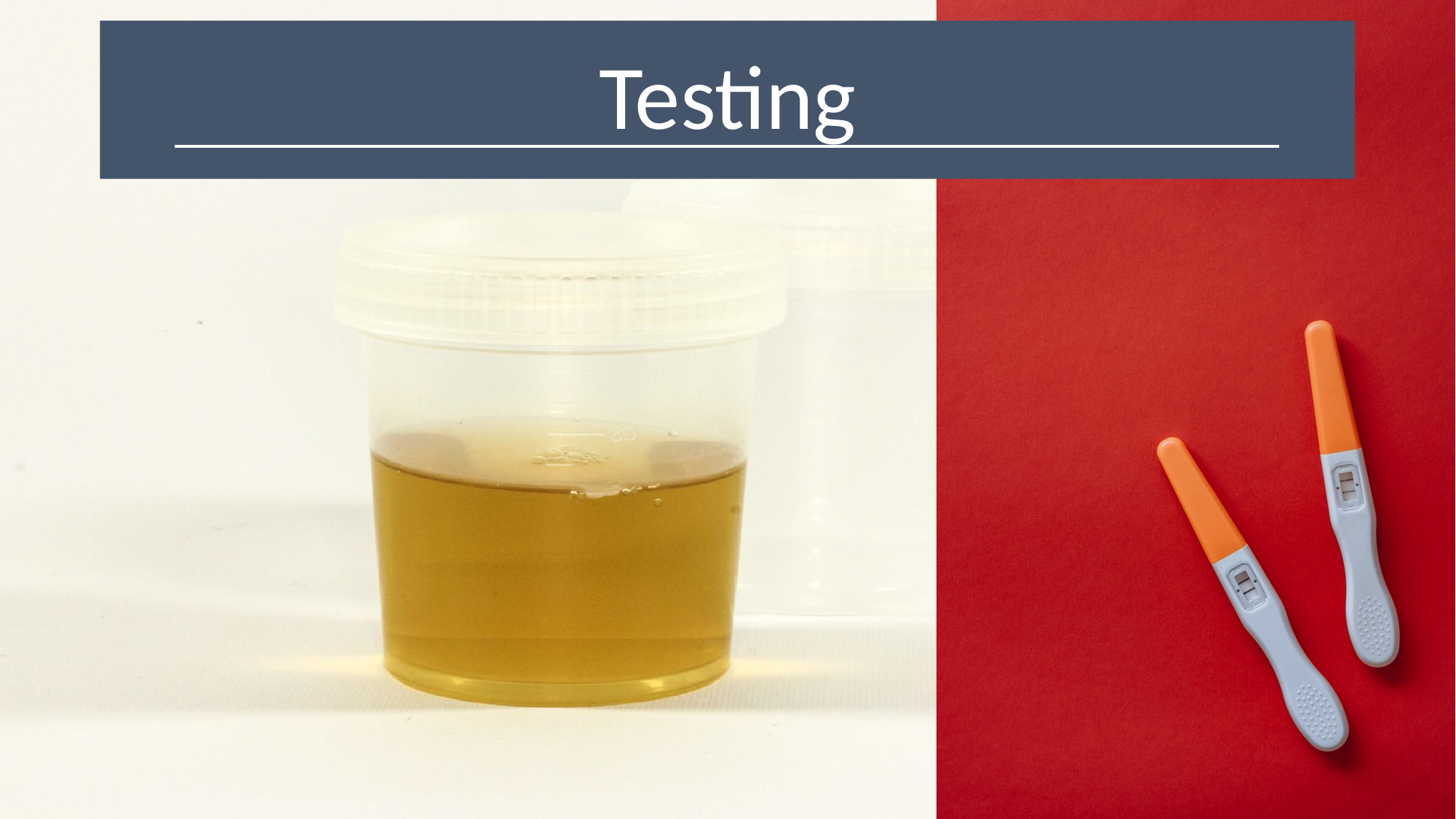

Testing

## Slide 5
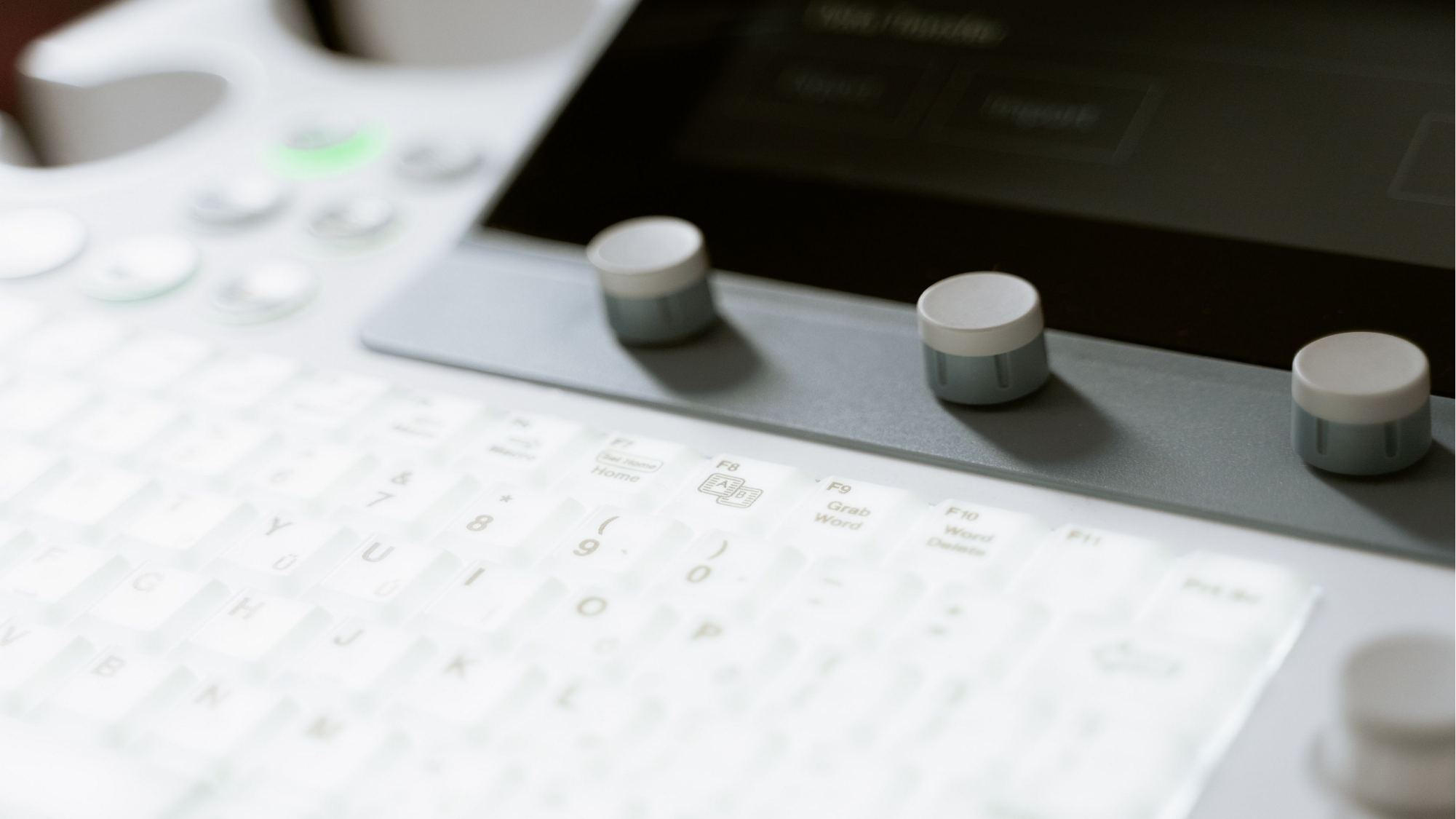

## Slide 6
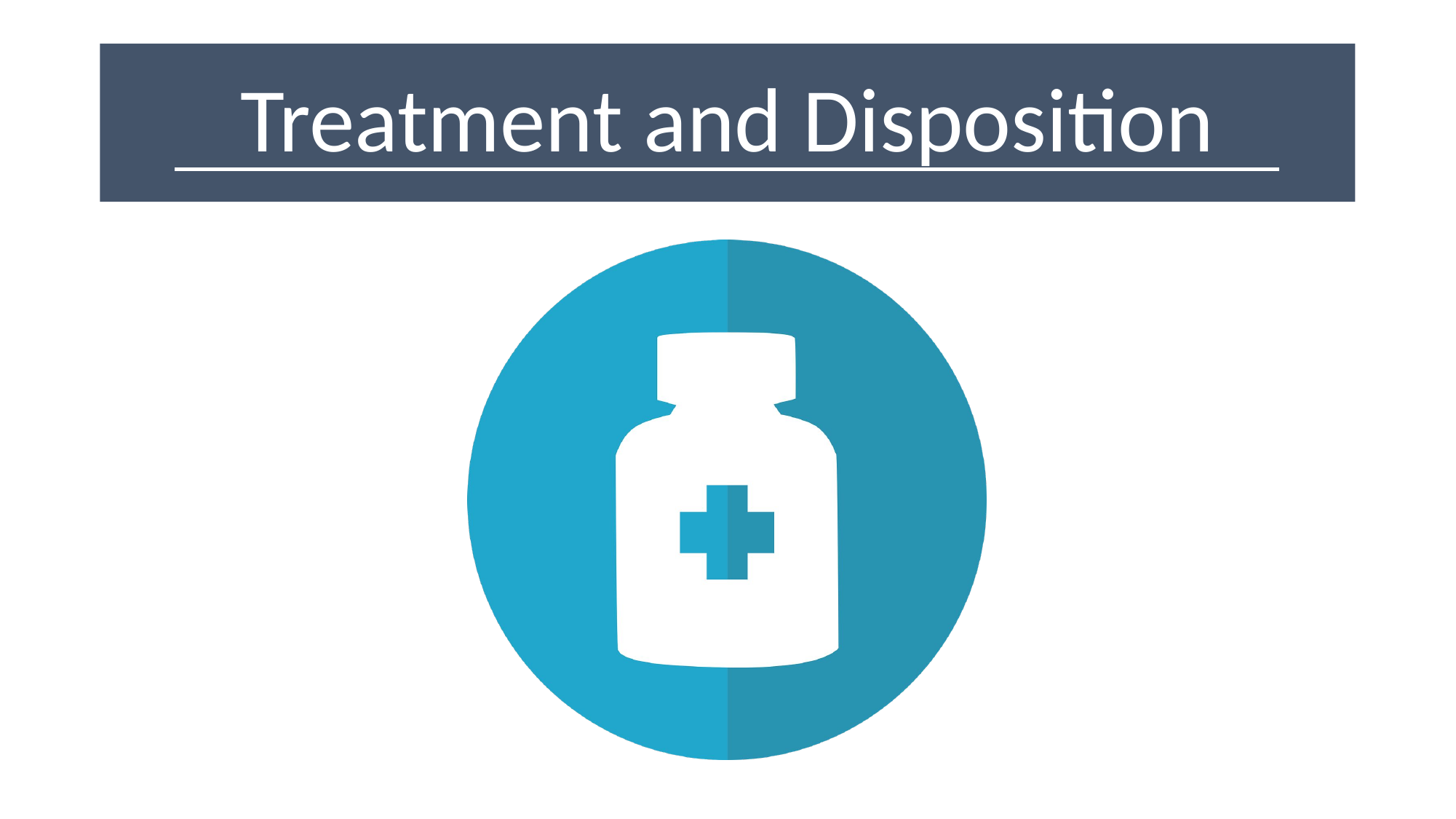

Treatment and Disposition

## Slide 7
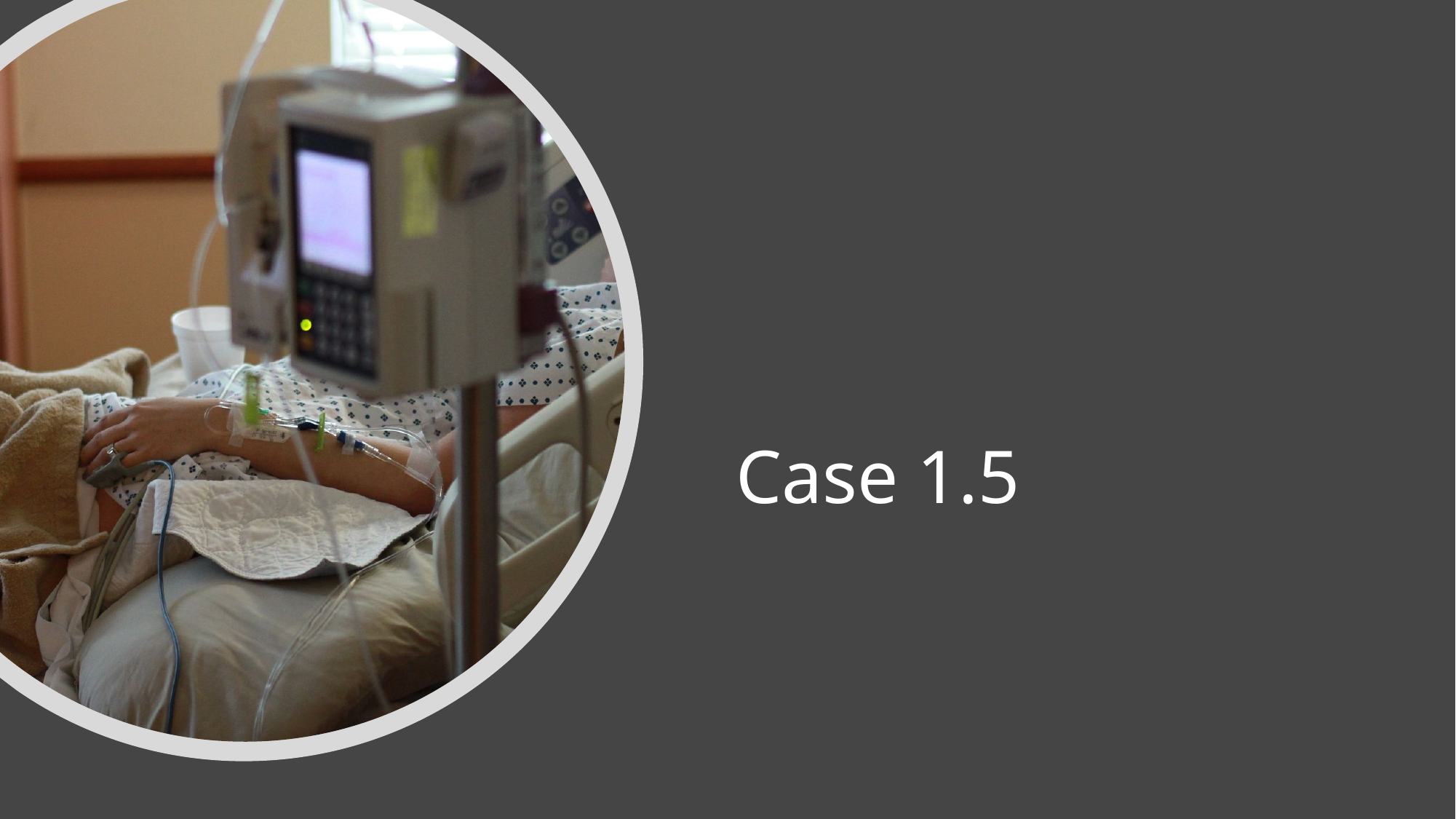

# Case 1.5

## Slide 8
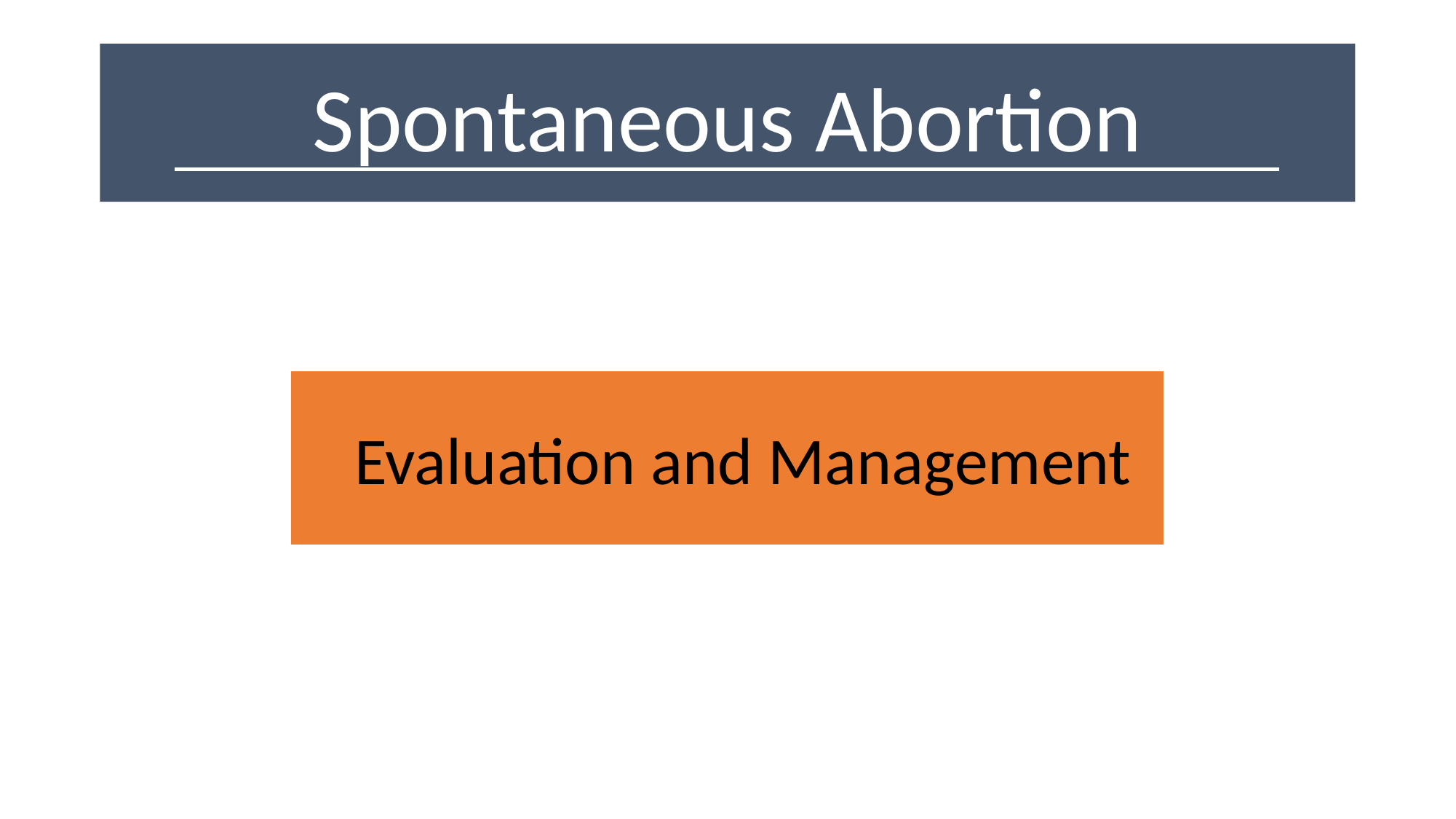

Spontaneous Abortion
Evaluation and Management

## Slide 9
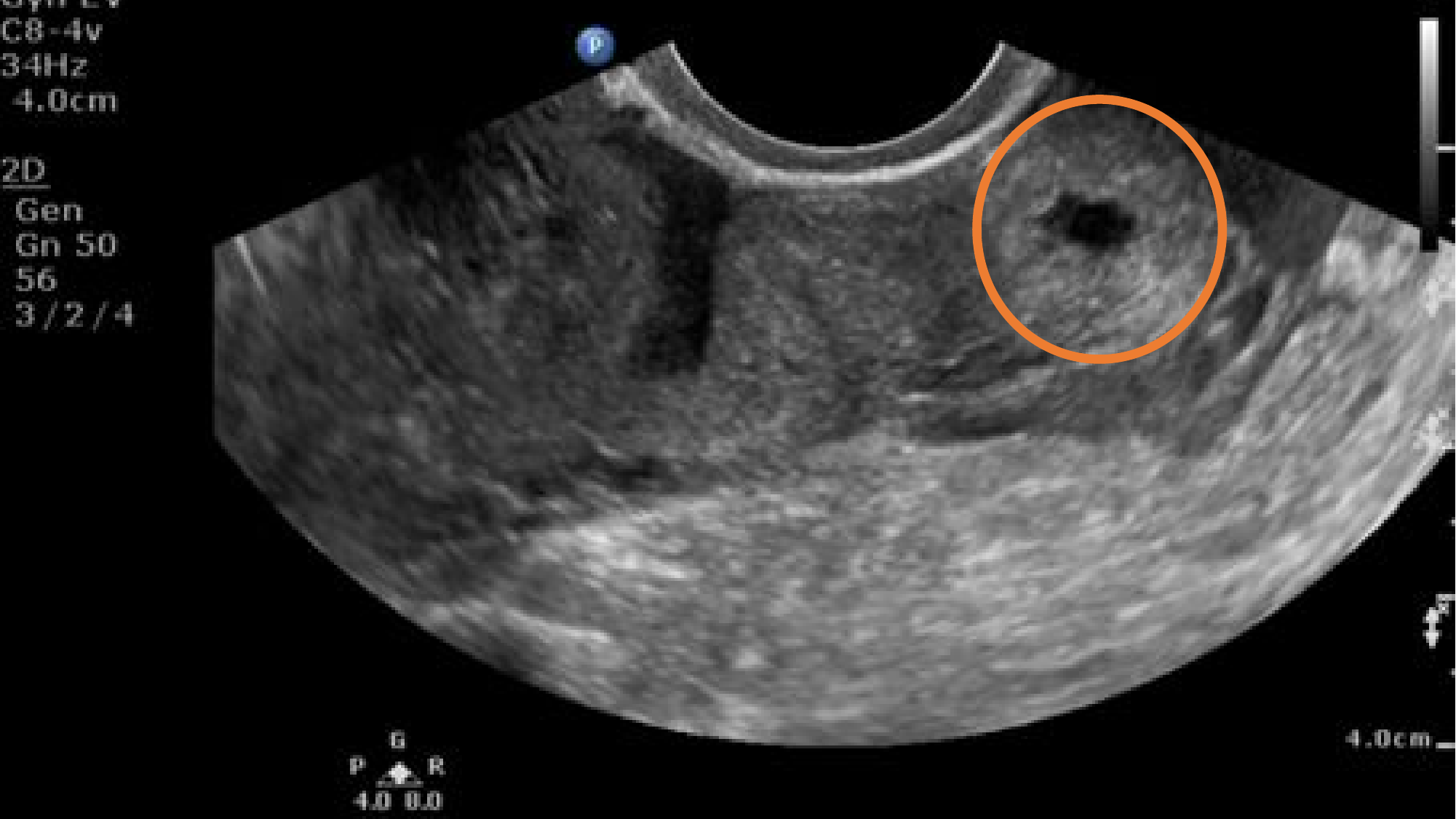

## Slide 10
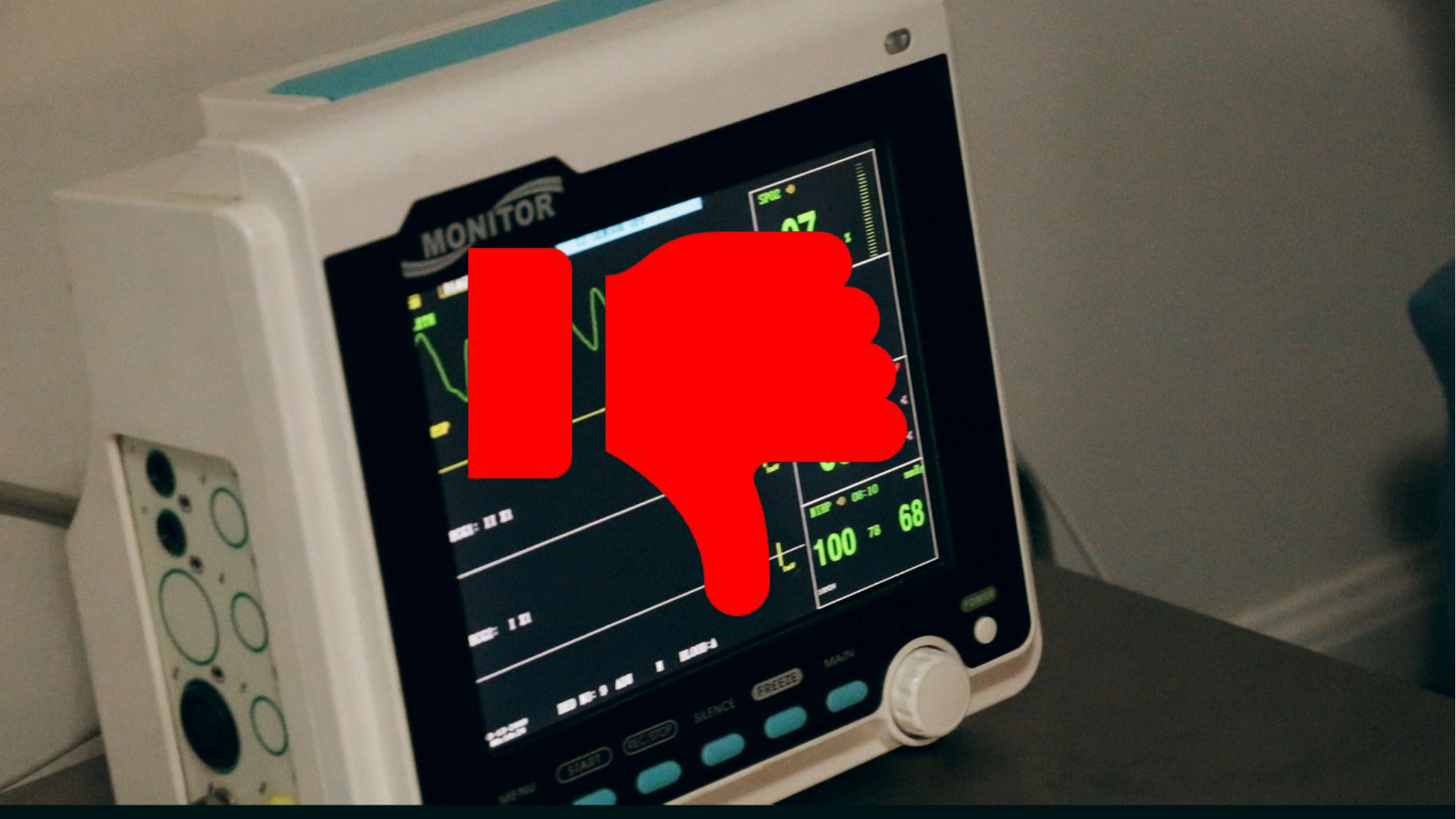

## Slide 11
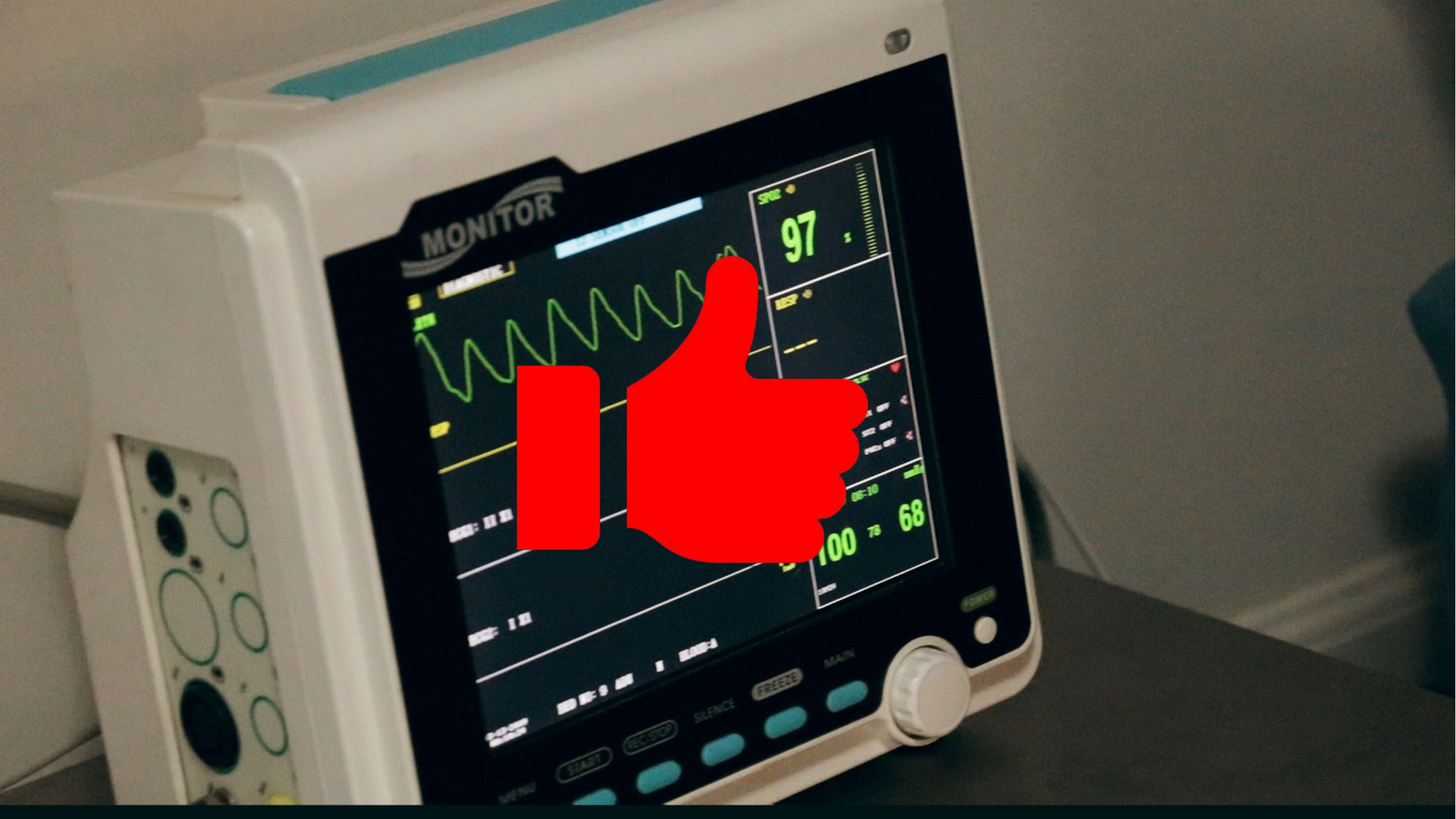

## Slide 12
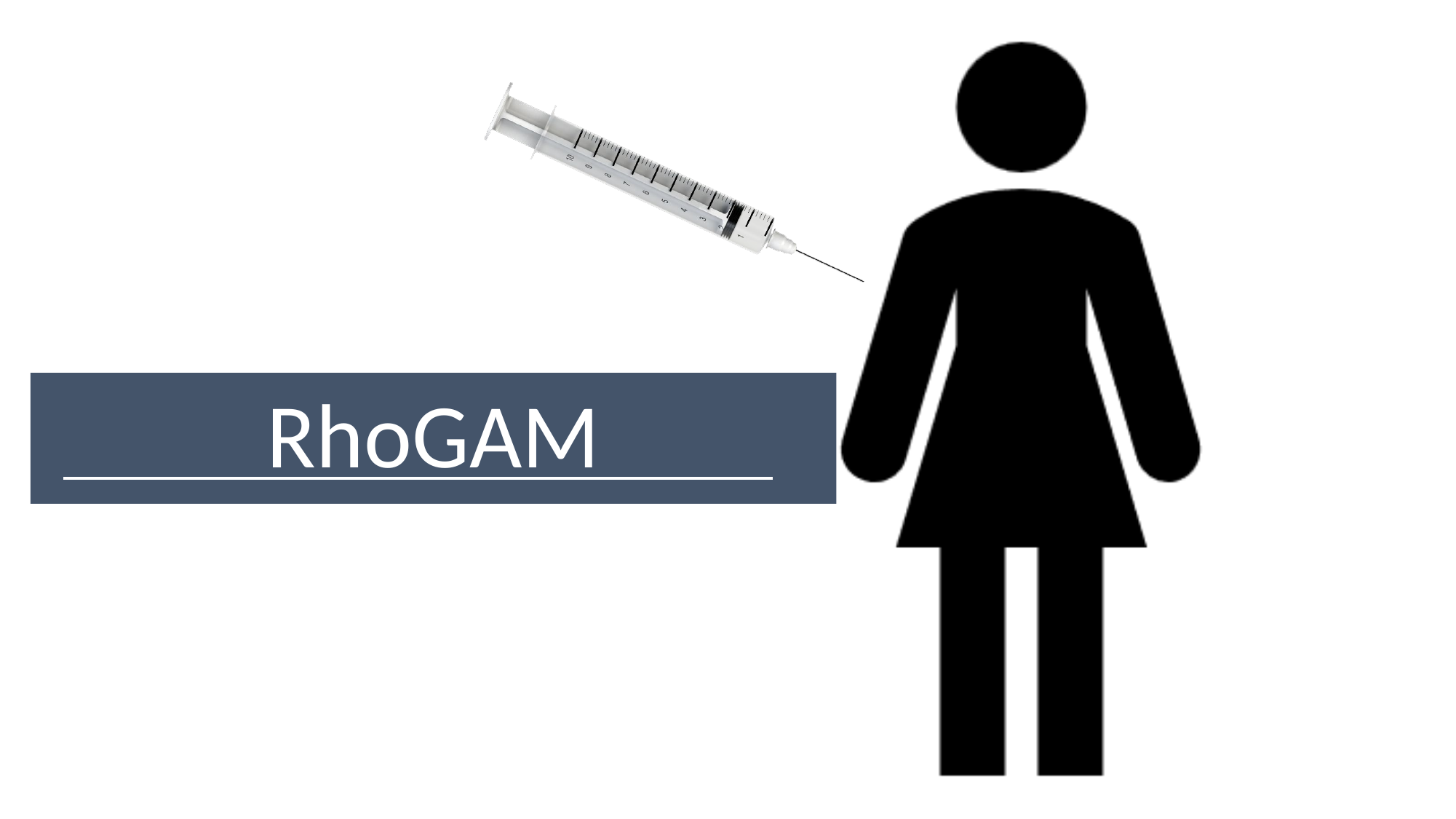

RhoGAM

## Slide 13
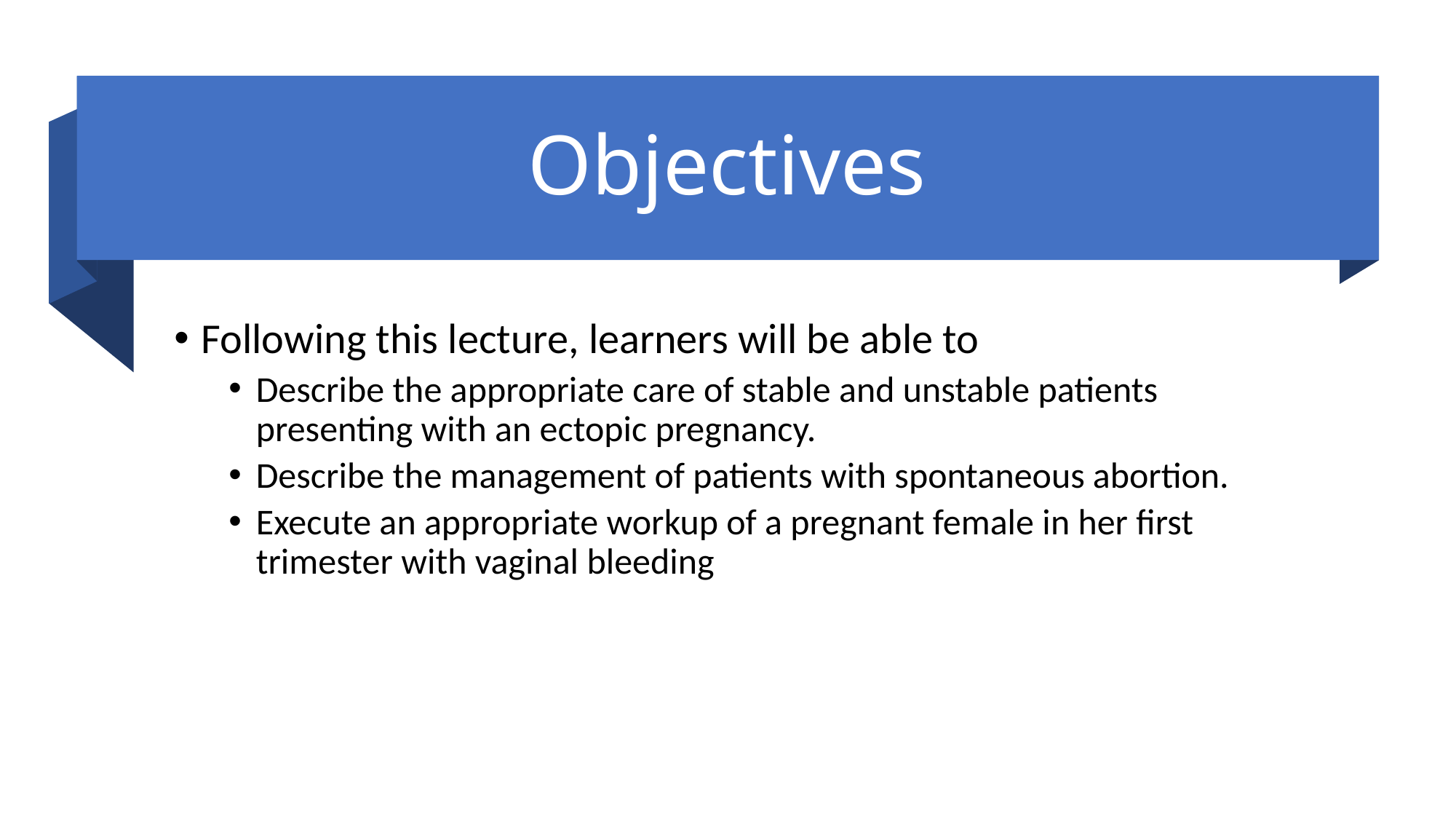

# Objectives
Following this lecture, learners will be able to
Describe the appropriate care of stable and unstable patients presenting with an ectopic pregnancy.
Describe the management of patients with spontaneous abortion.
Execute an appropriate workup of a pregnant female in her first trimester with vaginal bleeding

## Slide 14
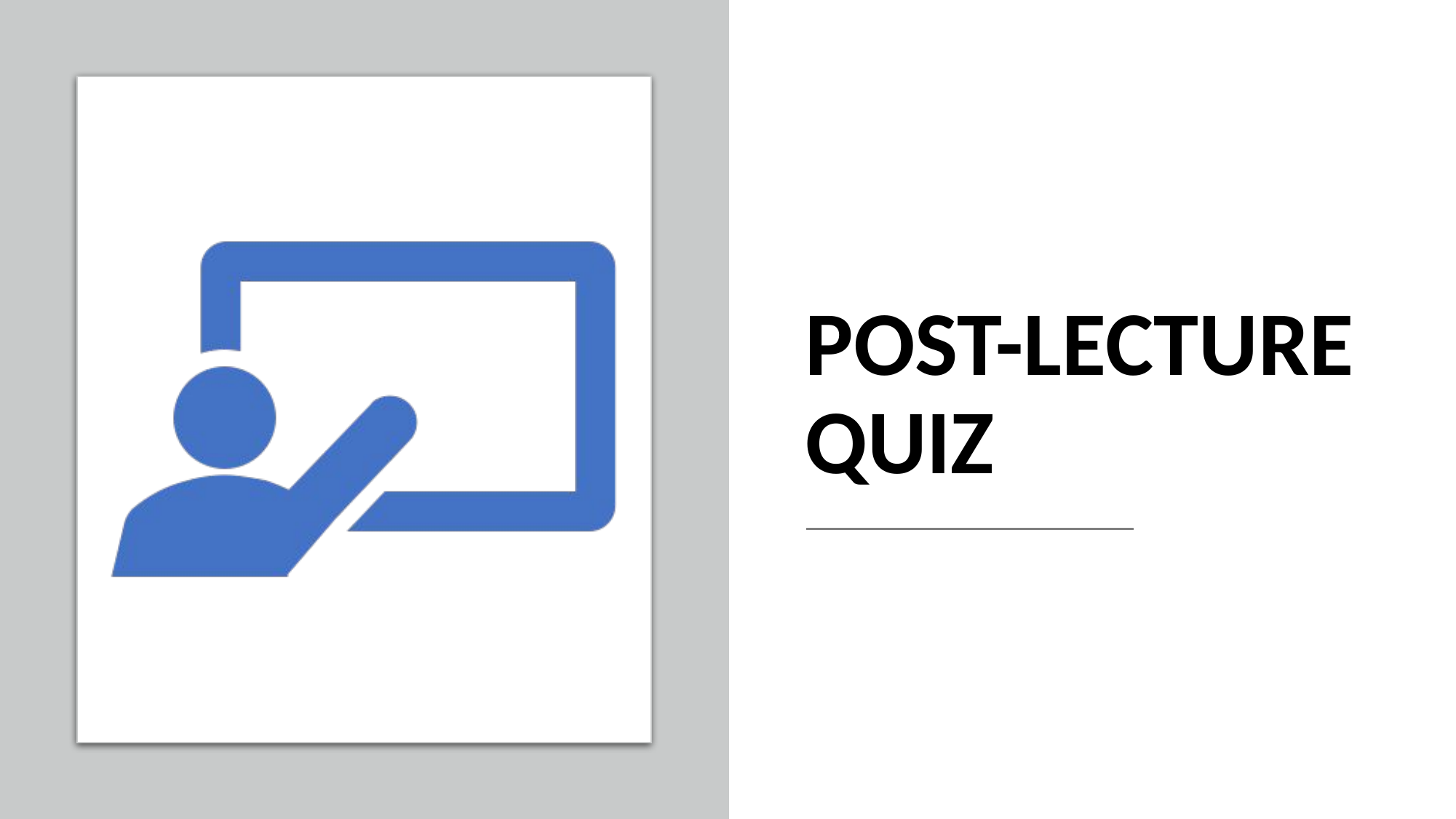

# POST-LECTURE QUIZ

## Slide 15
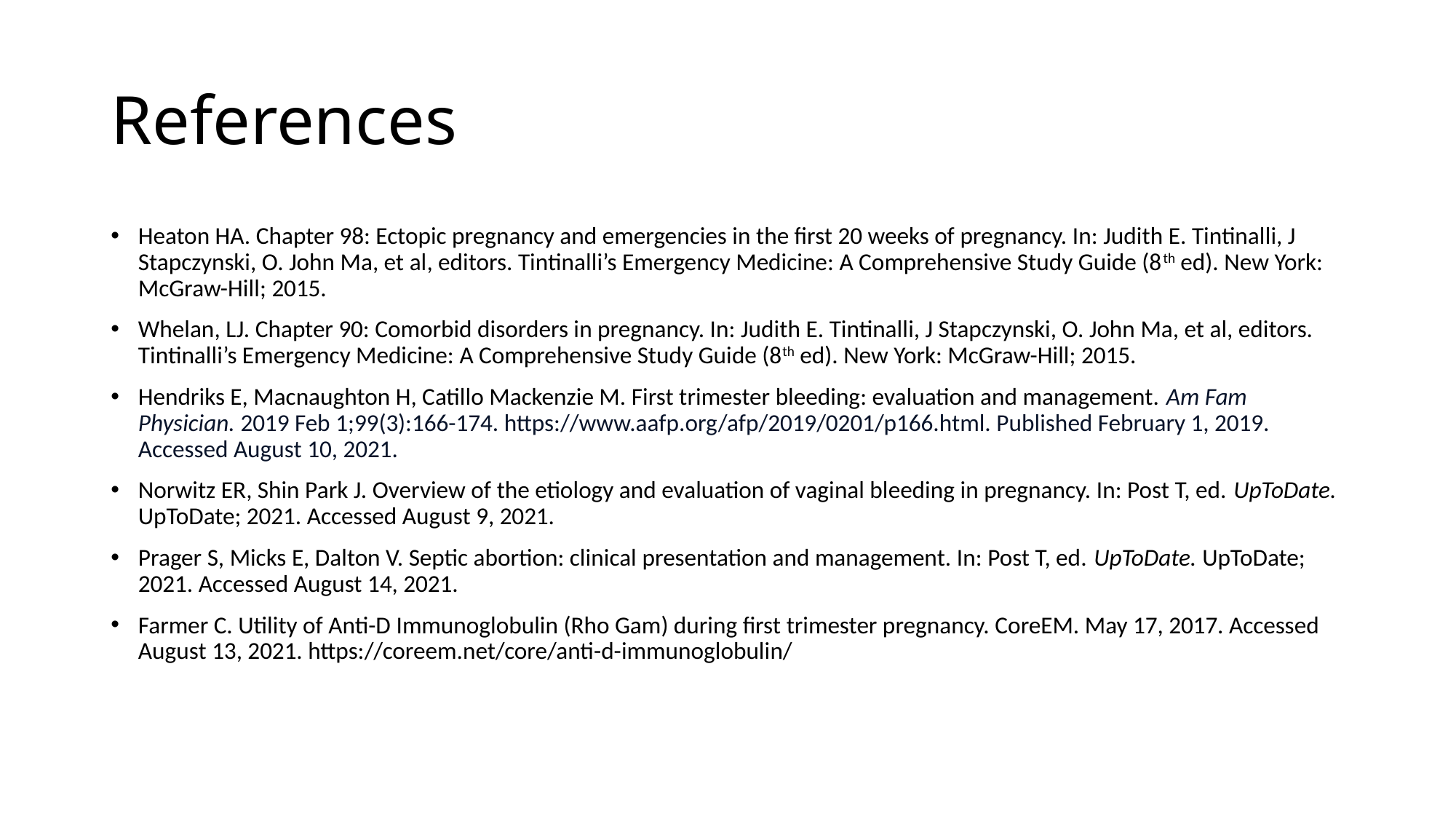

# References
Heaton HA. Chapter 98: Ectopic pregnancy and emergencies in the first 20 weeks of pregnancy. In: Judith E. Tintinalli, J Stapczynski, O. John Ma, et al, editors. Tintinalli’s Emergency Medicine: A Comprehensive Study Guide (8th ed). New York: McGraw-Hill; 2015.
Whelan, LJ. Chapter 90: Comorbid disorders in pregnancy. In: Judith E. Tintinalli, J Stapczynski, O. John Ma, et al, editors. Tintinalli’s Emergency Medicine: A Comprehensive Study Guide (8th ed). New York: McGraw-Hill; 2015.
Hendriks E, Macnaughton H, Catillo Mackenzie M. First trimester bleeding: evaluation and management. Am Fam Physician. 2019 Feb 1;99(3):166-174. https://www.aafp.org/afp/2019/0201/p166.html. Published February 1, 2019. Accessed August 10, 2021.
Norwitz ER, Shin Park J. Overview of the etiology and evaluation of vaginal bleeding in pregnancy. In: Post T, ed. UpToDate. UpToDate; 2021. Accessed August 9, 2021.
Prager S, Micks E, Dalton V. Septic abortion: clinical presentation and management. In: Post T, ed. UpToDate. UpToDate; 2021. Accessed August 14, 2021.
Farmer C. Utility of Anti-D Immunoglobulin (Rho Gam) during first trimester pregnancy. CoreEM. May 17, 2017. Accessed August 13, 2021. https://coreem.net/core/anti-d-immunoglobulin/
