# Supplementary material for: Emergency Medicine Obstetrics and Gynecology: A Case-Based Curriculum for Residents
Source: MedEdPORTAL. 2023 Aug 11;19:11330. doi: 10.15766/mep_2374-8265.11330 (PMC10415535; doi:10.15766/mep_2374-8265.11330)
Supplement: Supplementary file 1 — Ectopic Pregnancy and Emergencies in the First 20 Weeks.pptxPregnancy Emergencies After 20 Weeks.pptxDelivery Emergencies.pptxPelvic Pain in the Nonpregnant Patient.pptxVaginitis, Cervicitis, and PID.pptxAbnormal Uterine Bleeding.pptxLabor and Perimortem C-Section.pptxSession Review Questions.docxPrecurriculum Survey.docxPostcurriculum Survey.docx [file mep_2374-8265.11330-s001.zip › B. Pregnancy Emergencies After 20 Weeks.pptx]

## Slide 1
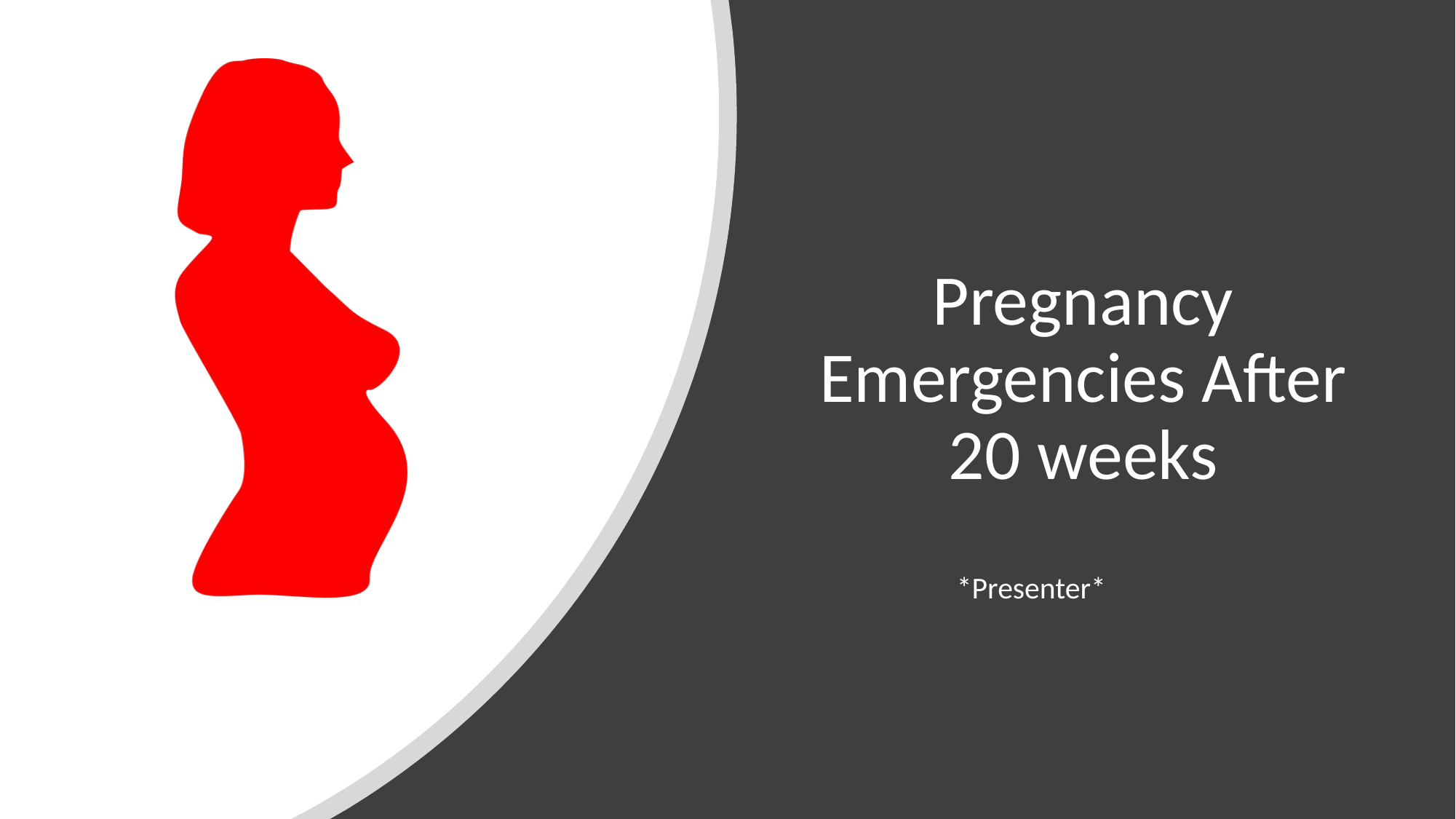

# Pregnancy Emergencies After 20 weeks
*Presenter*

## Slide 2
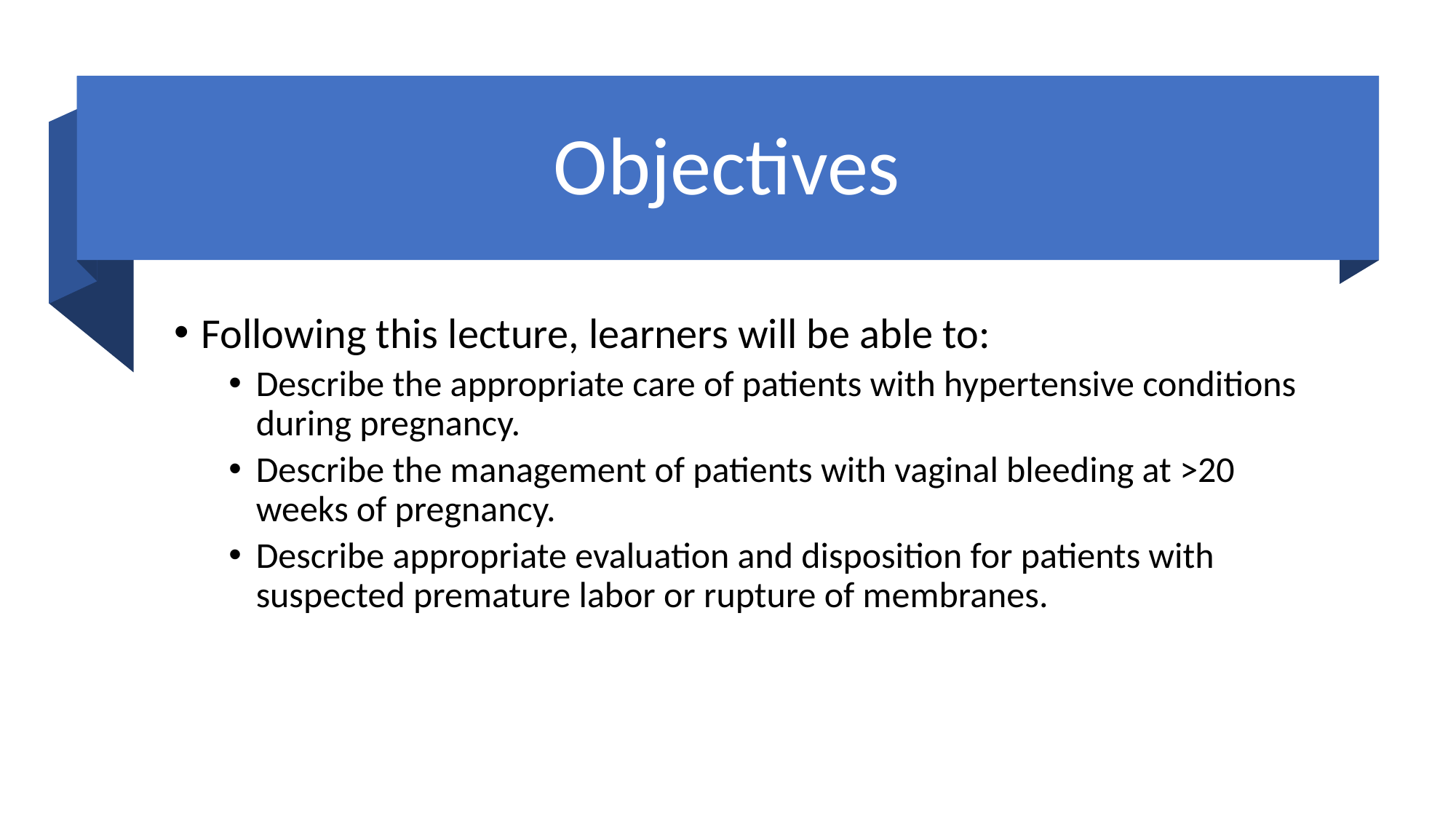

# Objectives
Following this lecture, learners will be able to:
Describe the appropriate care of patients with hypertensive conditions during pregnancy.
Describe the management of patients with vaginal bleeding at >20 weeks of pregnancy.
Describe appropriate evaluation and disposition for patients with suspected premature labor or rupture of membranes.

## Slide 3
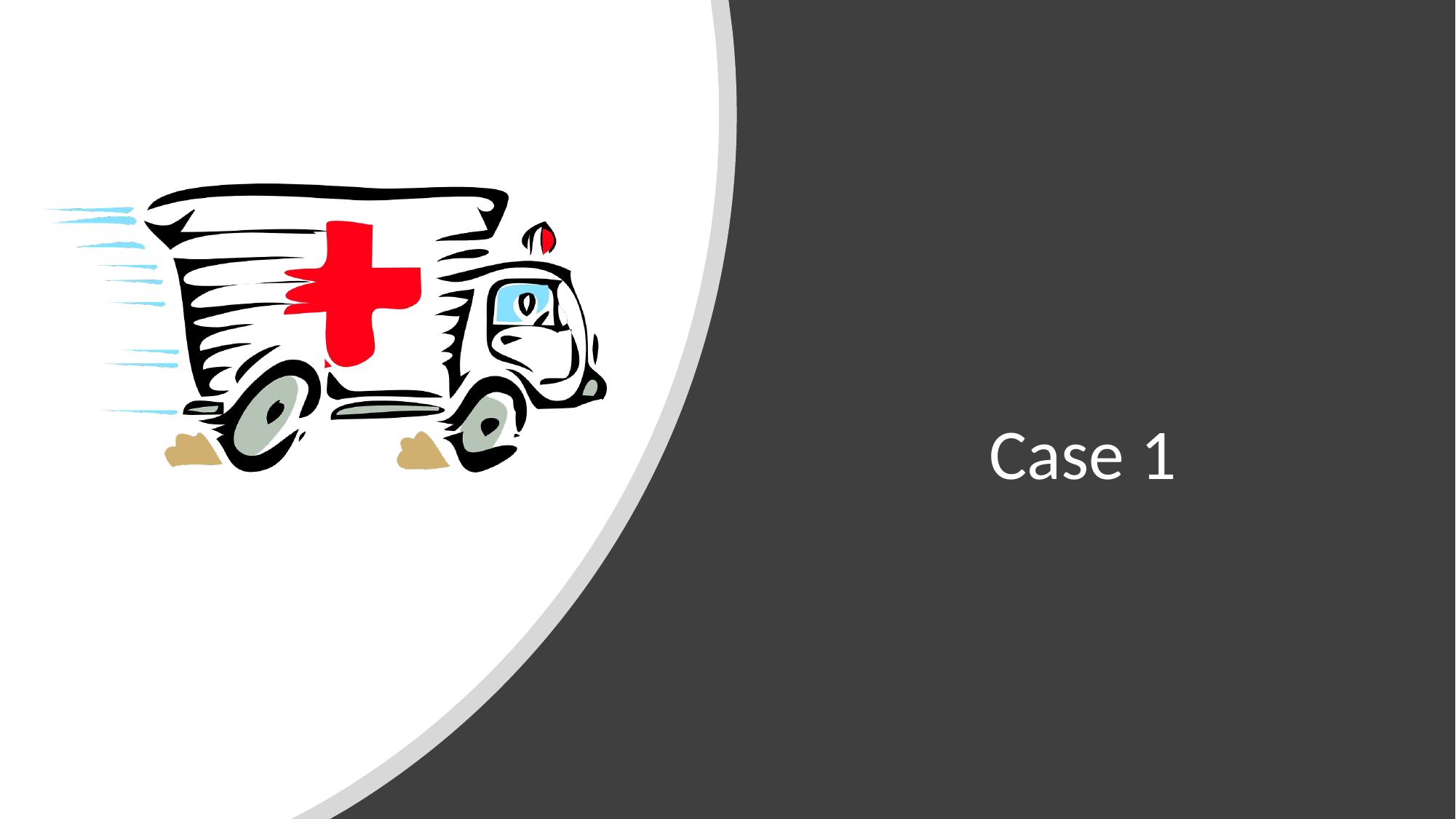

# Case 1

## Slide 4
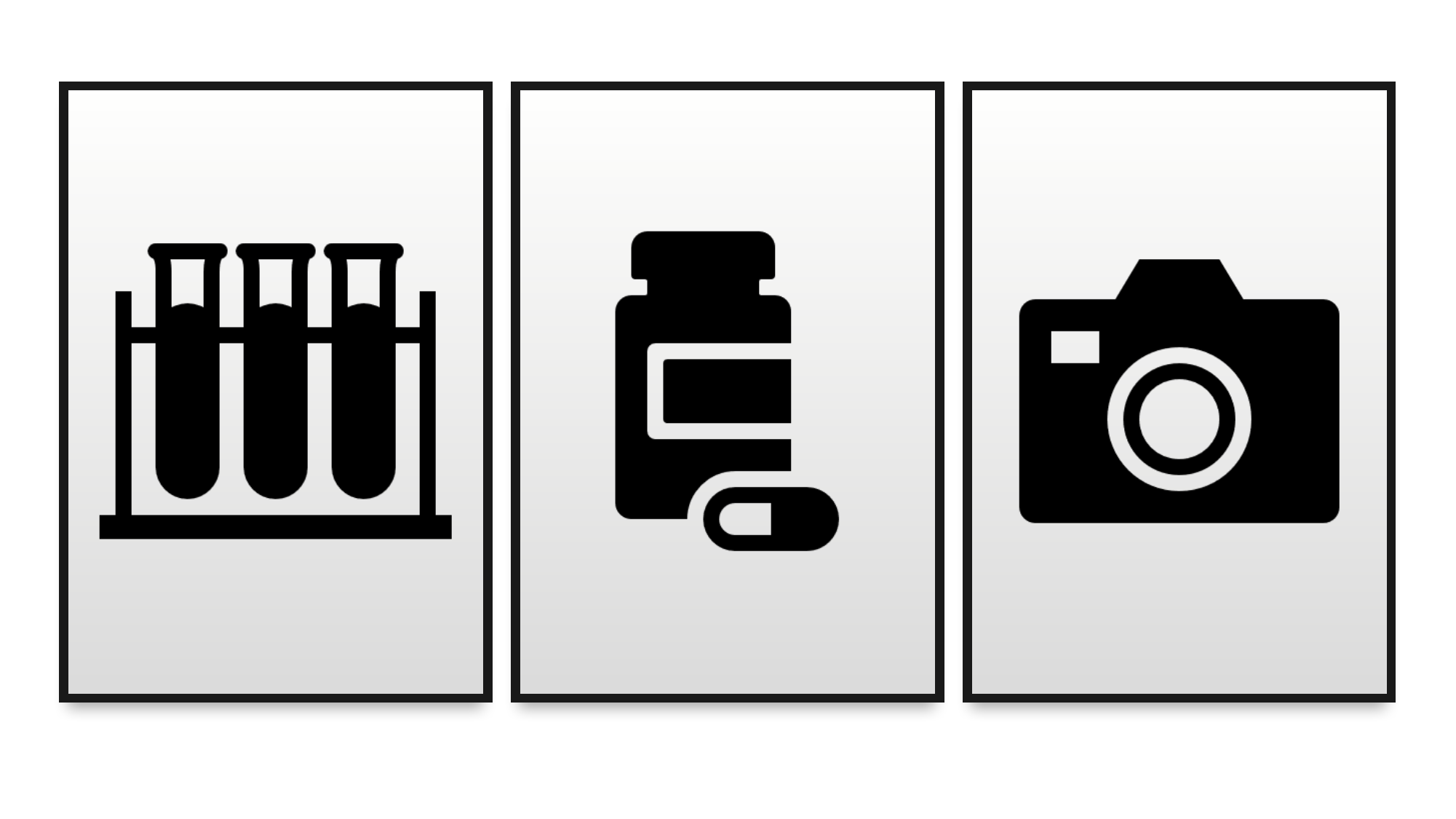

## Slide 5
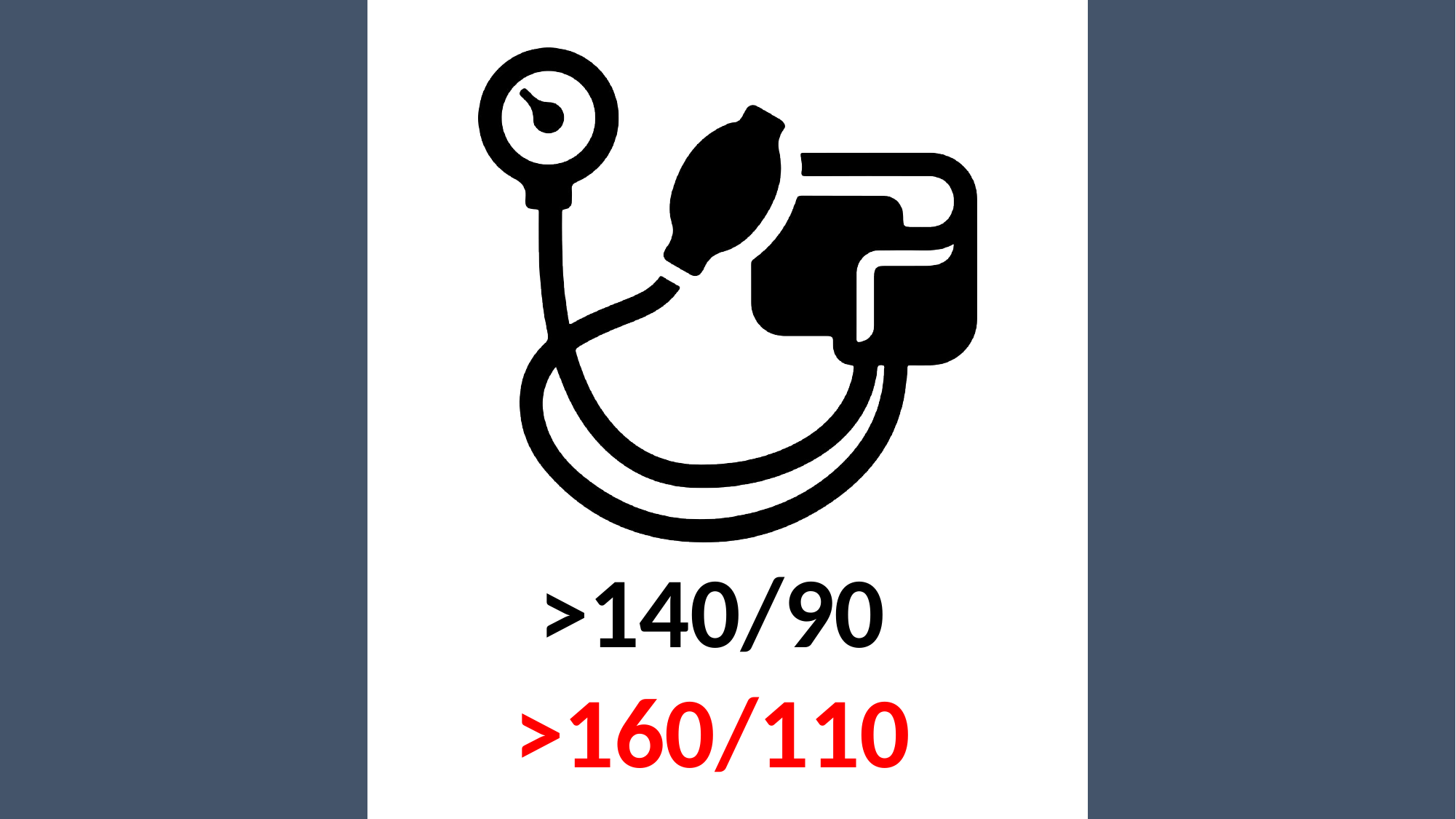

>140/90
>160/110

## Slide 6
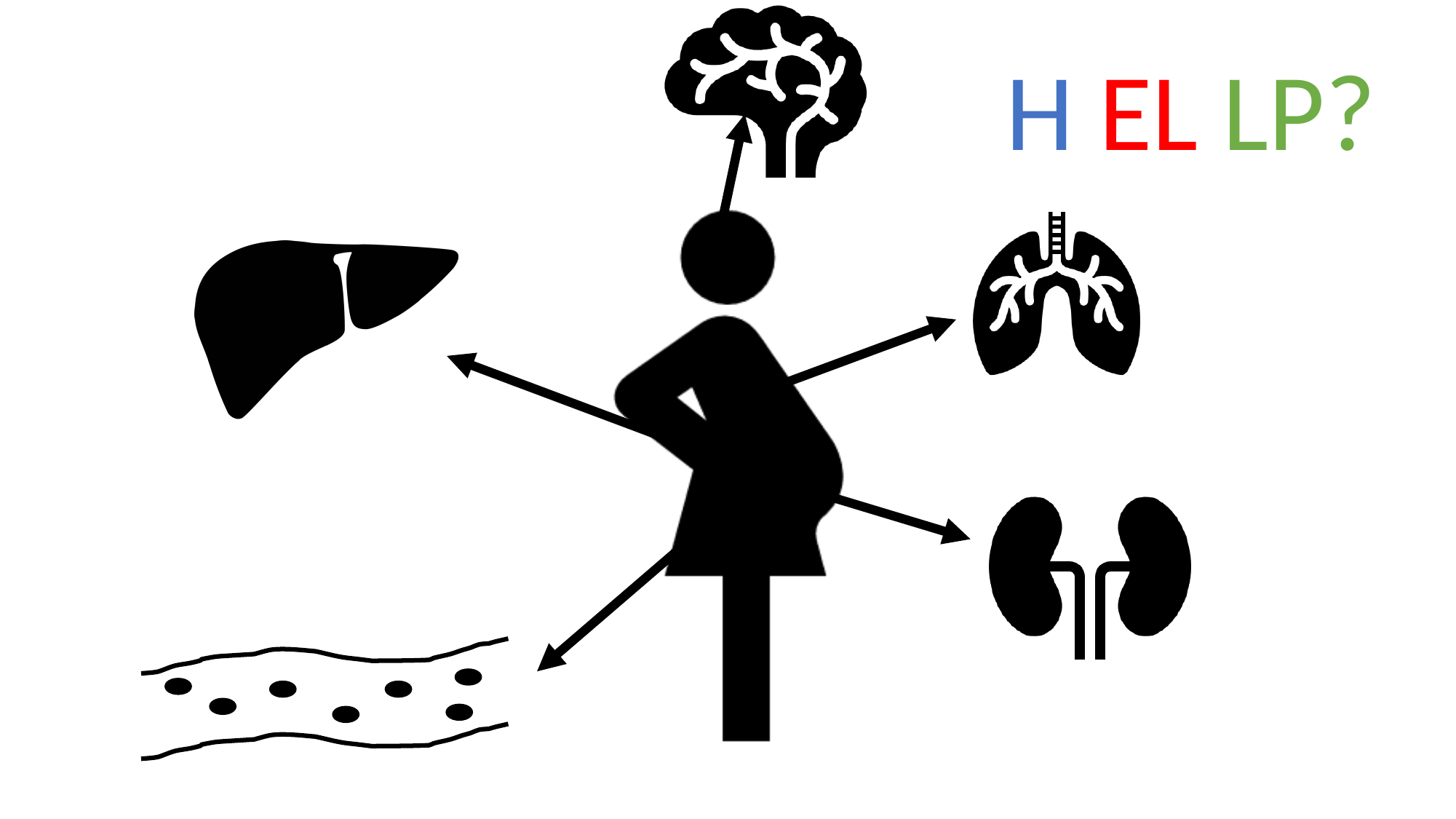

H EL LP?

## Slide 7
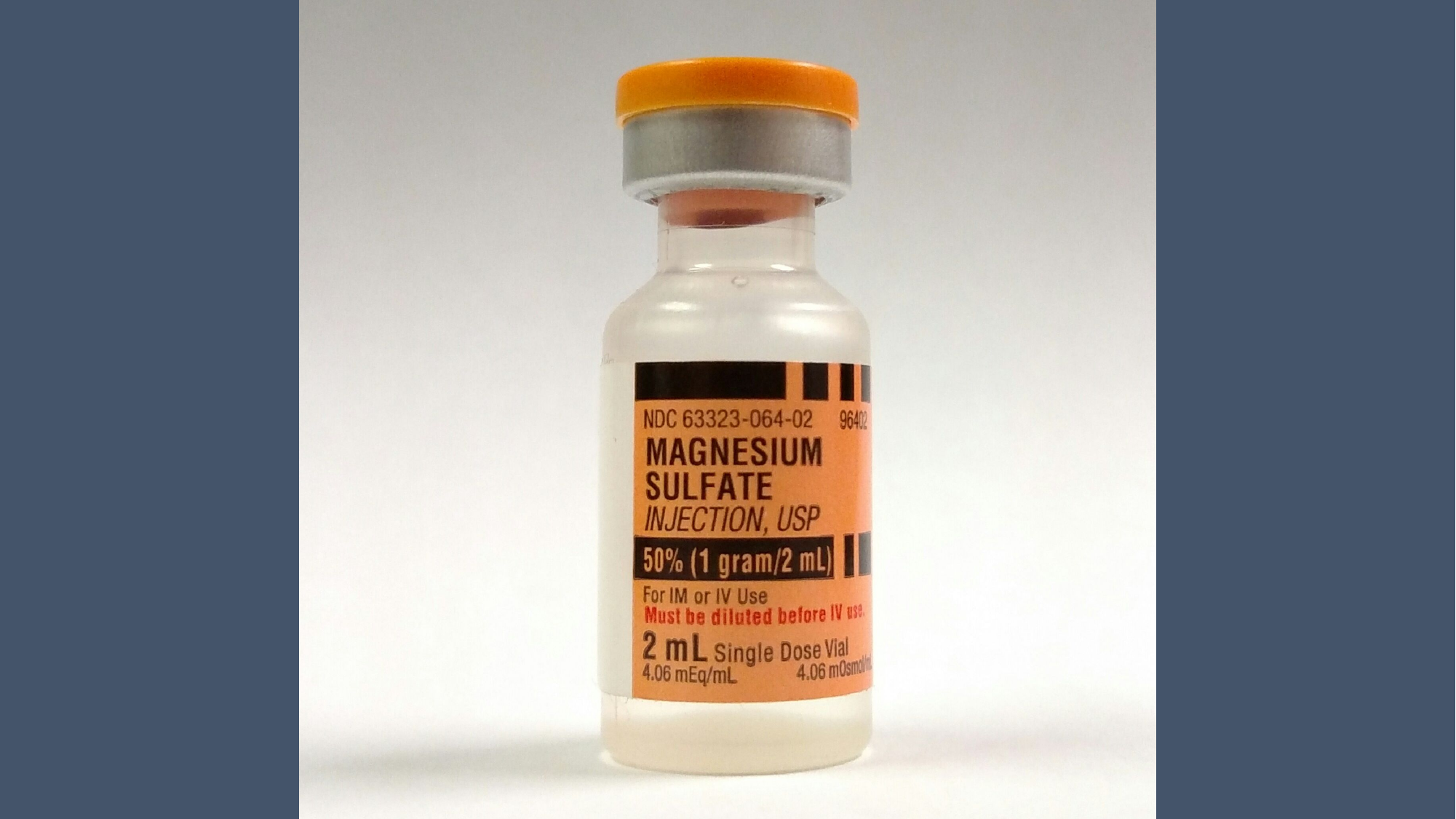

## Slide 8
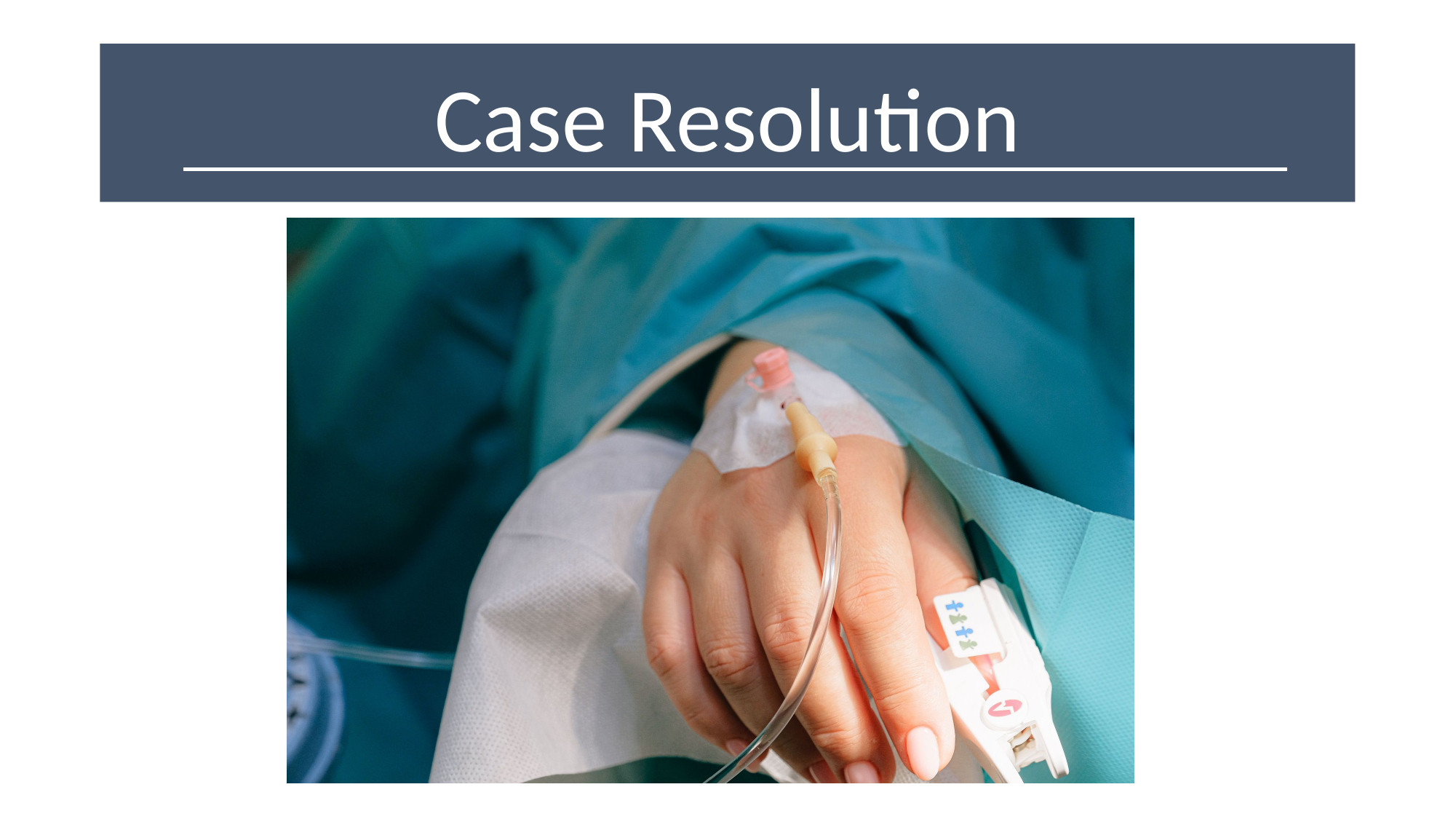

# Case Resolution

## Slide 9
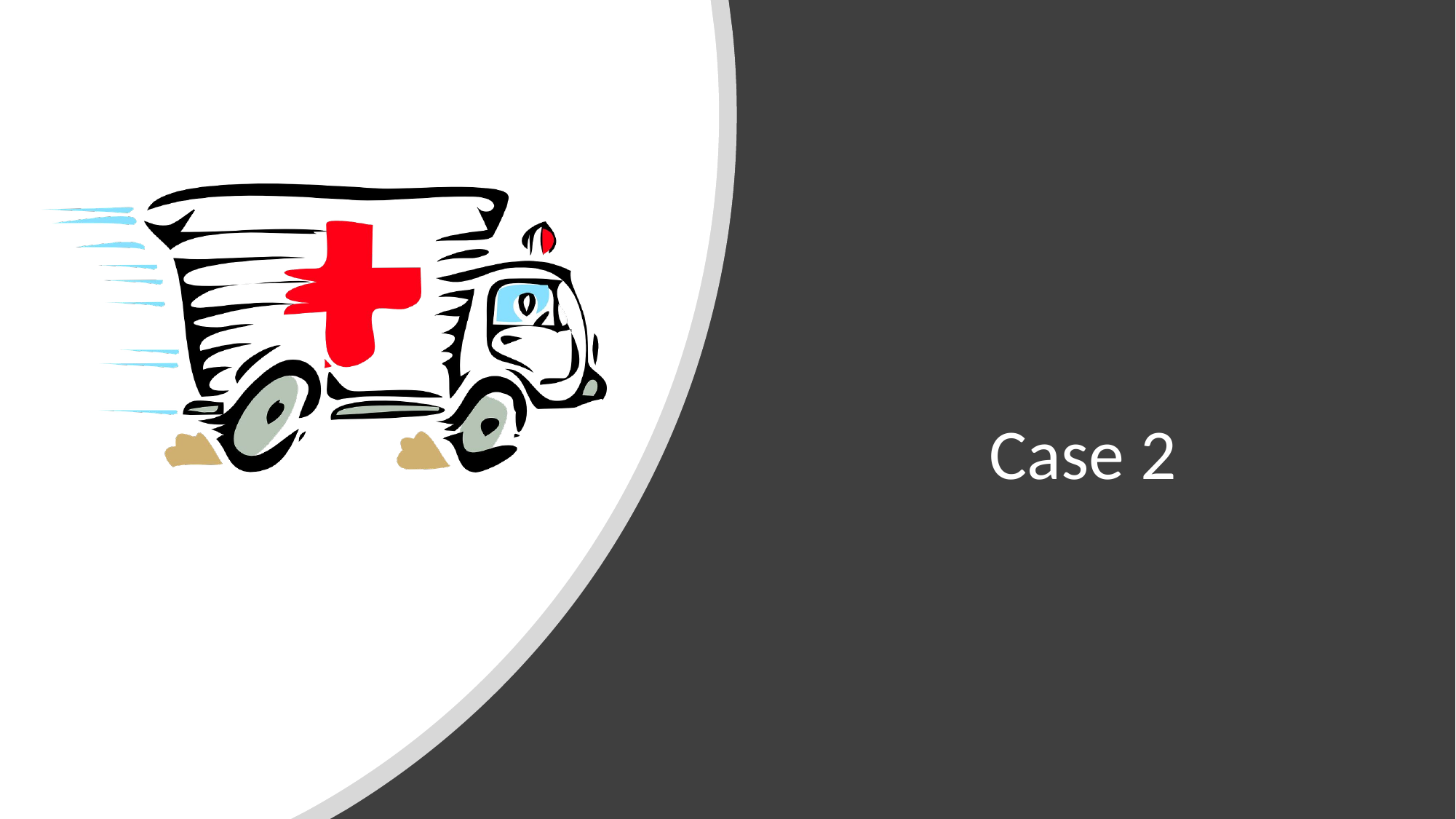

# Case 2

## Slide 10
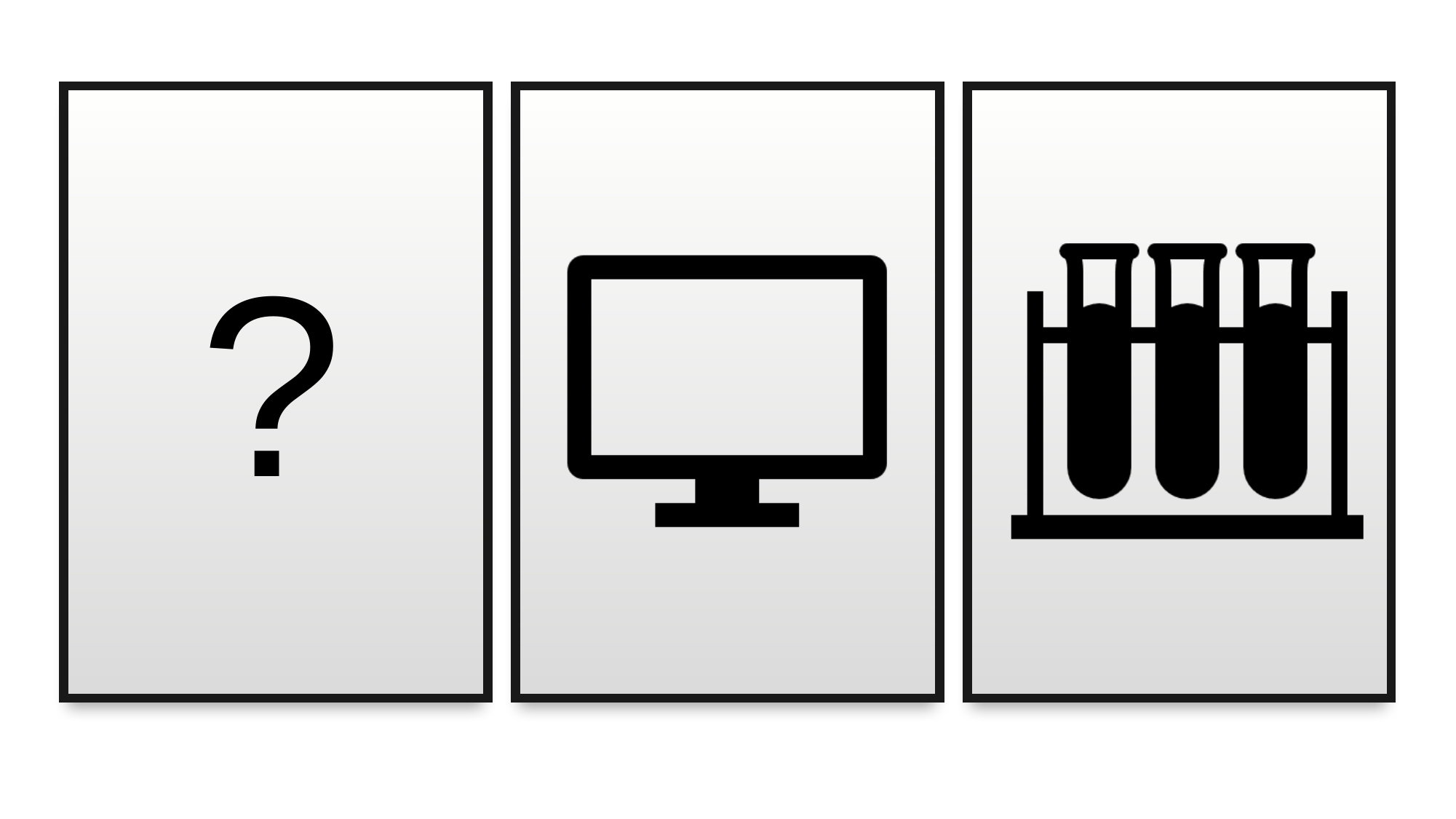

?

## Slide 11
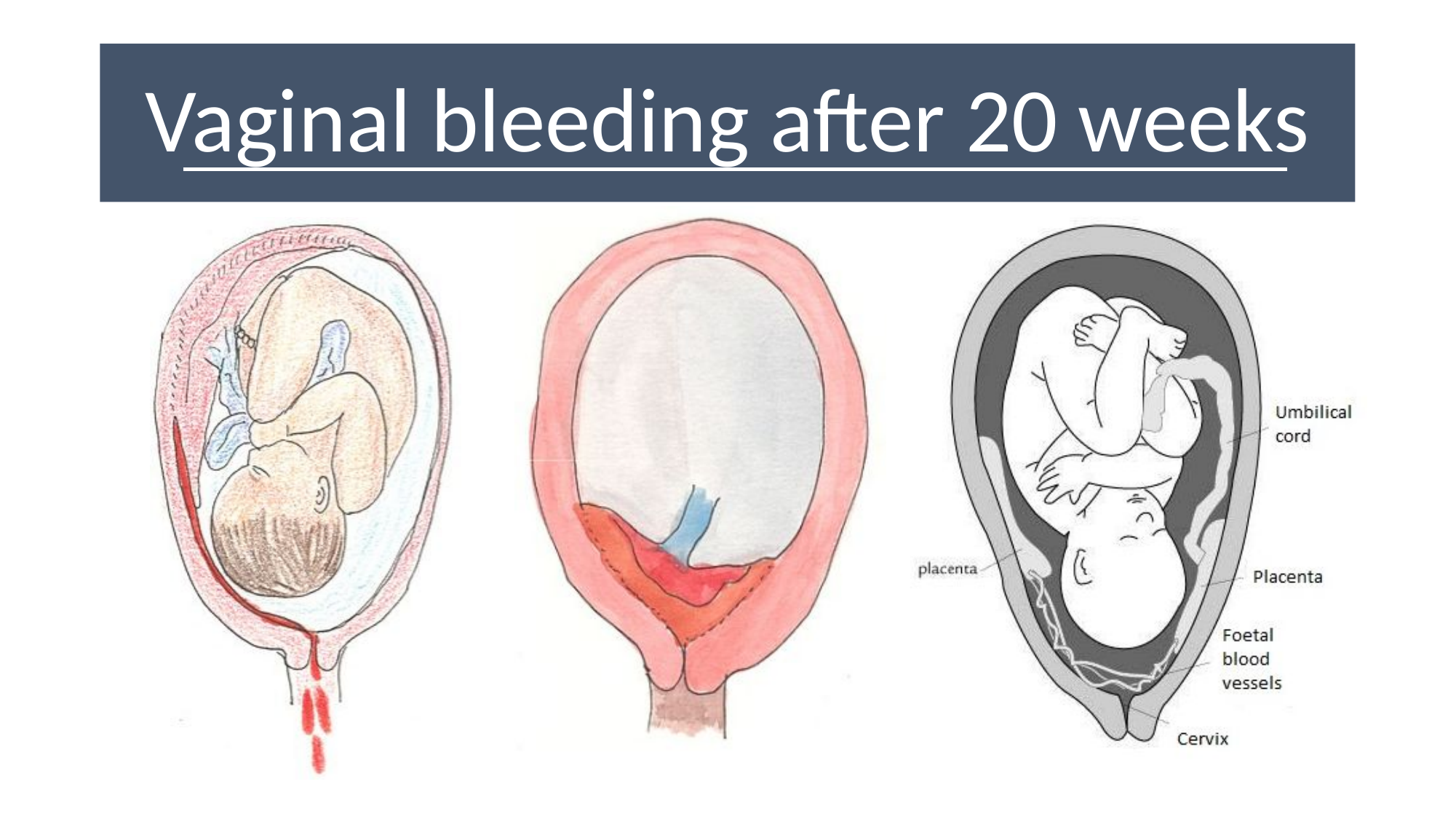

# Vaginal bleeding after 20 weeks

## Slide 12
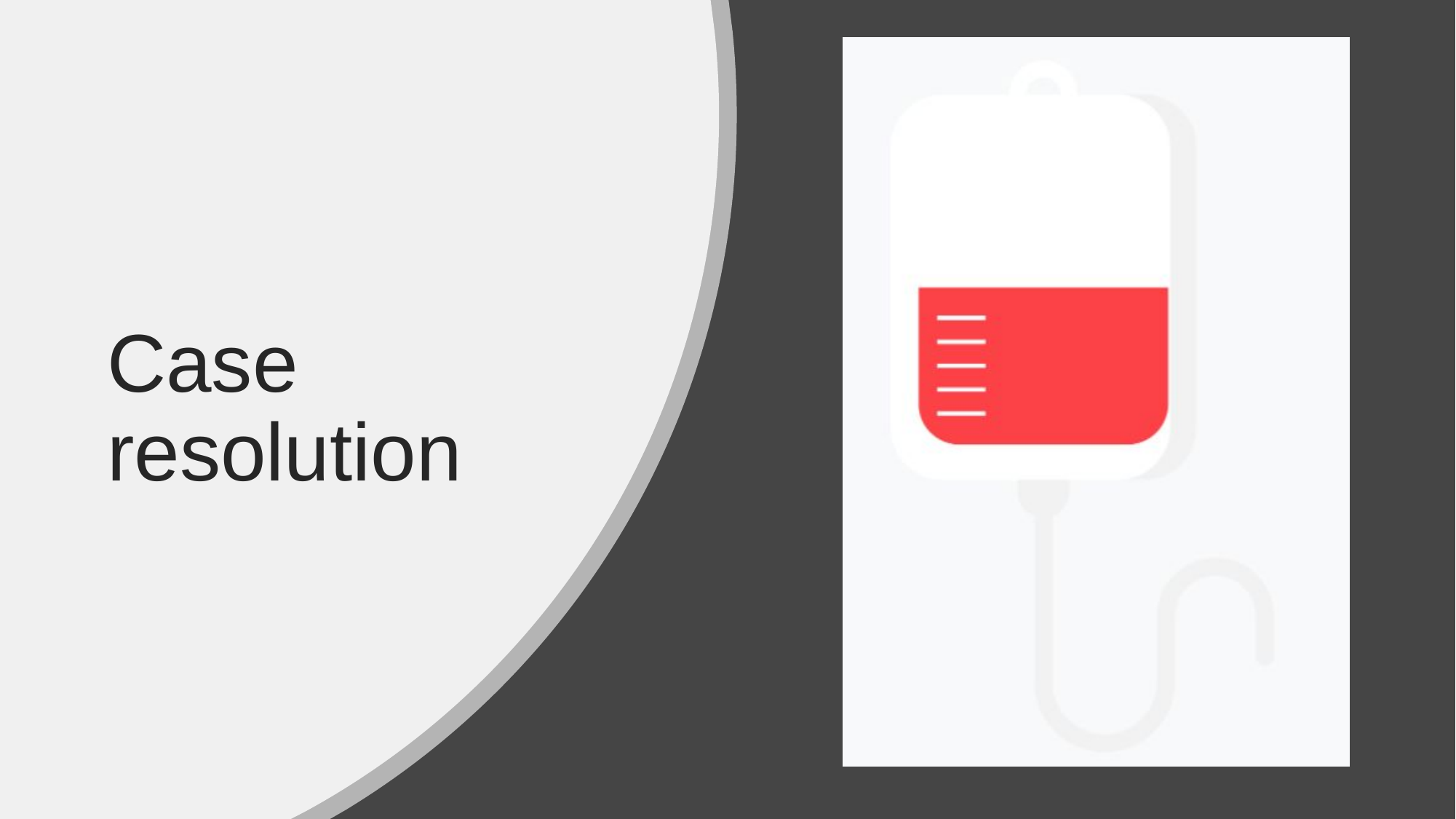

# Case resolution

## Slide 13
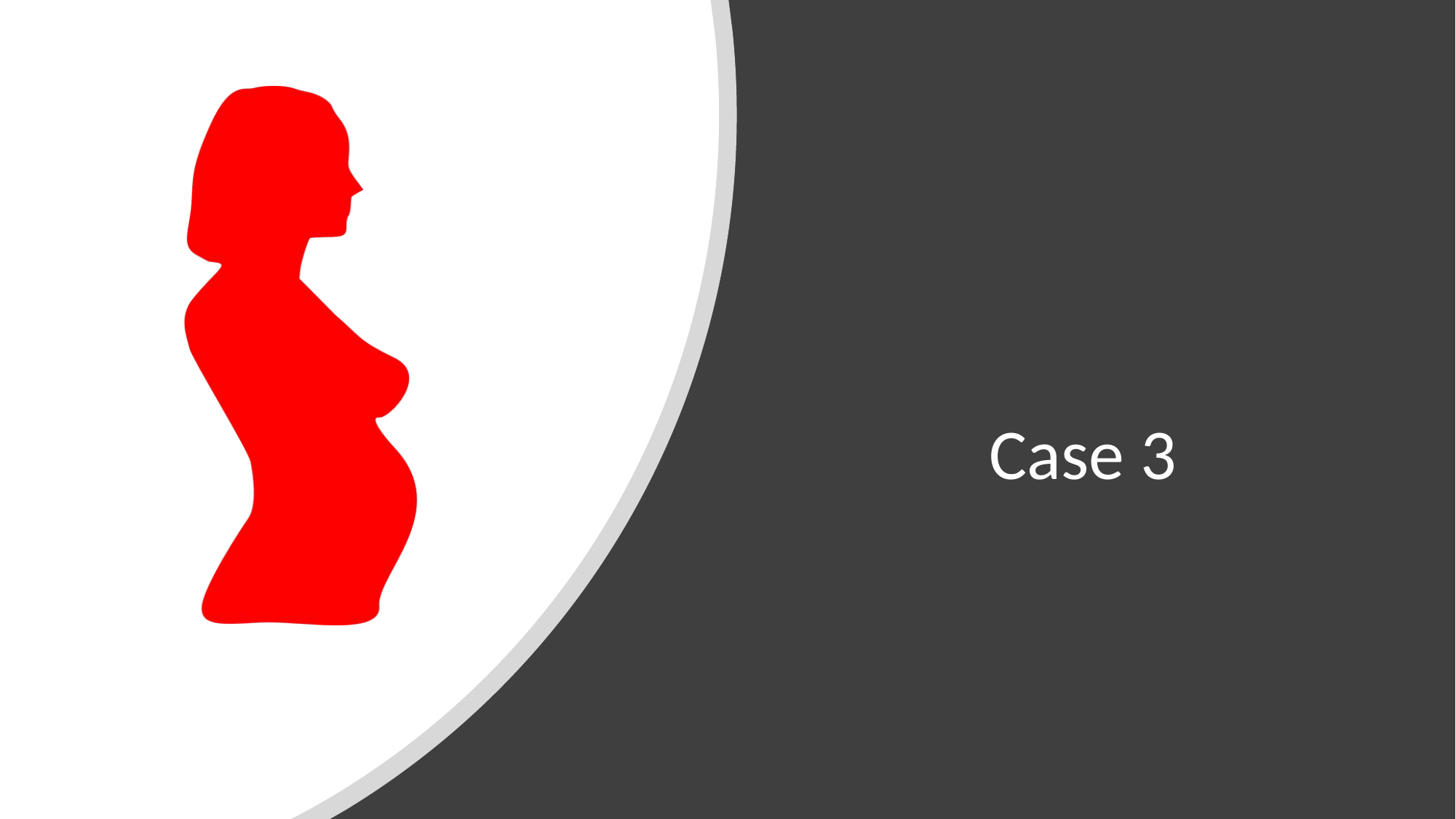

# Case 3

## Slide 14
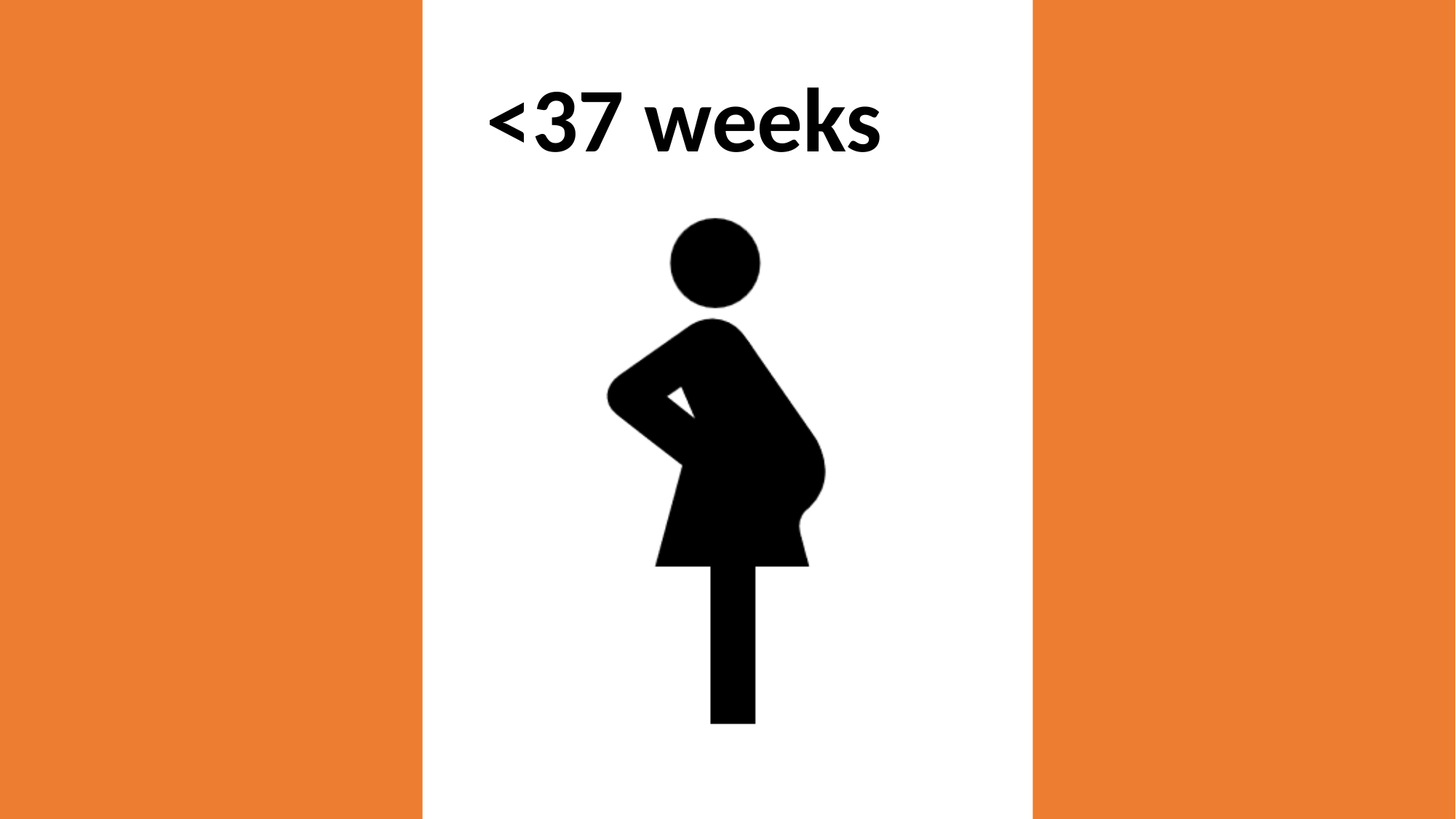

# <37 weeks

## Slide 15
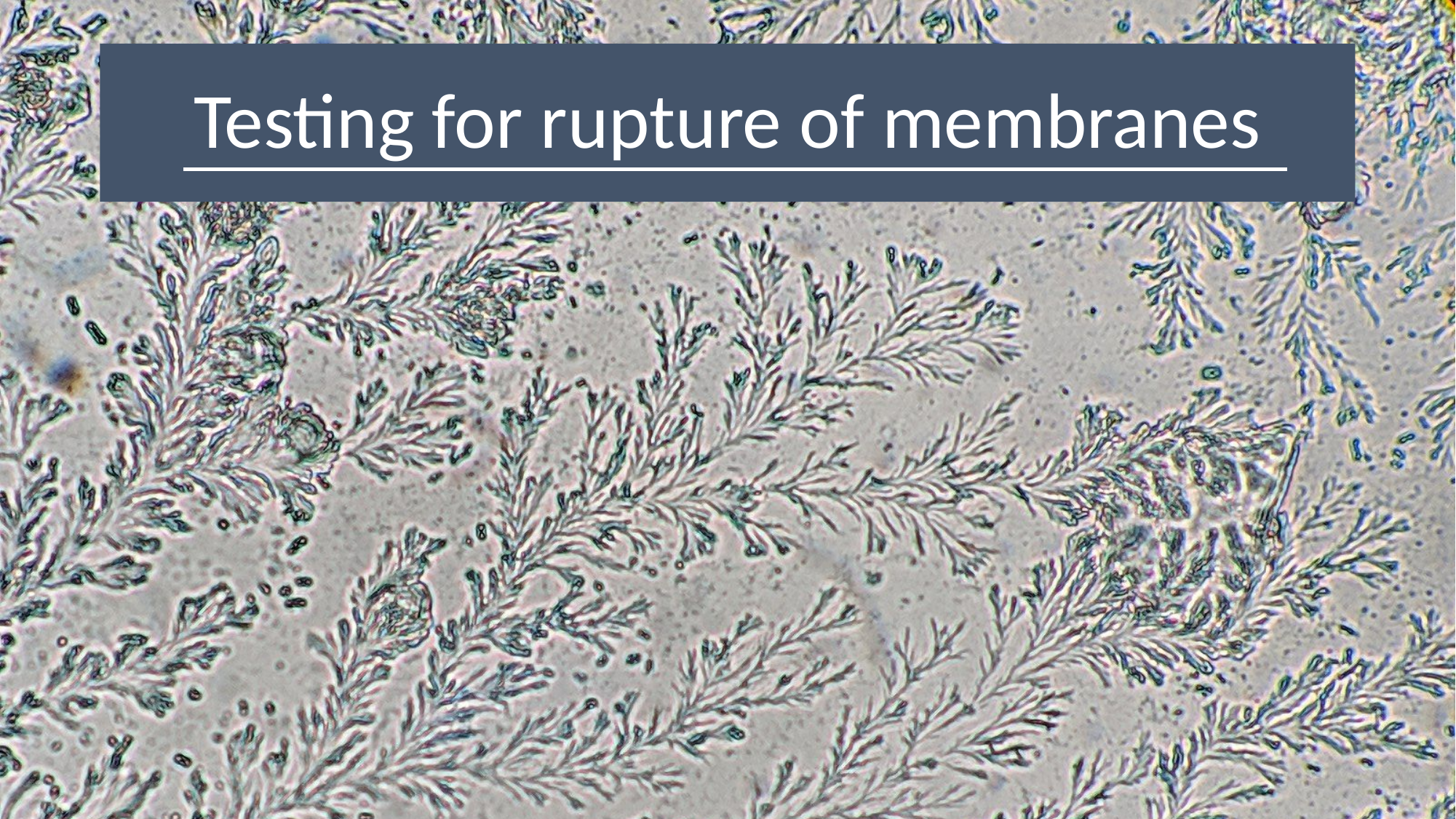

# Premature Rupture of Membranes
Testing for rupture of membranes

## Slide 16
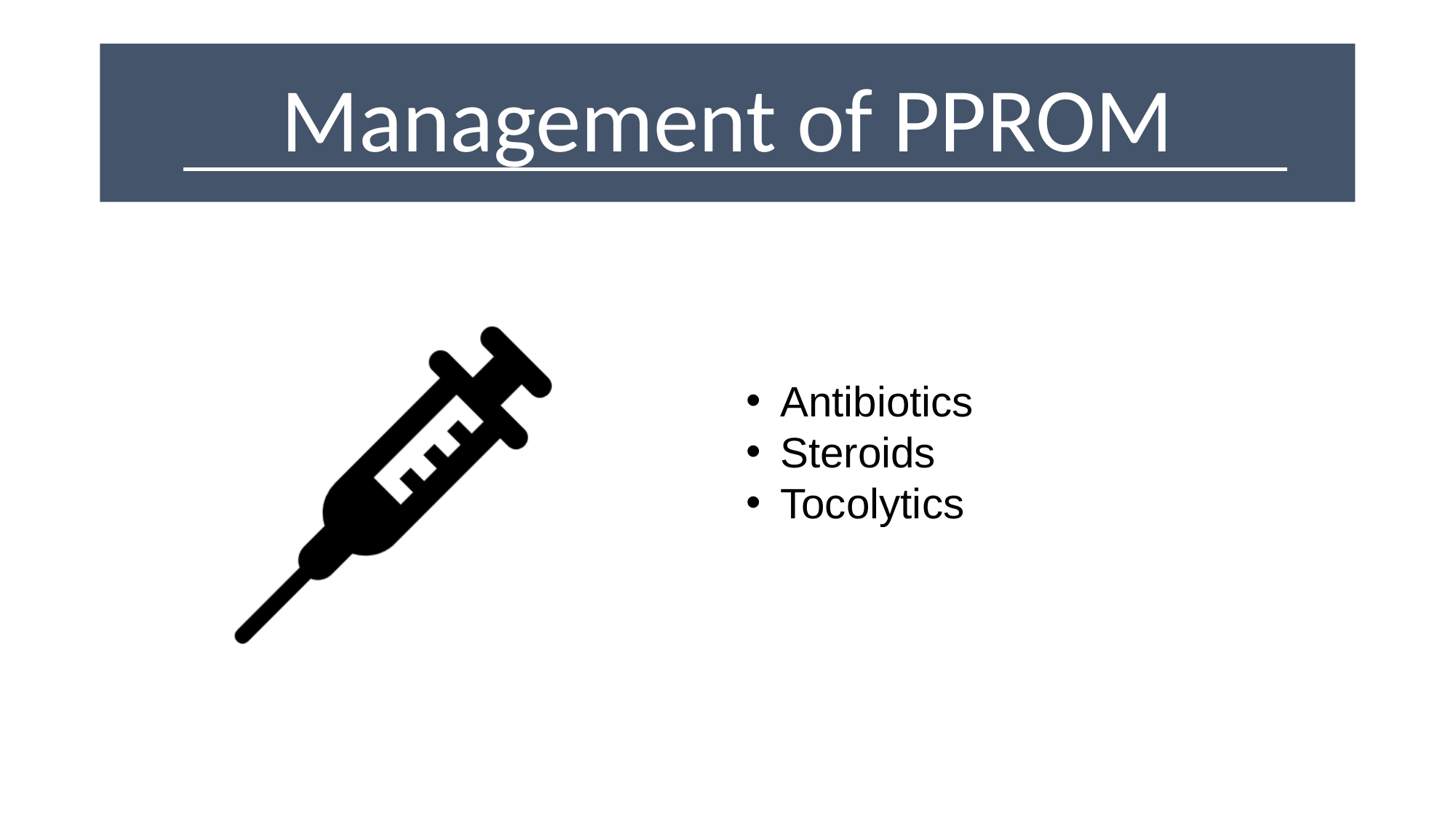

# Management of PPROM
Antibiotics
Steroids
Tocolytics

## Slide 17
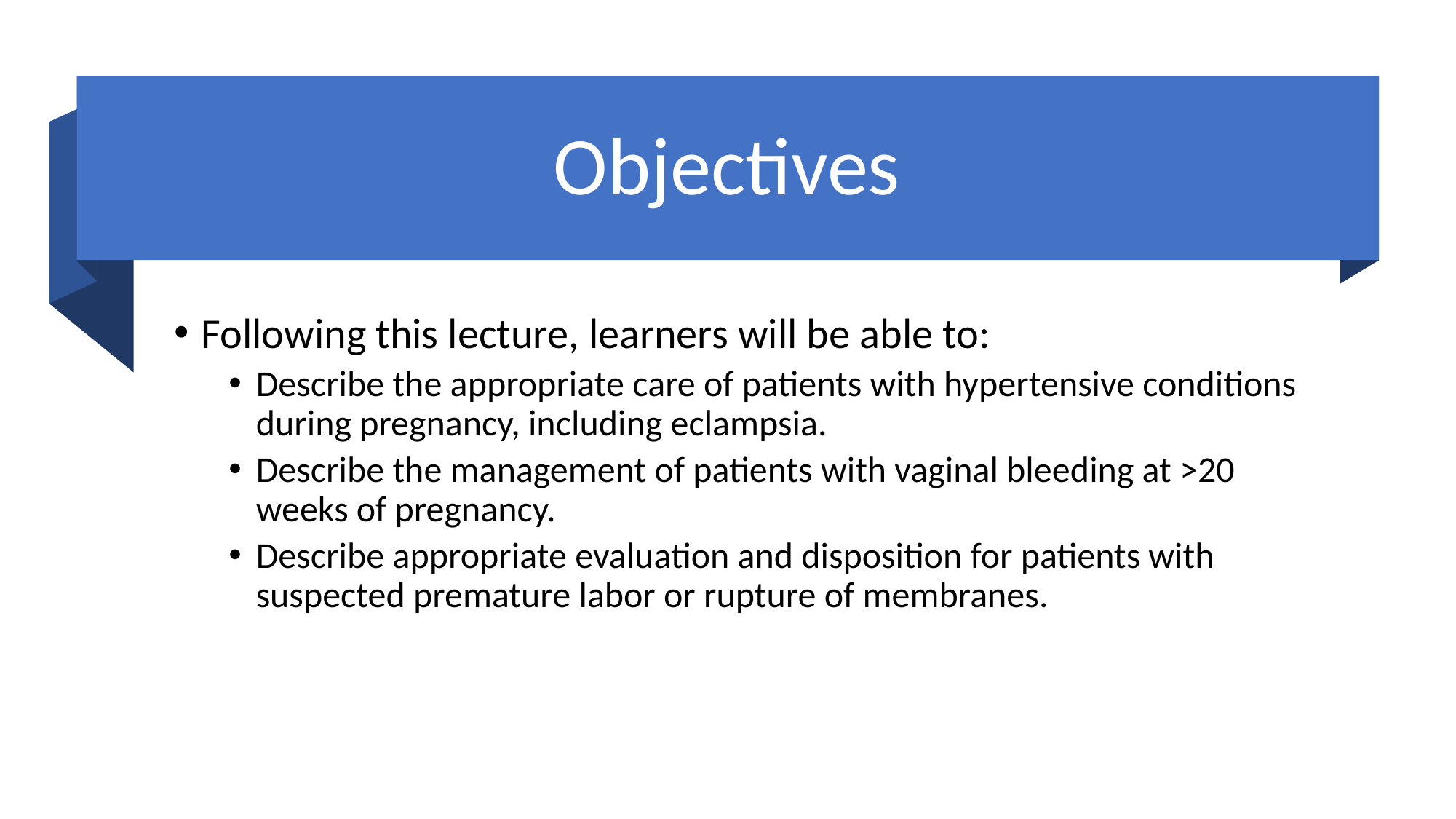

# Objectives
Following this lecture, learners will be able to:
Describe the appropriate care of patients with hypertensive conditions during pregnancy, including eclampsia.
Describe the management of patients with vaginal bleeding at >20 weeks of pregnancy.
Describe appropriate evaluation and disposition for patients with suspected premature labor or rupture of membranes.

## Slide 18
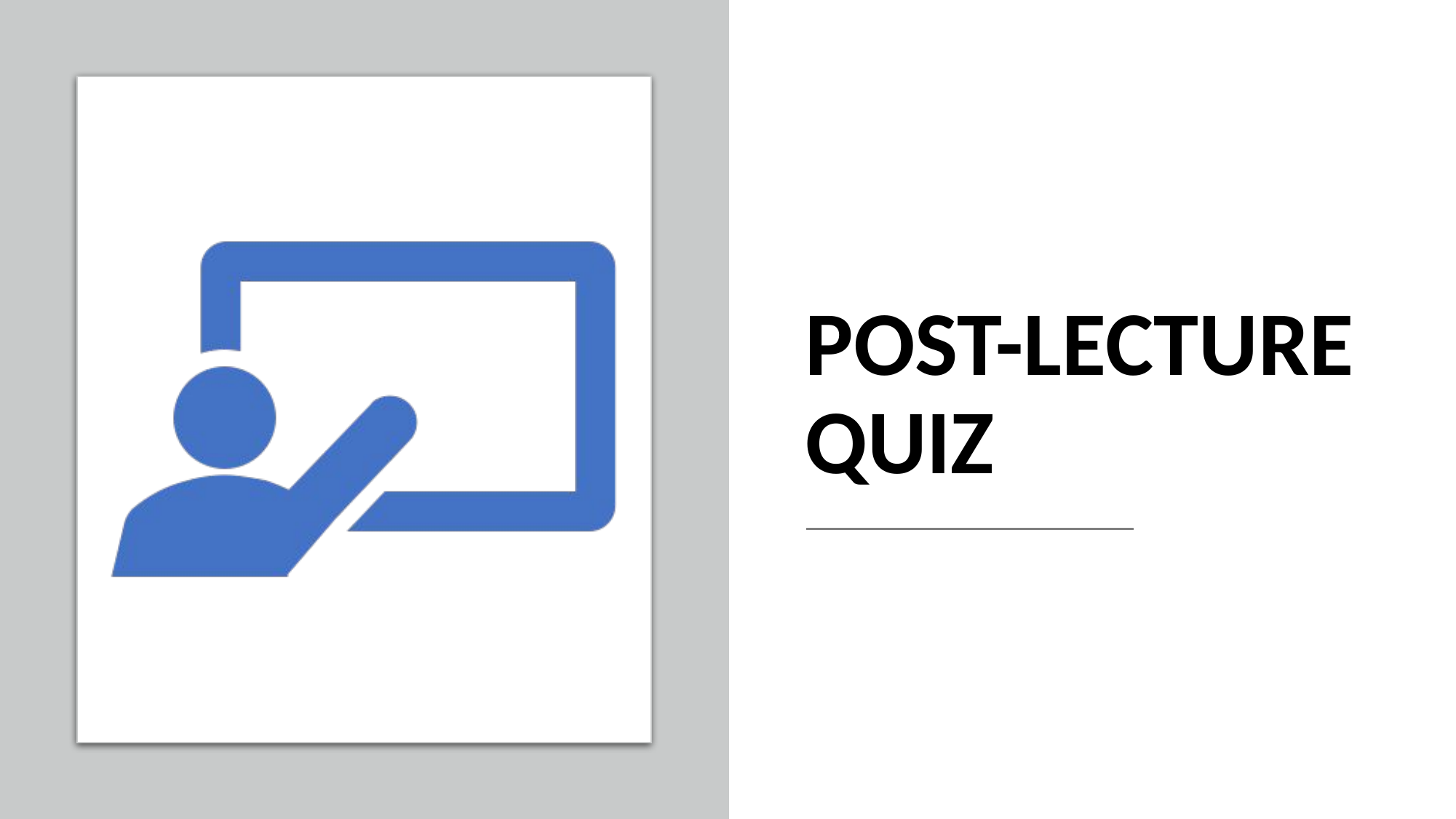

# POST-LECTURE QUIZ

## Slide 19
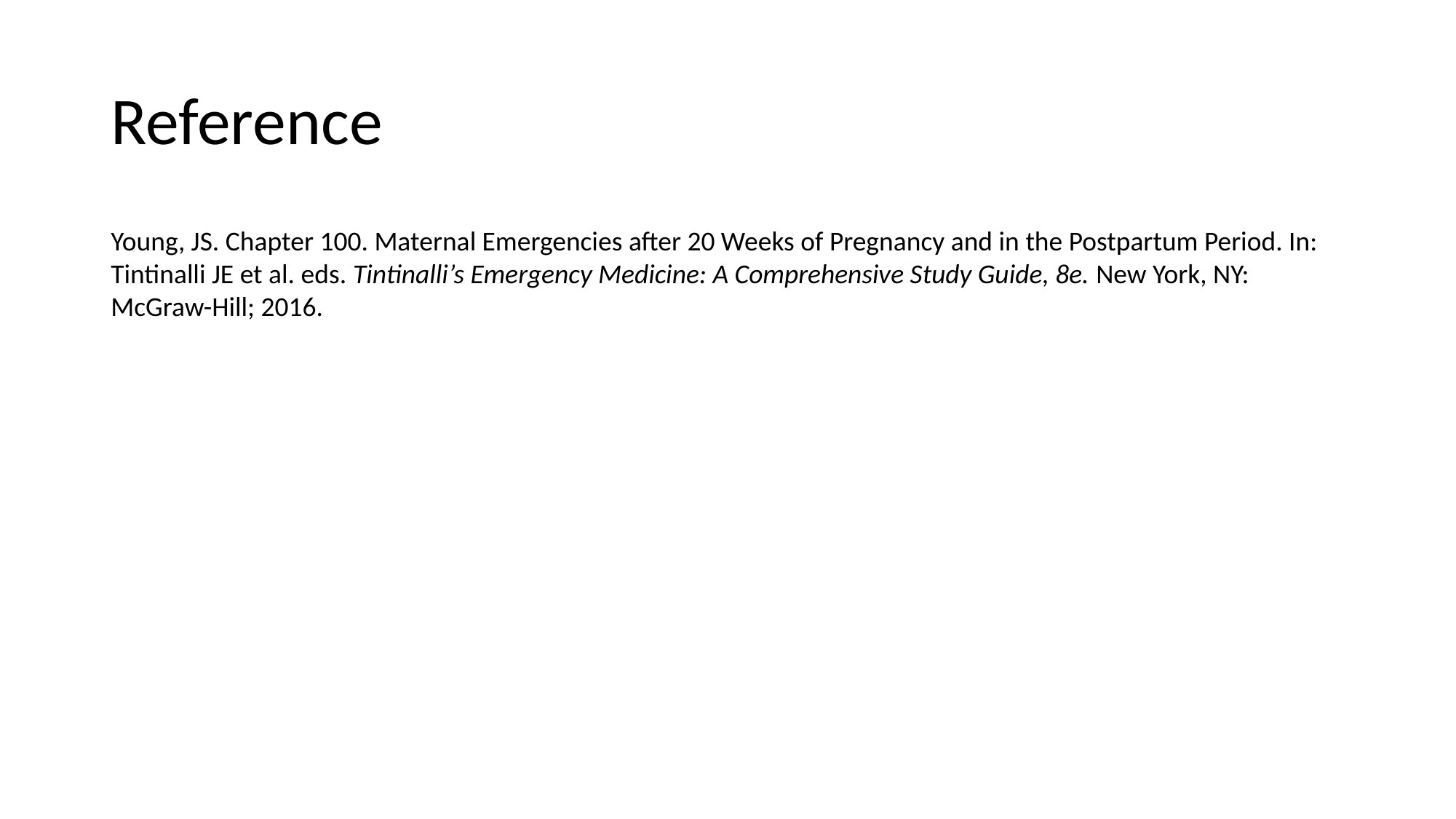

# Reference
Young, JS. Chapter 100. Maternal Emergencies after 20 Weeks of Pregnancy and in the Postpartum Period. In: Tintinalli JE et al. eds. Tintinalli’s Emergency Medicine: A Comprehensive Study Guide, 8e. New York, NY: McGraw-Hill; 2016.
