# Supplementary material for: Emergency Medicine Obstetrics and Gynecology: A Case-Based Curriculum for Residents
Source: MedEdPORTAL. 2023 Aug 11;19:11330. doi: 10.15766/mep_2374-8265.11330 (PMC10415535; doi:10.15766/mep_2374-8265.11330)
Supplement: Supplementary file 1 — Ectopic Pregnancy and Emergencies in the First 20 Weeks.pptxPregnancy Emergencies After 20 Weeks.pptxDelivery Emergencies.pptxPelvic Pain in the Nonpregnant Patient.pptxVaginitis, Cervicitis, and PID.pptxAbnormal Uterine Bleeding.pptxLabor and Perimortem C-Section.pptxSession Review Questions.docxPrecurriculum Survey.docxPostcurriculum Survey.docx [file mep_2374-8265.11330-s001.zip › C. Delivery Emergencies.pptx]

## Slide 1
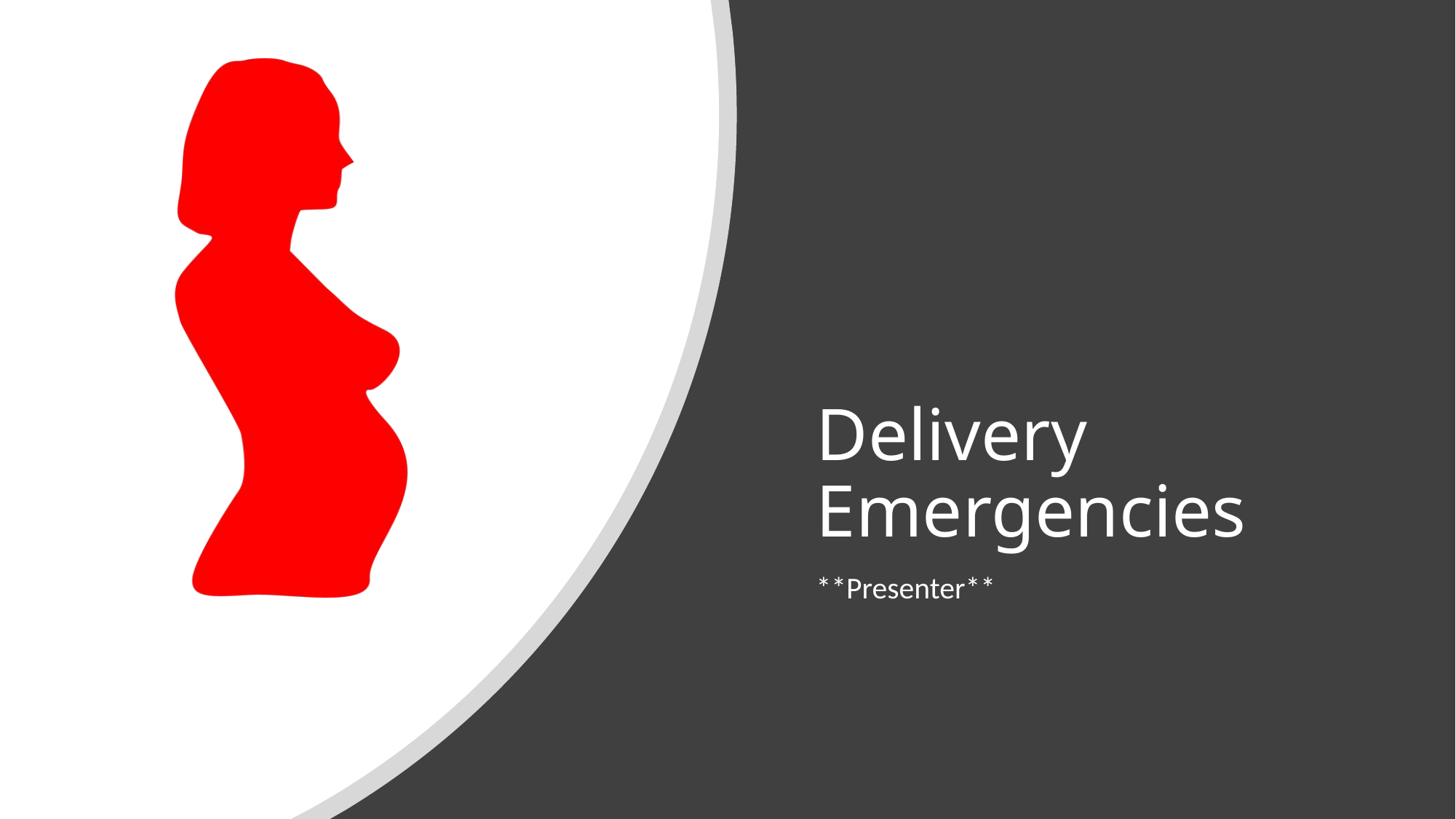

# Delivery Emergencies
**Presenter**

## Slide 2
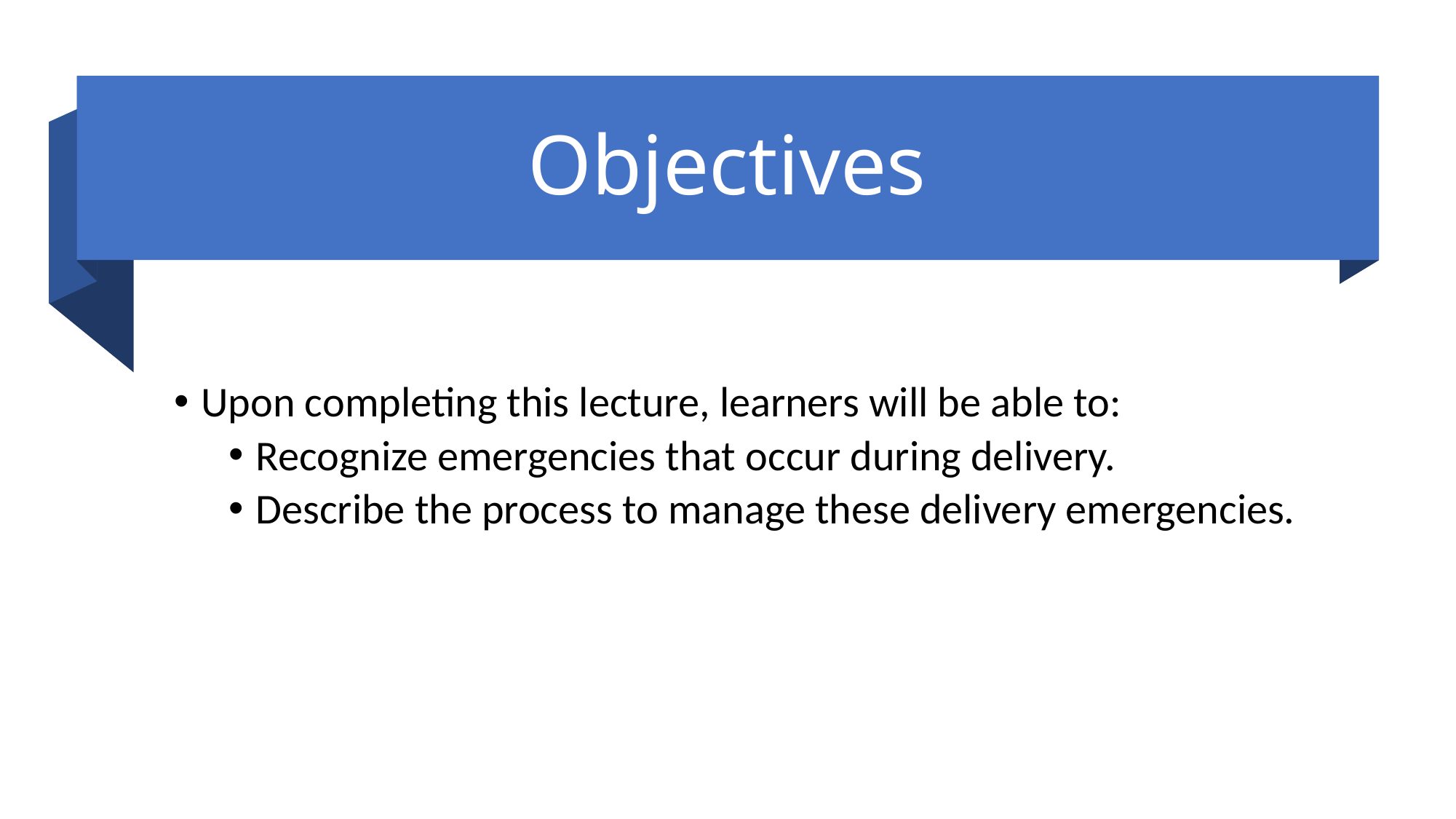

# Objectives
Upon completing this lecture, learners will be able to:
Recognize emergencies that occur during delivery.
Describe the process to manage these delivery emergencies.

## Slide 3
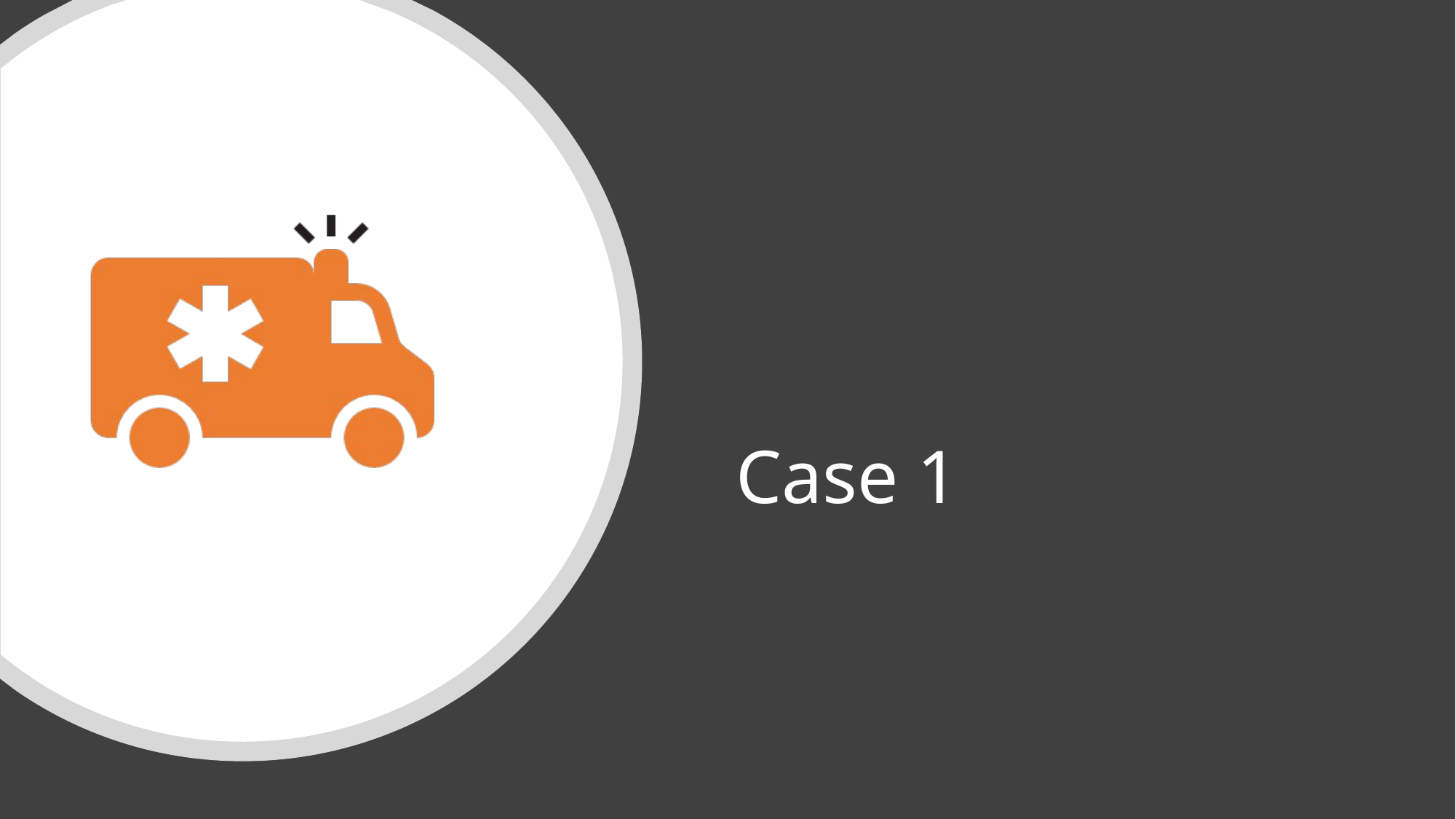

# Case 1

## Slide 4
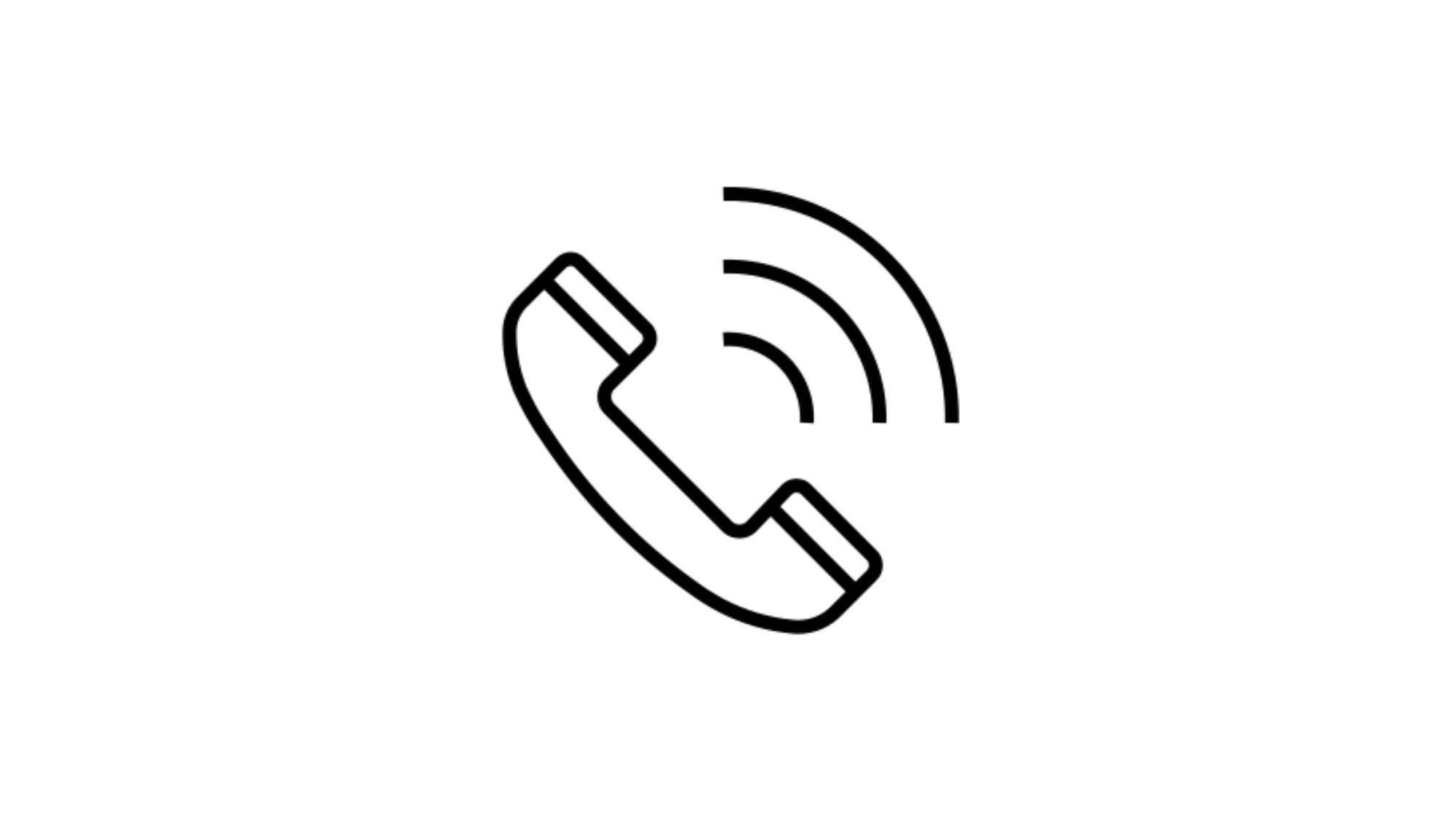

## Slide 5
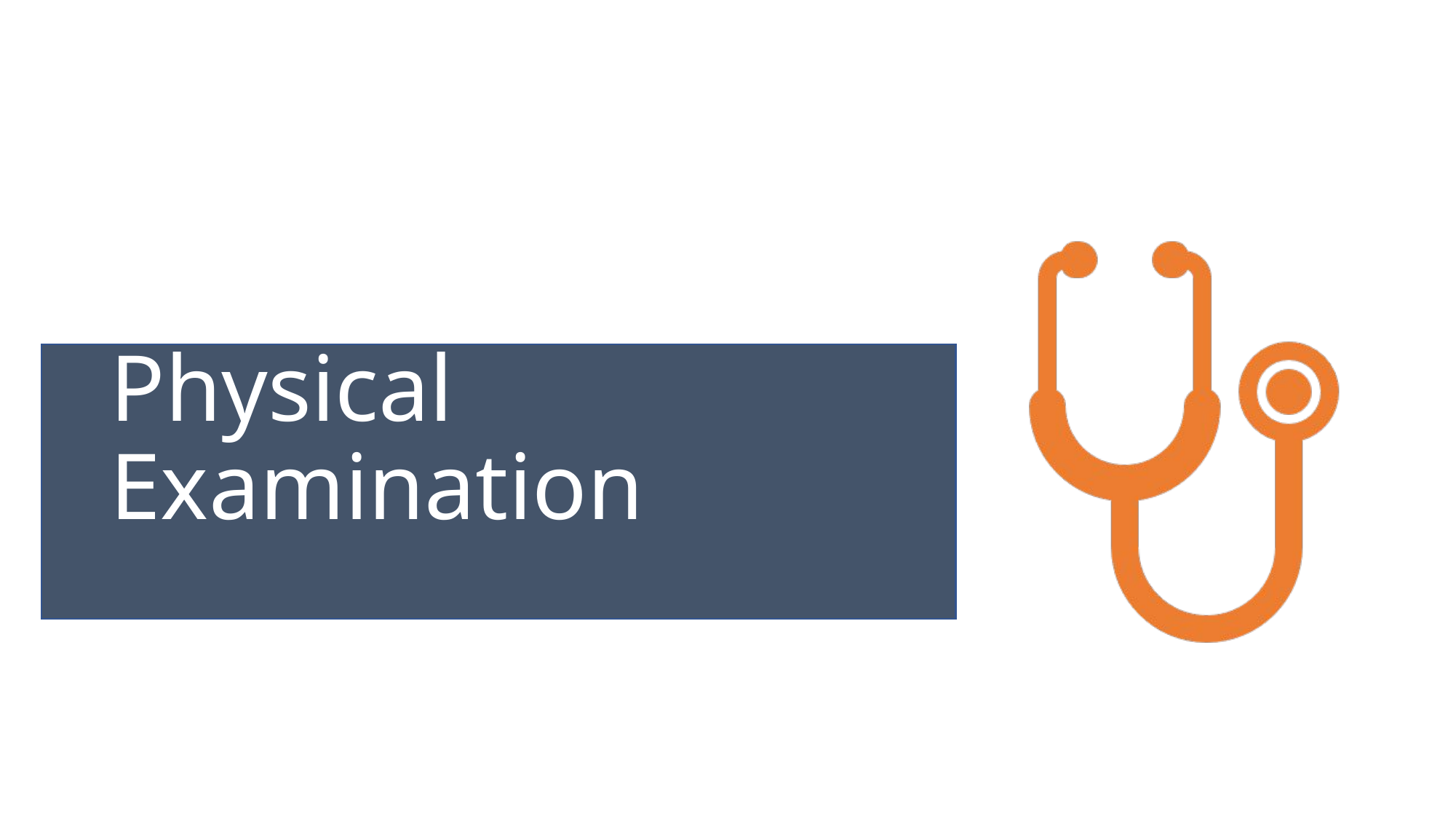

# Physical Examination

## Slide 6
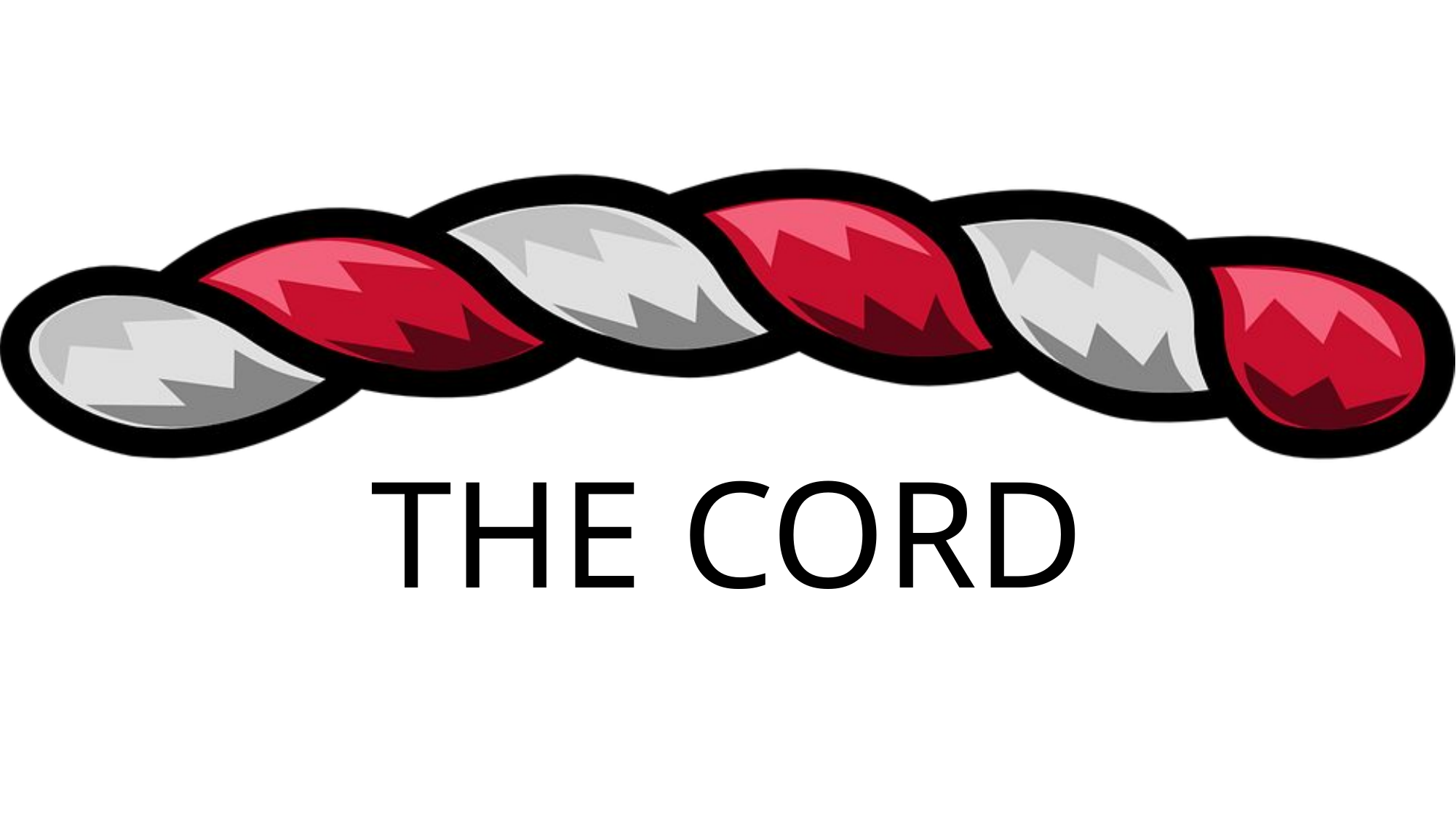

THE CORD

## Slide 7
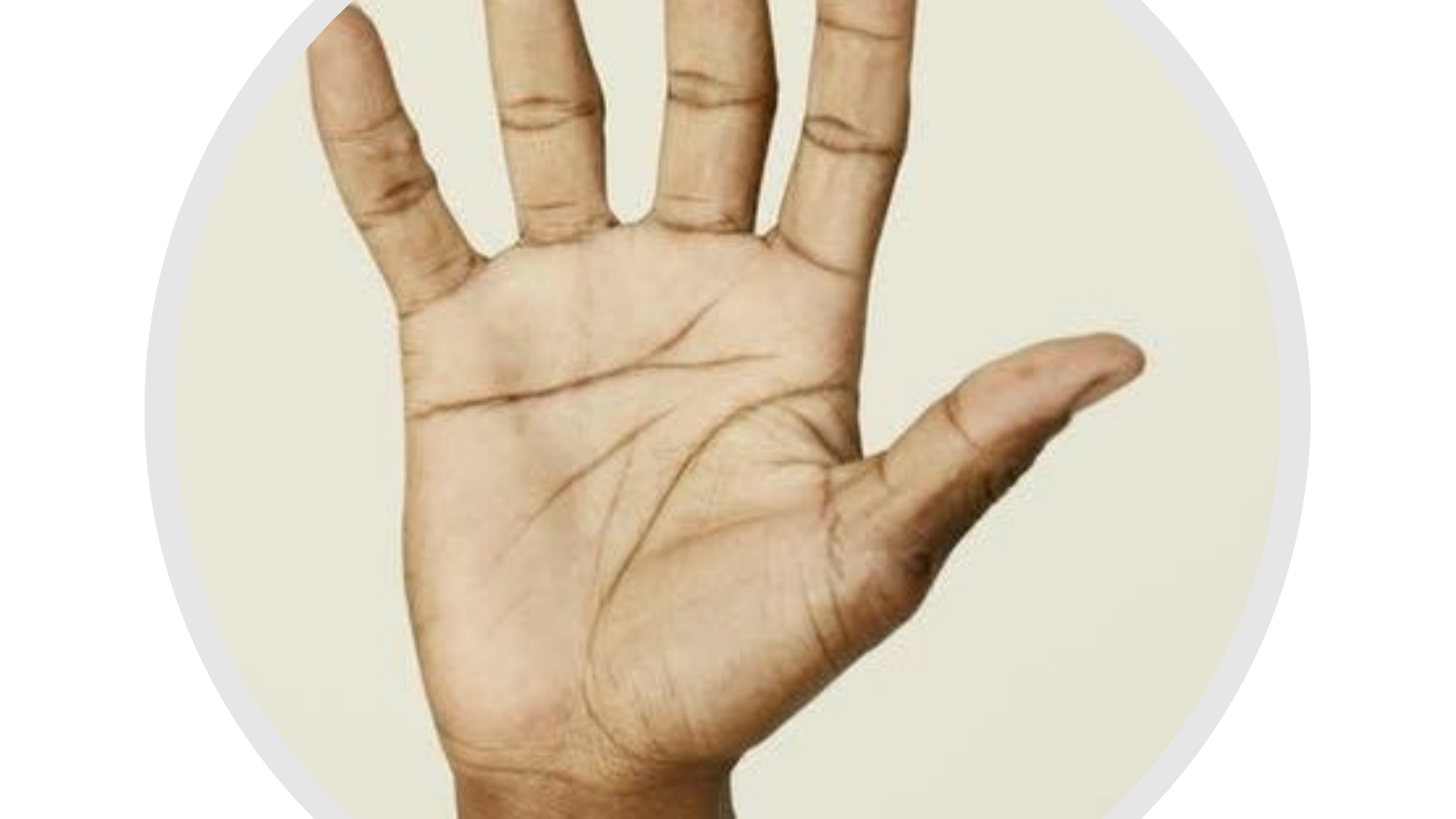

## Slide 8
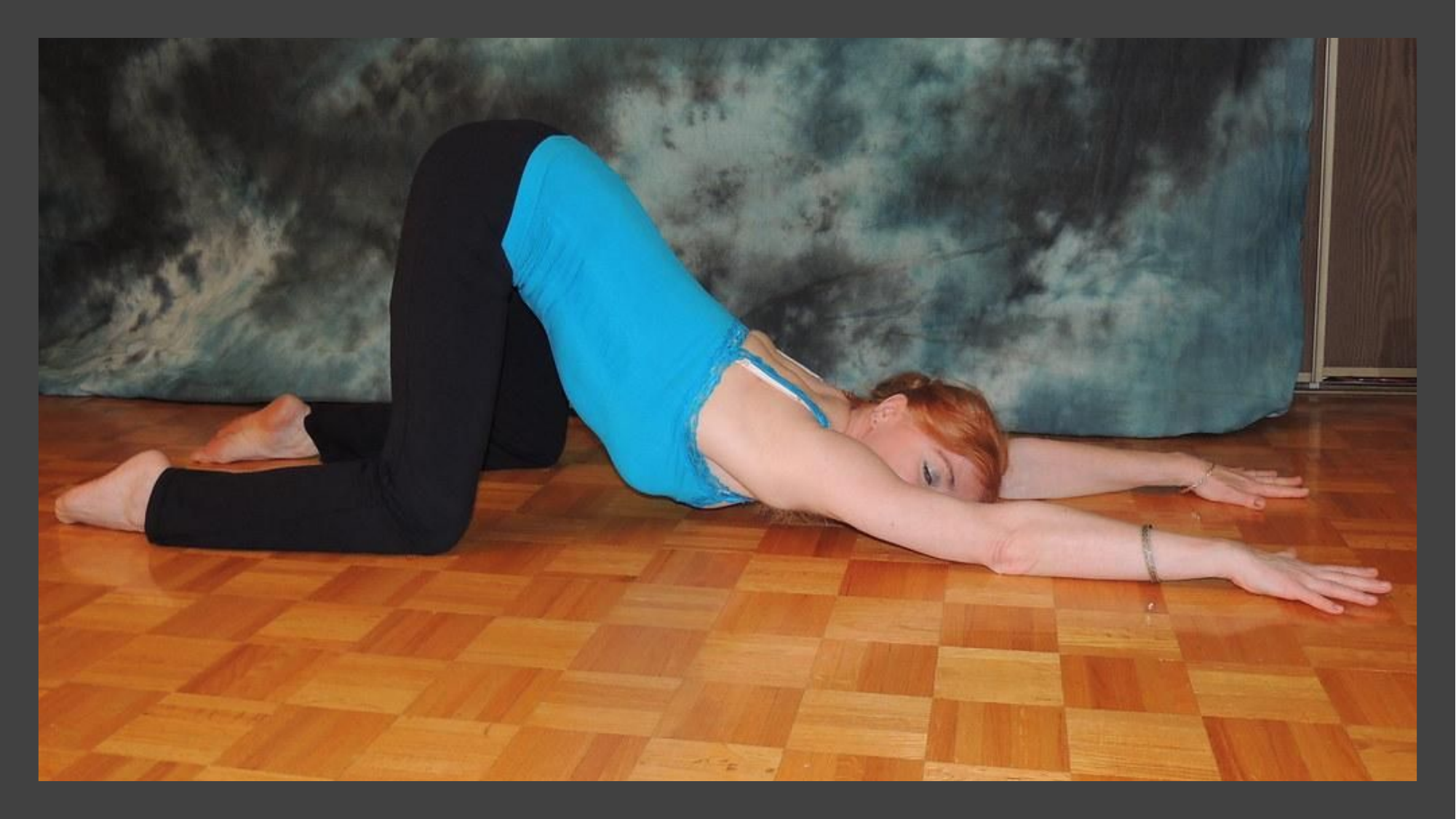

## Slide 9
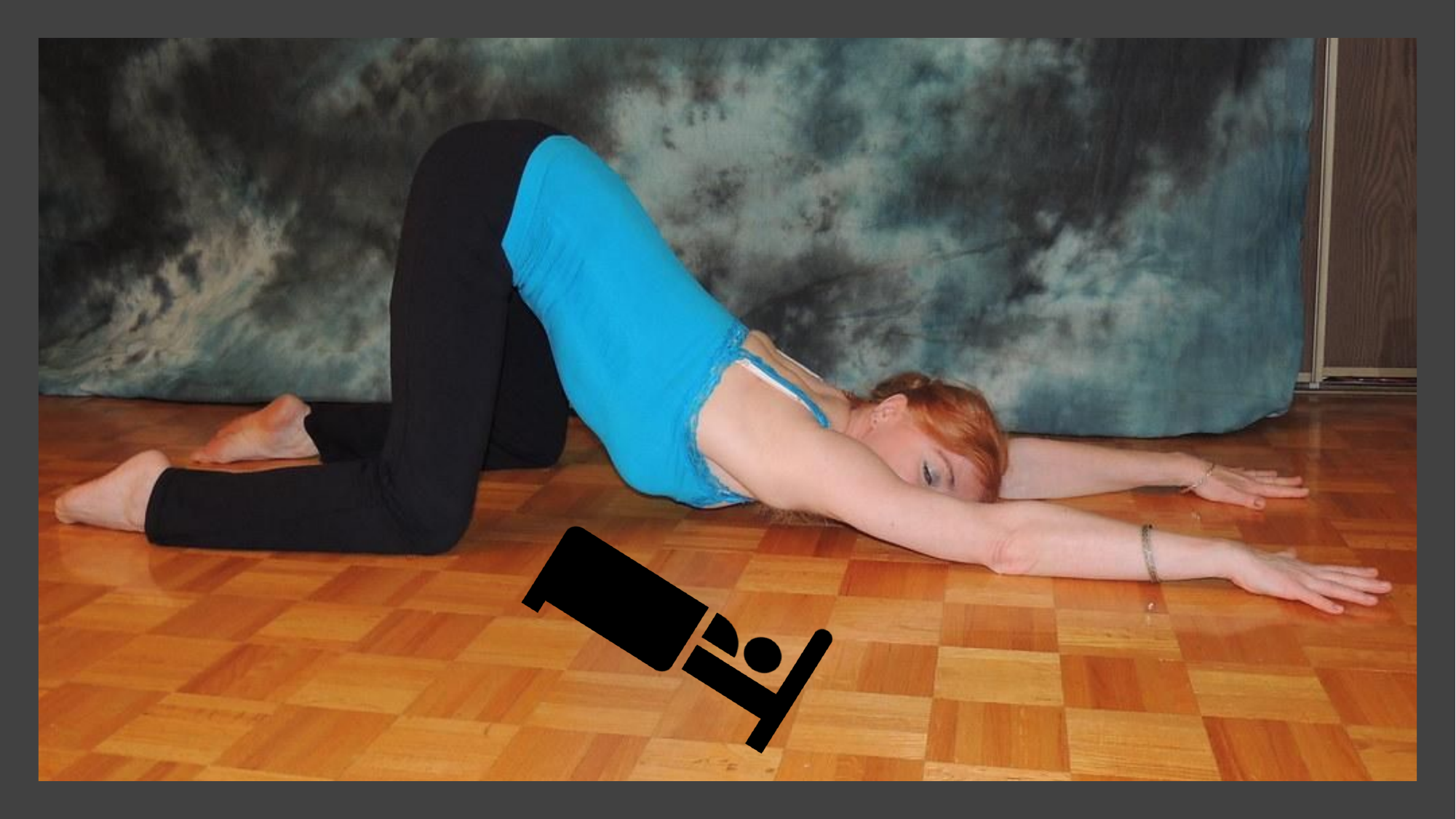

## Slide 10
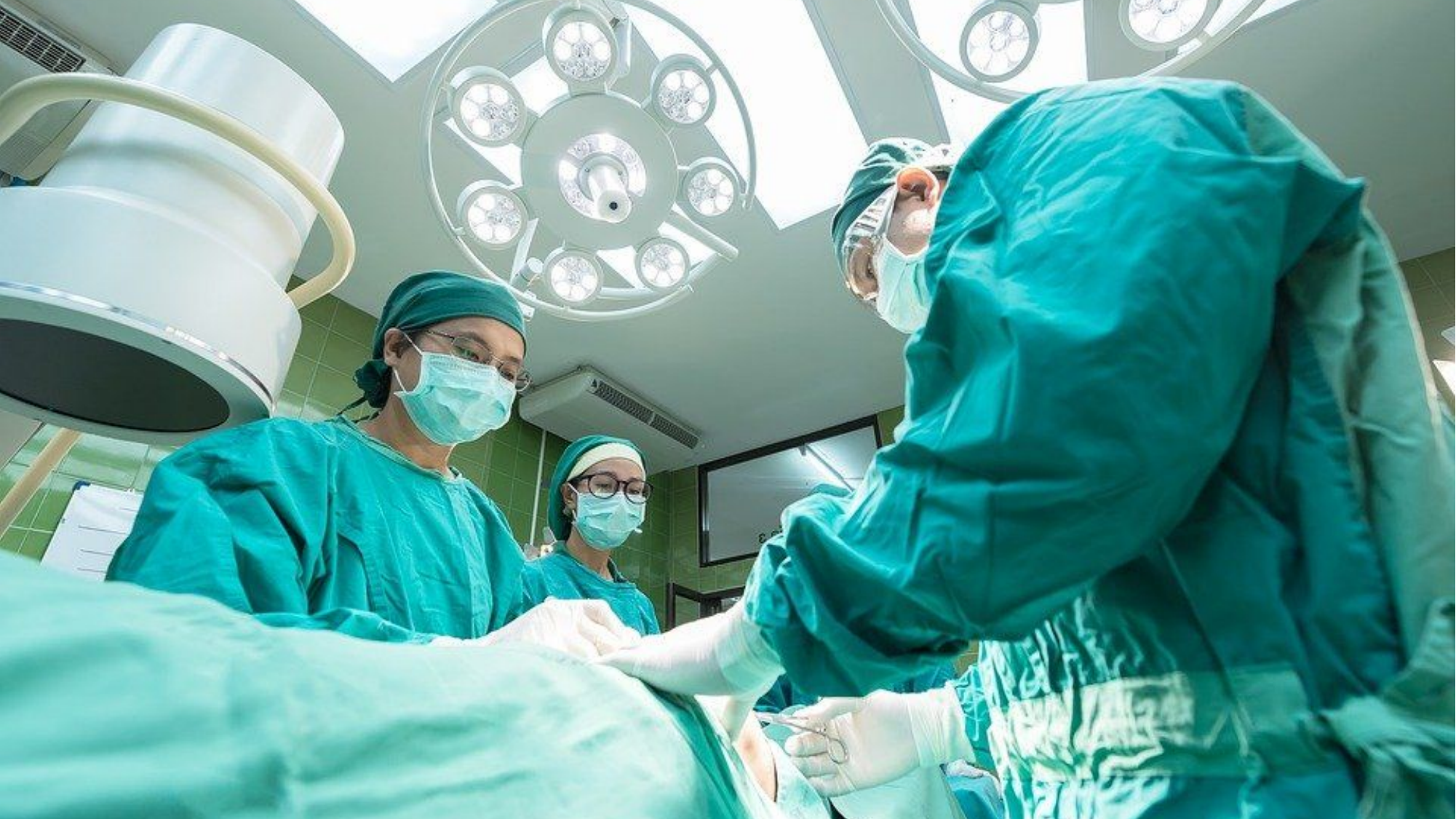

#
| |
| --- |

## Slide 11
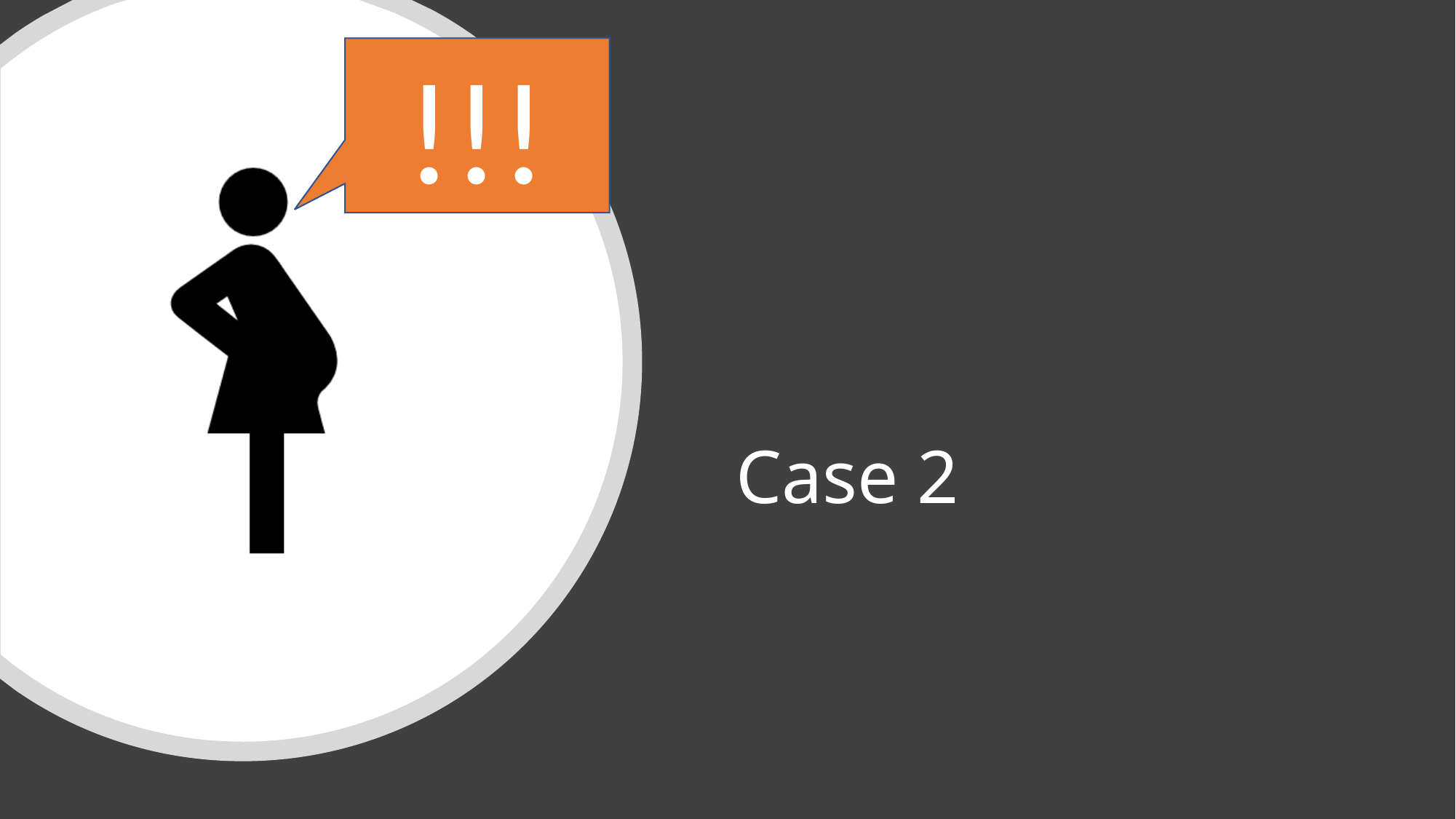

!!!
# Case 2

## Slide 12
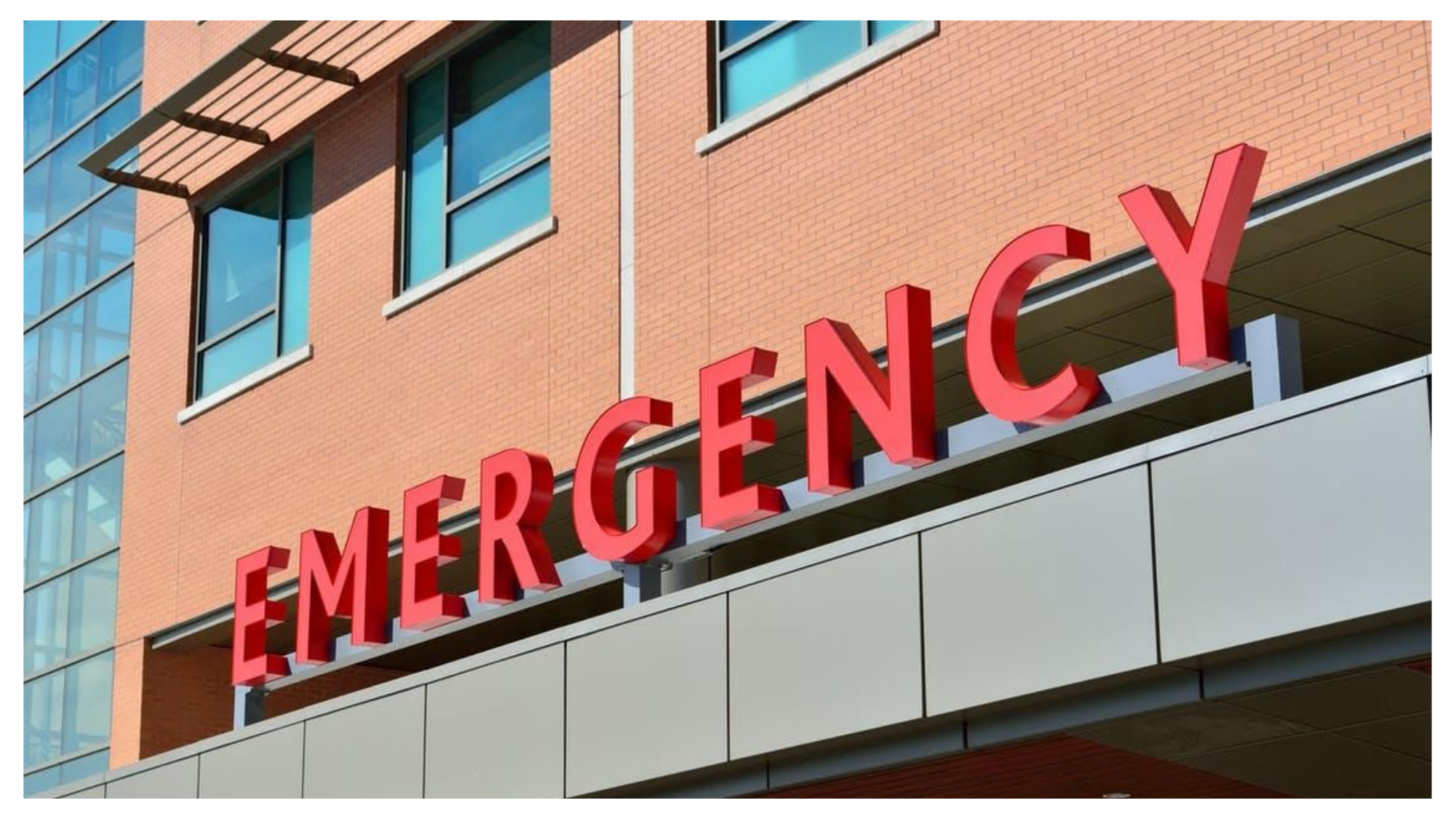

## Slide 13
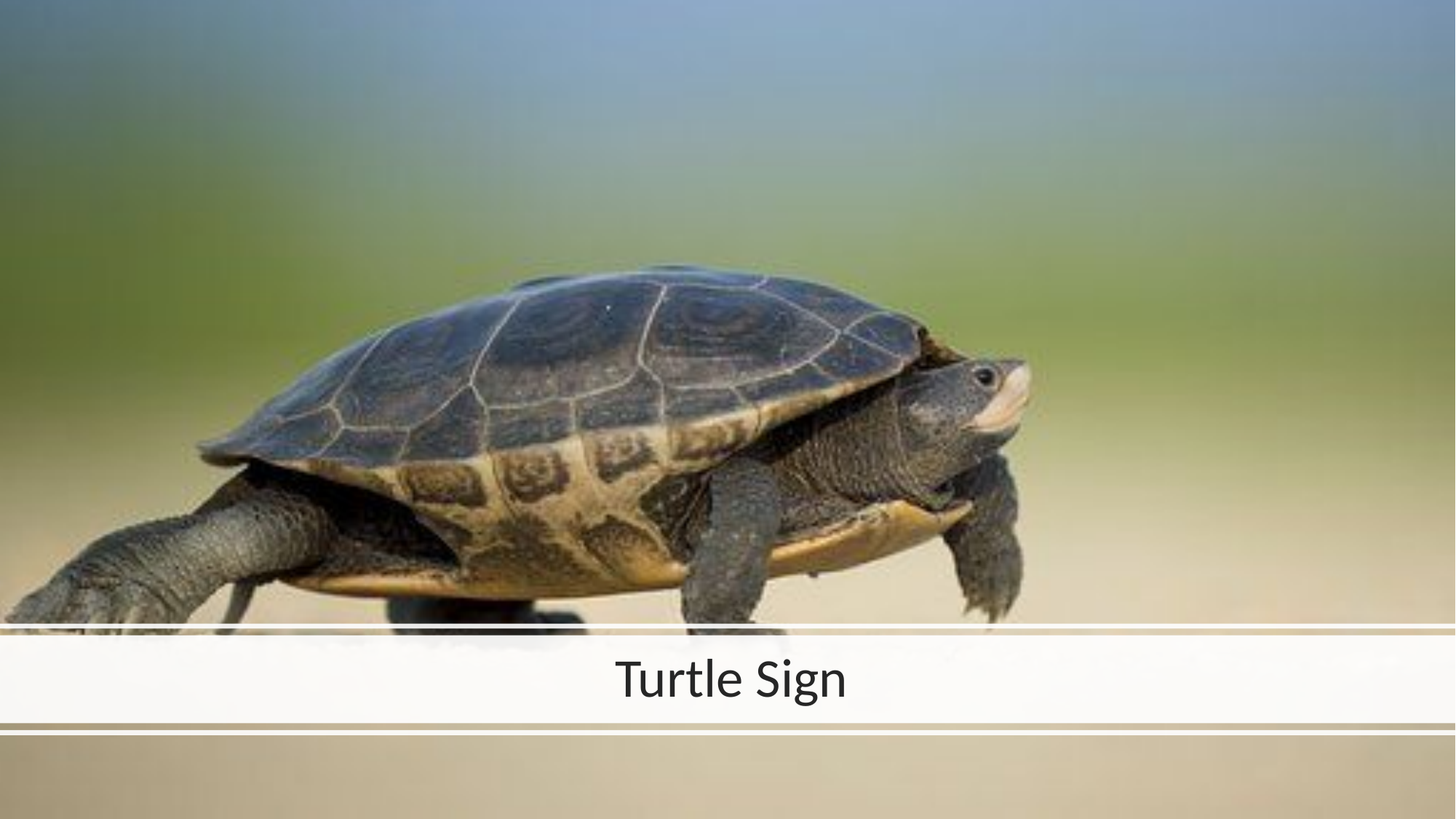

# Turtle Sign

## Slide 14
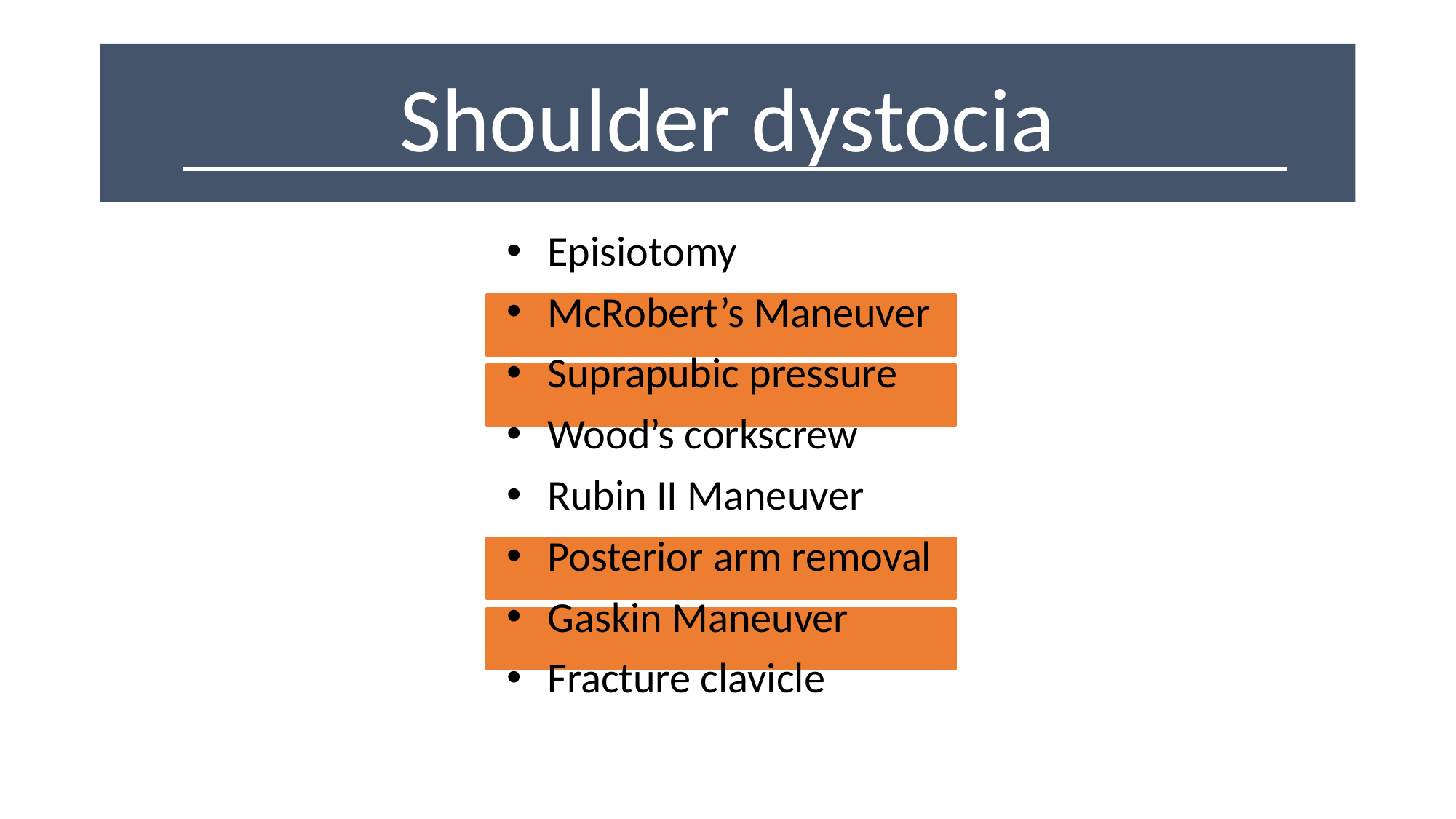

# Shoulder dystocia
Episiotomy
McRobert’s Maneuver
Suprapubic pressure
Wood’s corkscrew
Rubin II Maneuver
Posterior arm removal
Gaskin Maneuver
Fracture clavicle

## Slide 15
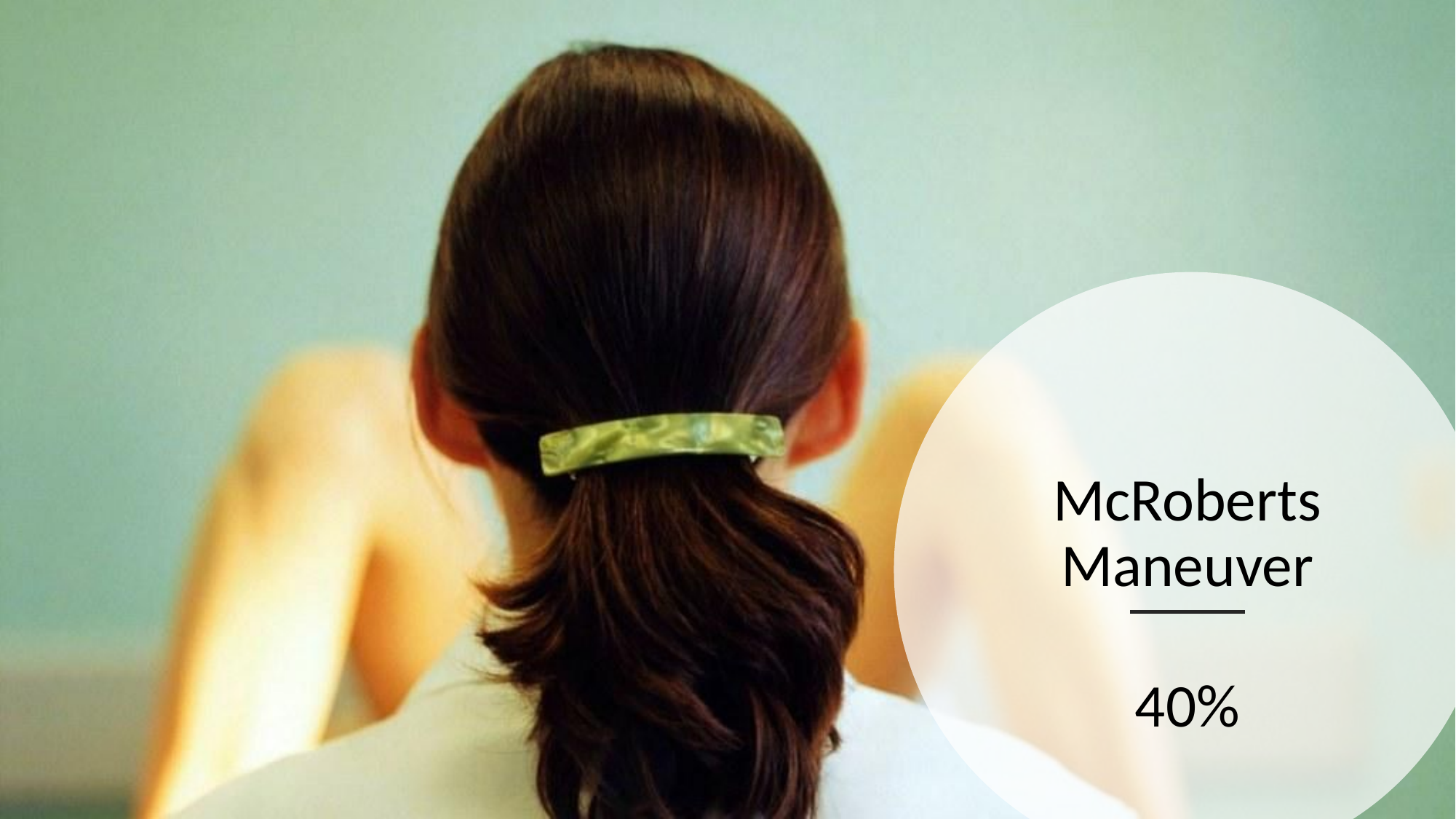

# McRoberts Maneuver
40%

## Slide 16
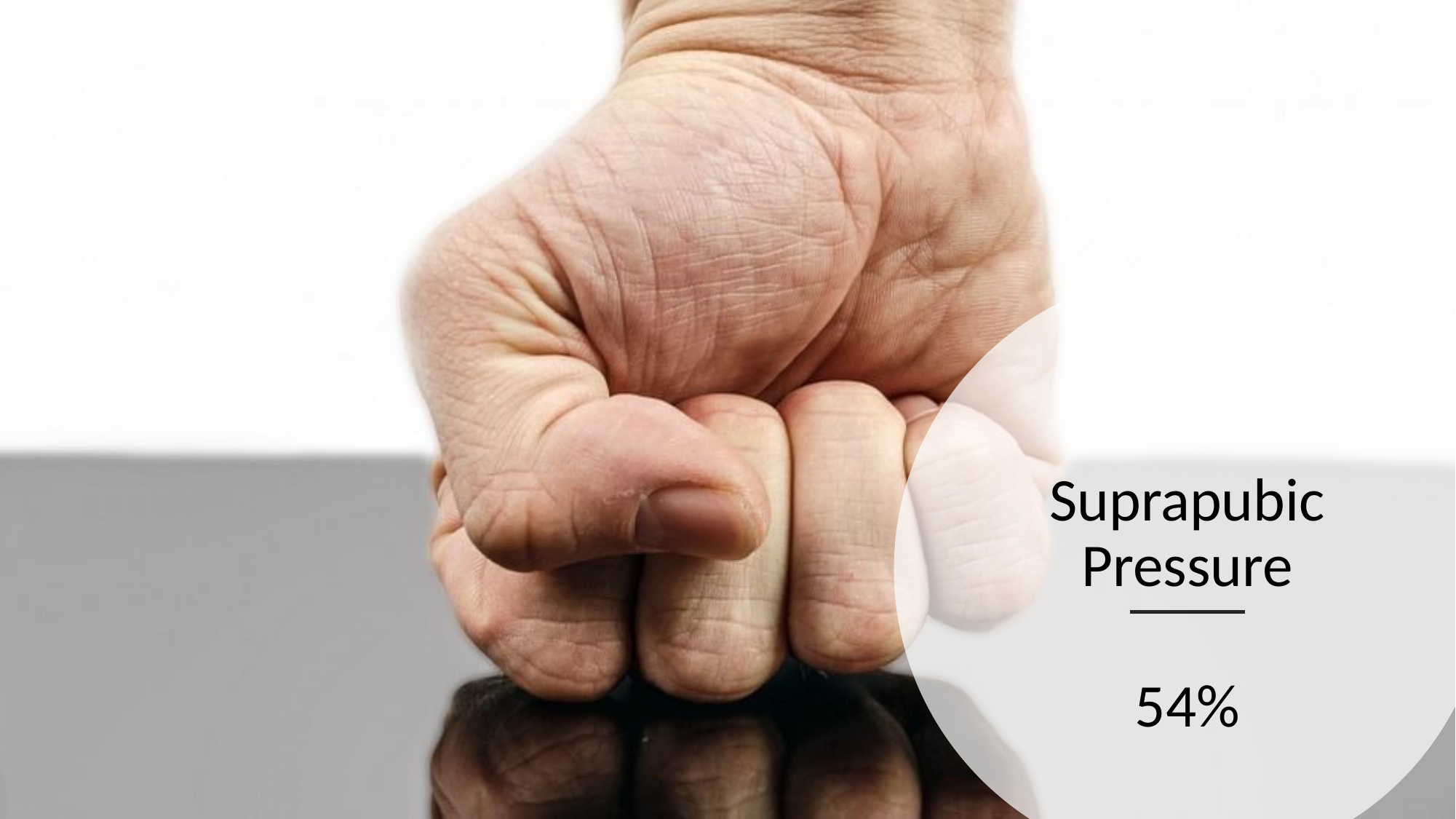

# Suprapubic Pressure
54%

## Slide 17
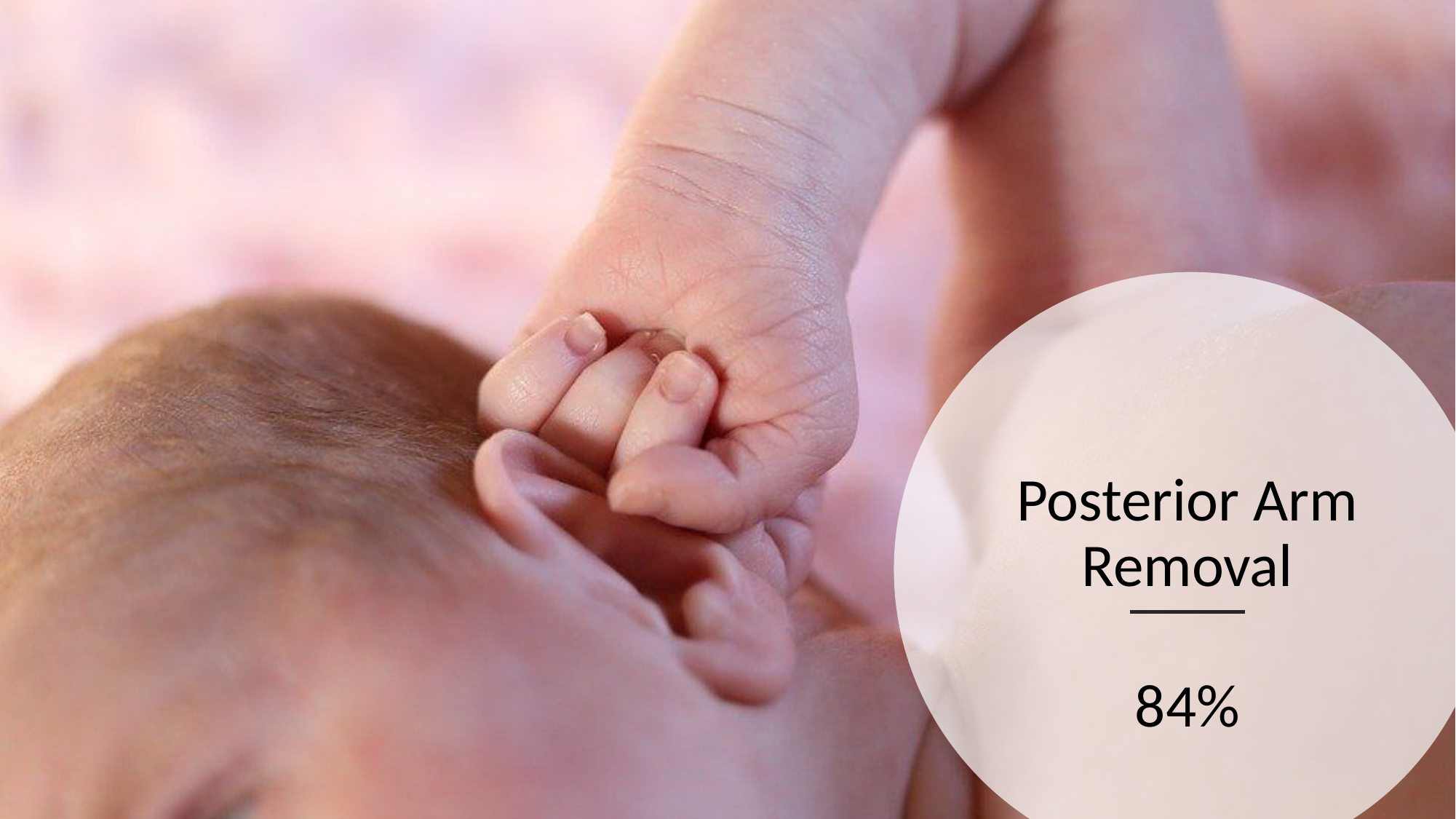

# Posterior Arm Removal
84%

## Slide 18
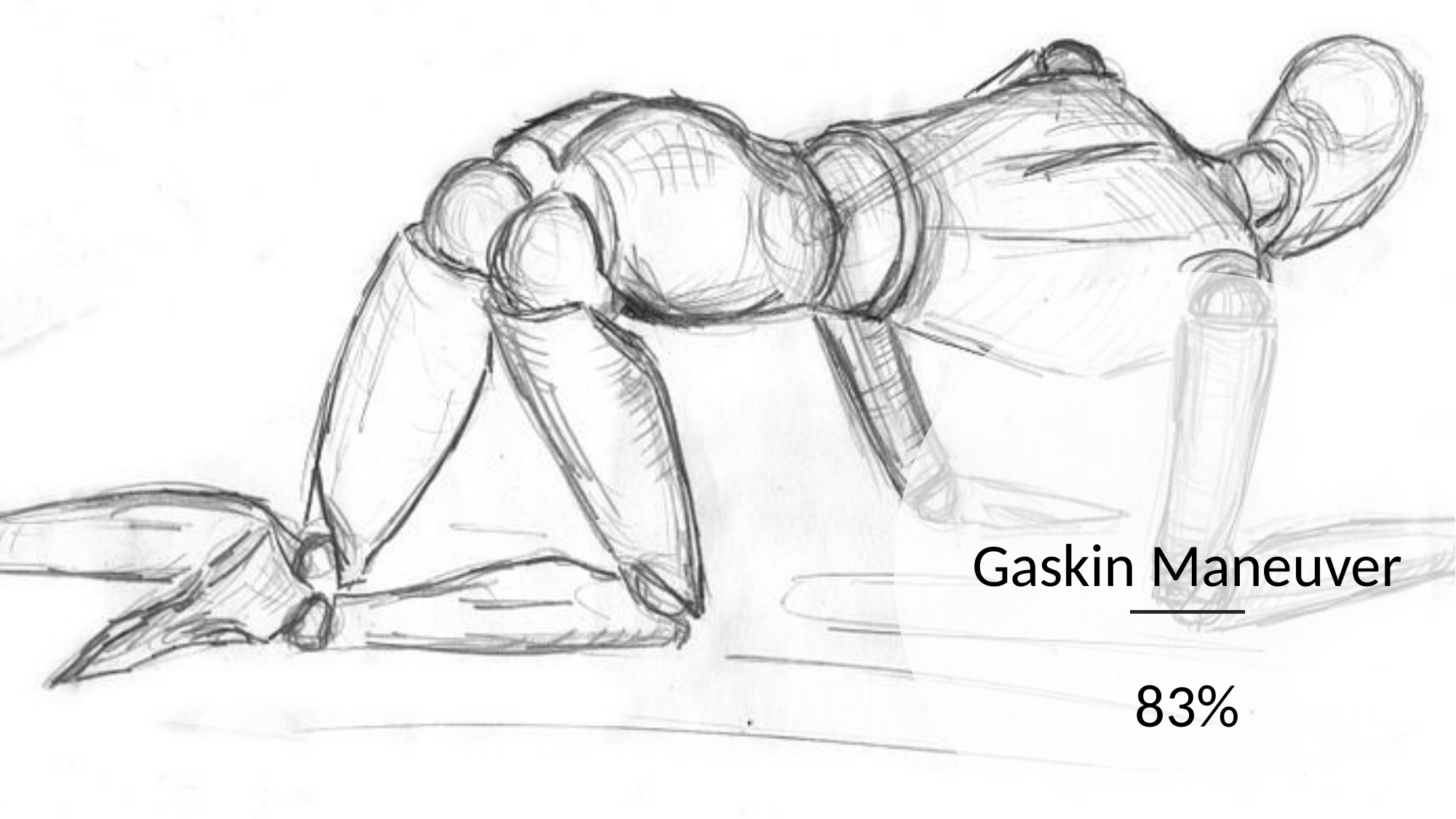

# Gaskin Maneuver
83%

## Slide 19
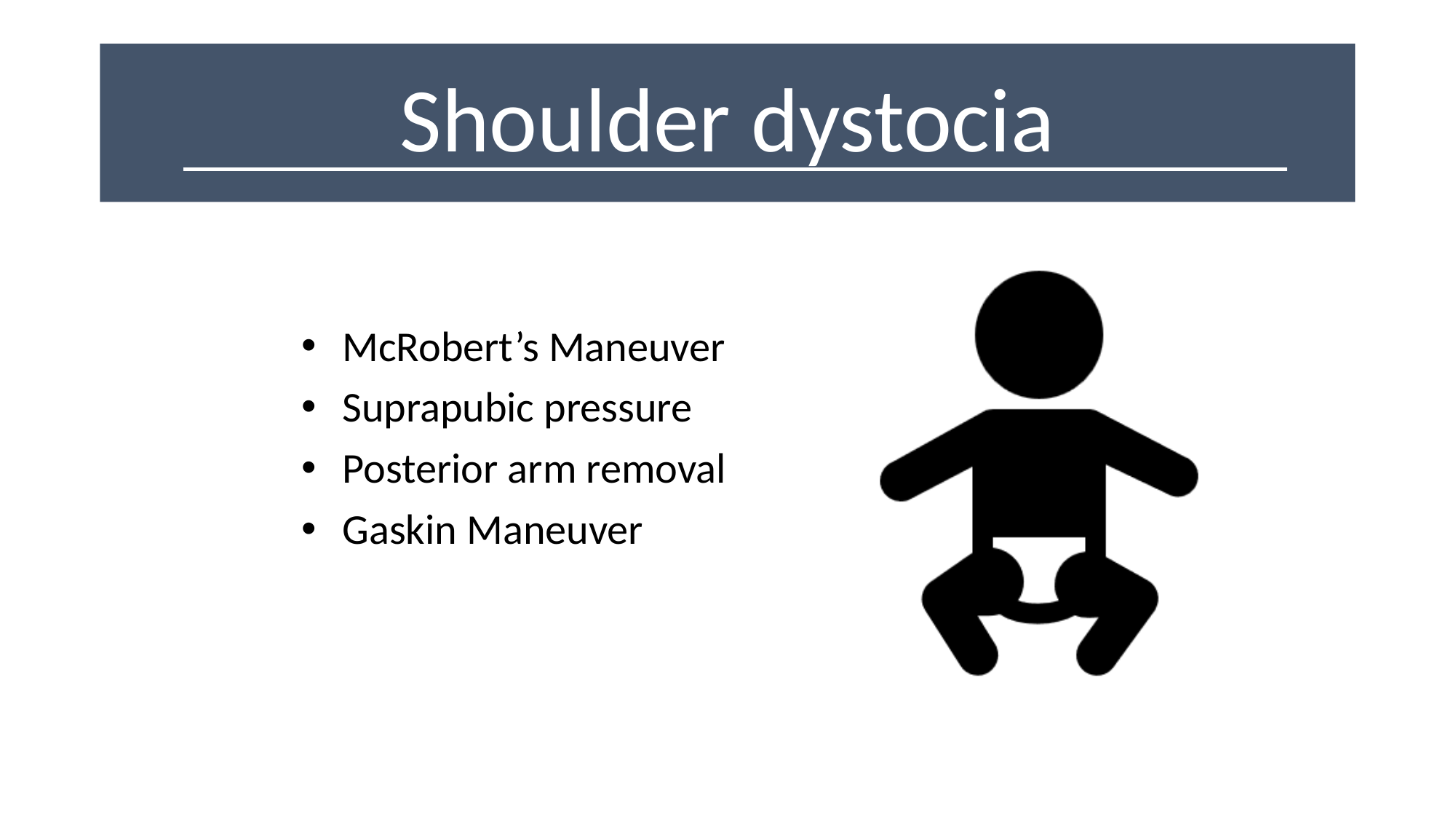

# Shoulder dystocia
McRobert’s Maneuver
Suprapubic pressure
Posterior arm removal
Gaskin Maneuver

## Slide 20
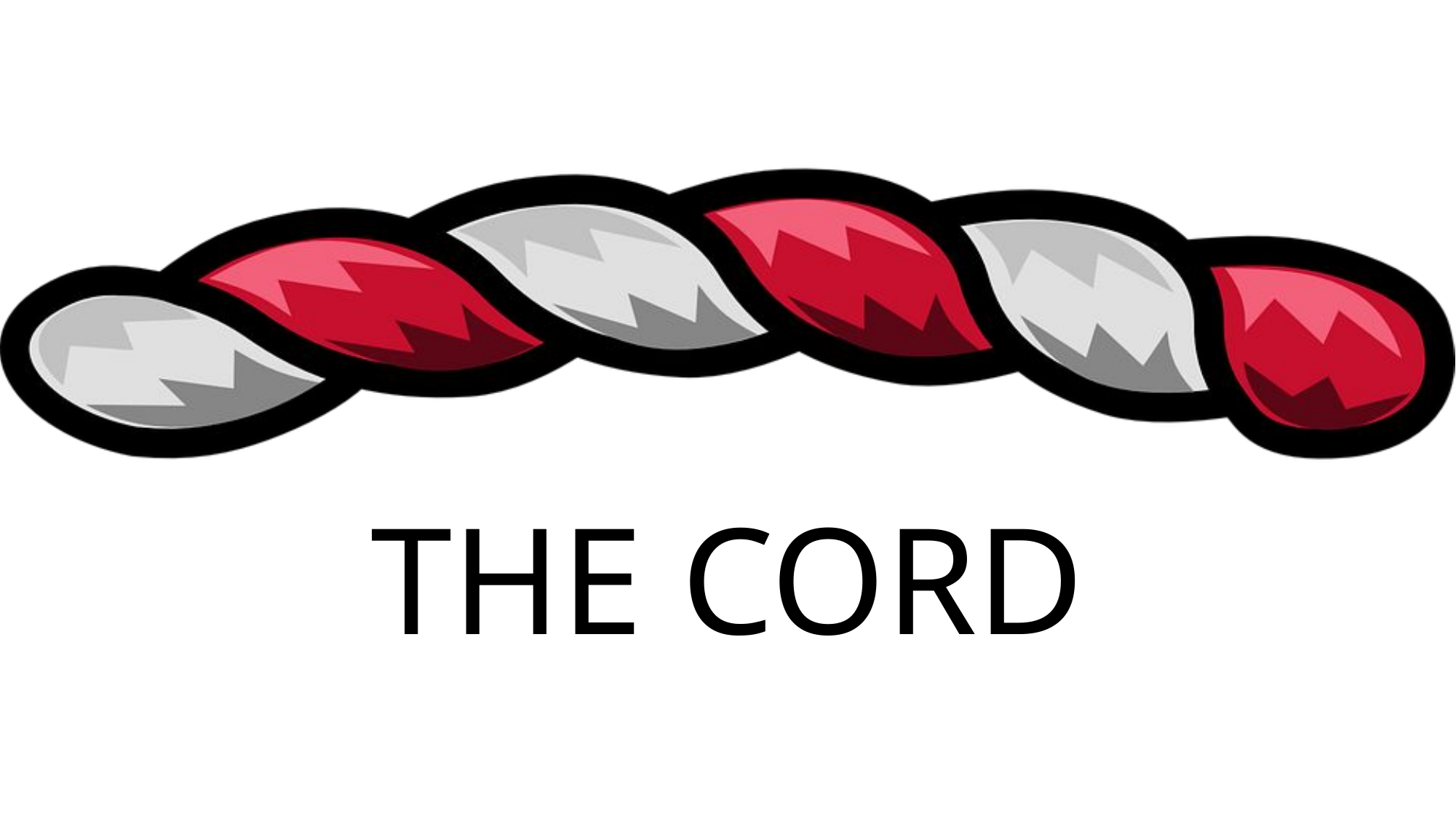

THE CORD

## Slide 21
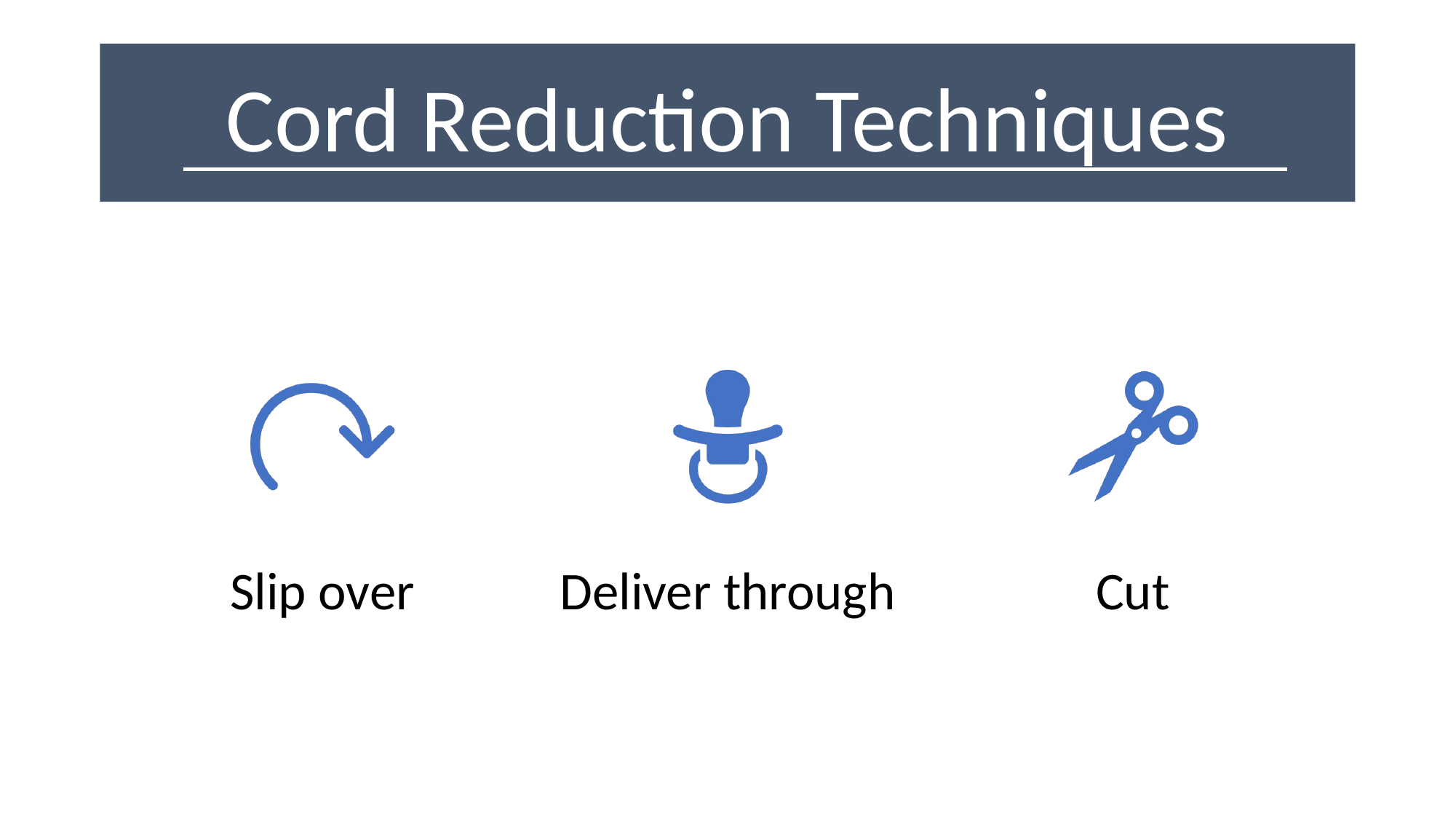

# Cord reduction techniques
Cord Reduction Techniques
Slip over
Deliver through
Cut

## Slide 22
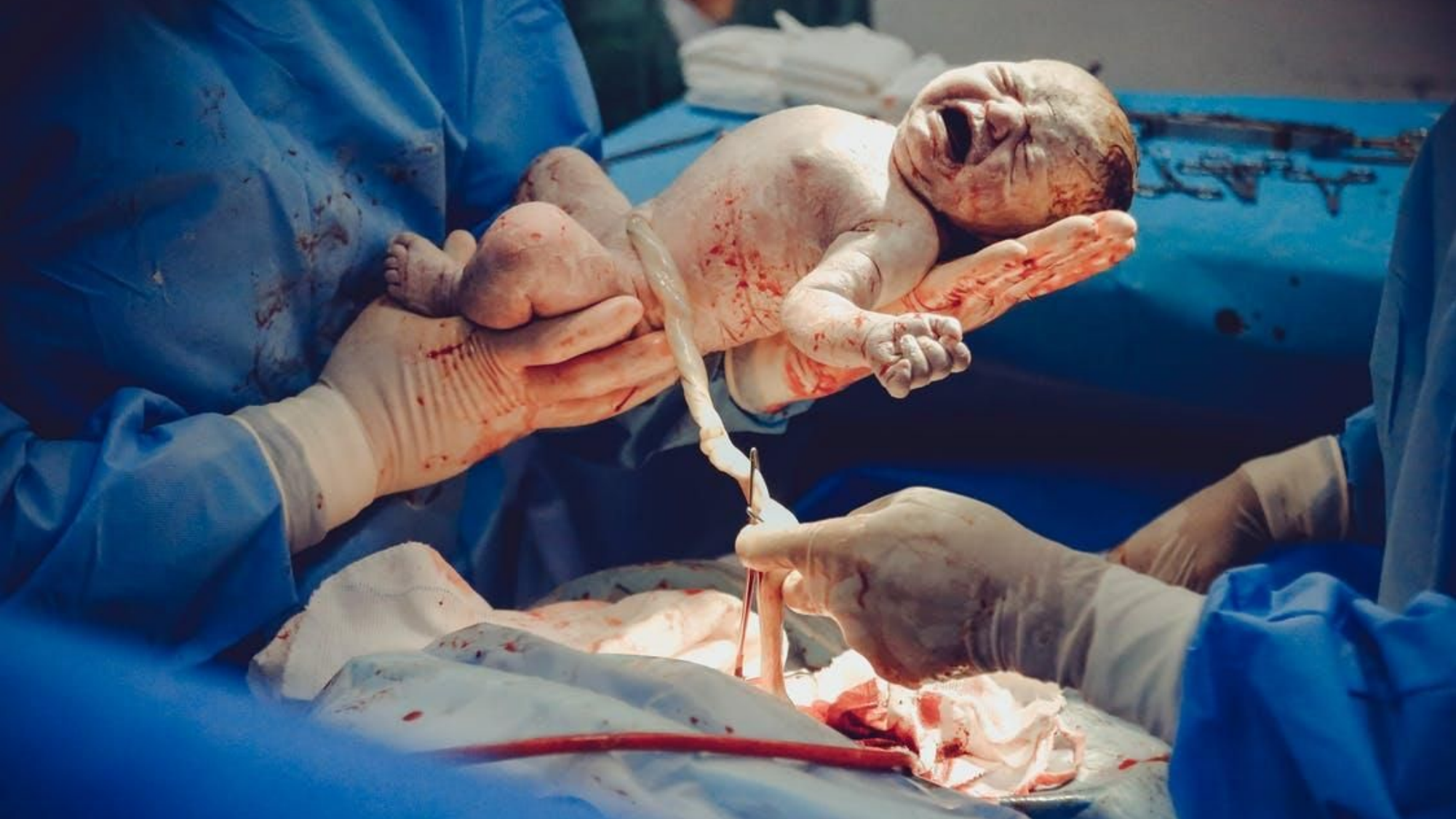

## Slide 23
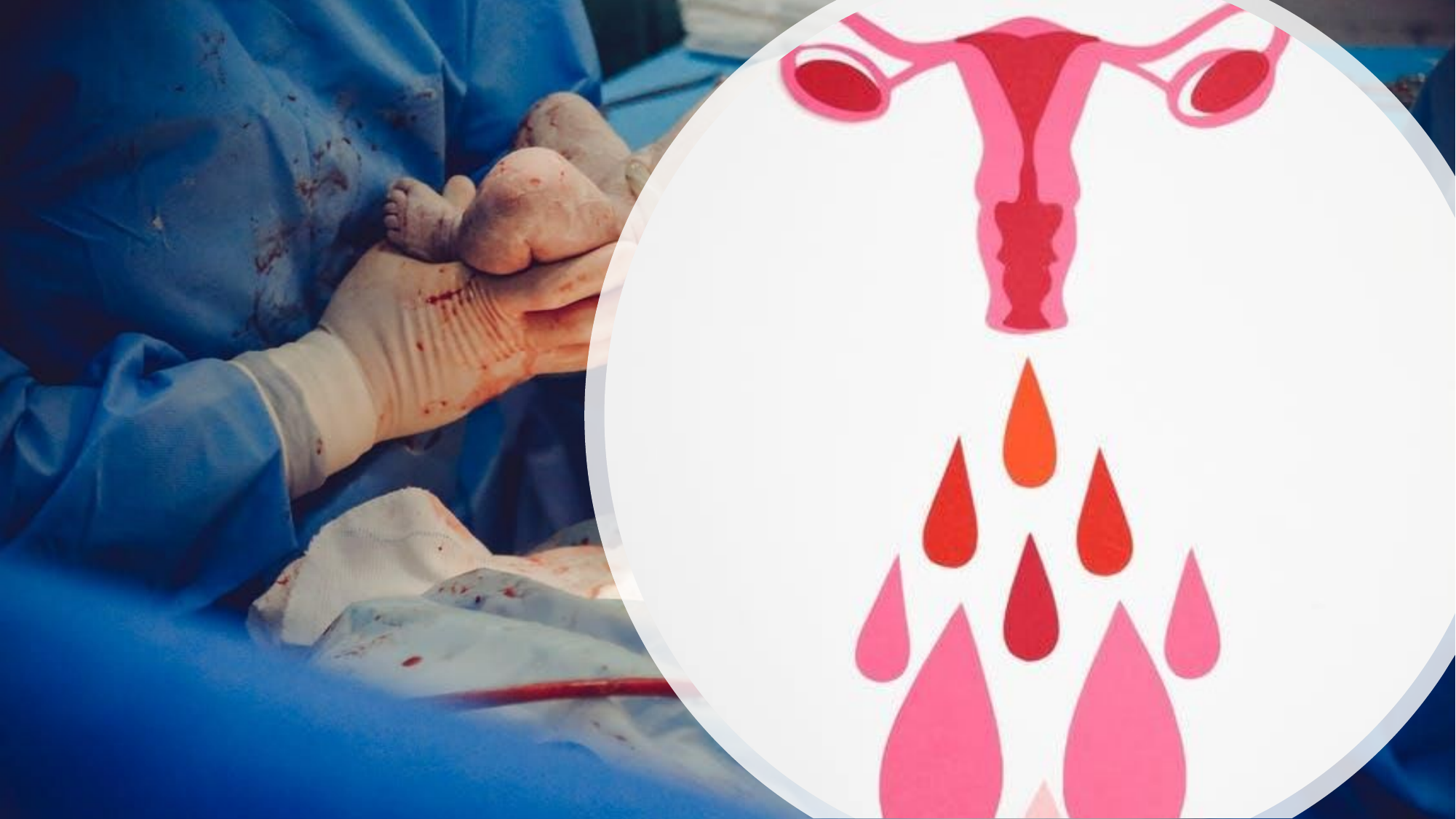

## Slide 24
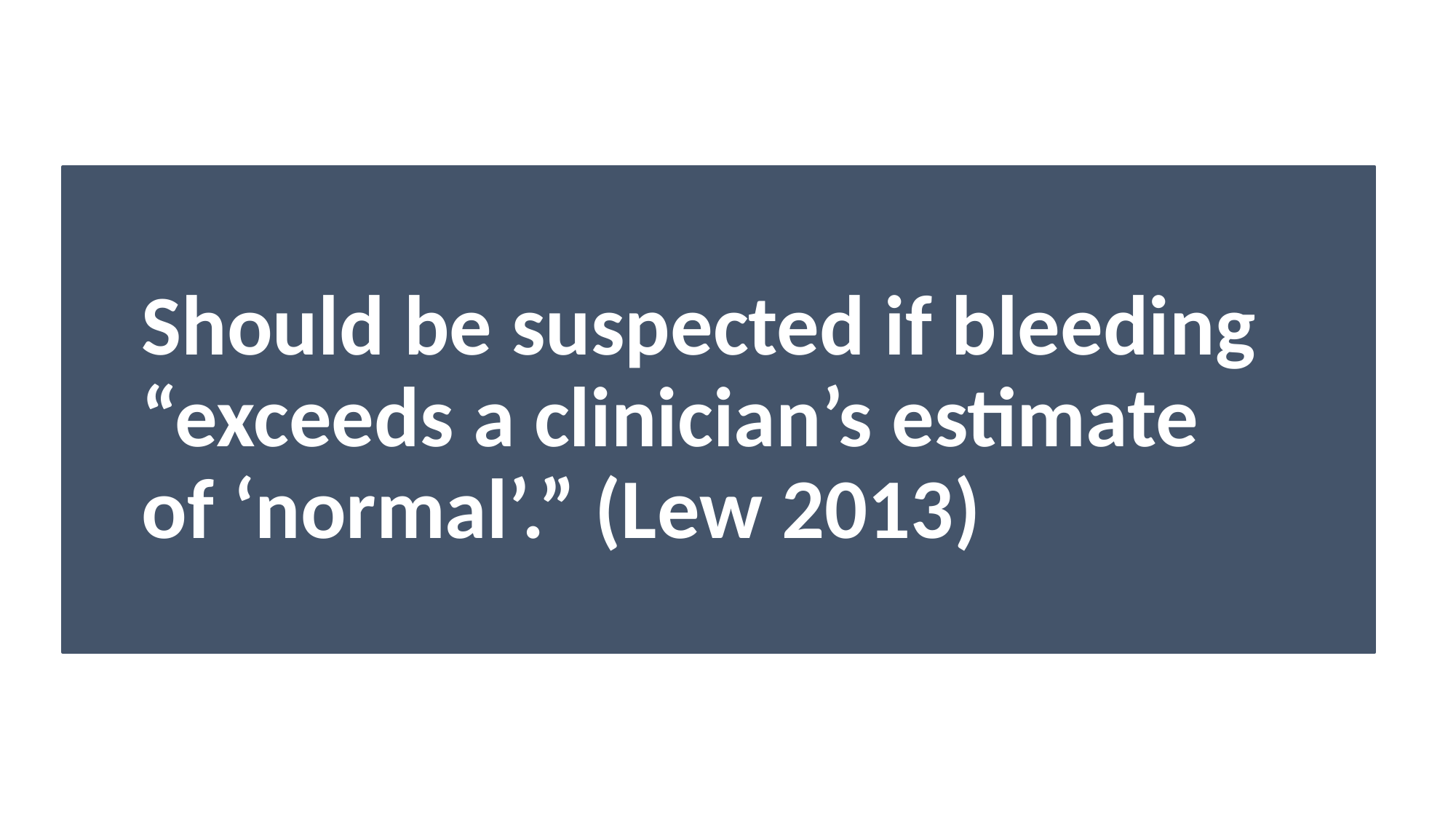

# Should be suspected if bleeding “exceeds a clinician’s estimate of ‘normal’.” (Lew 2013)

## Slide 25
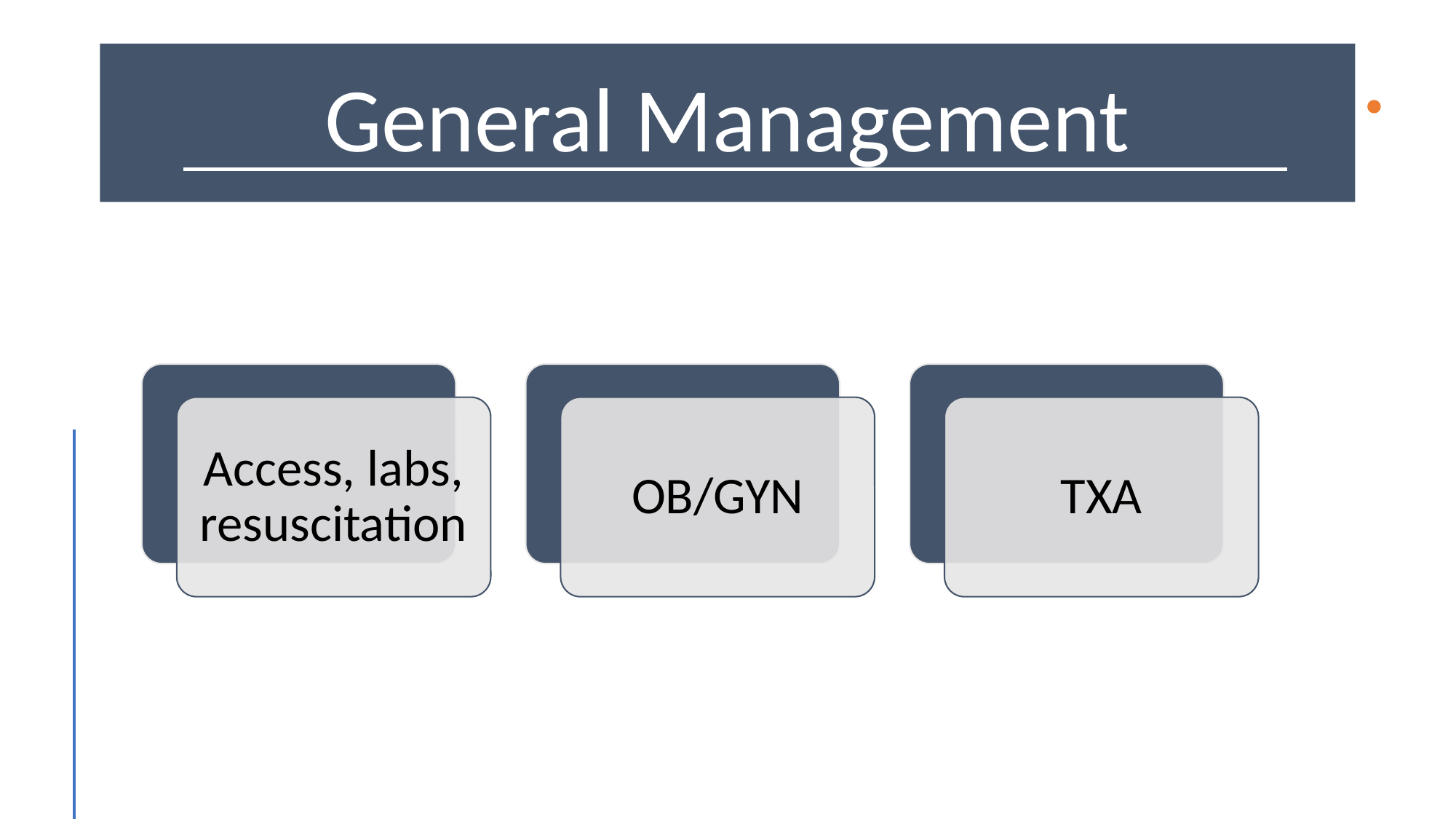

# General Management
Access, labs, resuscitation
OB/GYN
TXA

## Slide 26
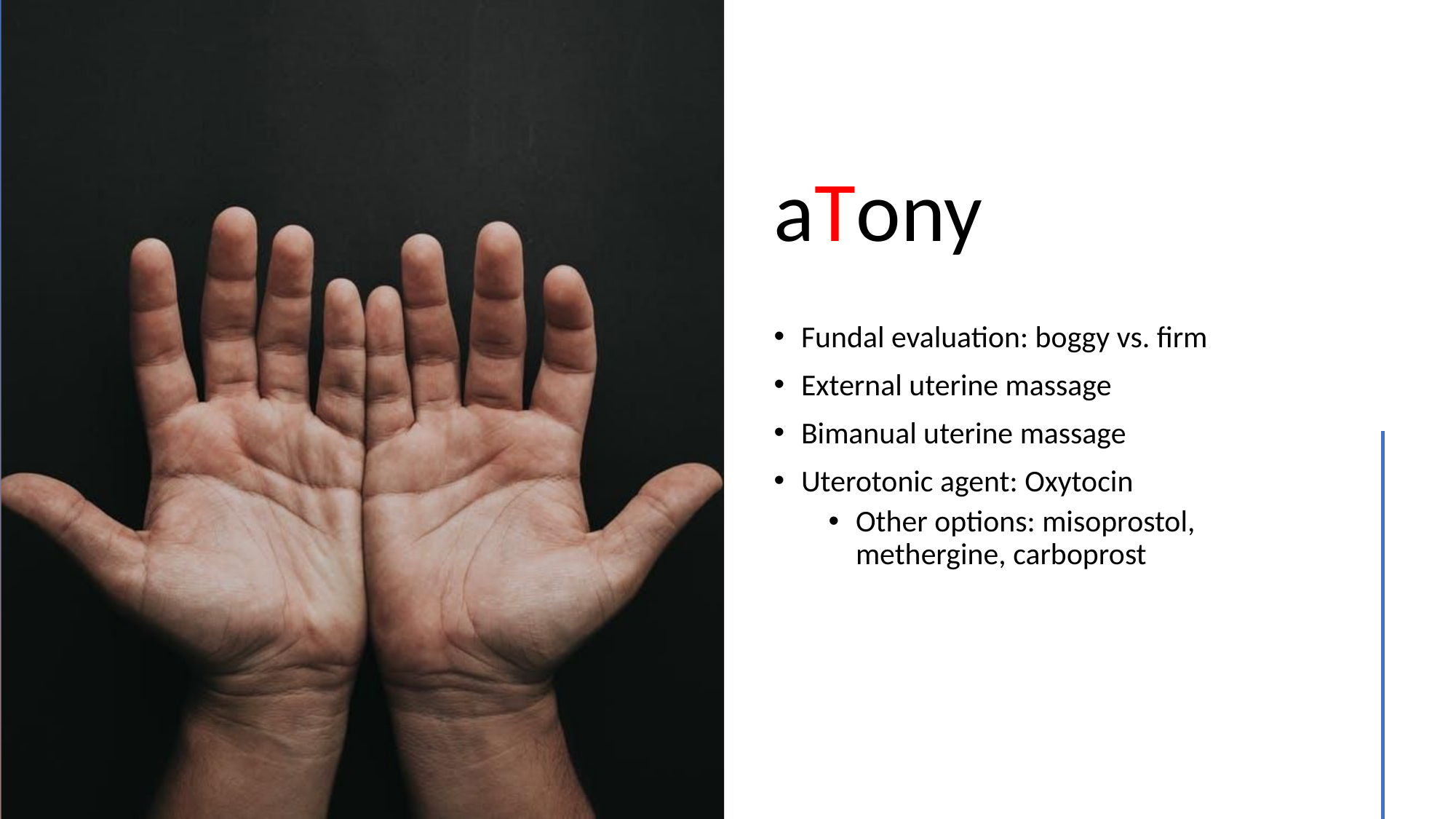

# aTony
Fundal evaluation: boggy vs. firm
External uterine massage
Bimanual uterine massage
Uterotonic agent: Oxytocin
Other options: misoprostol, methergine, carboprost

## Slide 27
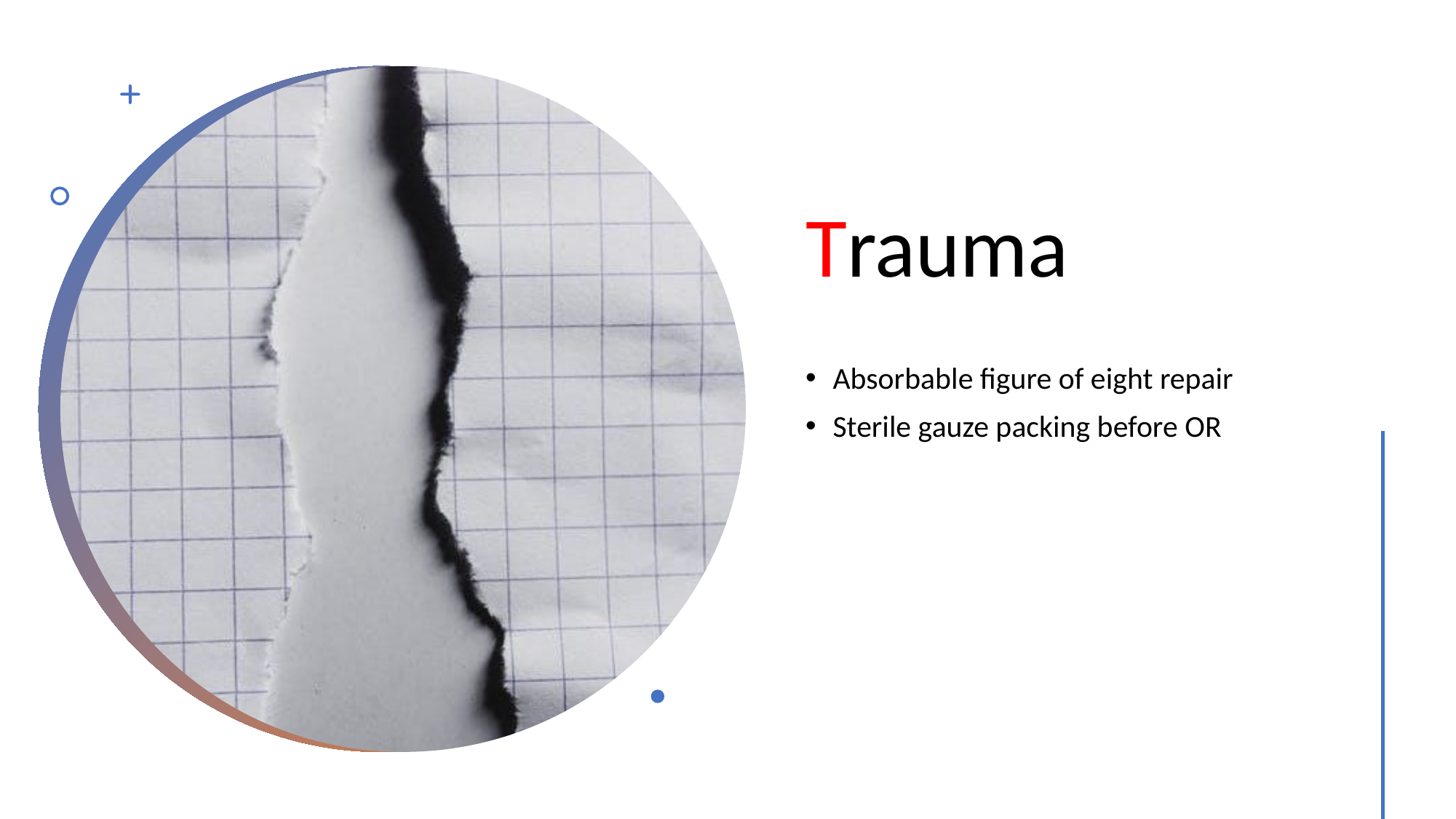

# Trauma
Absorbable figure of eight repair
Sterile gauze packing before OR

## Slide 28
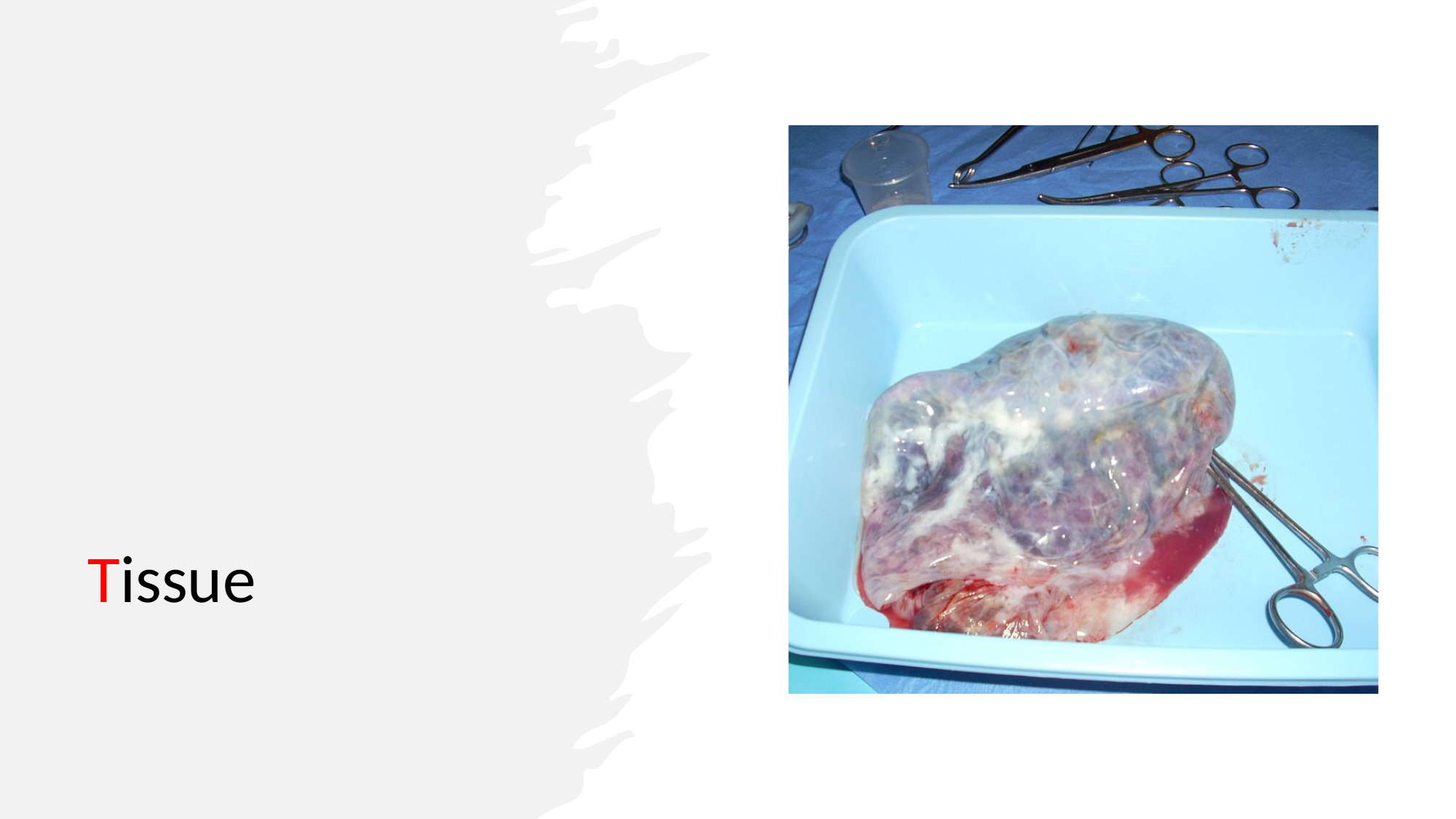

# Tissue

## Slide 29
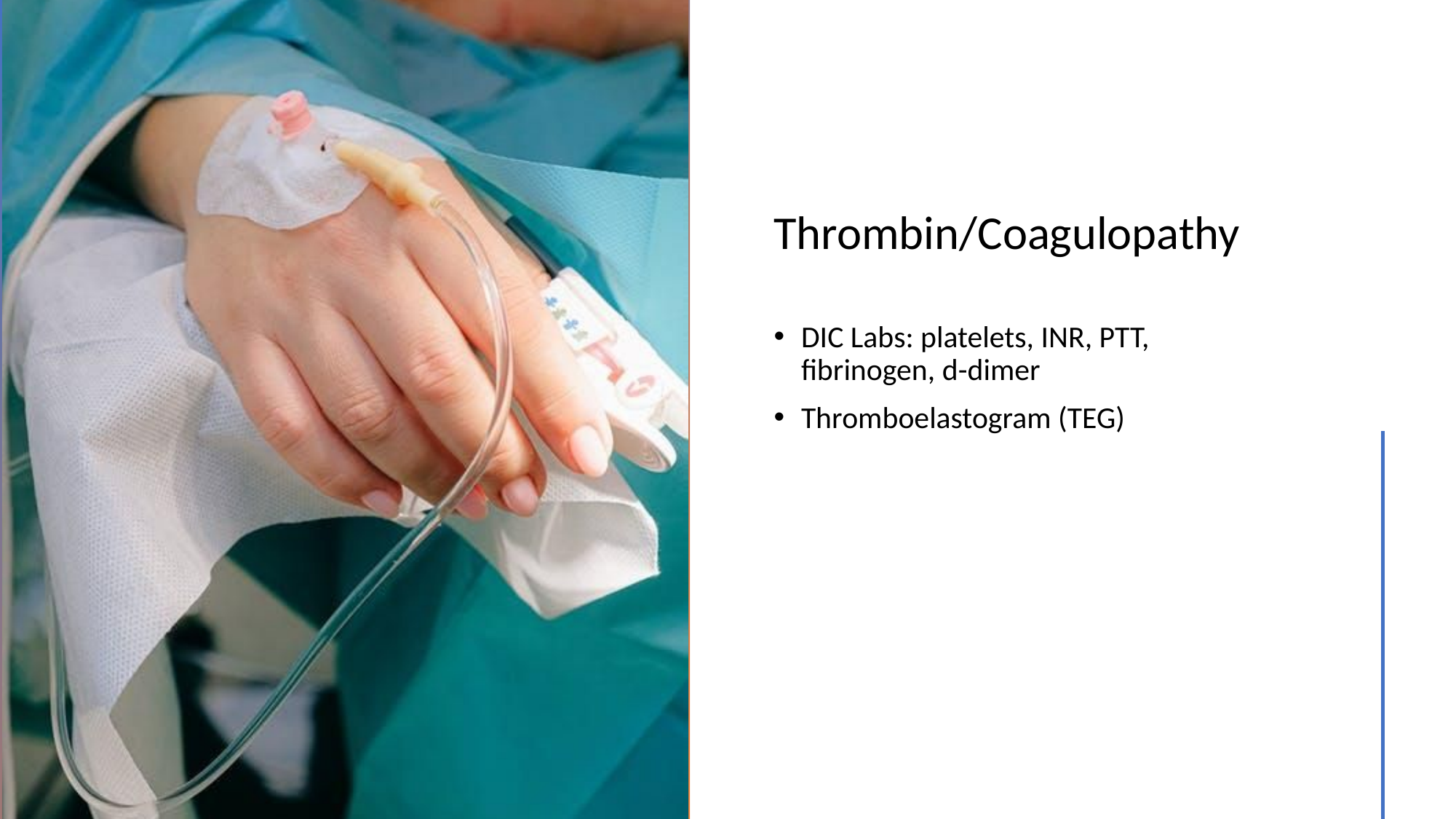

# Thrombin/Coagulopathy
DIC Labs: platelets, INR, PTT, fibrinogen, d-dimer
Thromboelastogram (TEG)

## Slide 30
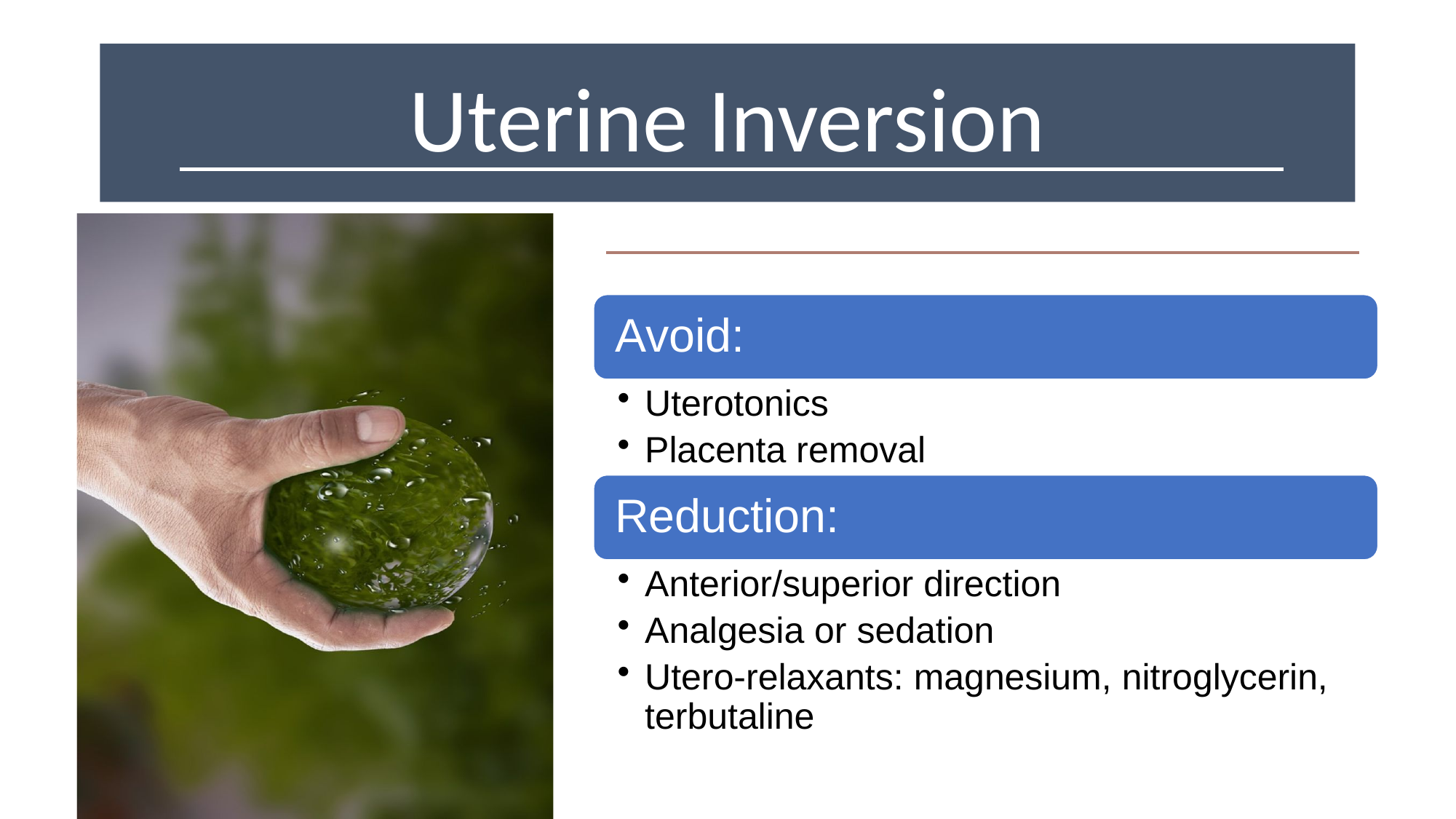

# Uterine Inversion

## Slide 31
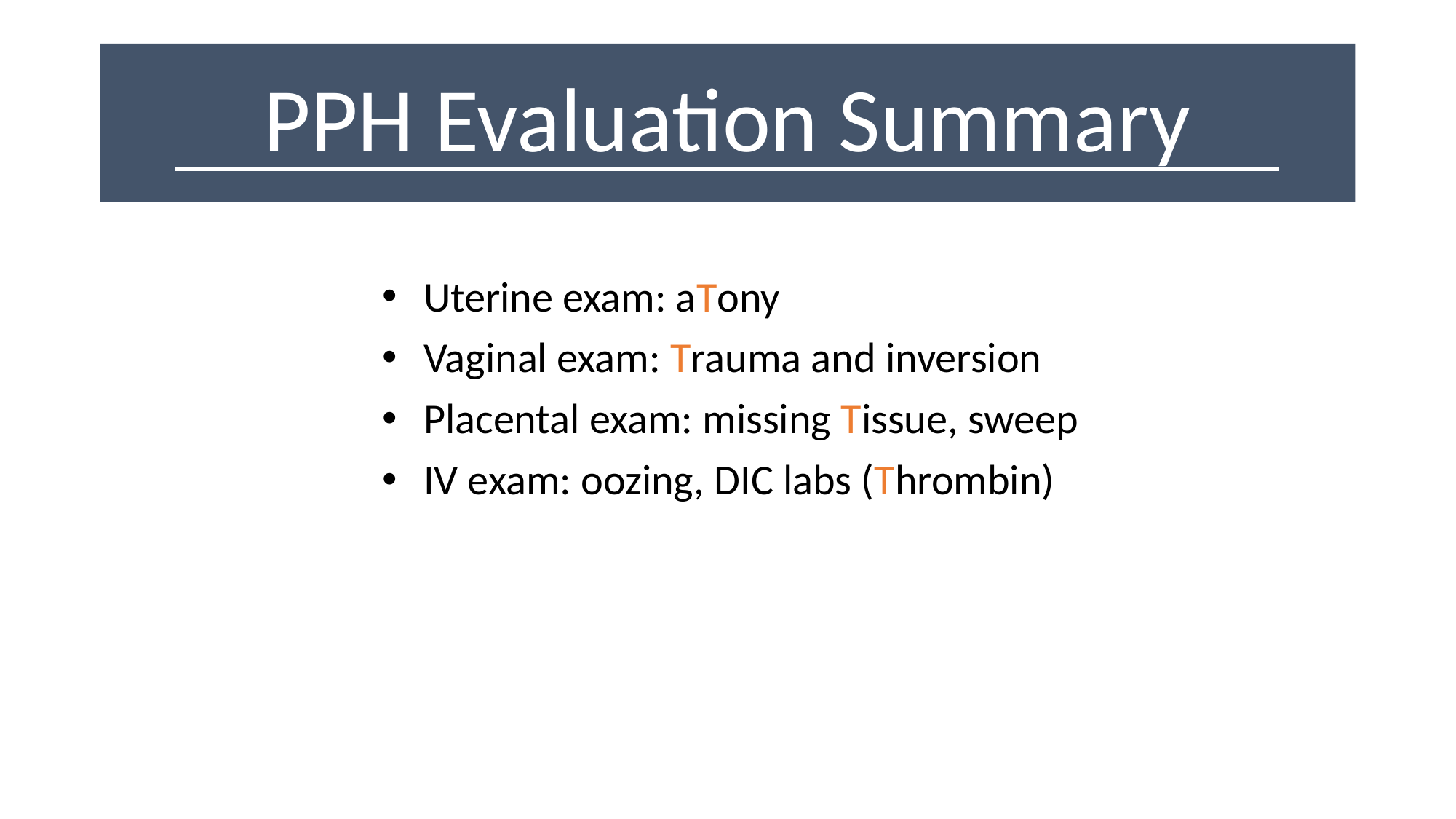

#
PPH Evaluation Summary
Uterine exam: aTony
Vaginal exam: Trauma and inversion
Placental exam: missing Tissue, sweep
IV exam: oozing, DIC labs (Thrombin)

## Slide 32
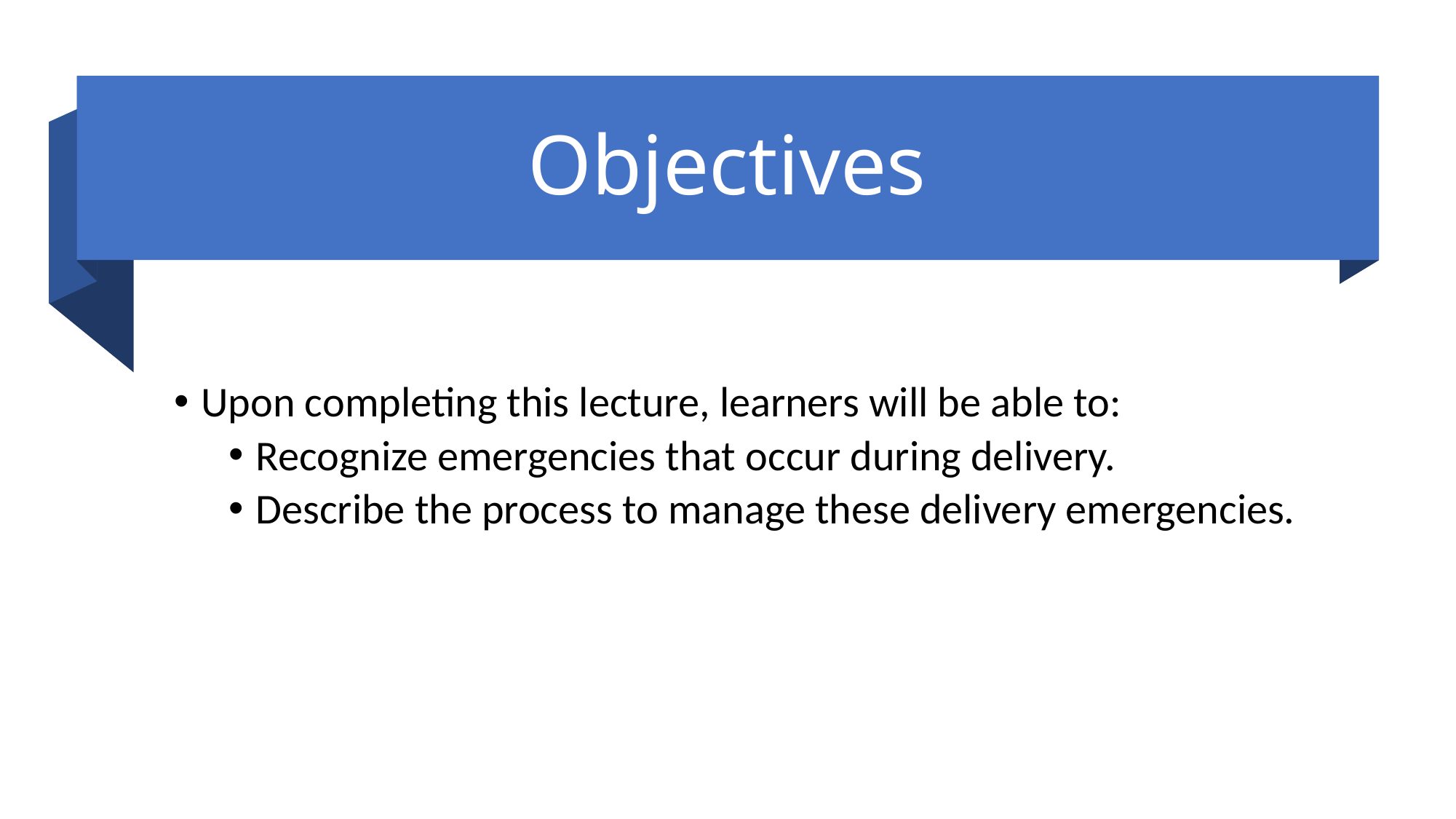

# Objectives
Upon completing this lecture, learners will be able to:
Recognize emergencies that occur during delivery.
Describe the process to manage these delivery emergencies.

## Slide 33
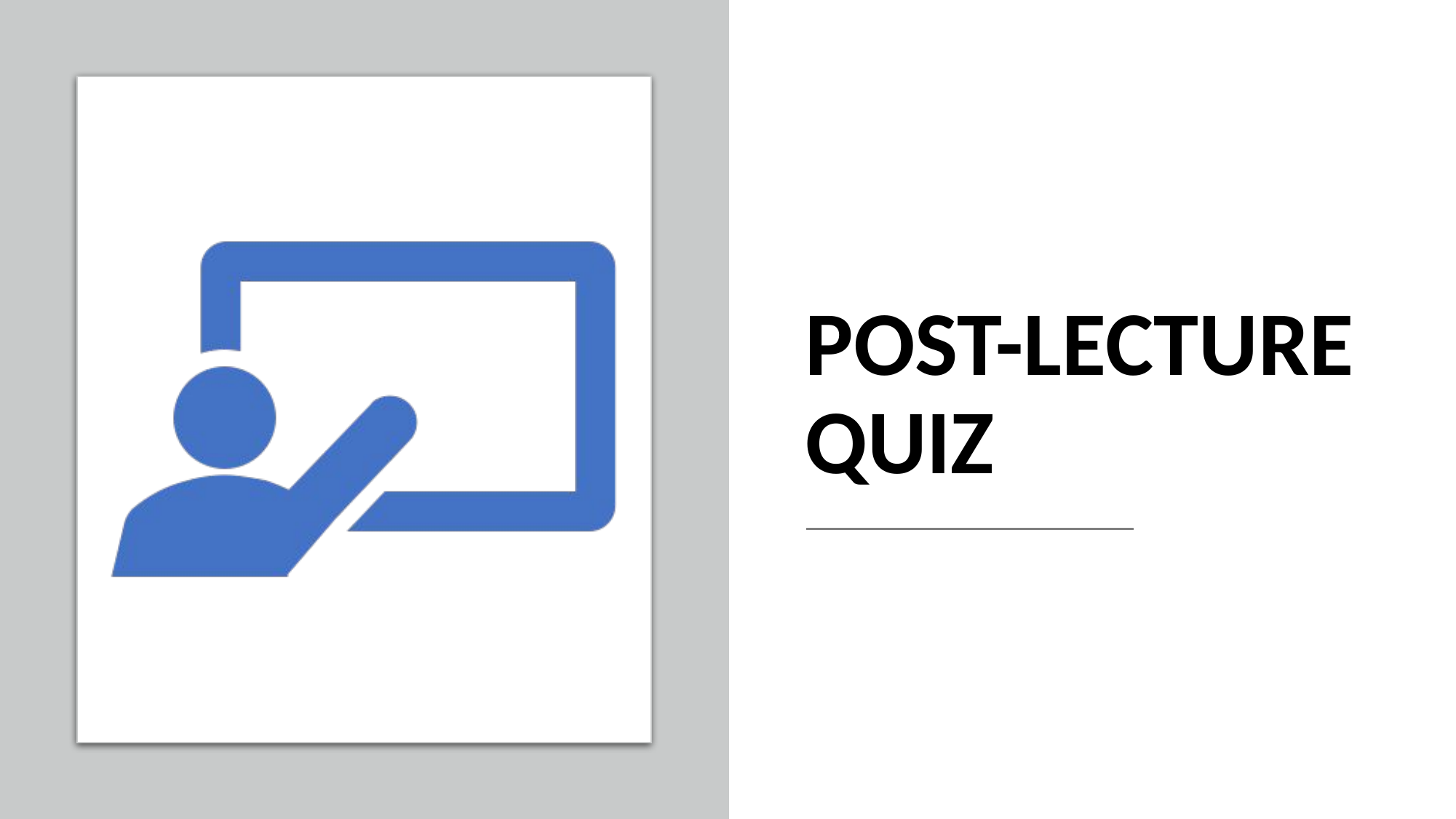

# POST-LECTURE QUIZ

## Slide 34
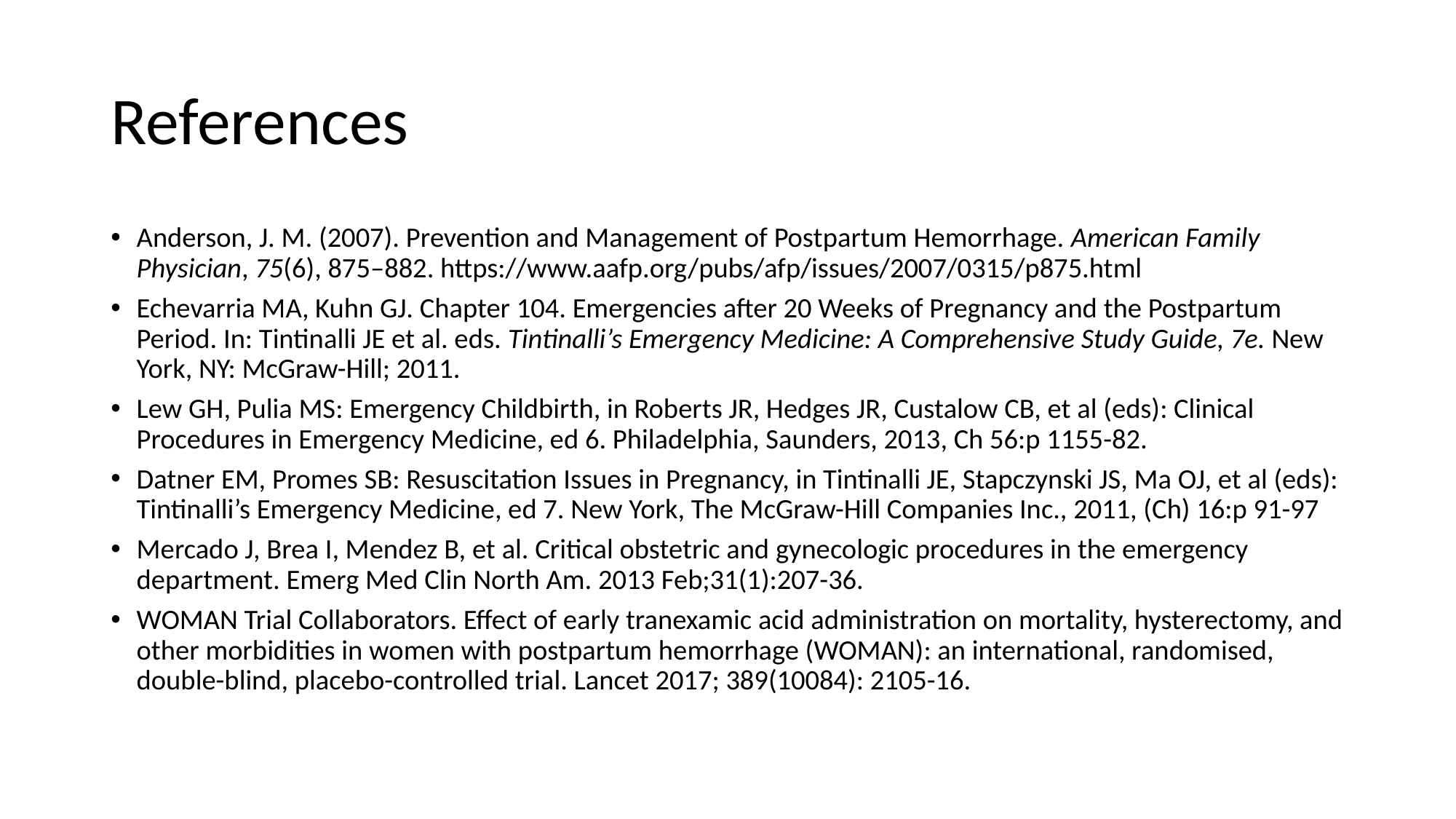

# References
Anderson, J. M. (2007). Prevention and Management of Postpartum Hemorrhage. American Family Physician, 75(6), 875–882. https://www.aafp.org/pubs/afp/issues/2007/0315/p875.html
Echevarria MA, Kuhn GJ. Chapter 104. Emergencies after 20 Weeks of Pregnancy and the Postpartum Period. In: Tintinalli JE et al. eds. Tintinalli’s Emergency Medicine: A Comprehensive Study Guide, 7e. New York, NY: McGraw-Hill; 2011.
Lew GH, Pulia MS: Emergency Childbirth, in Roberts JR, Hedges JR, Custalow CB, et al (eds): Clinical Procedures in Emergency Medicine, ed 6. Philadelphia, Saunders, 2013, Ch 56:p 1155-82.
Datner EM, Promes SB: Resuscitation Issues in Pregnancy, in Tintinalli JE, Stapczynski JS, Ma OJ, et al (eds): Tintinalli’s Emergency Medicine, ed 7. New York, The McGraw-Hill Companies Inc., 2011, (Ch) 16:p 91-97
Mercado J, Brea I, Mendez B, et al. Critical obstetric and gynecologic procedures in the emergency department. Emerg Med Clin North Am. 2013 Feb;31(1):207-36.
WOMAN Trial Collaborators. Effect of early tranexamic acid administration on mortality, hysterectomy, and other morbidities in women with postpartum hemorrhage (WOMAN): an international, randomised, double-blind, placebo-controlled trial. Lancet 2017; 389(10084): 2105-16.
