# Supplementary material for: Emergency Medicine Obstetrics and Gynecology: A Case-Based Curriculum for Residents
Source: MedEdPORTAL. 2023 Aug 11;19:11330. doi: 10.15766/mep_2374-8265.11330 (PMC10415535; doi:10.15766/mep_2374-8265.11330)
Supplement: Supplementary file 1 — Ectopic Pregnancy and Emergencies in the First 20 Weeks.pptxPregnancy Emergencies After 20 Weeks.pptxDelivery Emergencies.pptxPelvic Pain in the Nonpregnant Patient.pptxVaginitis, Cervicitis, and PID.pptxAbnormal Uterine Bleeding.pptxLabor and Perimortem C-Section.pptxSession Review Questions.docxPrecurriculum Survey.docxPostcurriculum Survey.docx [file mep_2374-8265.11330-s001.zip › D. Pelvic Pain in the Nonpregnant Patient.pptx]

## Slide 1
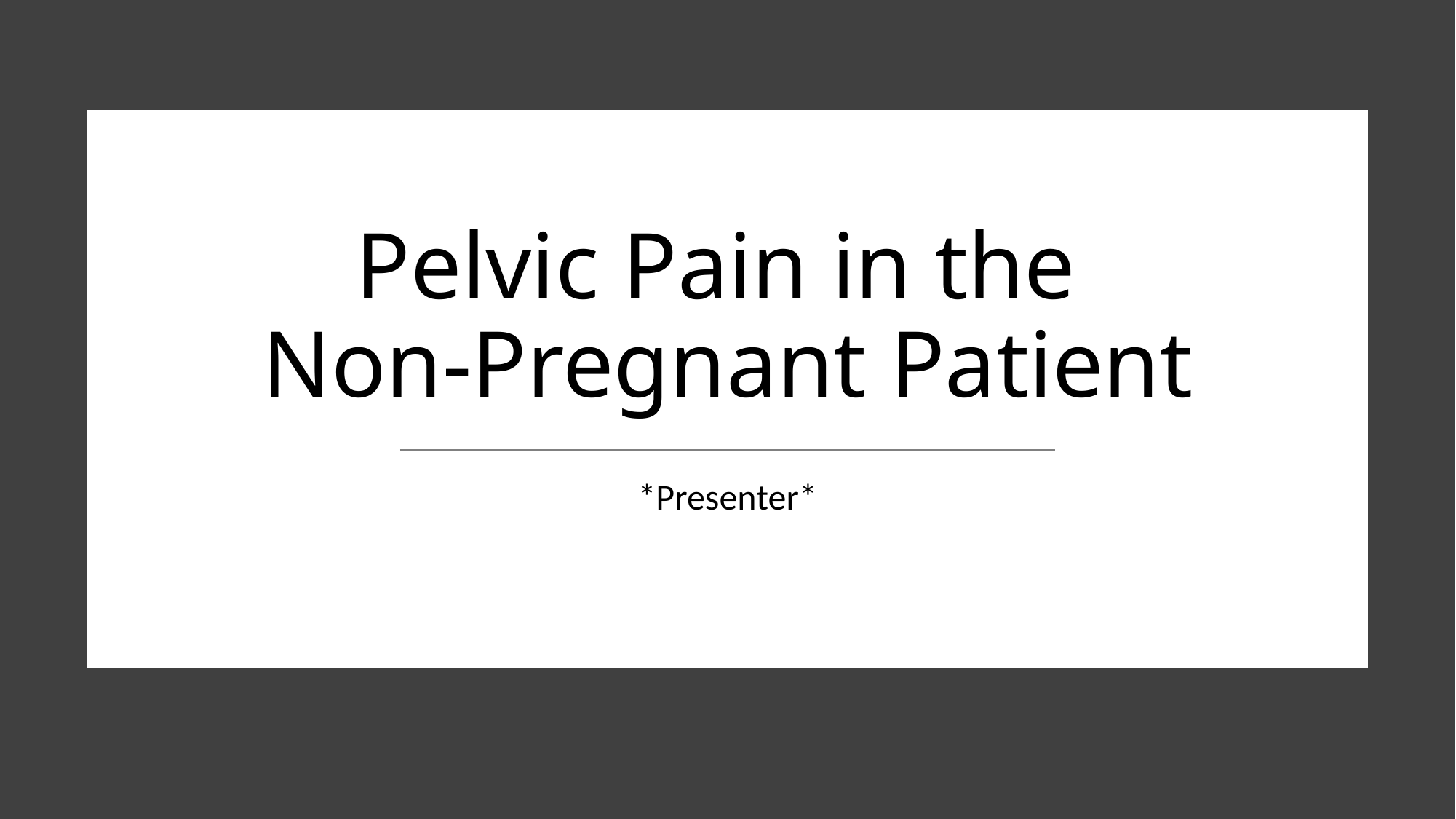

# Pelvic Pain in the Non-Pregnant Patient
*Presenter*

## Slide 2
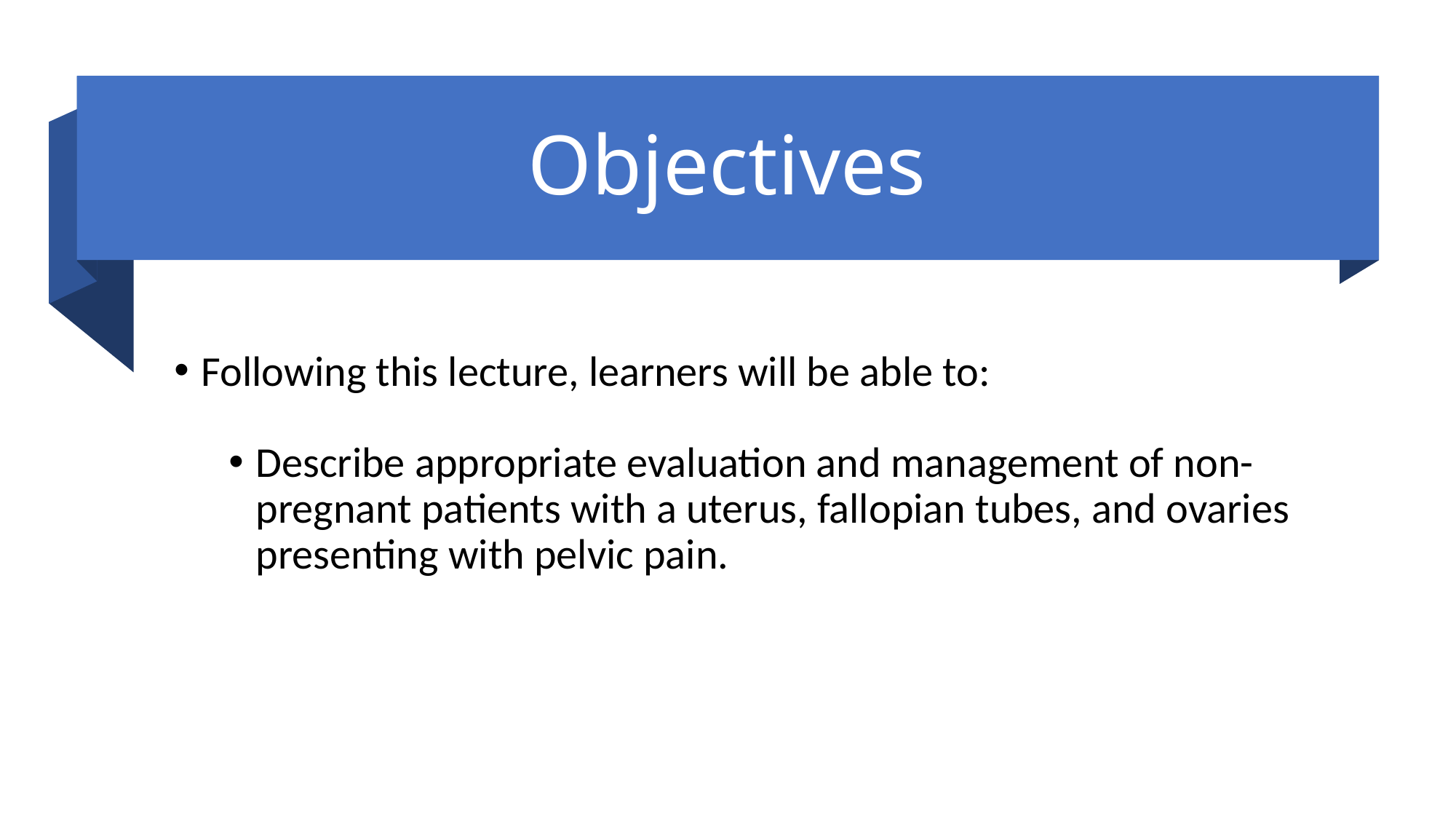

# Objectives
Following this lecture, learners will be able to:
Describe appropriate evaluation and management of non-pregnant patients with a uterus, fallopian tubes, and ovaries presenting with pelvic pain.

## Slide 3
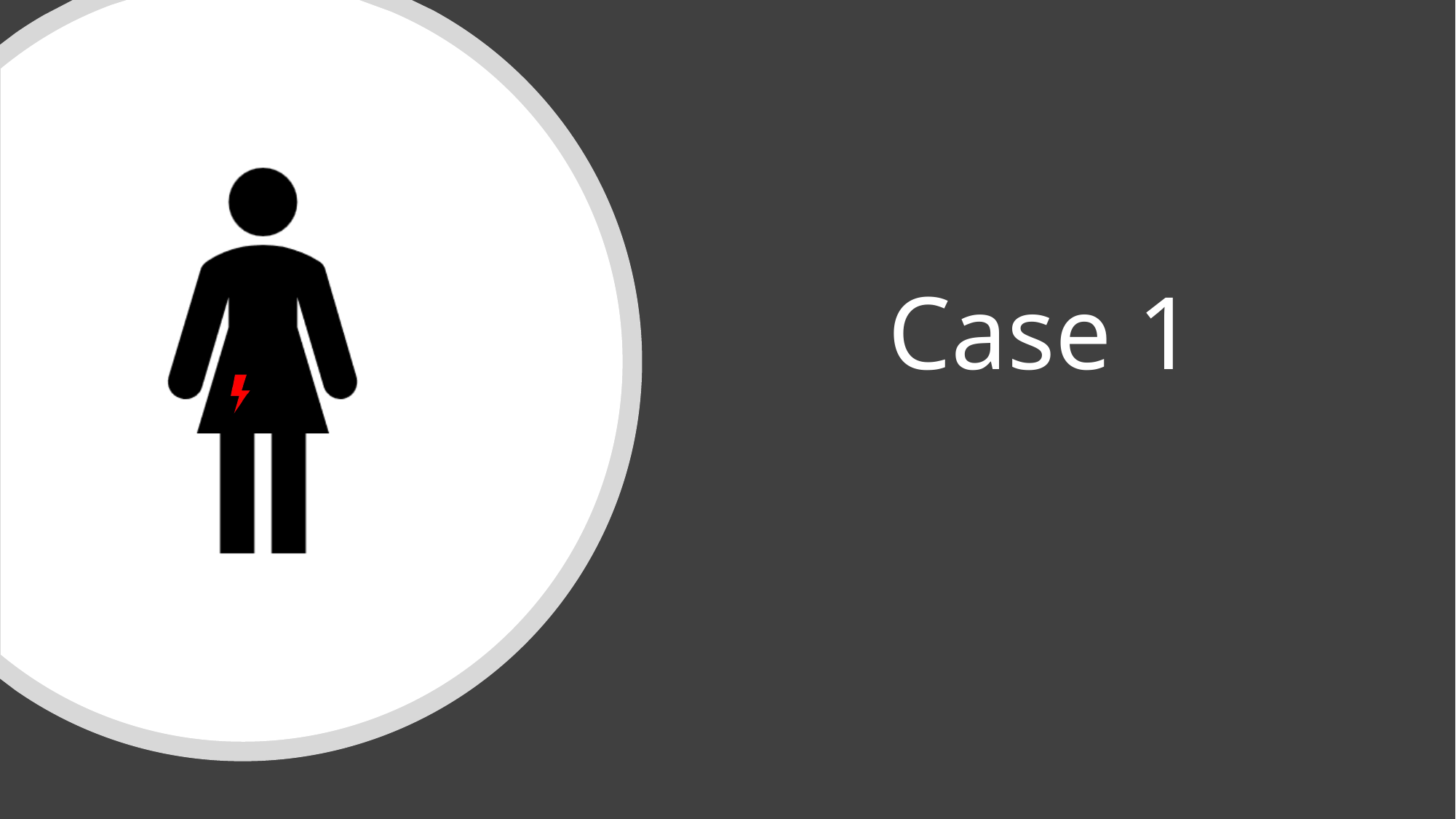

# Case 1

## Slide 4
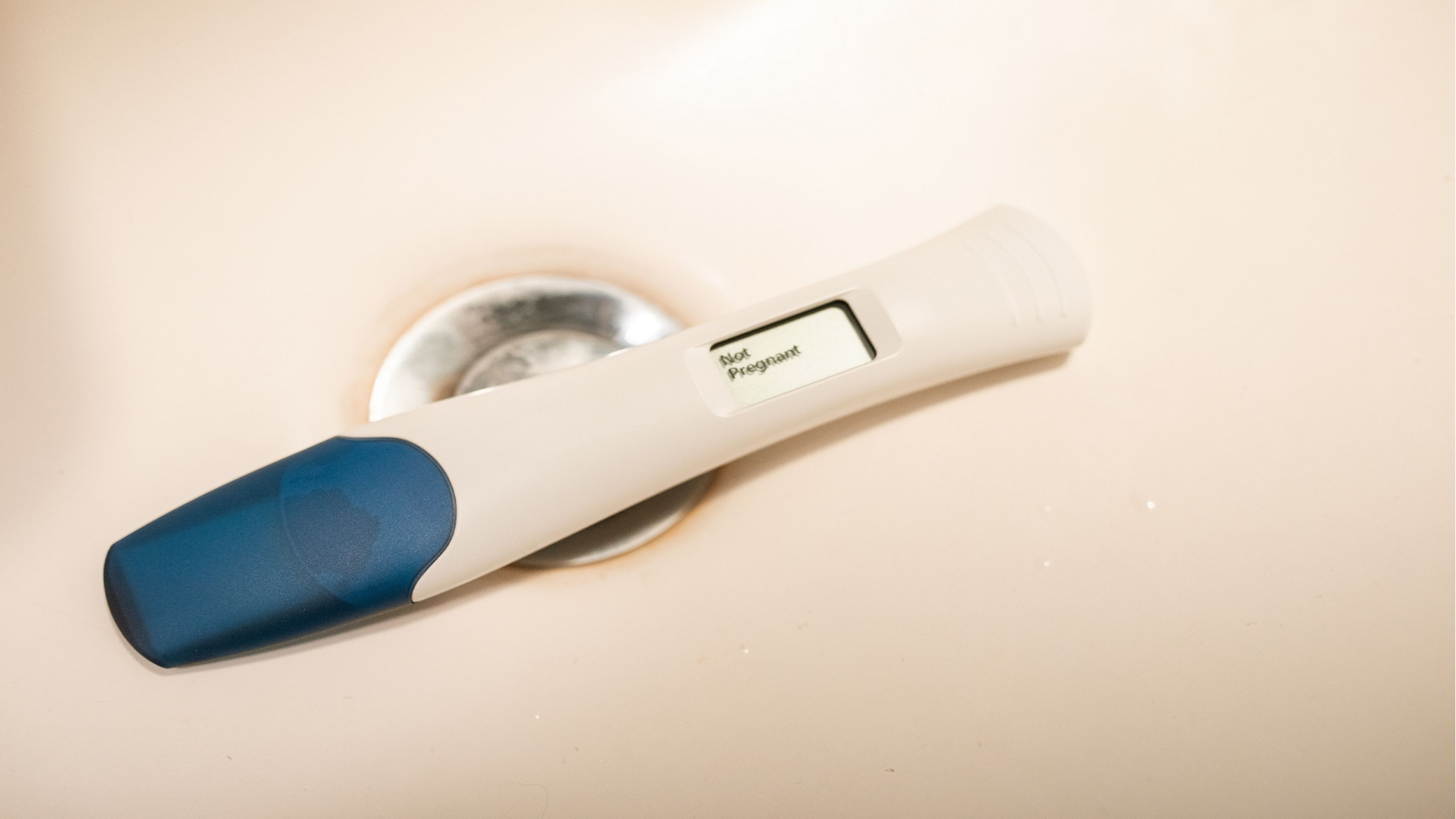

#

## Slide 5
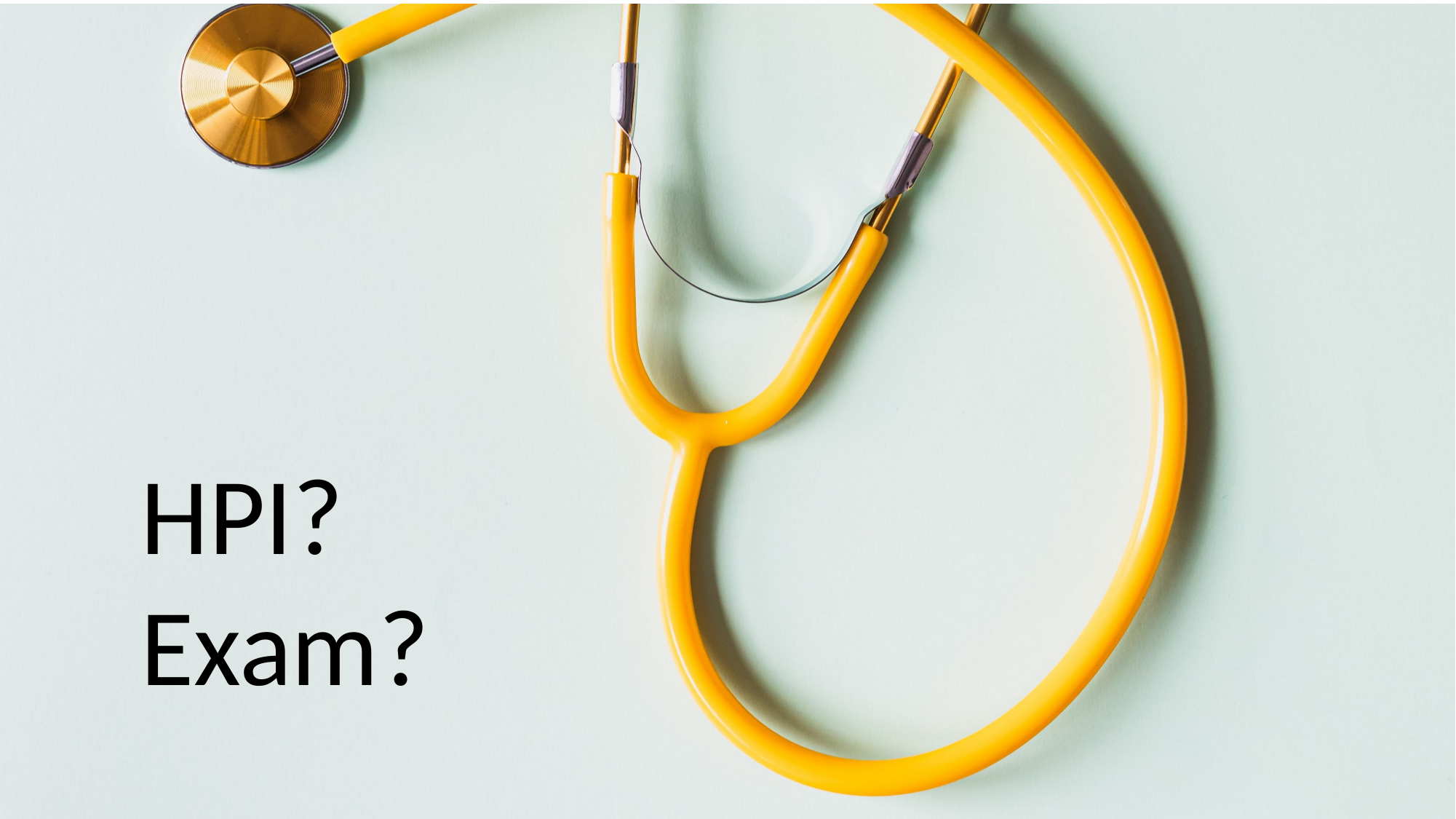

HPI?
Exam?

## Slide 6
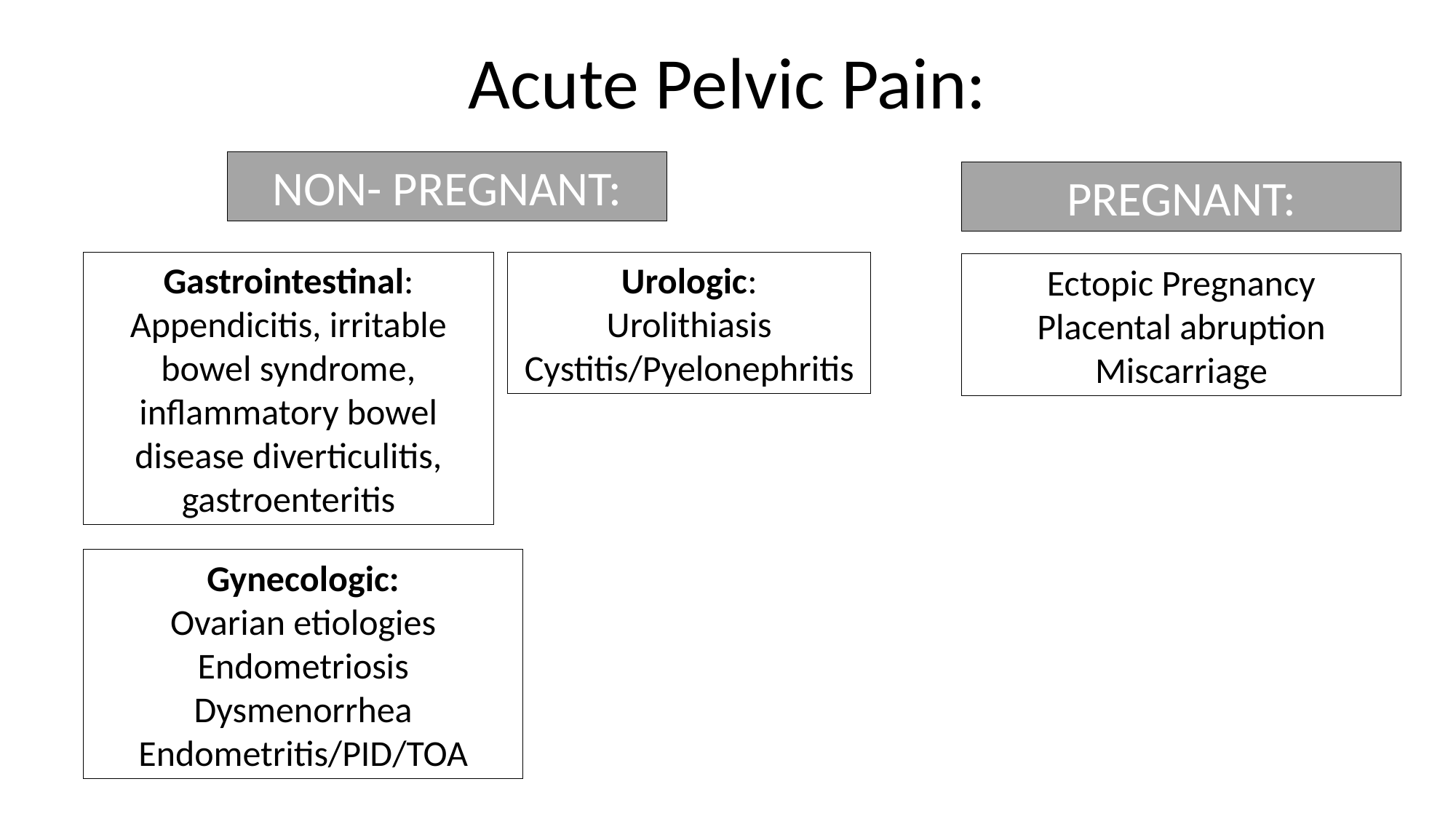

# Acute Pelvic Pain:
NON- PREGNANT:
PREGNANT:
Gastrointestinal:
Appendicitis, irritable bowel syndrome, inflammatory bowel disease diverticulitis, gastroenteritis
Urologic:
Urolithiasis
Cystitis/Pyelonephritis
Ectopic Pregnancy
Placental abruption
Miscarriage
Gynecologic:
Ovarian etiologies
Endometriosis
Dysmenorrhea
Endometritis/PID/TOA

## Slide 7
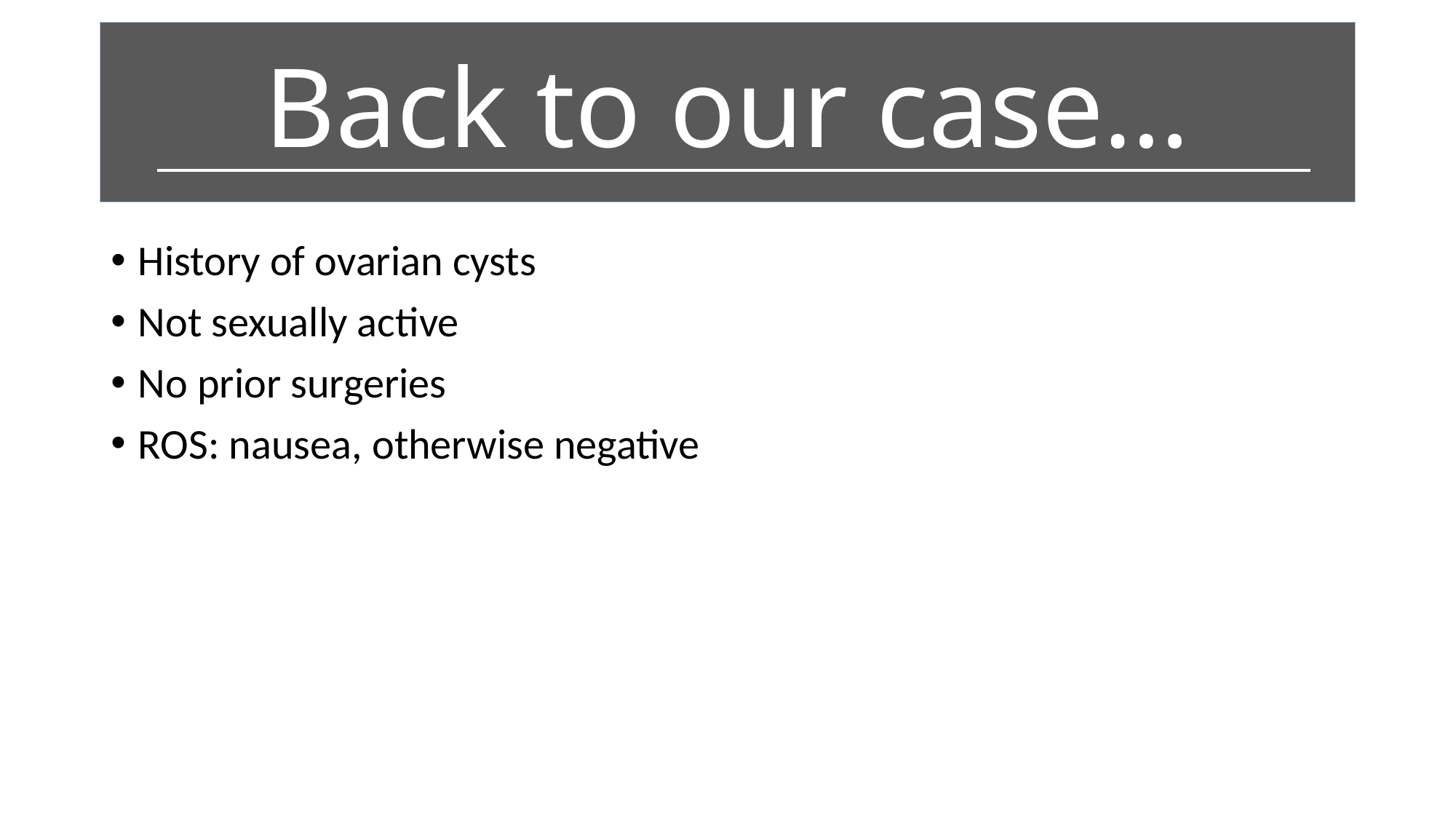

# Back to our case…
History of ovarian cysts
Not sexually active
No prior surgeries
ROS: nausea, otherwise negative

## Slide 8
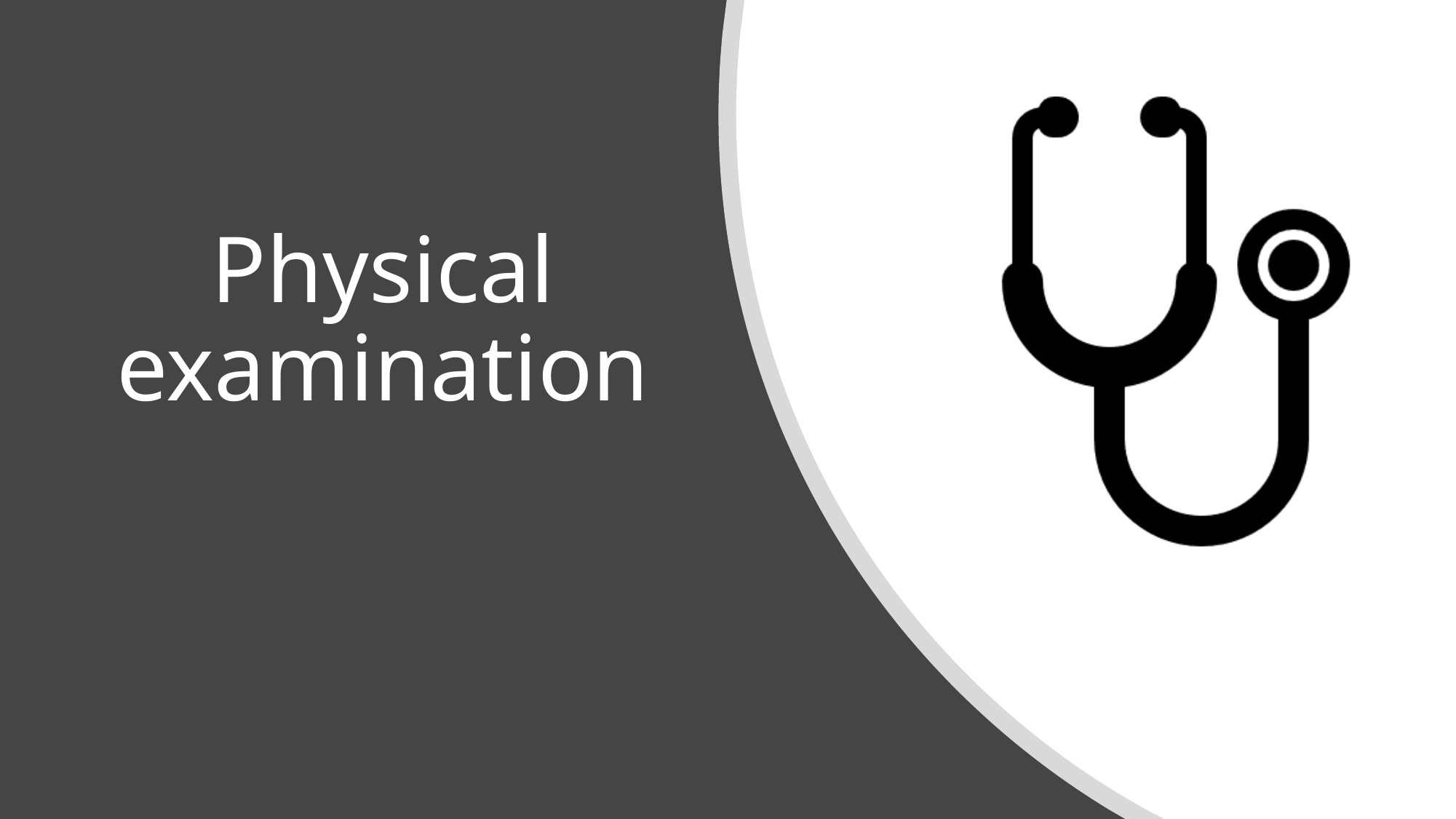

# Physical examination

## Slide 9
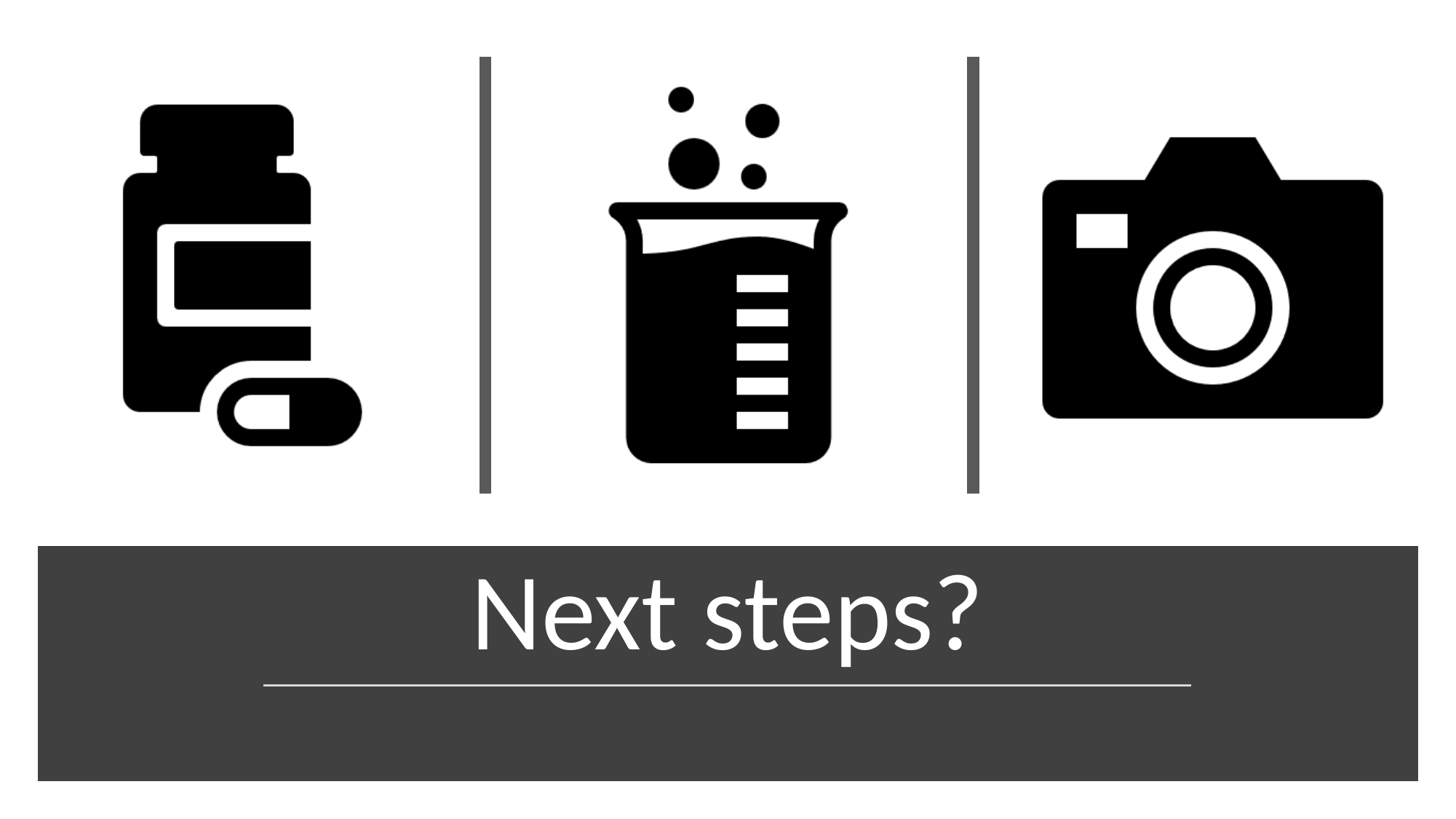

# Next steps?

## Slide 10
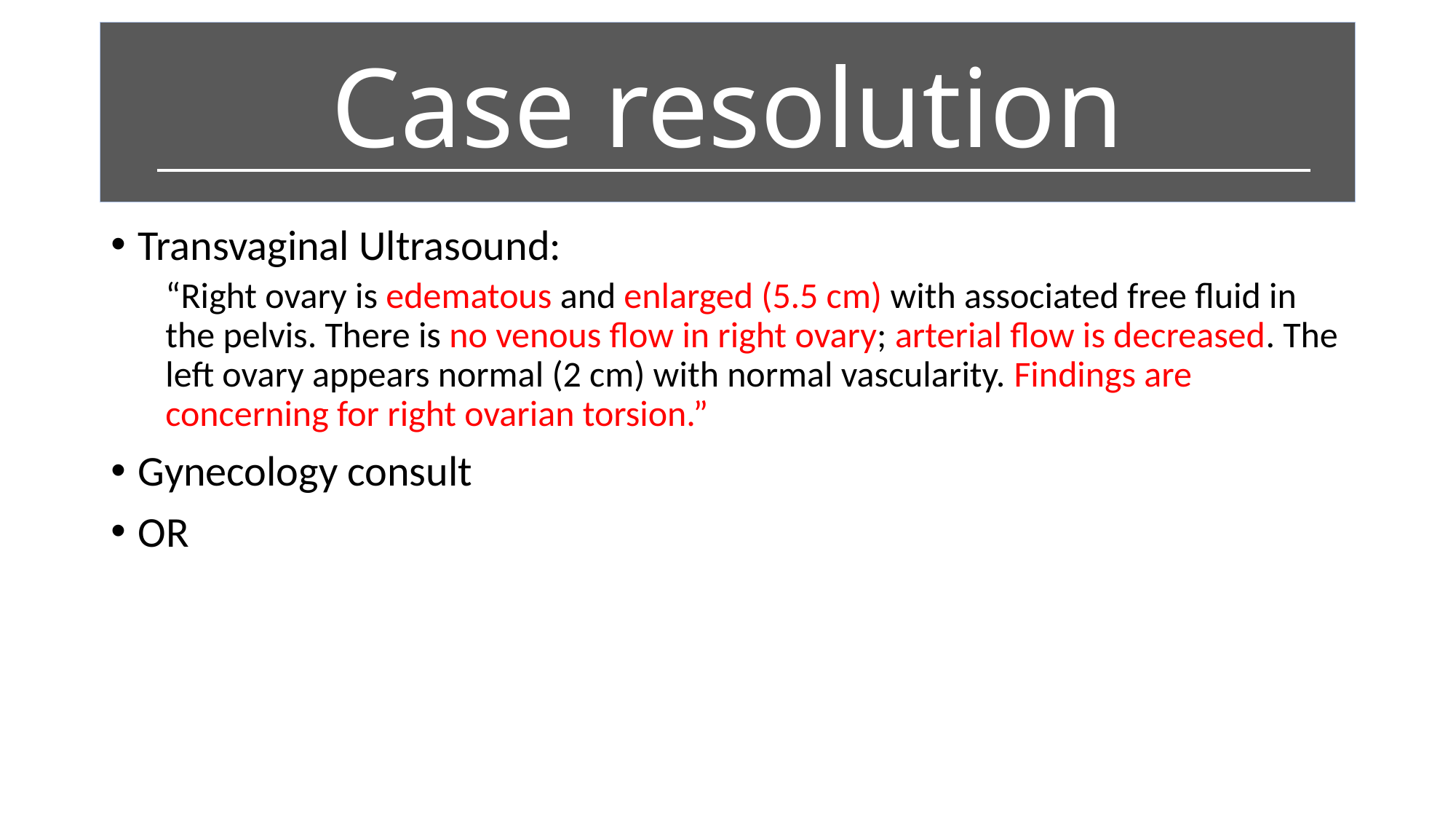

Case resolution
#
Transvaginal Ultrasound:
“Right ovary is edematous and enlarged (5.5 cm) with associated free fluid in the pelvis. There is no venous flow in right ovary; arterial flow is decreased. The left ovary appears normal (2 cm) with normal vascularity. Findings are concerning for right ovarian torsion.”
Gynecology consult
OR

## Slide 11
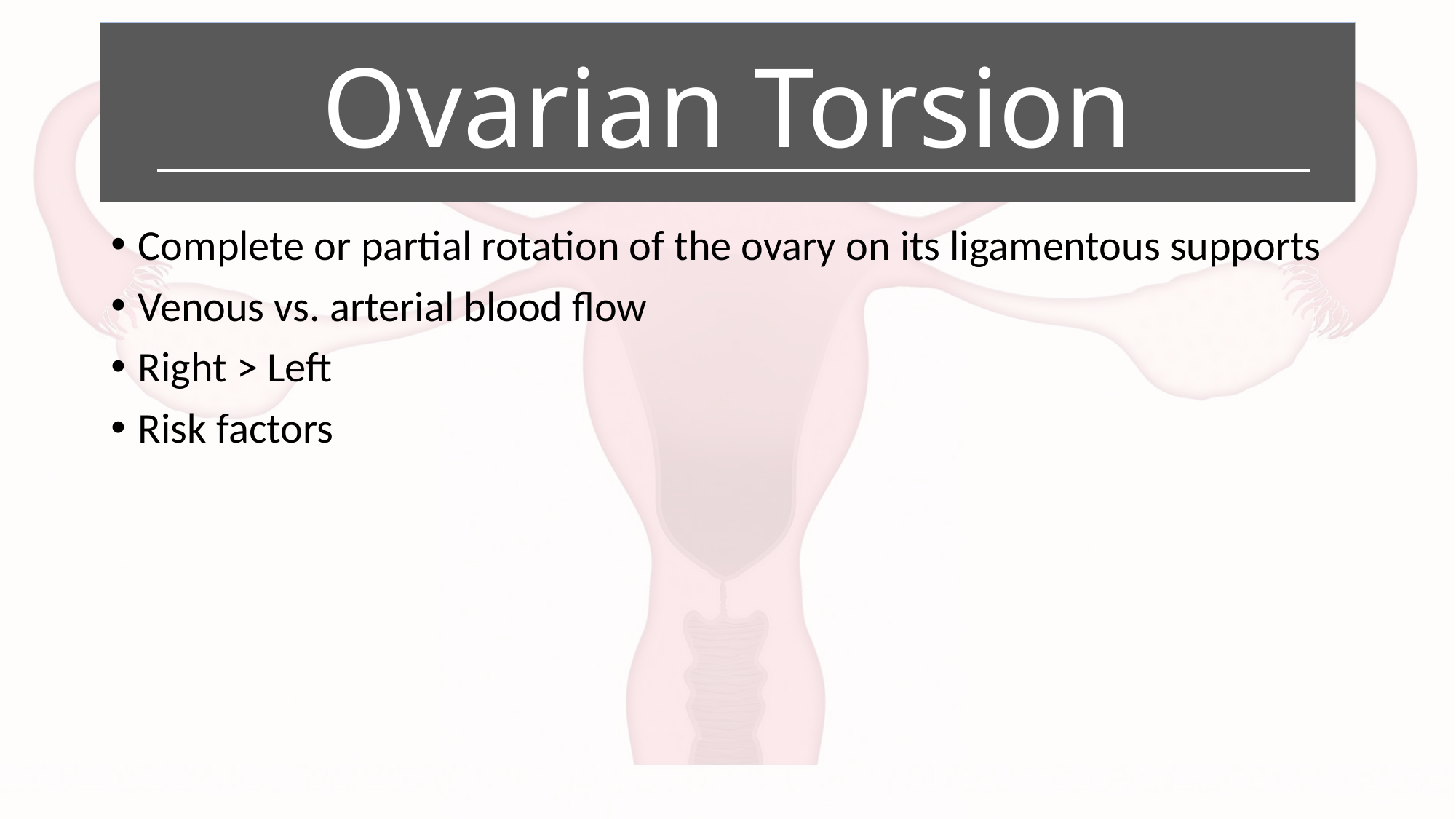

Ovarian Torsion
#
Complete or partial rotation of the ovary on its ligamentous supports
Venous vs. arterial blood flow
Right > Left
Risk factors

## Slide 12
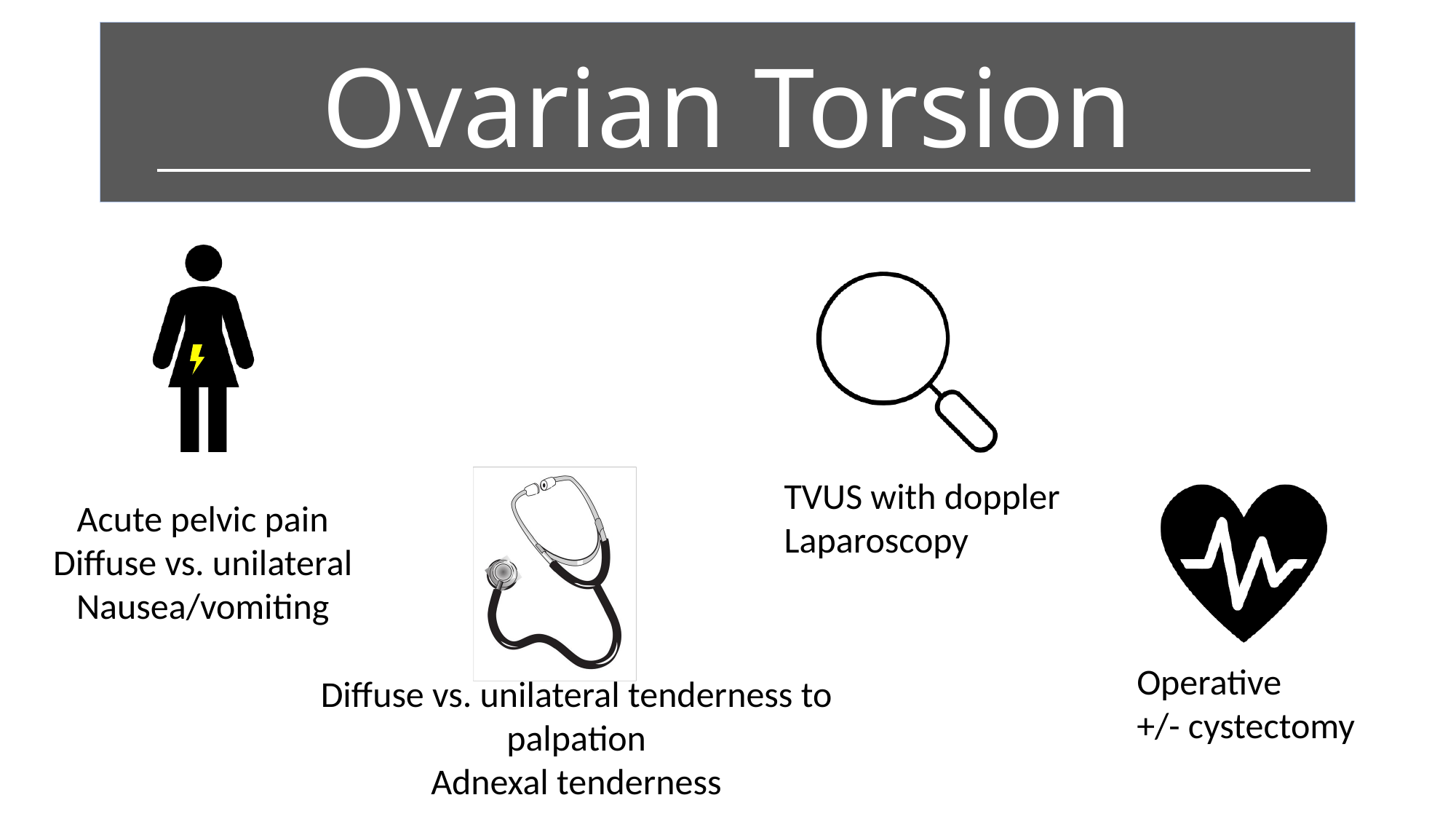

Ovarian Torsion
#
TVUS with doppler
Laparoscopy
Acute pelvic pain
Diffuse vs. unilateral
Nausea/vomiting
Operative
+/- cystectomy
Diffuse vs. unilateral tenderness to palpation
Adnexal tenderness

## Slide 13
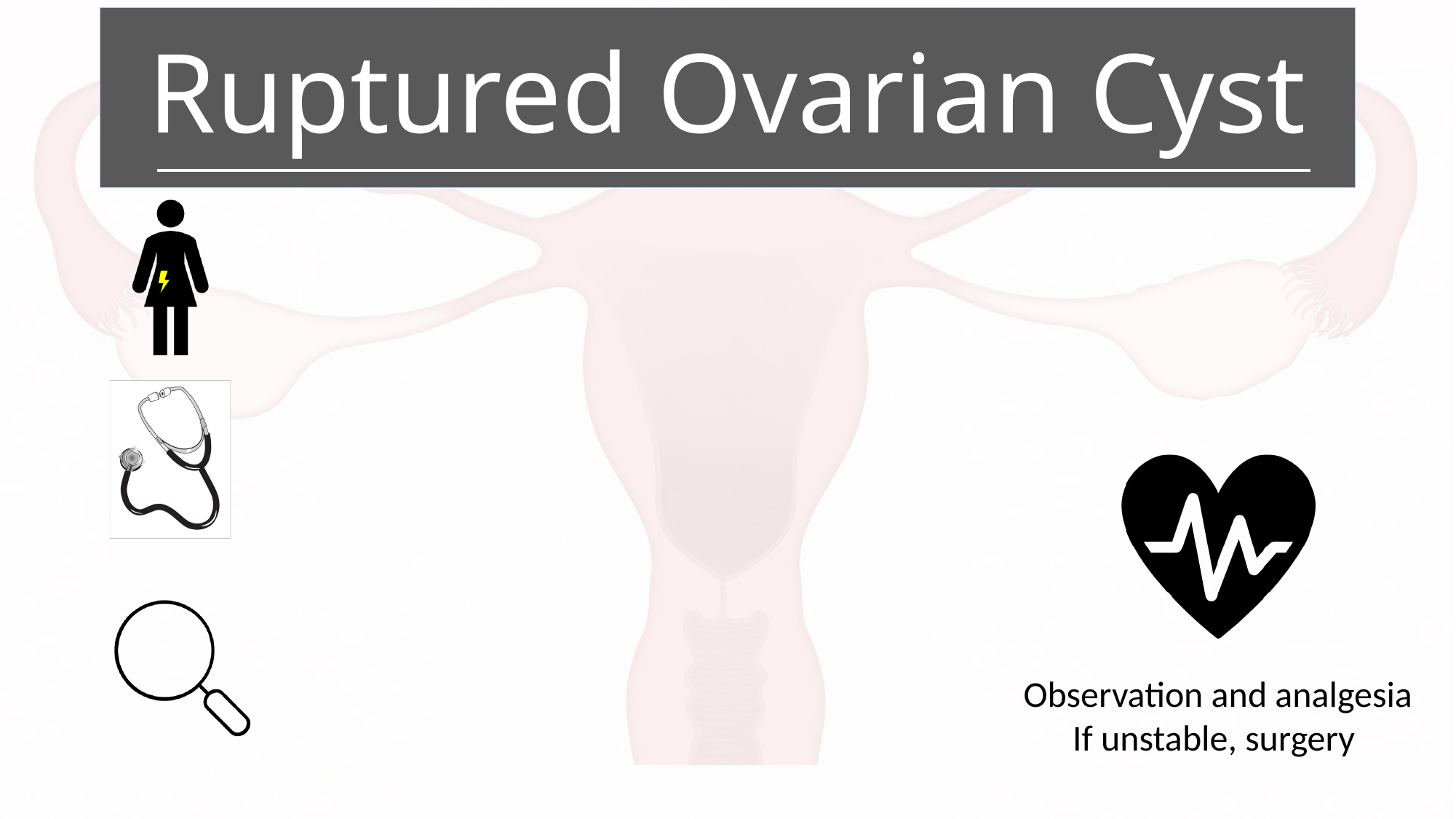

Ruptured Ovarian Cyst
Observation and analgesia
If unstable, surgery

## Slide 14
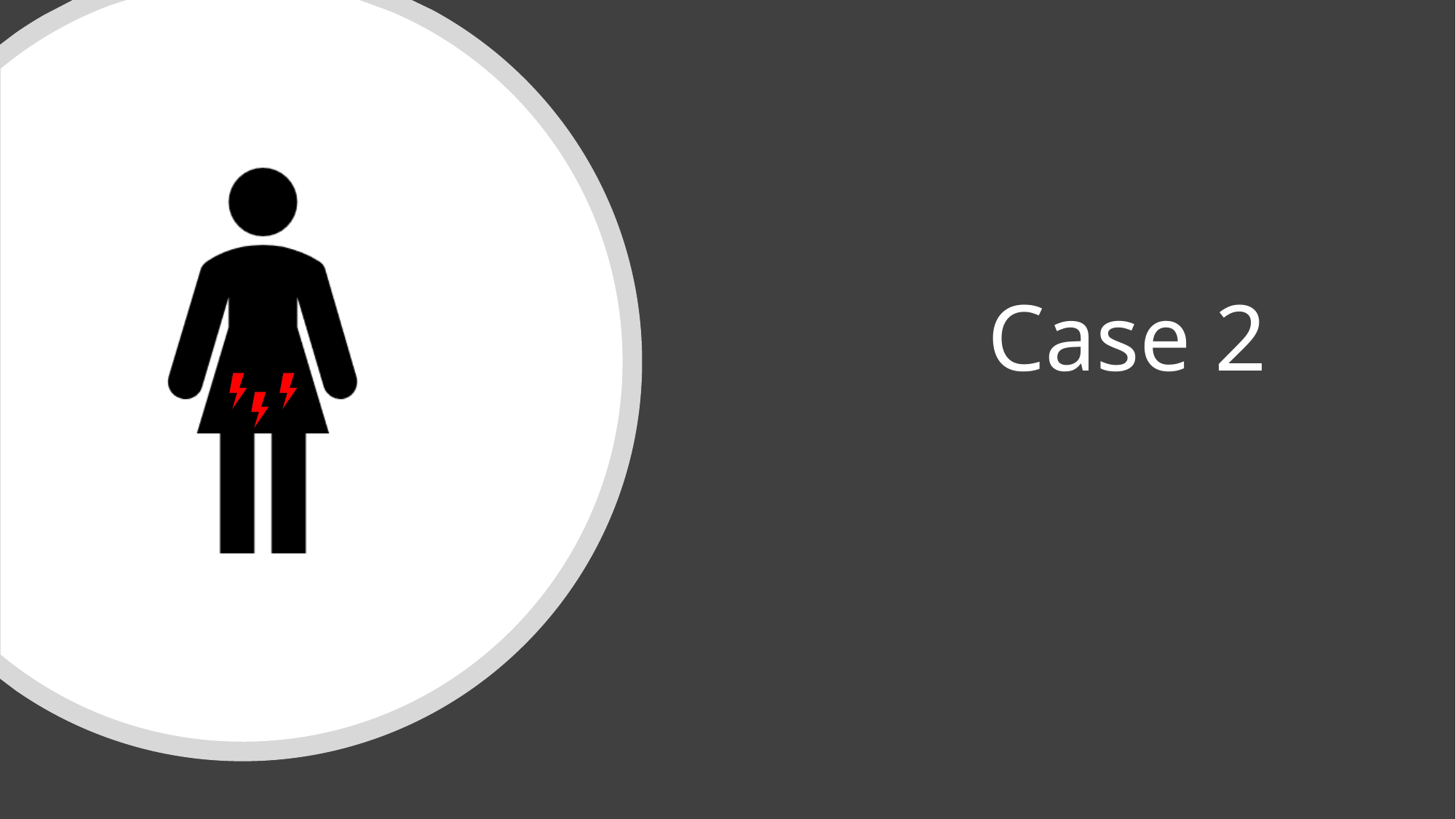

# Case 2

## Slide 15
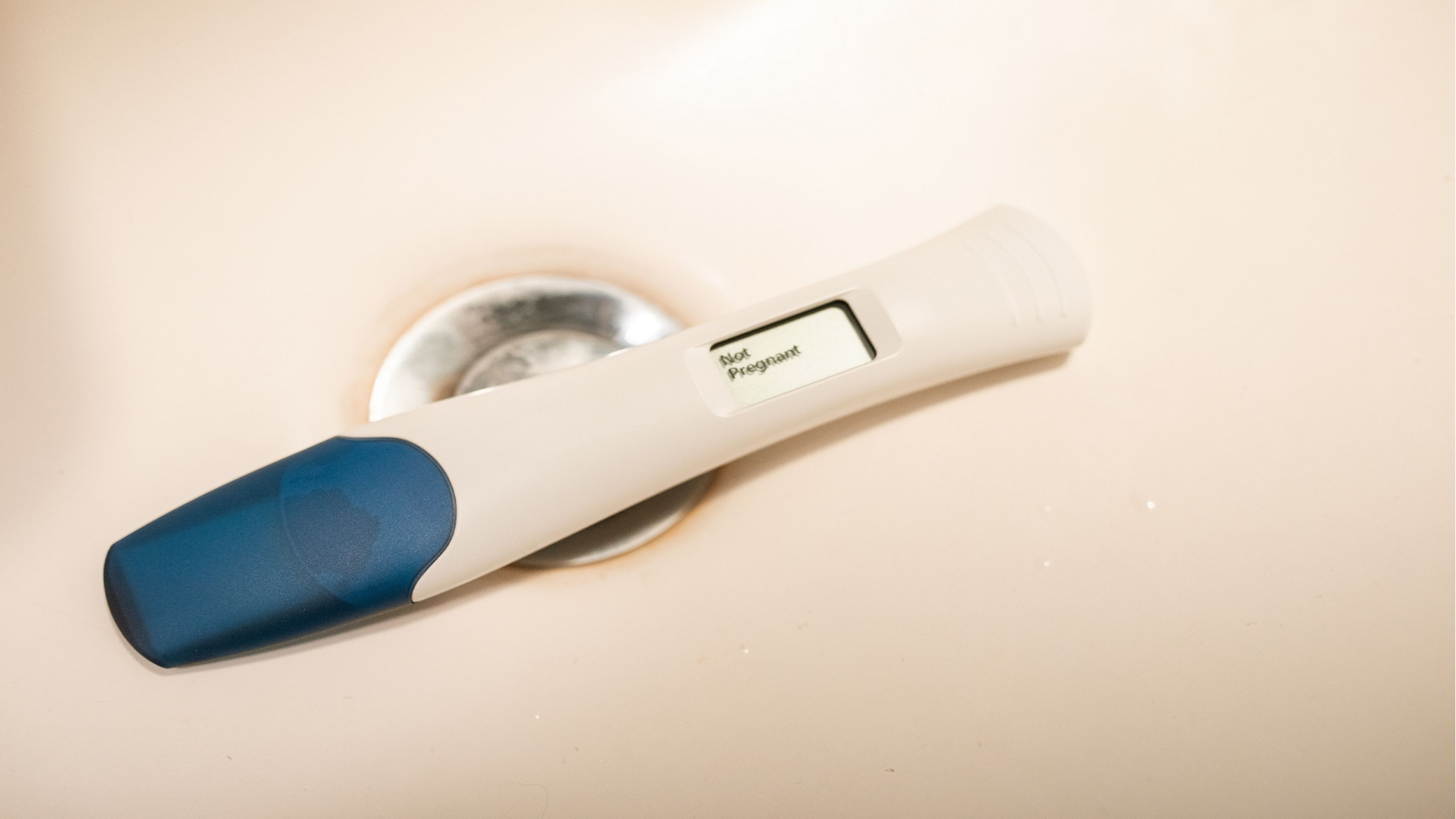

#

## Slide 16
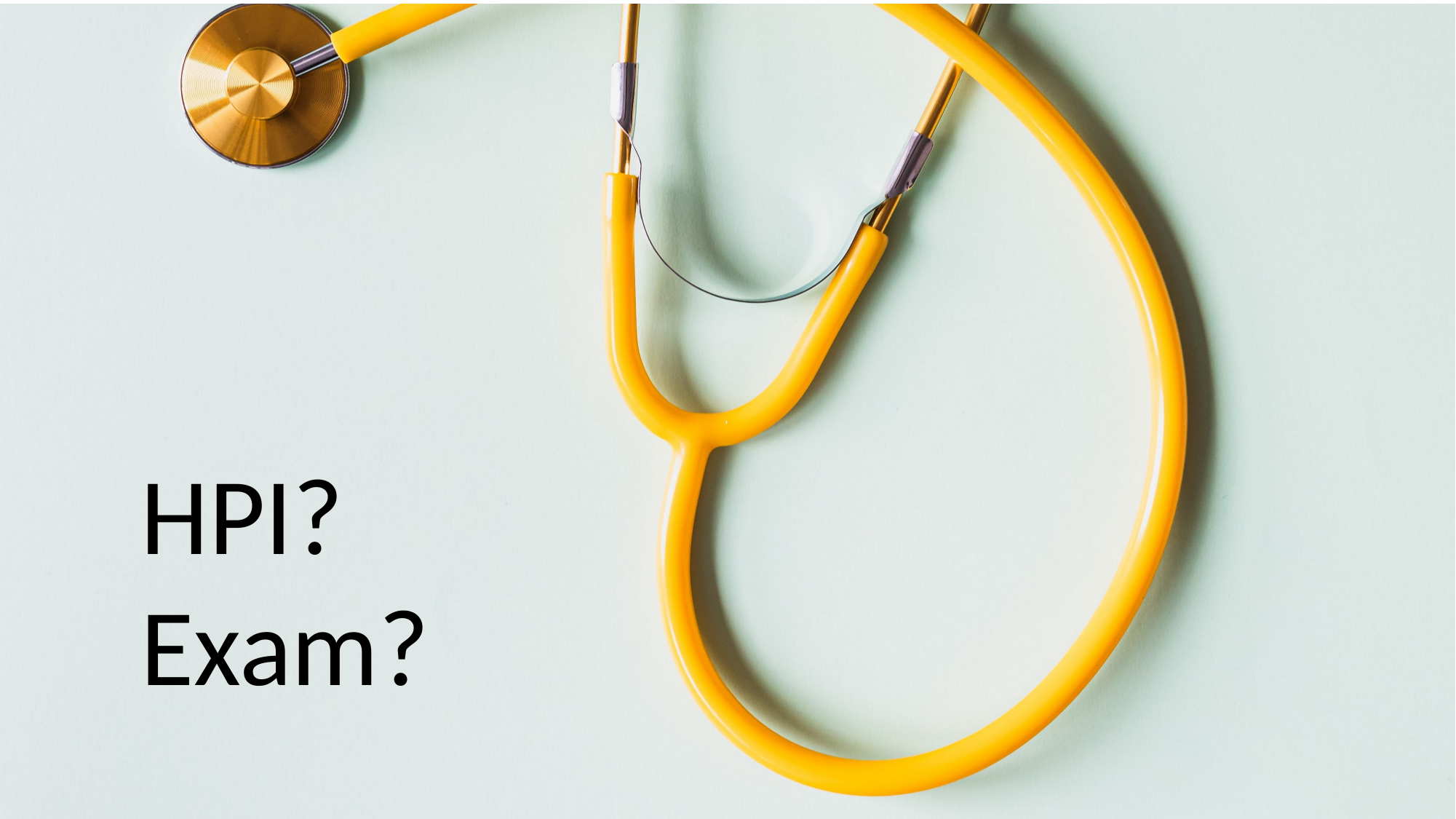

HPI?
Exam?

## Slide 17
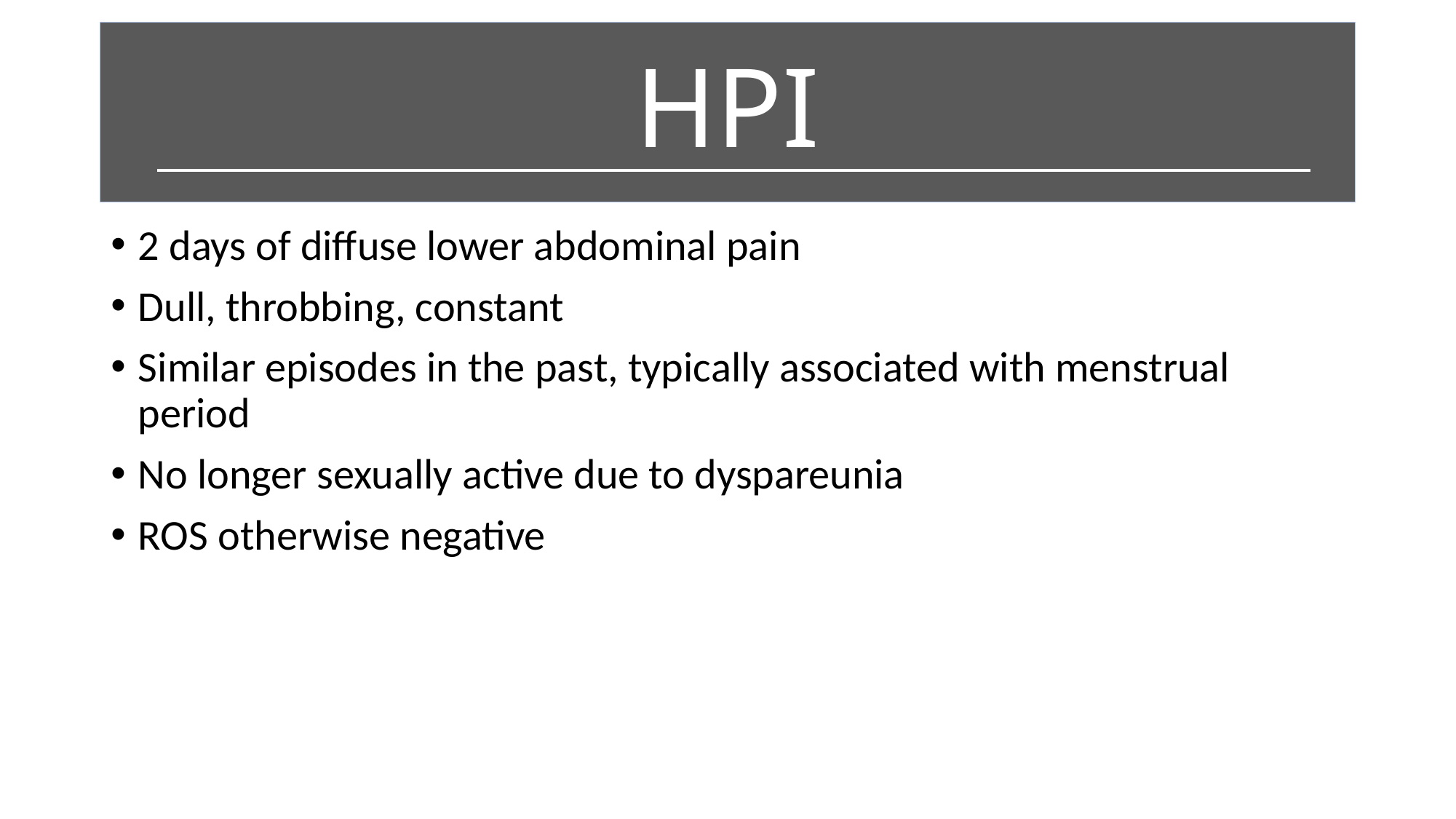

HPI
#
2 days of diffuse lower abdominal pain
Dull, throbbing, constant
Similar episodes in the past, typically associated with menstrual period
No longer sexually active due to dyspareunia
ROS otherwise negative

## Slide 18
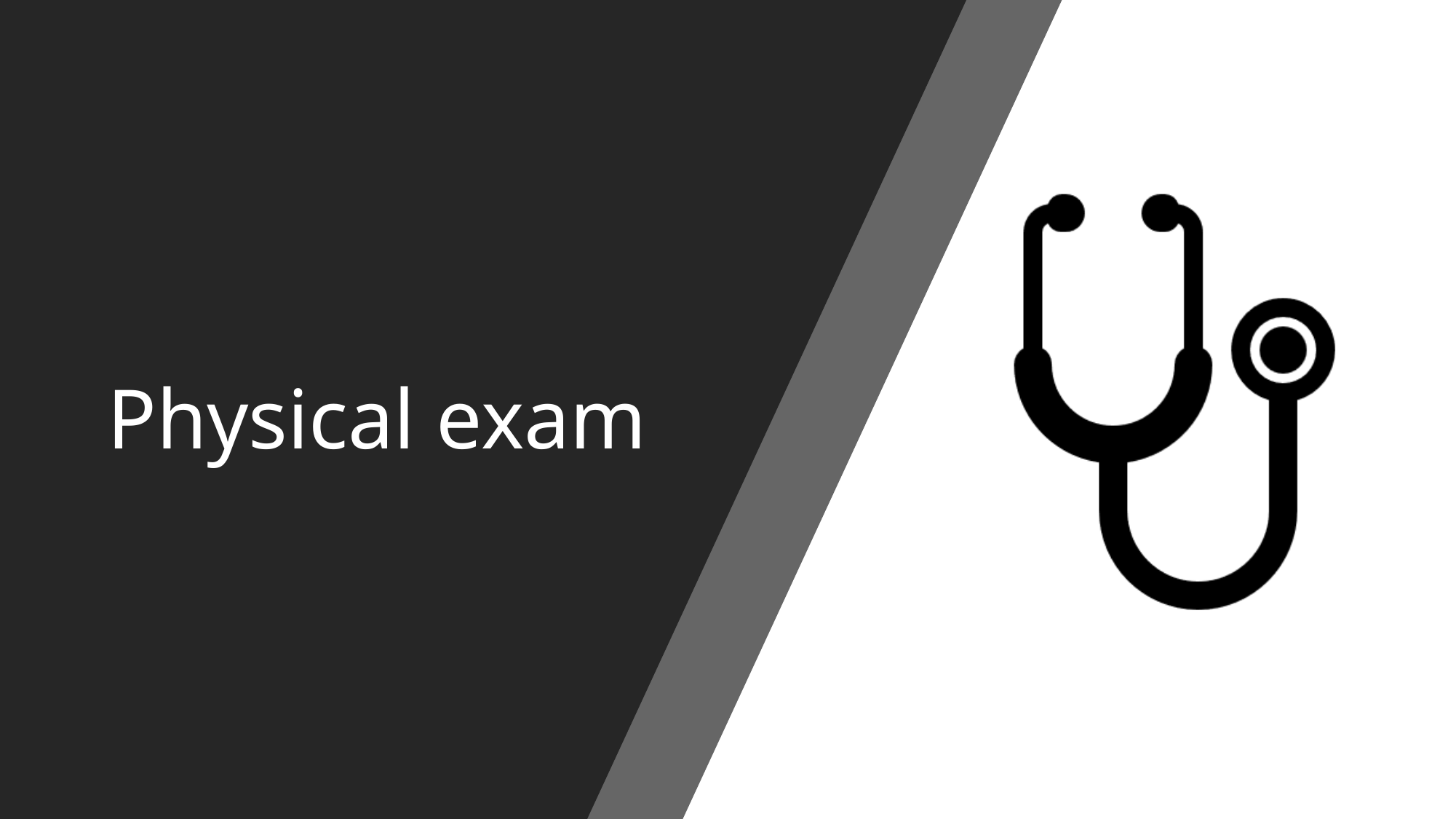

# Physical exam

## Slide 19
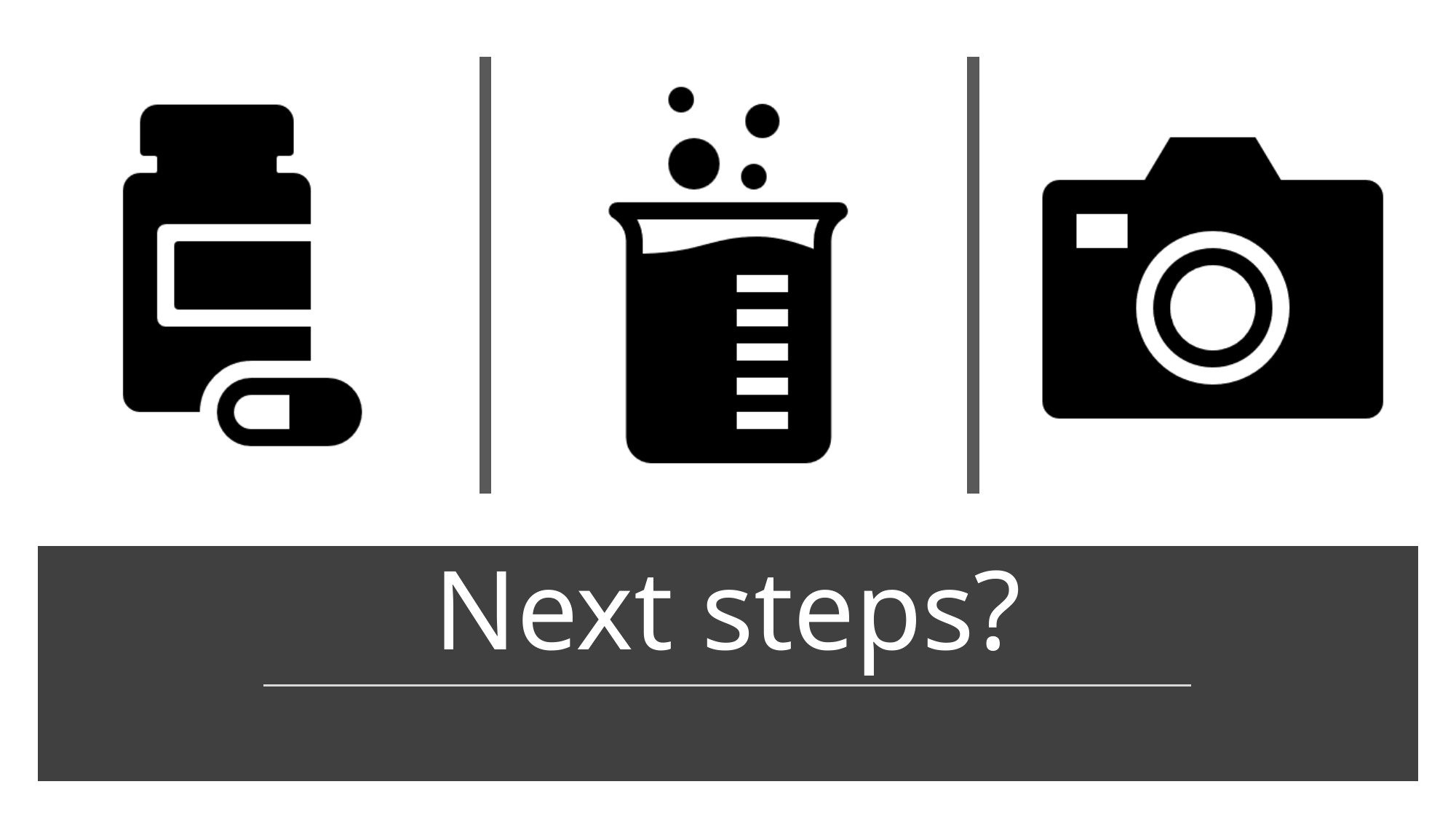

# Next steps?

## Slide 20
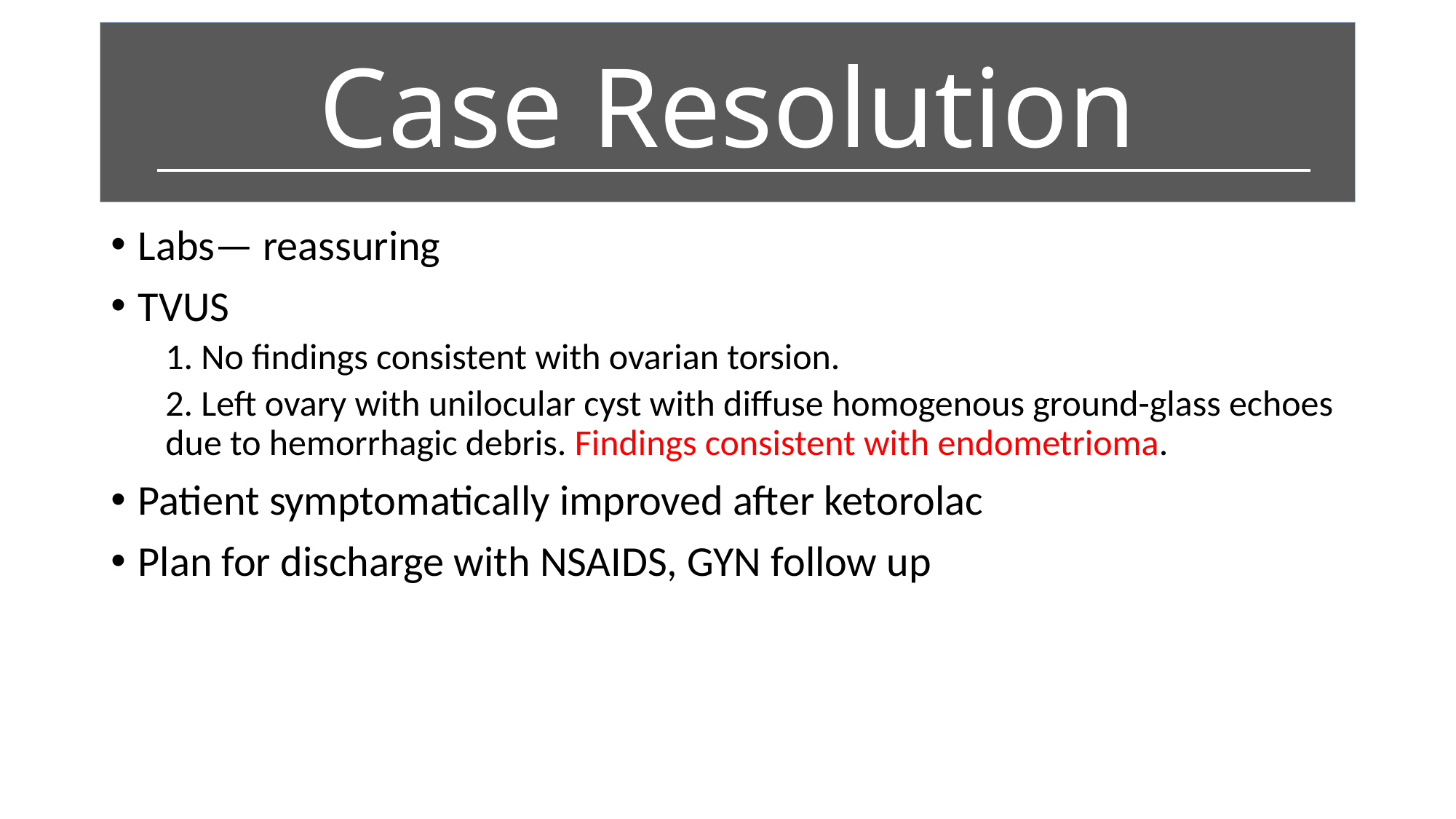

Case Resolution
#
Labs— reassuring
TVUS
1. No findings consistent with ovarian torsion.
2. Left ovary with unilocular cyst with diffuse homogenous ground-glass echoes due to hemorrhagic debris. Findings consistent with endometrioma.
Patient symptomatically improved after ketorolac
Plan for discharge with NSAIDS, GYN follow up

## Slide 21
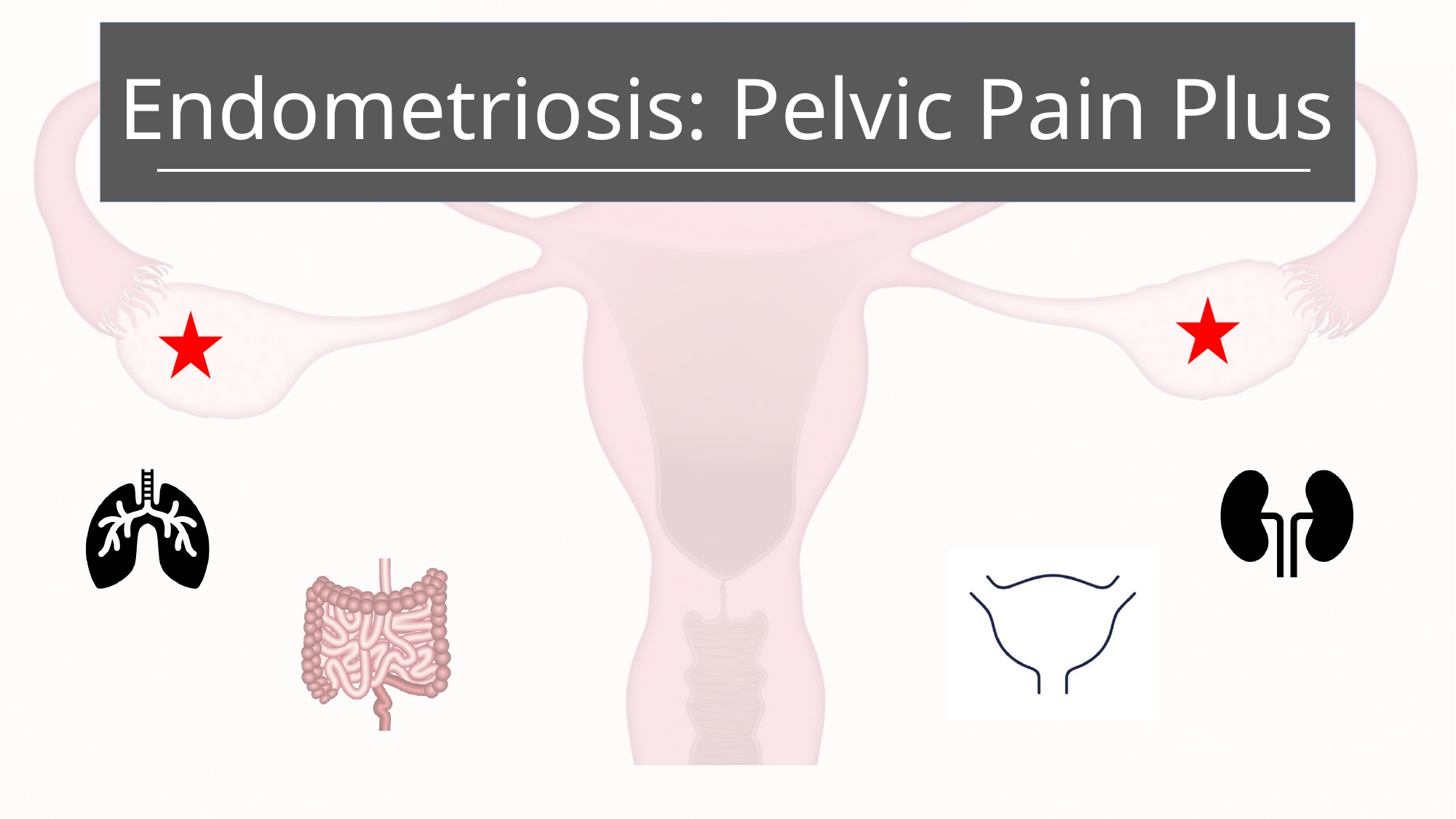

Endometriosis: Pelvic Pain Plus
#

## Slide 22
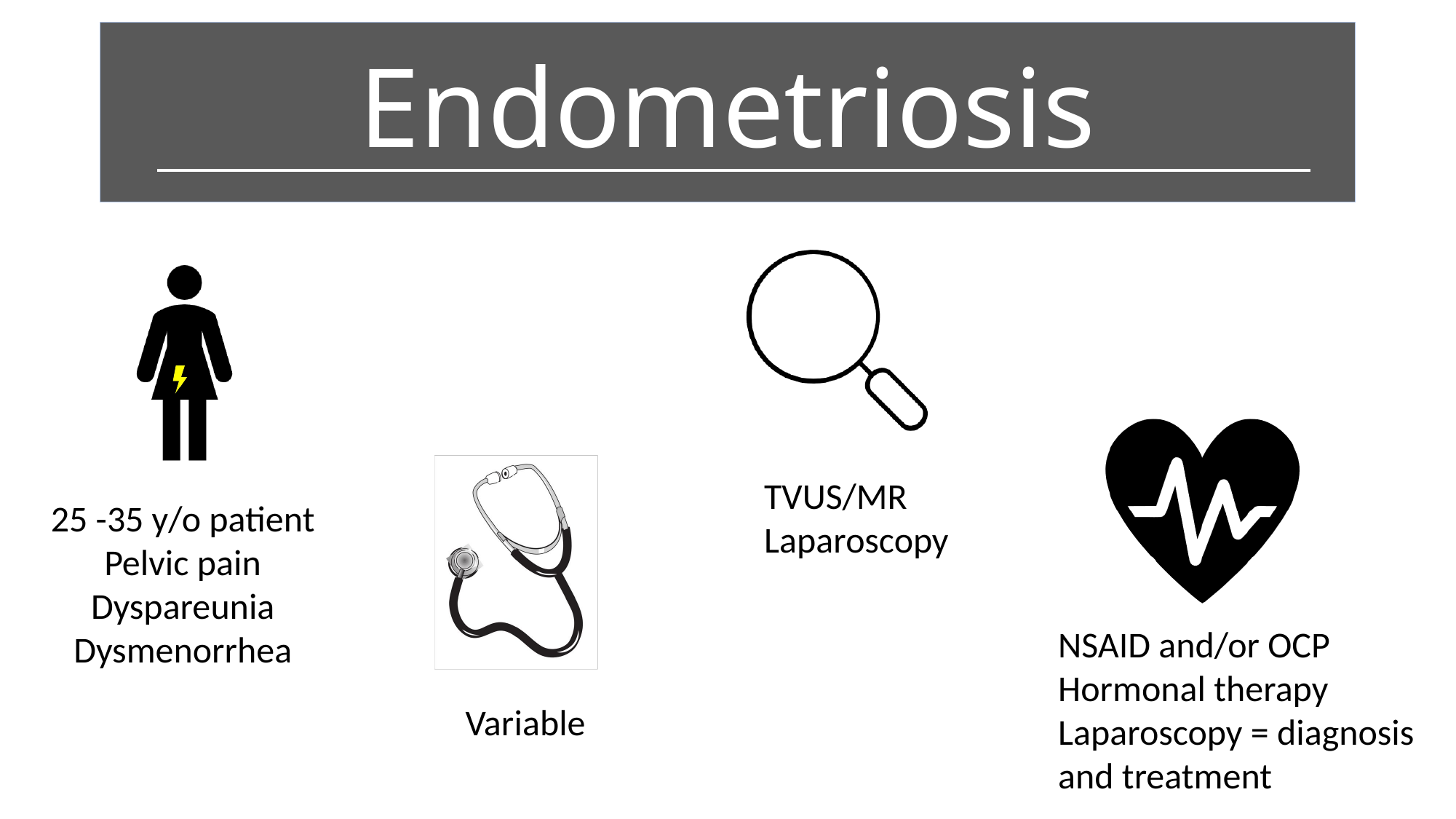

Endometriosis
#
TVUS/MR
Laparoscopy
25 -35 y/o patient
Pelvic pain
Dyspareunia
Dysmenorrhea
NSAID and/or OCP
Hormonal therapy
Laparoscopy = diagnosis
and treatment
Variable

## Slide 23
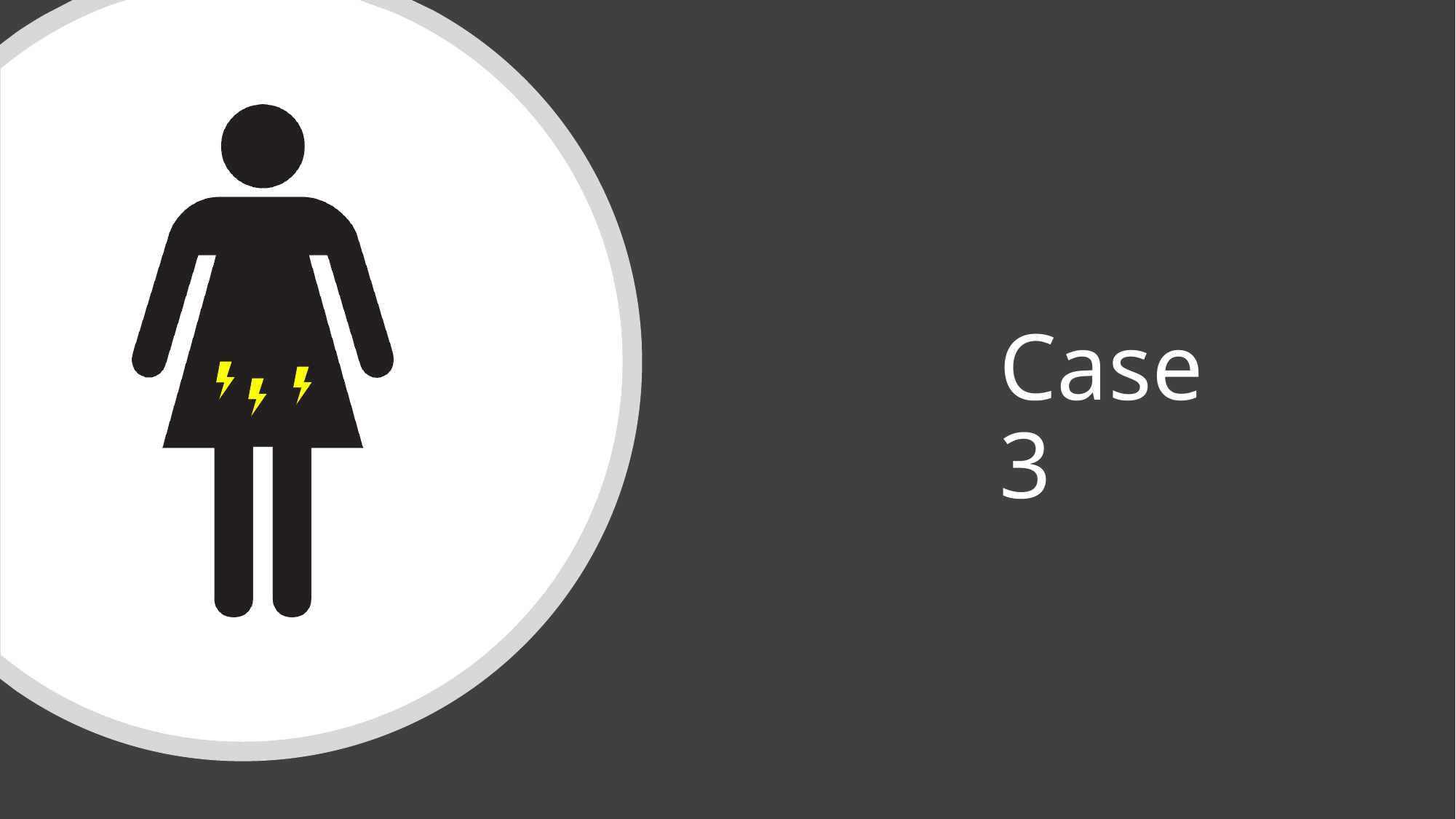

# Case 3

## Slide 24
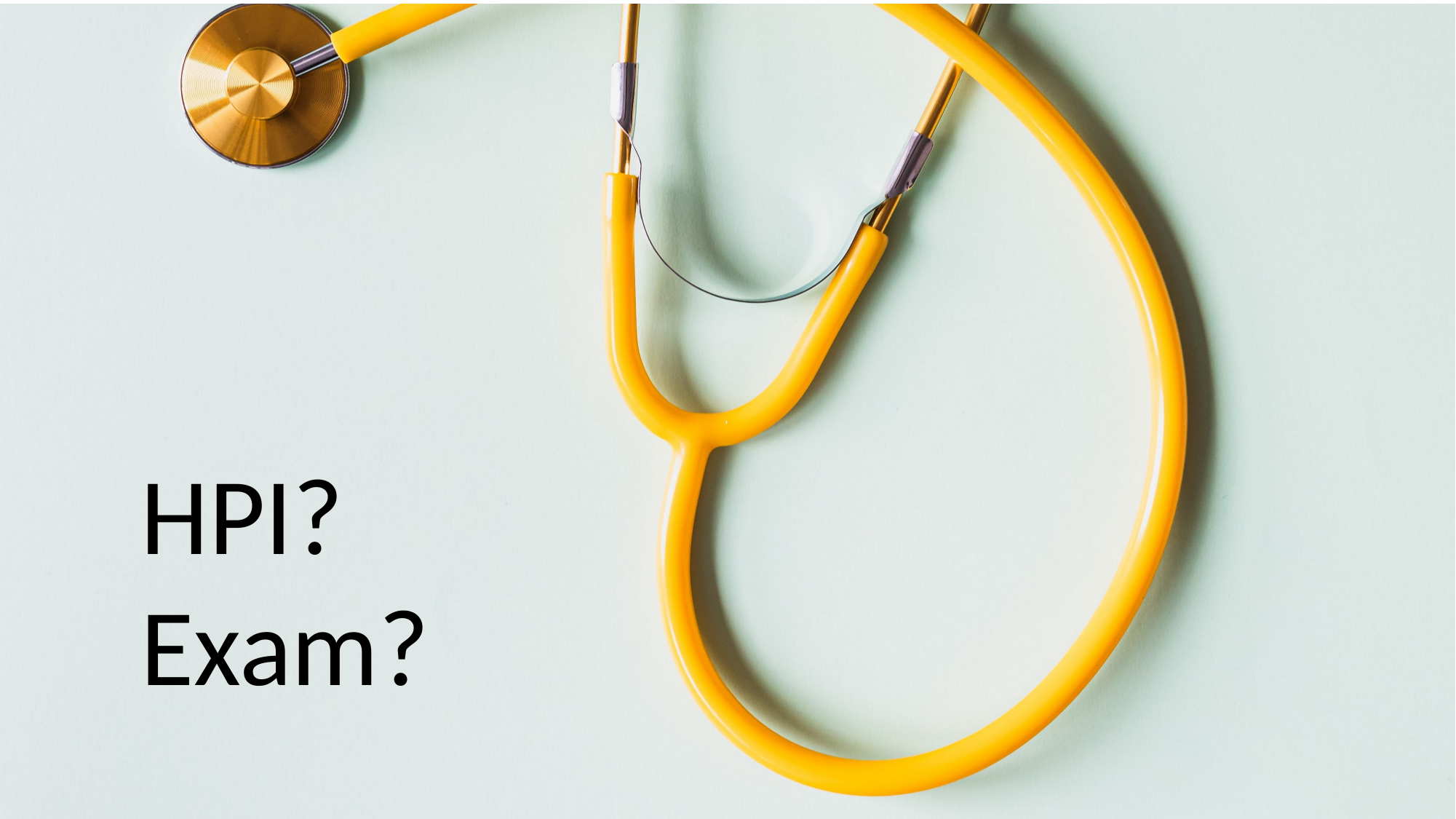

HPI?
Exam?

## Slide 25
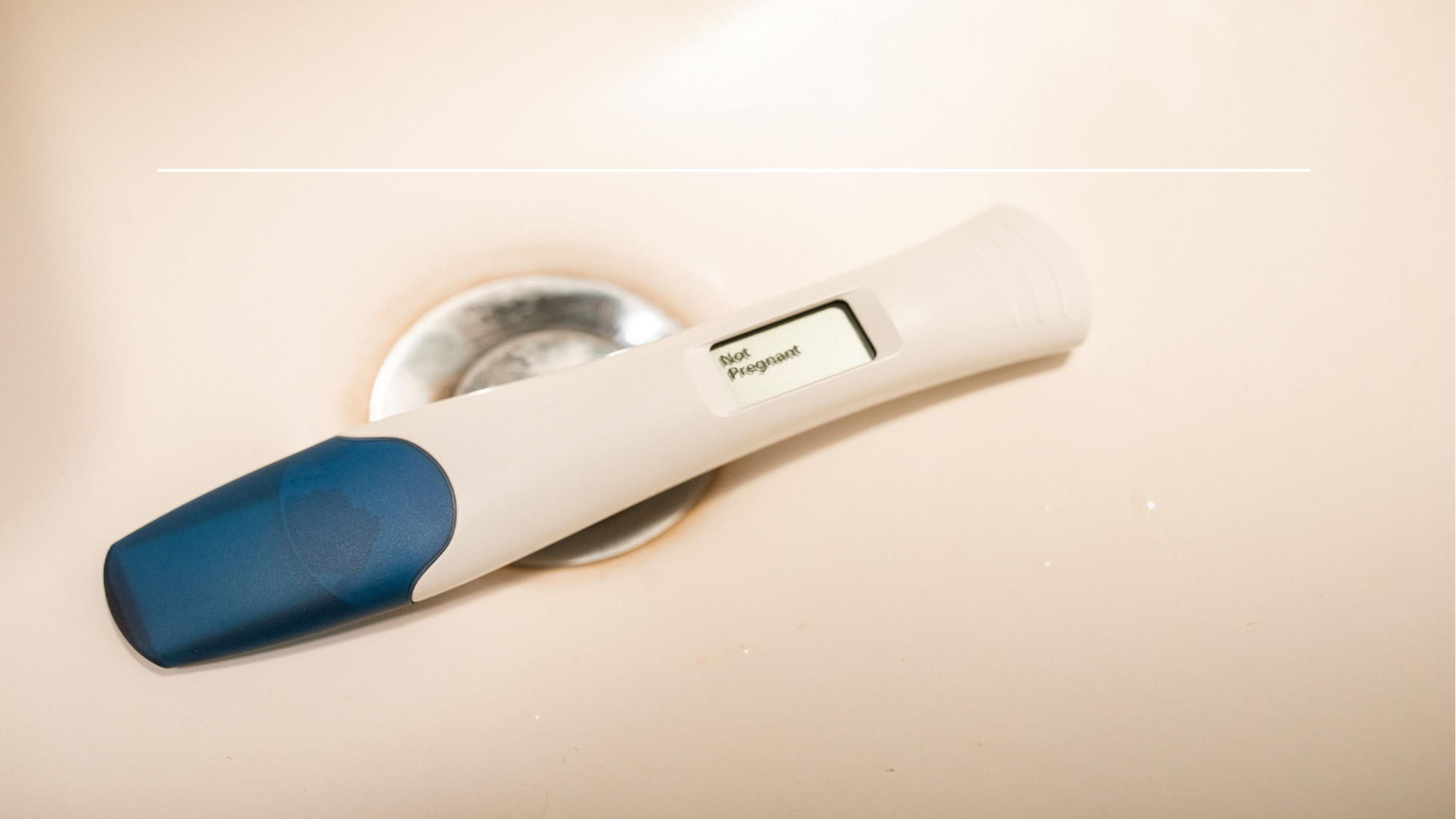

#

## Slide 26
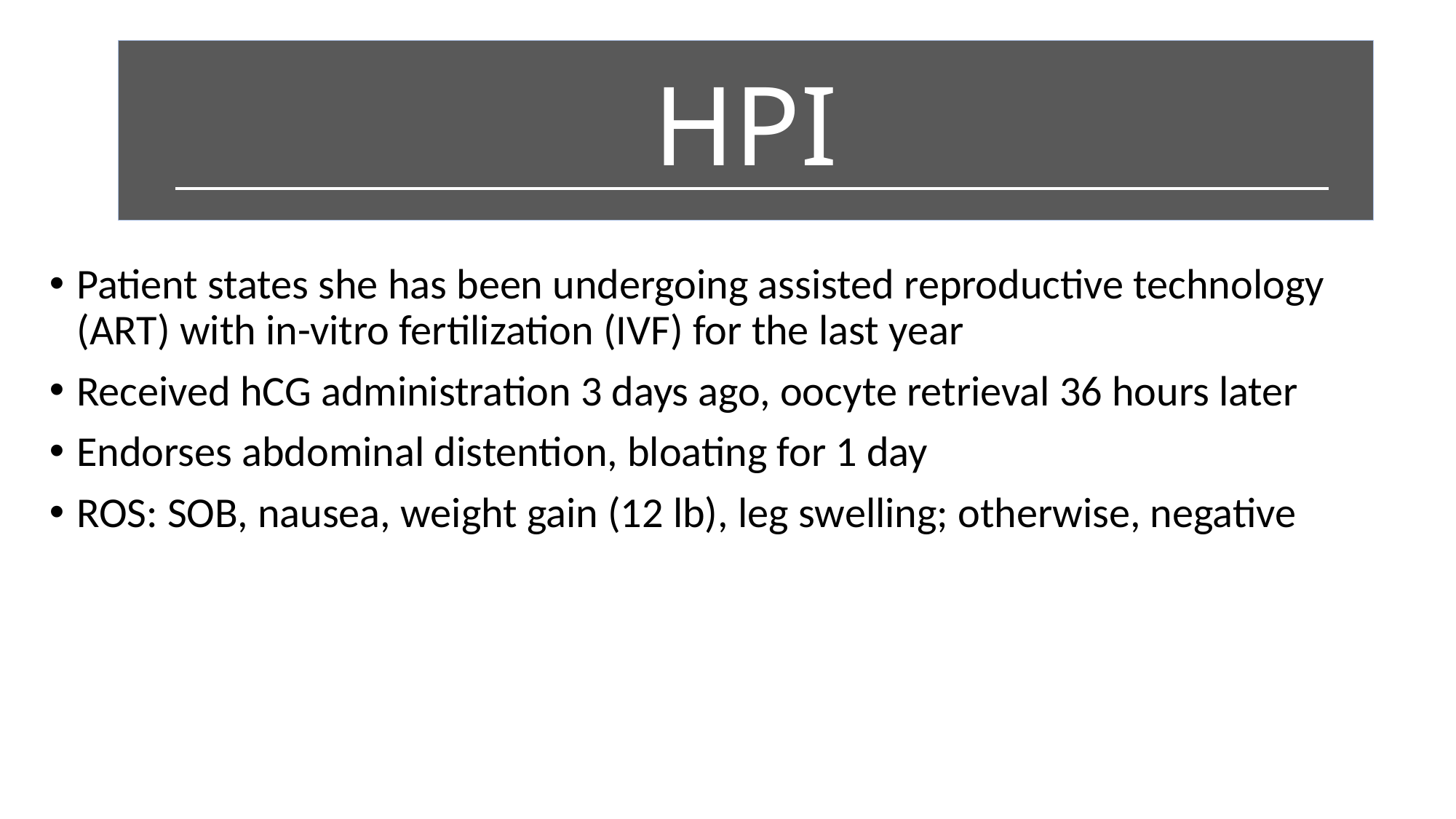

HPI
#
Patient states she has been undergoing assisted reproductive technology (ART) with in-vitro fertilization (IVF) for the last year
Received hCG administration 3 days ago, oocyte retrieval 36 hours later
Endorses abdominal distention, bloating for 1 day
ROS: SOB, nausea, weight gain (12 lb), leg swelling; otherwise, negative

## Slide 27
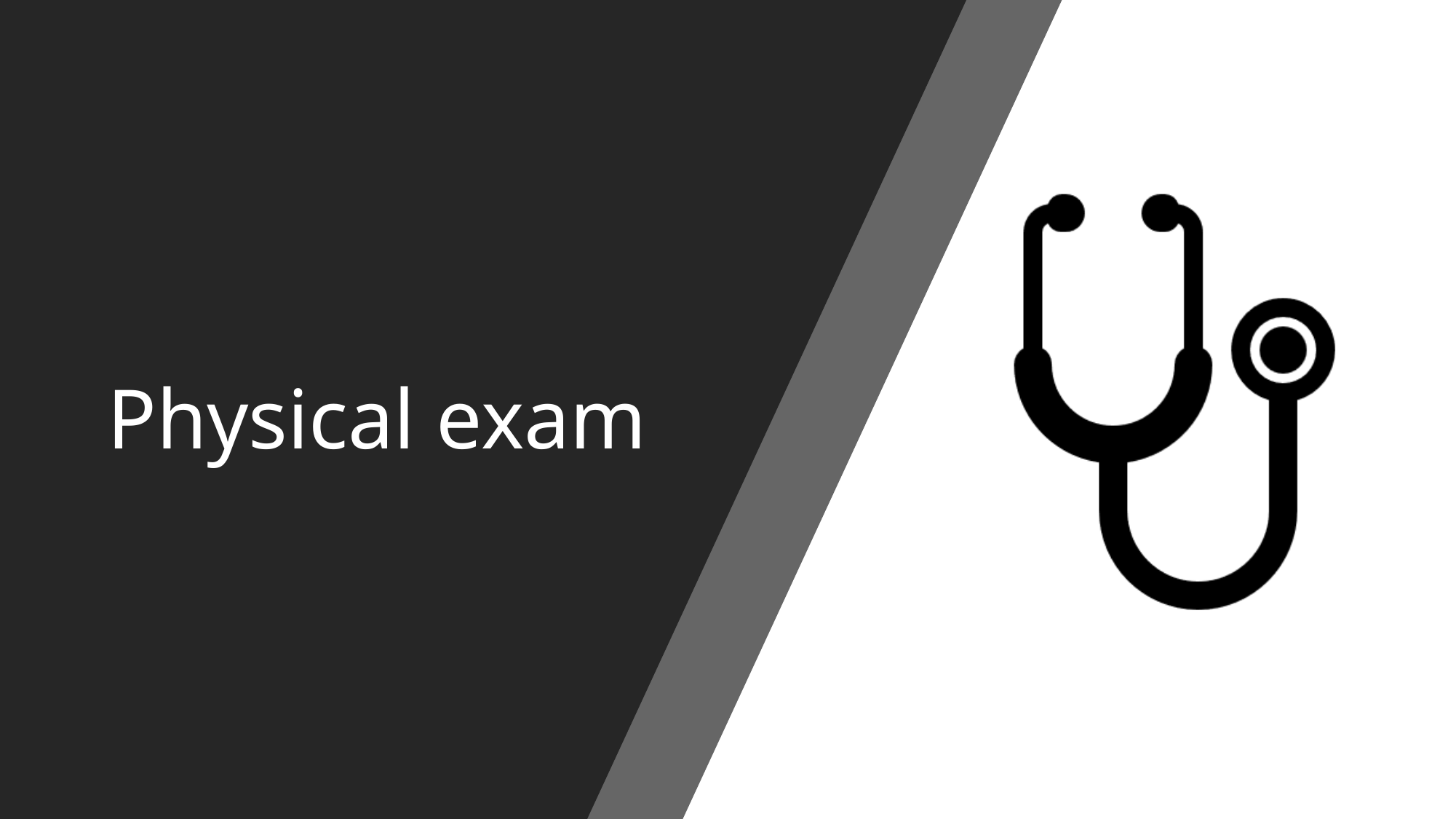

# Physical exam

## Slide 28
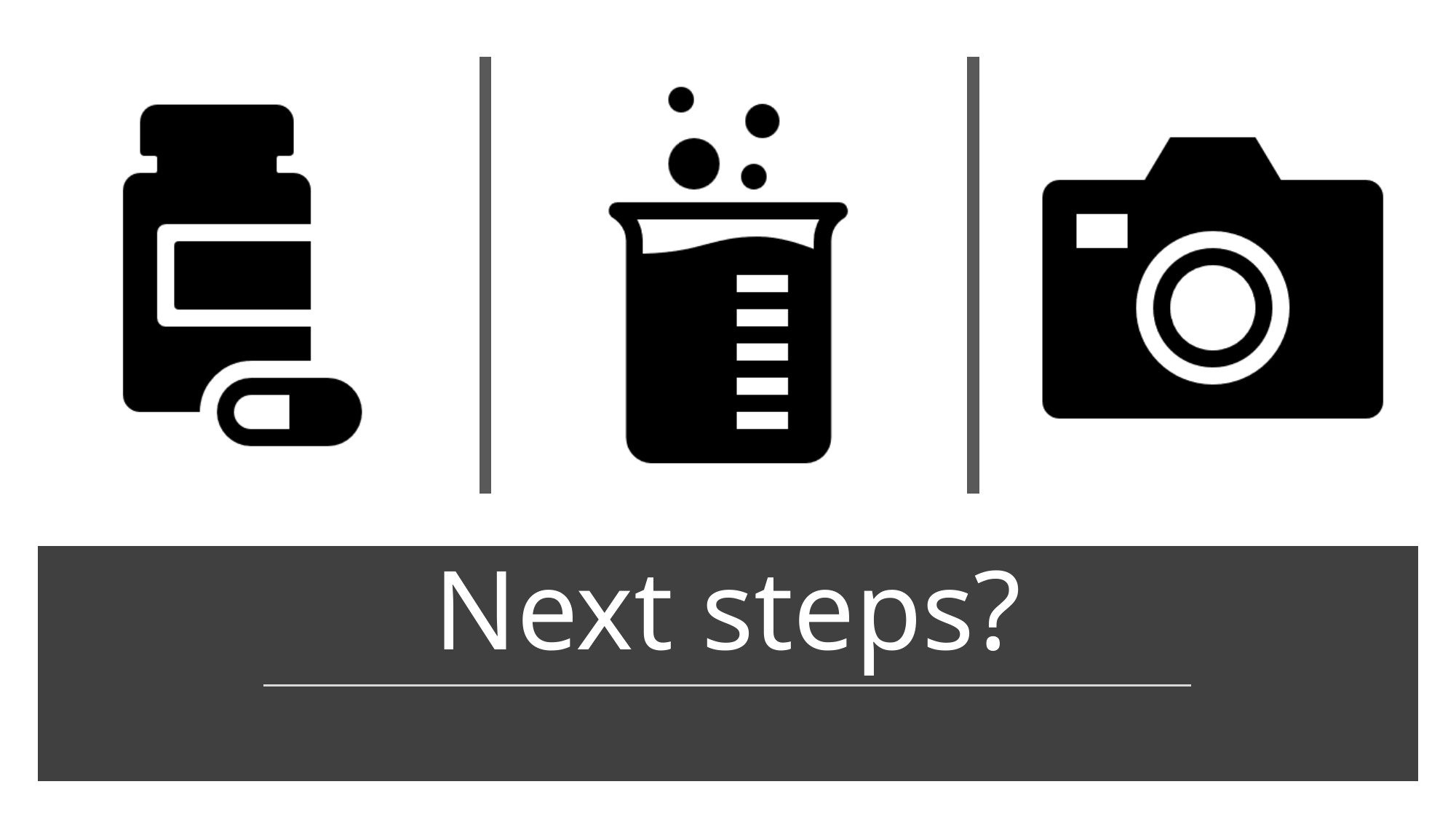

# Next steps?

## Slide 29
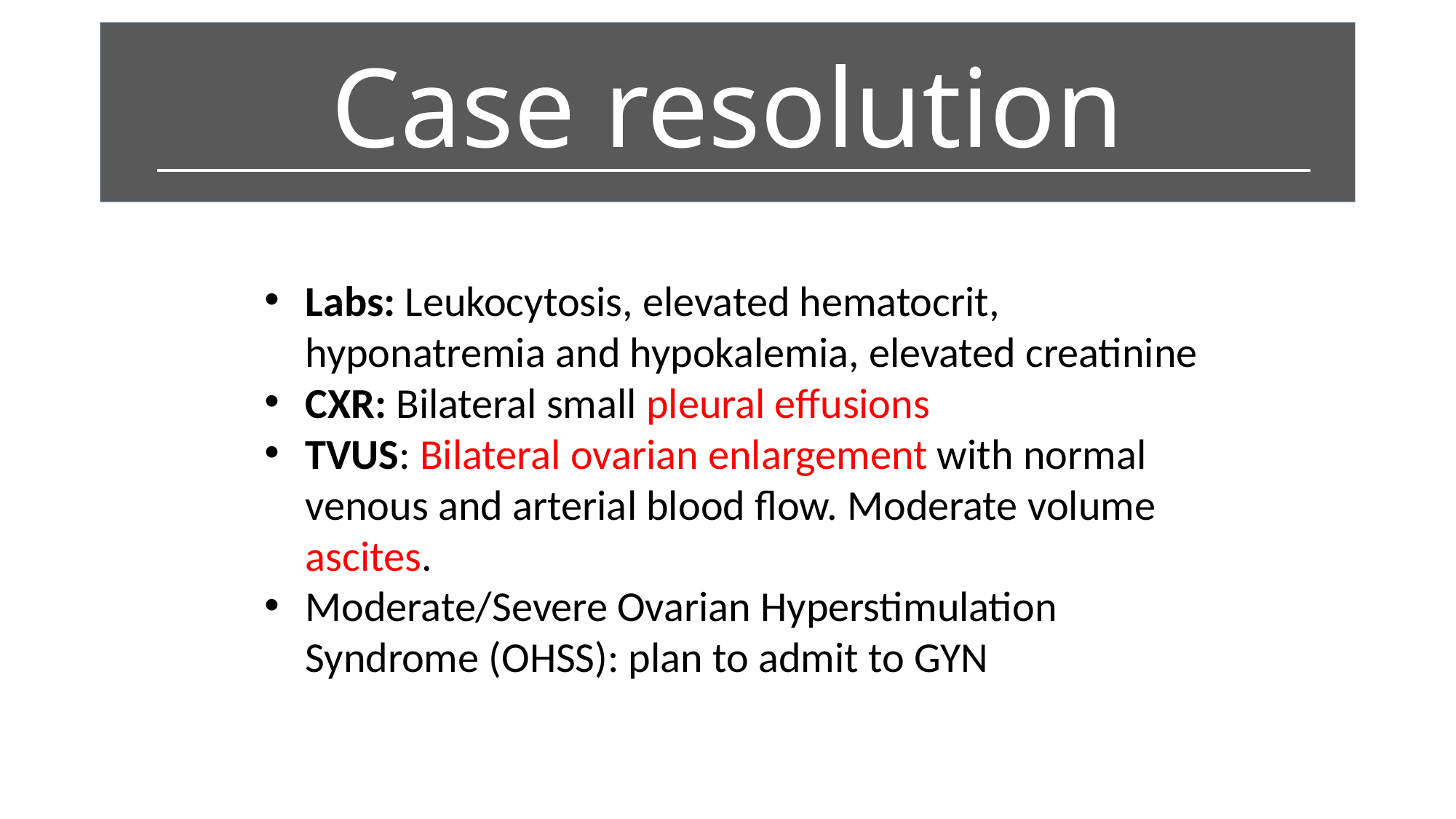

Case resolution
Labs: Leukocytosis, elevated hematocrit, hyponatremia and hypokalemia, elevated creatinine
CXR: Bilateral small pleural effusions
TVUS: Bilateral ovarian enlargement with normal venous and arterial blood flow. Moderate volume ascites.
Moderate/Severe Ovarian Hyperstimulation Syndrome (OHSS): plan to admit to GYN

## Slide 30
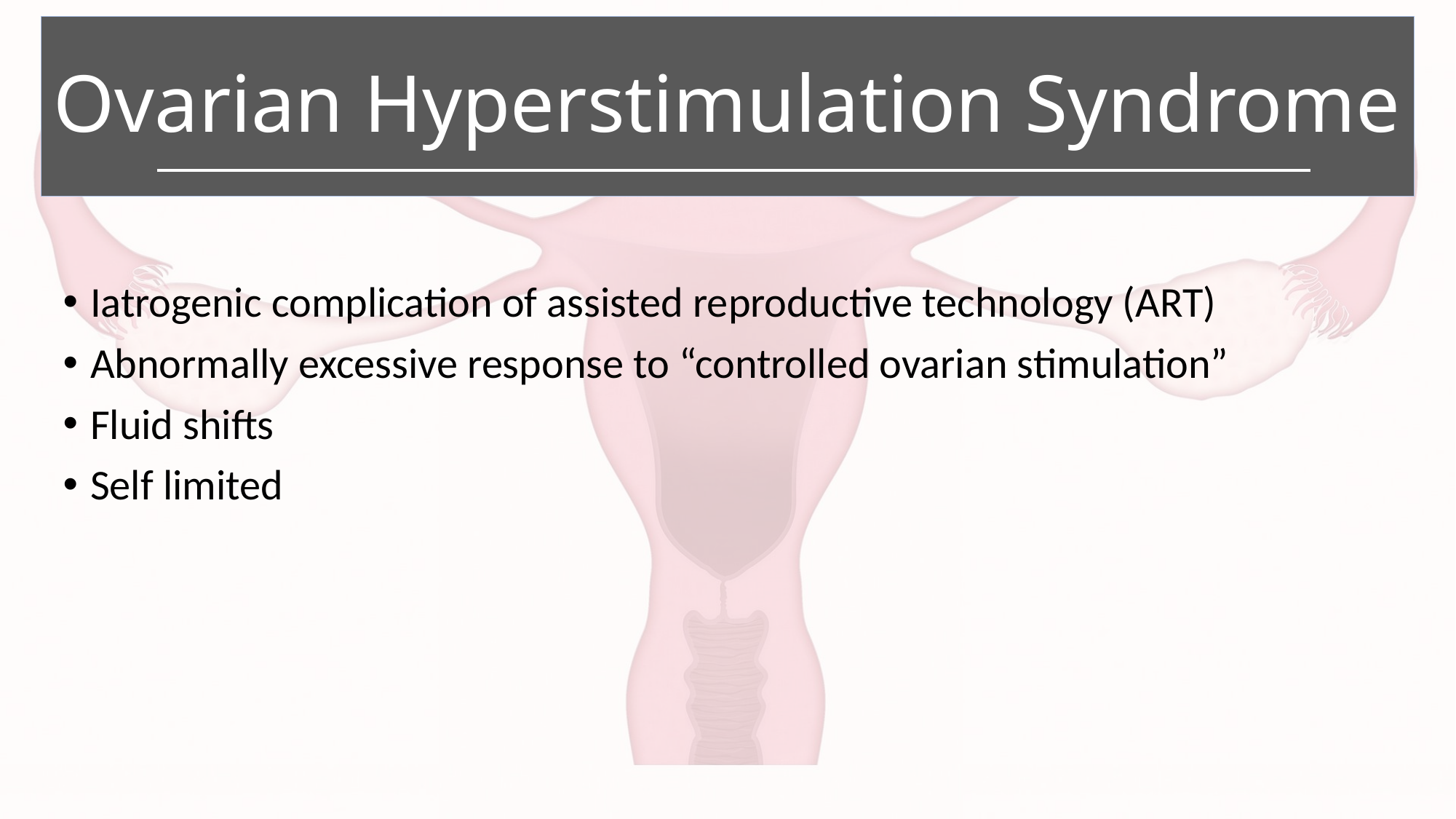

Ovarian Hyperstimulation Syndrome
Iatrogenic complication of assisted reproductive technology (ART)
Abnormally excessive response to “controlled ovarian stimulation”
Fluid shifts
Self limited

## Slide 31
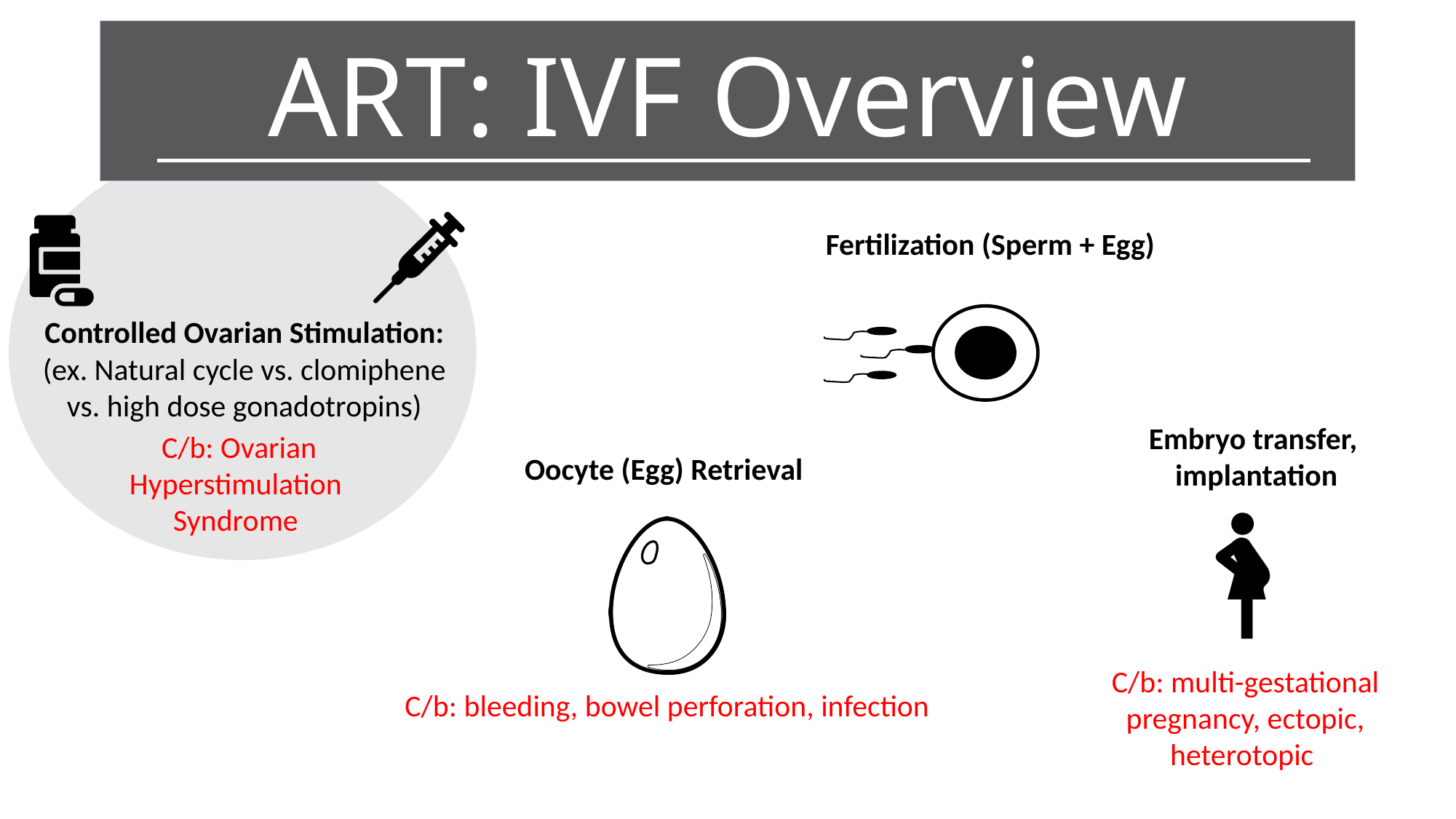

ART: IVF Overview
#
Fertilization (Sperm + Egg)
Controlled Ovarian Stimulation:
(ex. Natural cycle vs. clomiphene vs. high dose gonadotropins)
Embryo transfer,
 implantation
 C/b: Ovarian Hyperstimulation Syndrome
Oocyte (Egg) Retrieval
C/b: multi-gestational pregnancy, ectopic, heterotopic
C/b: bleeding, bowel perforation, infection

## Slide 32
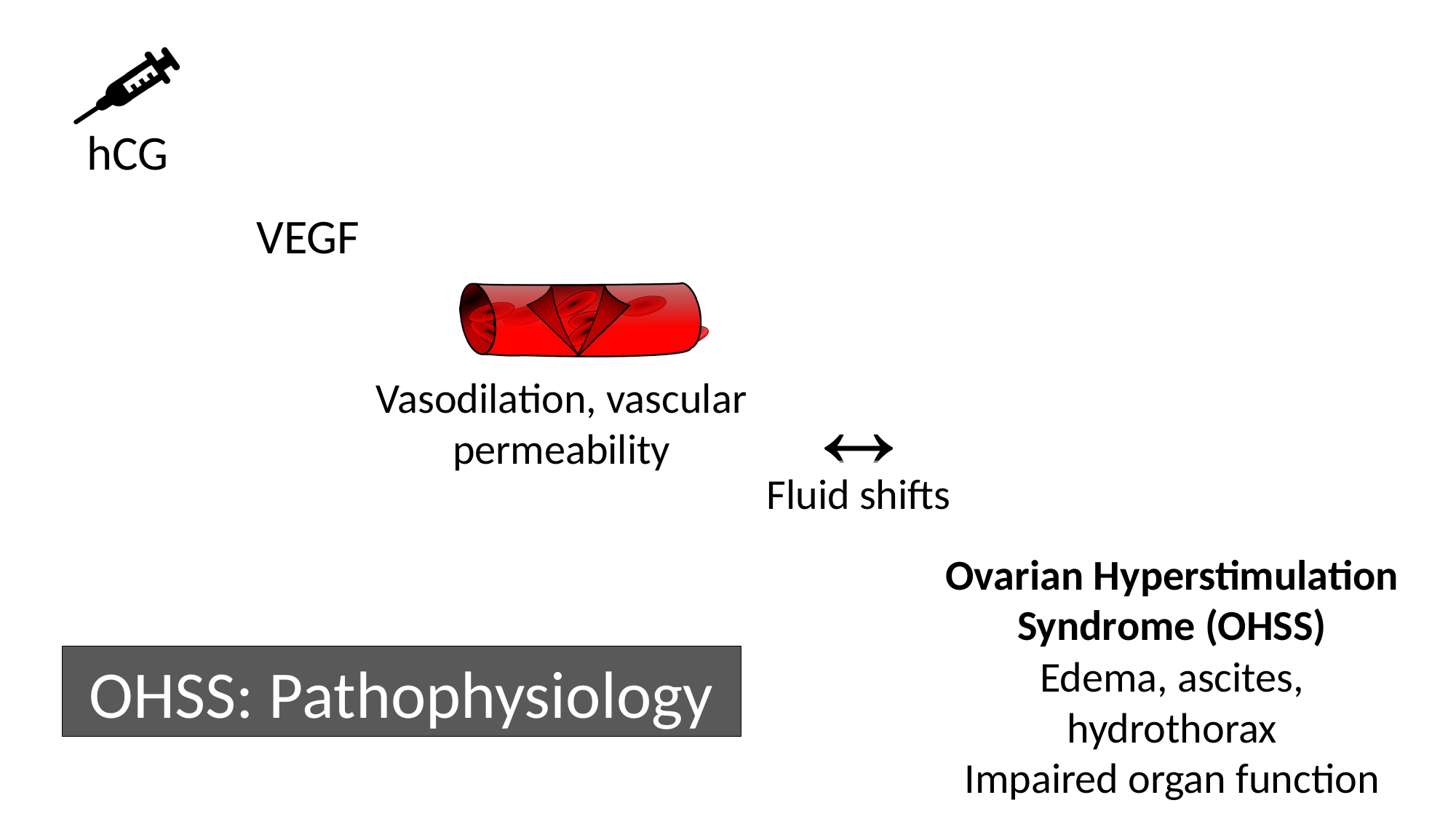

hCG
Vasodilation, vascular permeability
Fluid shifts
Ovarian Hyperstimulation Syndrome (OHSS)
Edema, ascites, hydrothorax
Impaired organ function
OHSS: Pathophysiology

## Slide 33
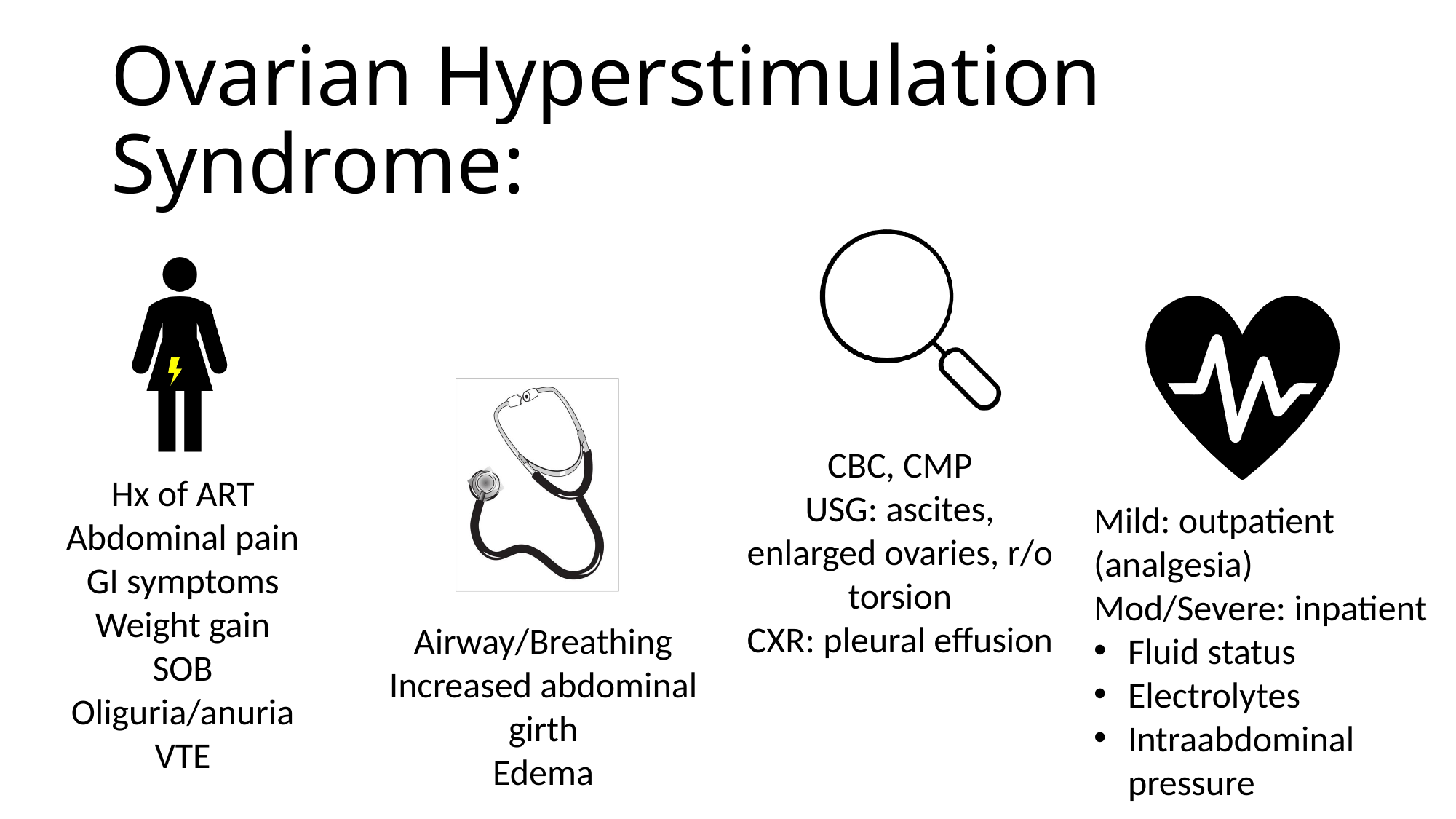

# Ovarian Hyperstimulation Syndrome:
CBC, CMP
USG: ascites, enlarged ovaries, r/o torsion
CXR: pleural effusion
Hx of ART
Abdominal pain
GI symptoms
Weight gain
SOB
Oliguria/anuria
VTE
Mild: outpatient (analgesia)
Mod/Severe: inpatient
Fluid status
Electrolytes
Intraabdominal pressure
Airway/Breathing
Increased abdominal girth
Edema

## Slide 34
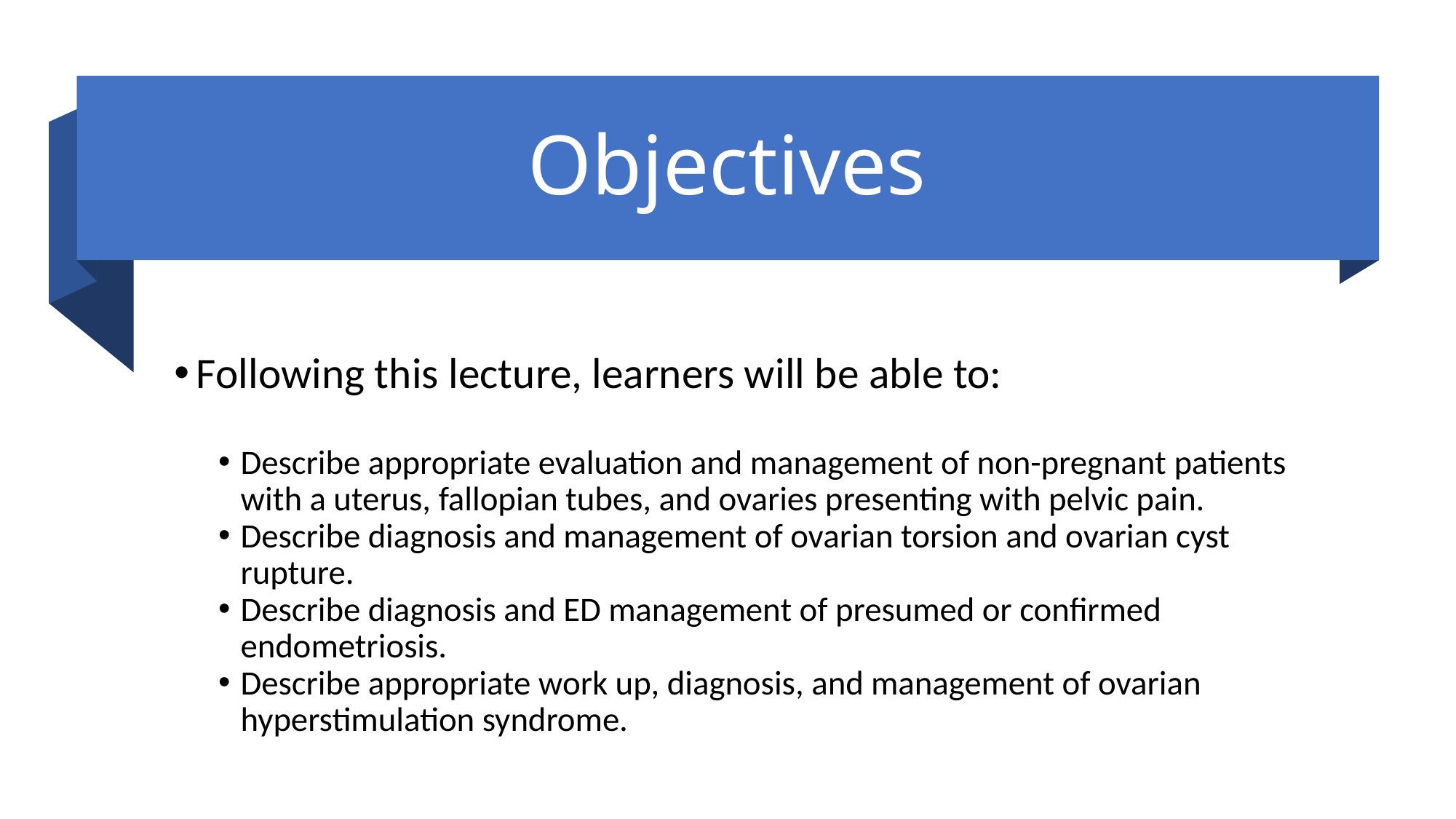

# Objectives
Following this lecture, learners will be able to:
Describe appropriate evaluation and management of non-pregnant patients with a uterus, fallopian tubes, and ovaries presenting with pelvic pain.
Describe diagnosis and management of ovarian torsion and ovarian cyst rupture.
Describe diagnosis and ED management of presumed or confirmed endometriosis.
Describe appropriate work up, diagnosis, and management of ovarian hyperstimulation syndrome.

## Slide 35
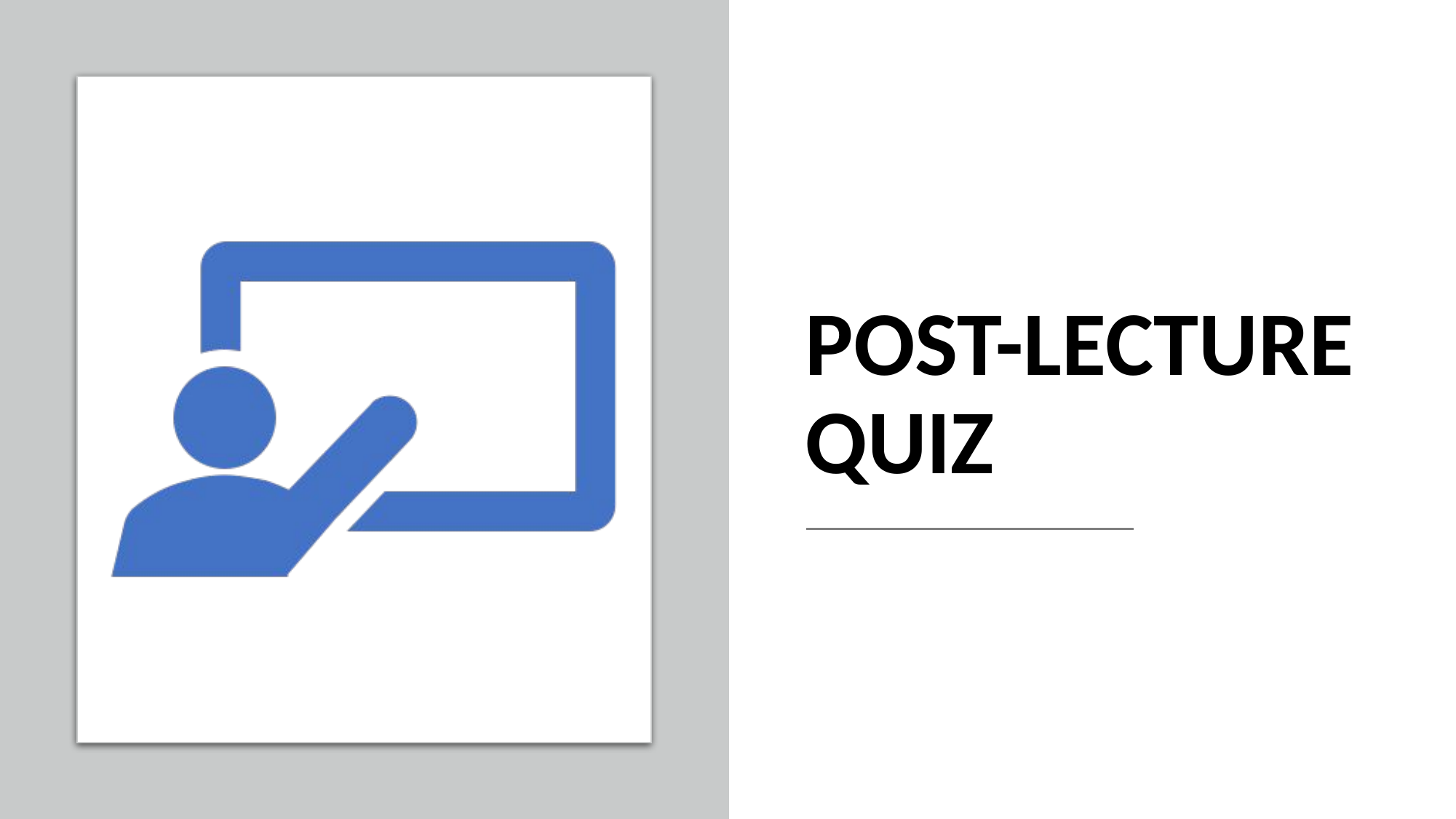

# POST-LECTURE QUIZ

## Slide 36
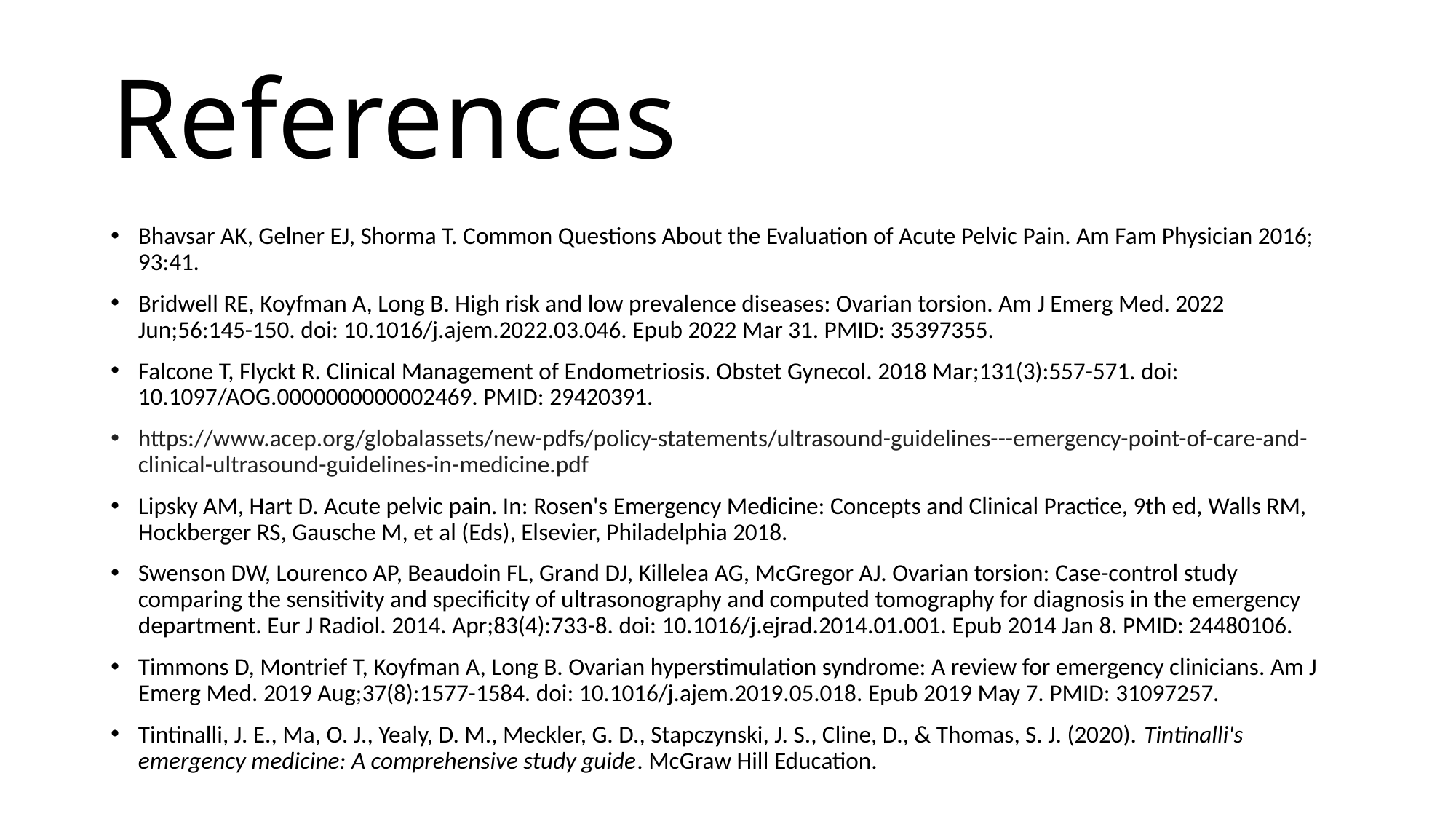

# References
Bhavsar AK, Gelner EJ, Shorma T. Common Questions About the Evaluation of Acute Pelvic Pain. Am Fam Physician 2016; 93:41.
Bridwell RE, Koyfman A, Long B. High risk and low prevalence diseases: Ovarian torsion. Am J Emerg Med. 2022 Jun;56:145-150. doi: 10.1016/j.ajem.2022.03.046. Epub 2022 Mar 31. PMID: 35397355.
Falcone T, Flyckt R. Clinical Management of Endometriosis. Obstet Gynecol. 2018 Mar;131(3):557-571. doi: 10.1097/AOG.0000000000002469. PMID: 29420391.
https://www.acep.org/globalassets/new-pdfs/policy-statements/ultrasound-guidelines---emergency-point-of-care-and-clinical-ultrasound-guidelines-in-medicine.pdf
Lipsky AM, Hart D. Acute pelvic pain. In: Rosen's Emergency Medicine: Concepts and Clinical Practice, 9th ed, Walls RM, Hockberger RS, Gausche M, et al (Eds), Elsevier, Philadelphia 2018.
Swenson DW, Lourenco AP, Beaudoin FL, Grand DJ, Killelea AG, McGregor AJ. Ovarian torsion: Case-control study comparing the sensitivity and specificity of ultrasonography and computed tomography for diagnosis in the emergency department. Eur J Radiol. 2014. Apr;83(4):733-8. doi: 10.1016/j.ejrad.2014.01.001. Epub 2014 Jan 8. PMID: 24480106.
Timmons D, Montrief T, Koyfman A, Long B. Ovarian hyperstimulation syndrome: A review for emergency clinicians. Am J Emerg Med. 2019 Aug;37(8):1577-1584. doi: 10.1016/j.ajem.2019.05.018. Epub 2019 May 7. PMID: 31097257.
Tintinalli, J. E., Ma, O. J., Yealy, D. M., Meckler, G. D., Stapczynski, J. S., Cline, D., & Thomas, S. J. (2020). Tintinalli's emergency medicine: A comprehensive study guide. McGraw Hill Education.
