# Supplementary material for: Emergency Medicine Obstetrics and Gynecology: A Case-Based Curriculum for Residents
Source: MedEdPORTAL. 2023 Aug 11;19:11330. doi: 10.15766/mep_2374-8265.11330 (PMC10415535; doi:10.15766/mep_2374-8265.11330)
Supplement: Supplementary file 1 — Ectopic Pregnancy and Emergencies in the First 20 Weeks.pptxPregnancy Emergencies After 20 Weeks.pptxDelivery Emergencies.pptxPelvic Pain in the Nonpregnant Patient.pptxVaginitis, Cervicitis, and PID.pptxAbnormal Uterine Bleeding.pptxLabor and Perimortem C-Section.pptxSession Review Questions.docxPrecurriculum Survey.docxPostcurriculum Survey.docx [file mep_2374-8265.11330-s001.zip › E. Vaginitis, Cervicitis, and PID.pptx]

## Slide 1
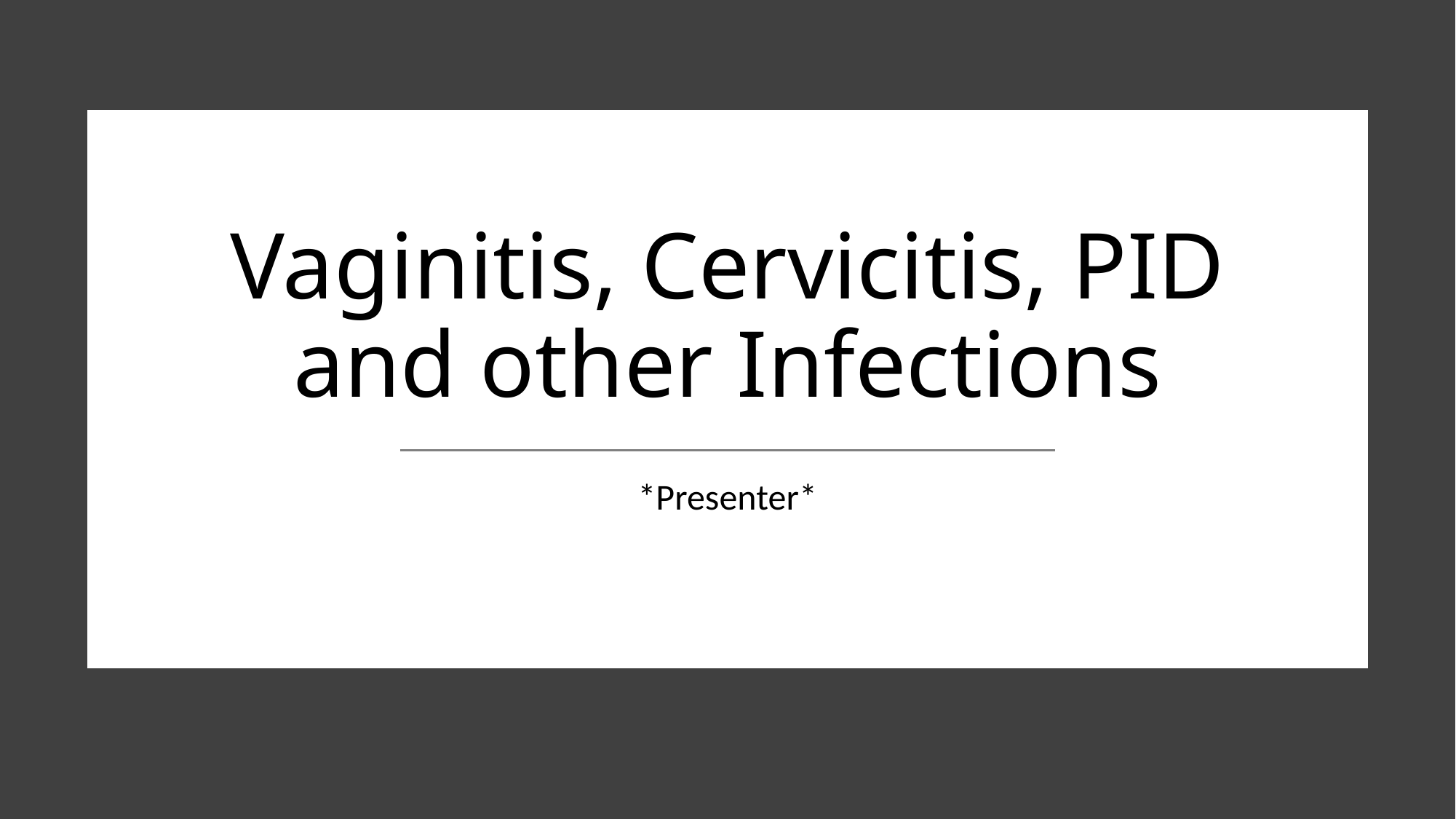

# Vaginitis, Cervicitis, PID and other Infections
*Presenter*

## Slide 2
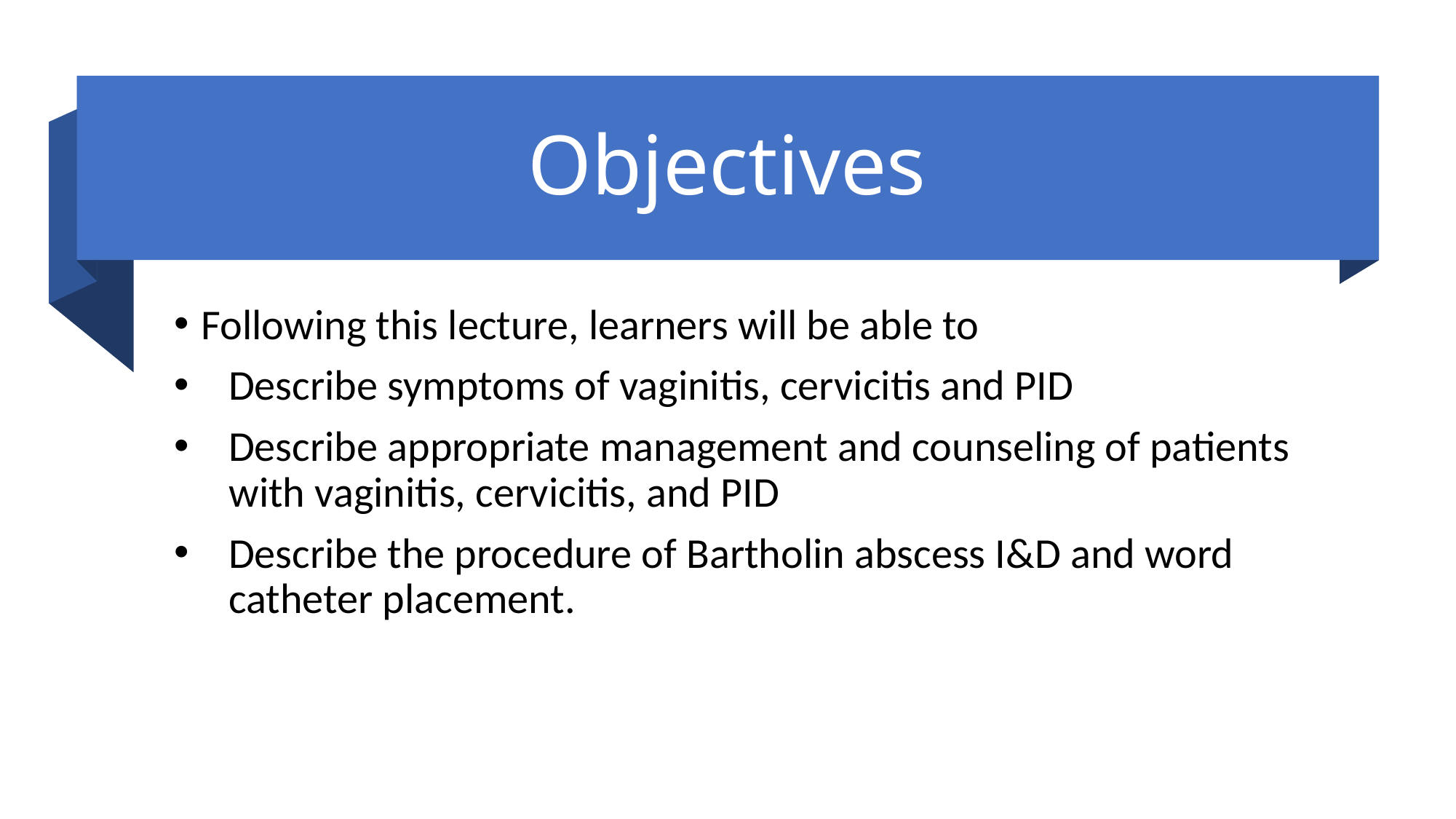

# Objectives
Following this lecture, learners will be able to
Describe symptoms of vaginitis, cervicitis and PID
Describe appropriate management and counseling of patients with vaginitis, cervicitis, and PID
Describe the procedure of Bartholin abscess I&D and word catheter placement.

## Slide 3
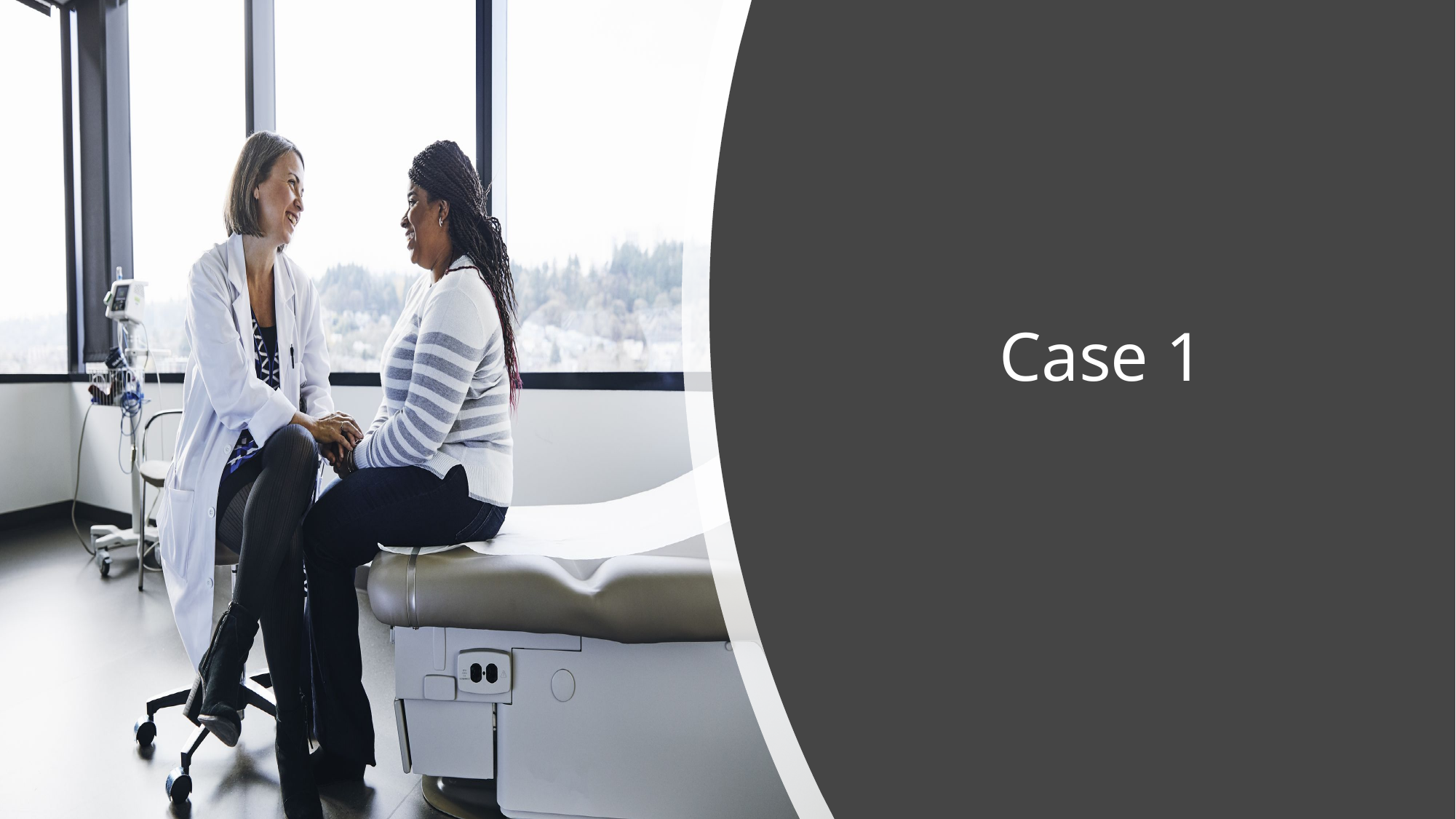

# Case 1

## Slide 4
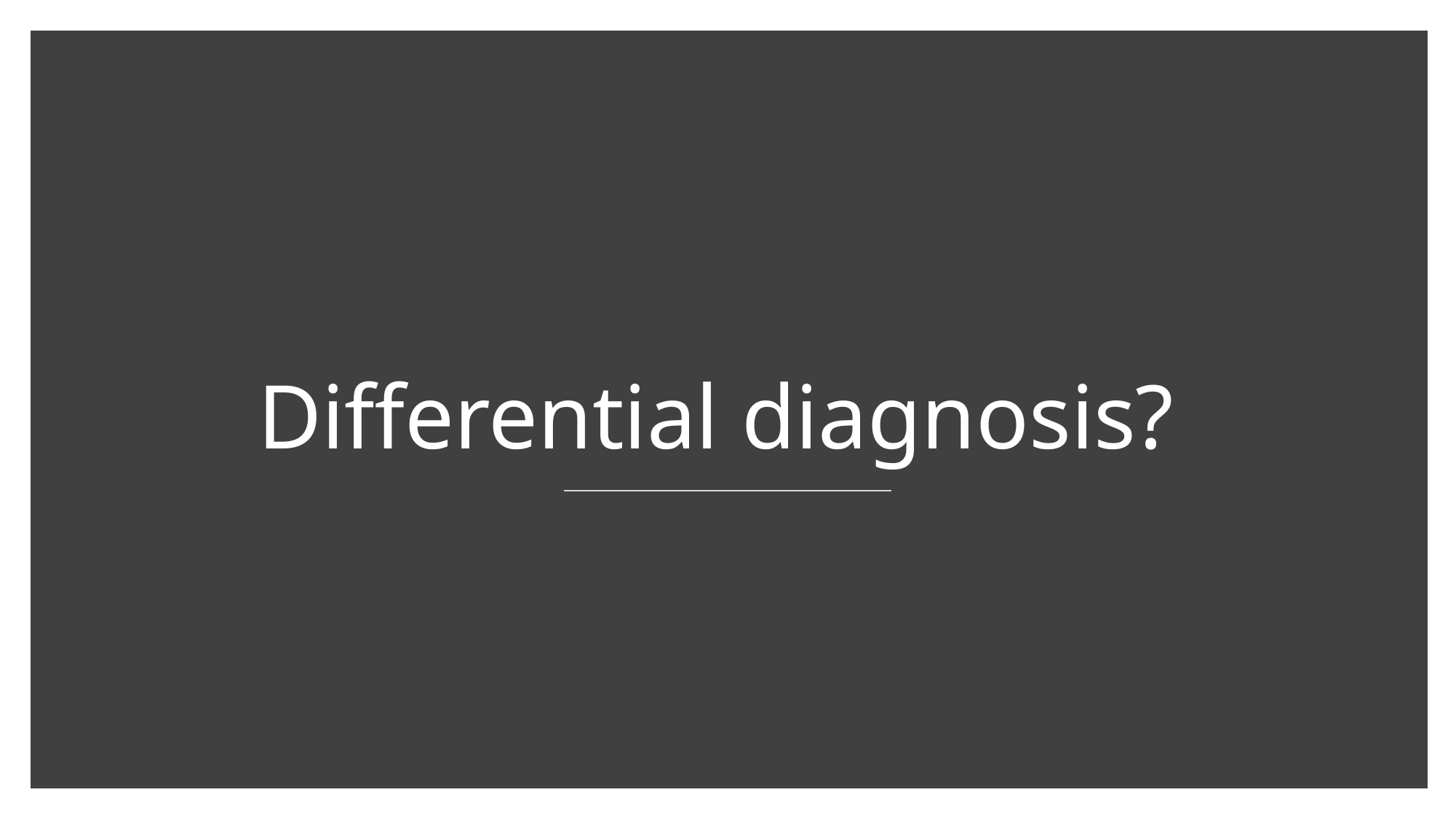

# Differential diagnosis?

## Slide 5
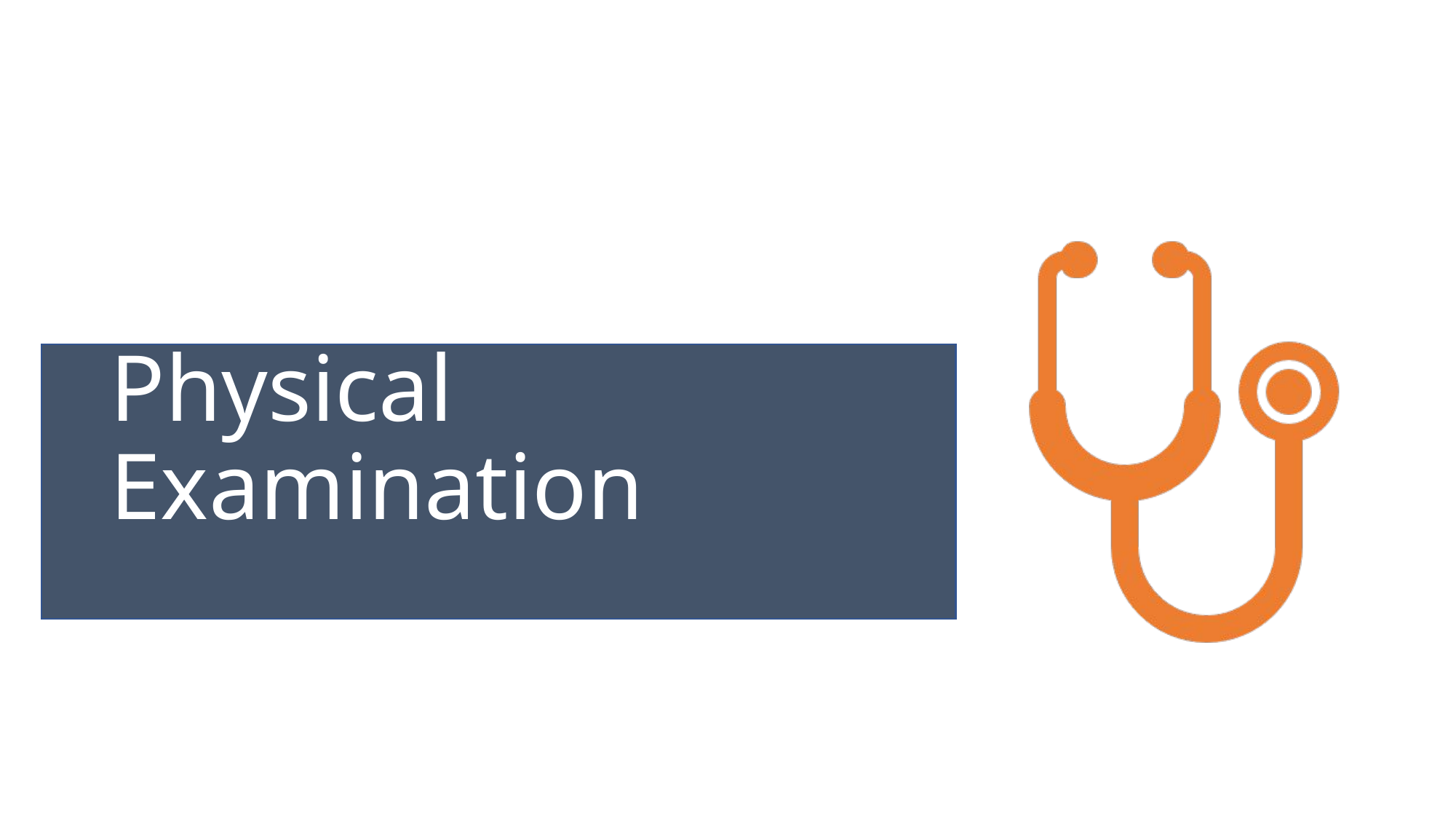

# Physical Examination

## Slide 6
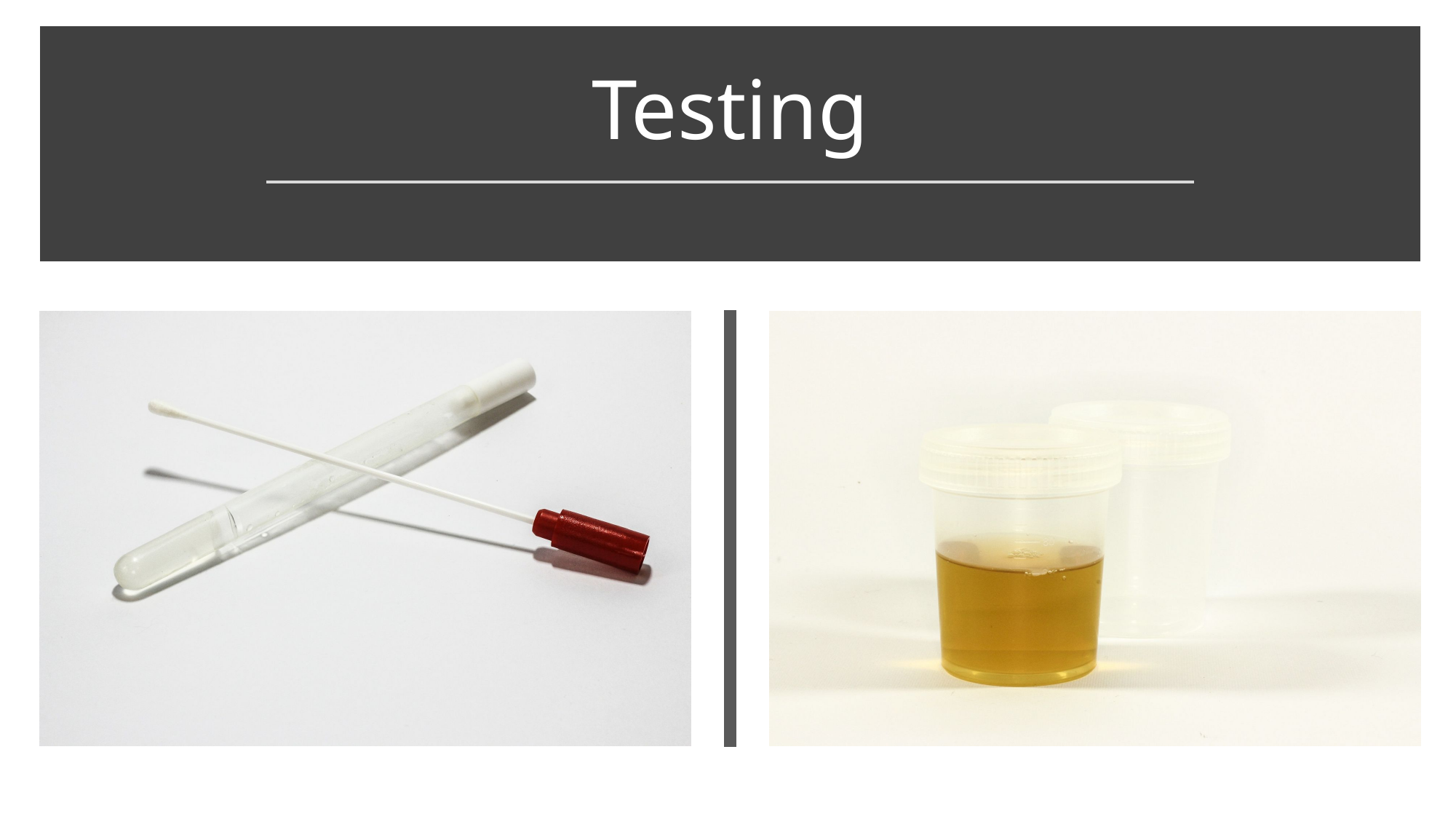

# Testing

## Slide 7
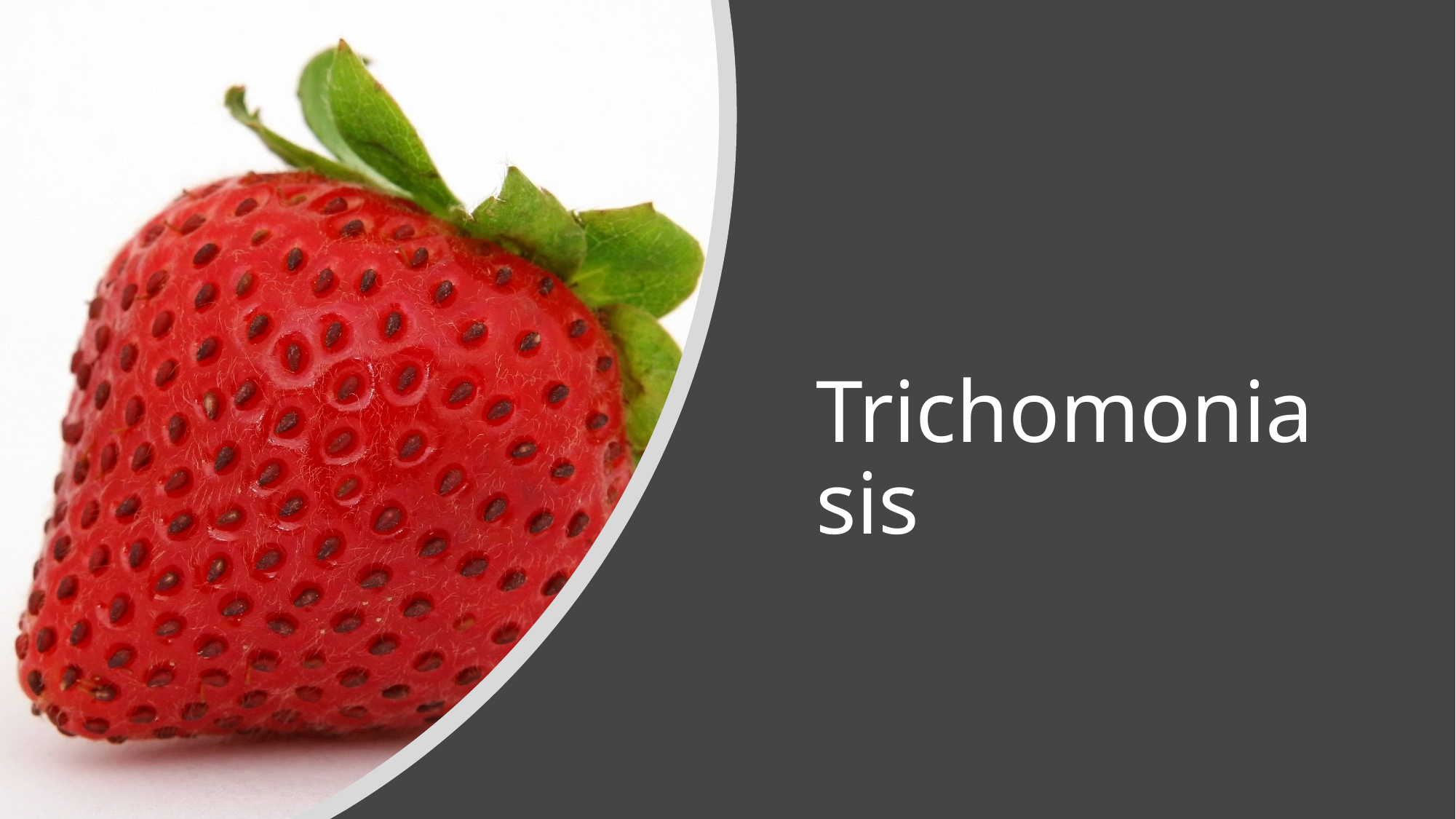

# Trichomoniasis

## Slide 8
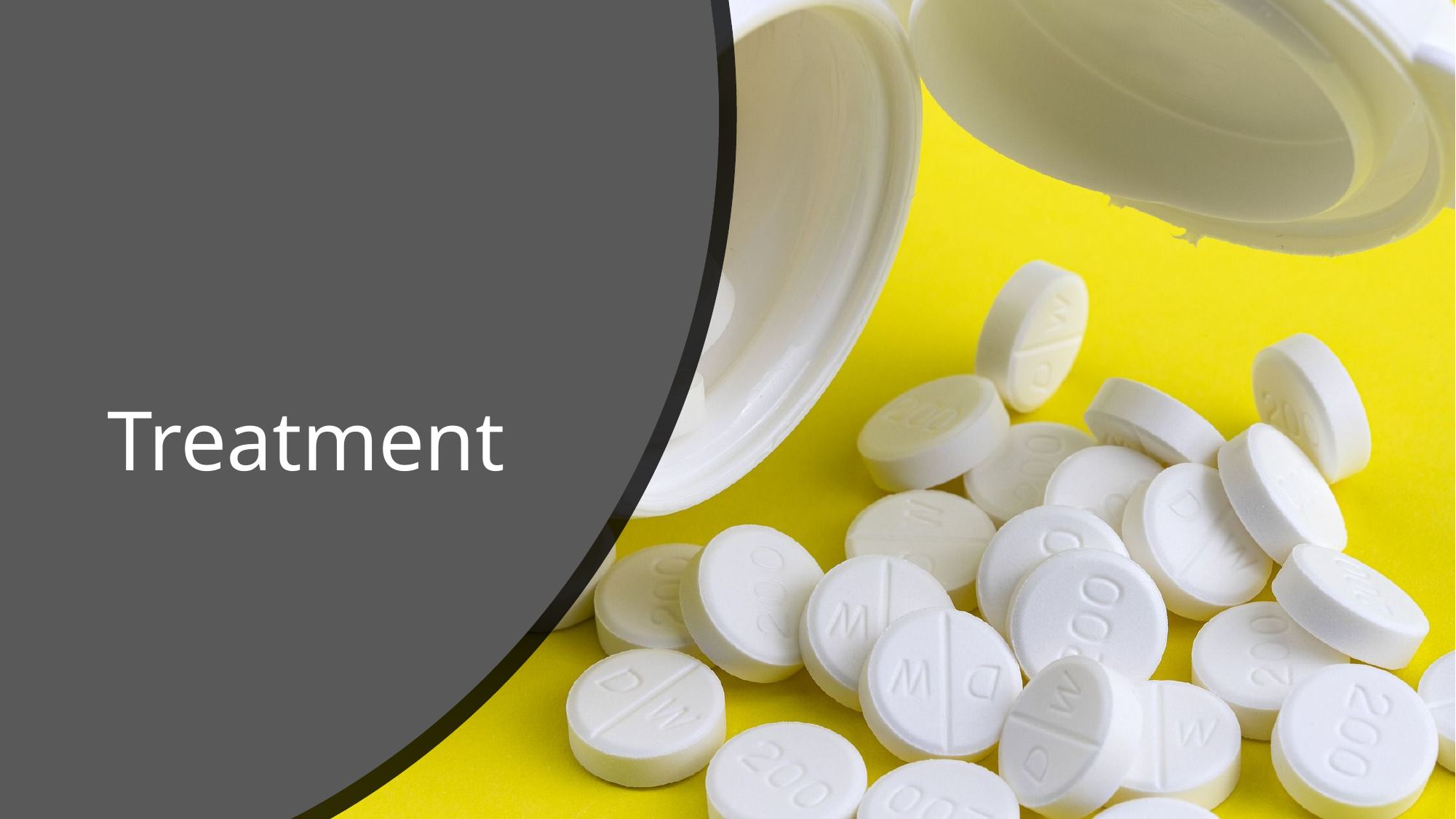

# Treatment

## Slide 9
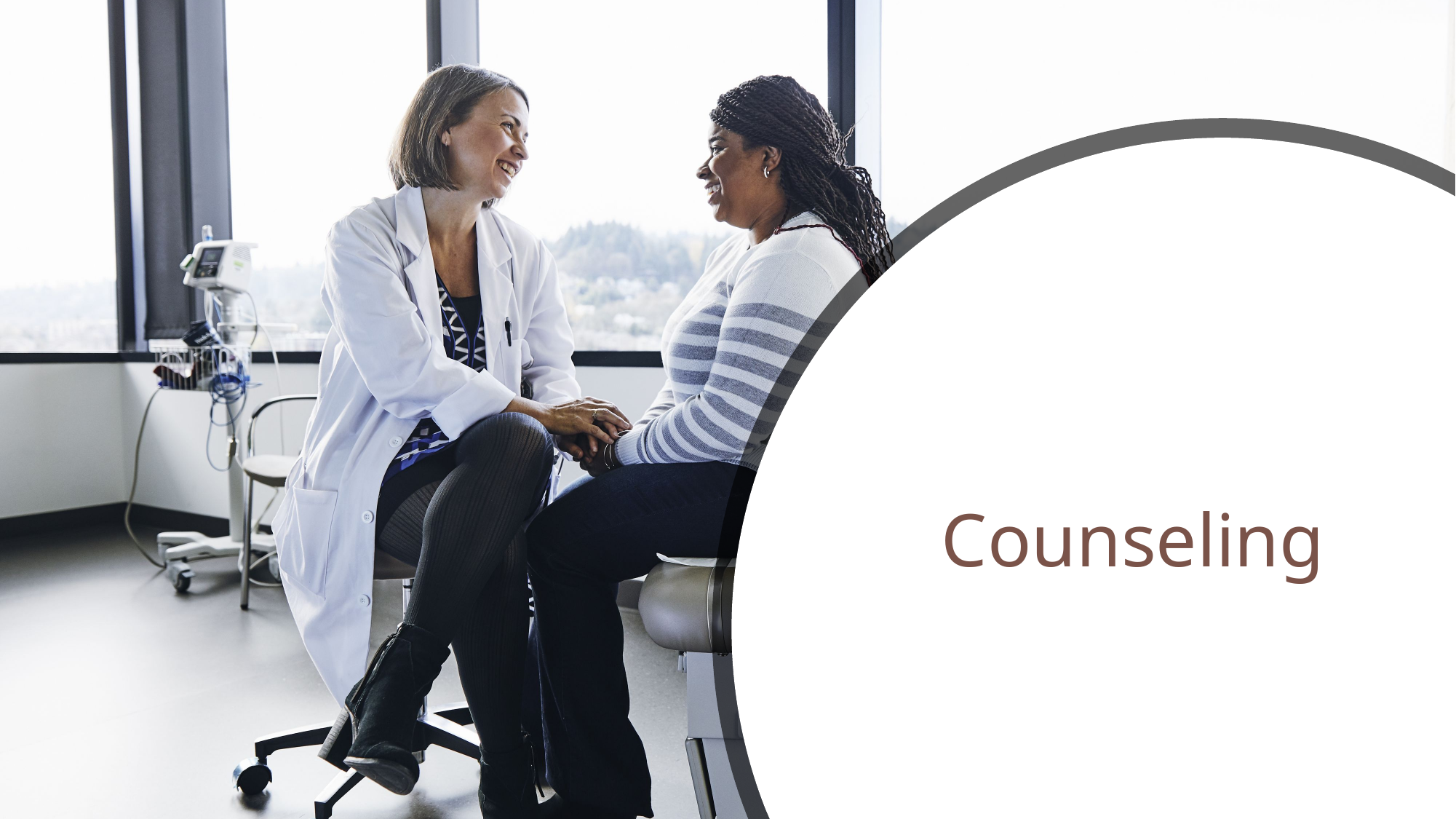

# Counseling

## Slide 10
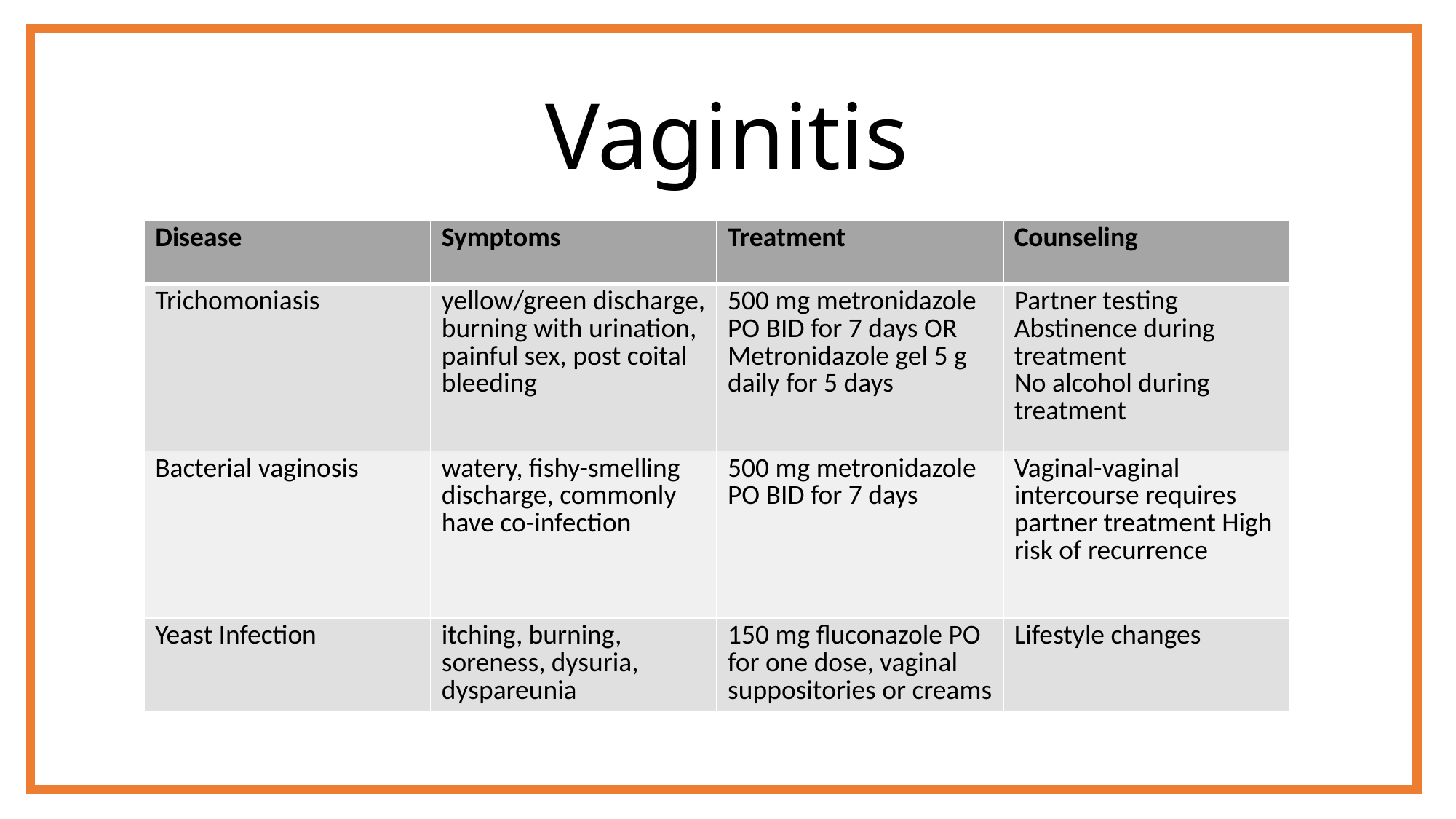

# Vaginitis
| Disease | Symptoms | Treatment | Counseling |
| --- | --- | --- | --- |
| Trichomoniasis | yellow/green discharge, burning with urination, painful sex, post coital bleeding | 500 mg metronidazole PO BID for 7 days OR Metronidazole gel 5 g daily for 5 days | Partner testing Abstinence during treatment No alcohol during treatment |
| Bacterial vaginosis | watery, fishy-smelling discharge, commonly have co-infection | 500 mg metronidazole PO BID for 7 days | Vaginal-vaginal intercourse requires partner treatment High risk of recurrence |
| Yeast Infection | itching, burning, soreness, dysuria, dyspareunia | 150 mg fluconazole PO for one dose, vaginal suppositories or creams | Lifestyle changes |

## Slide 11
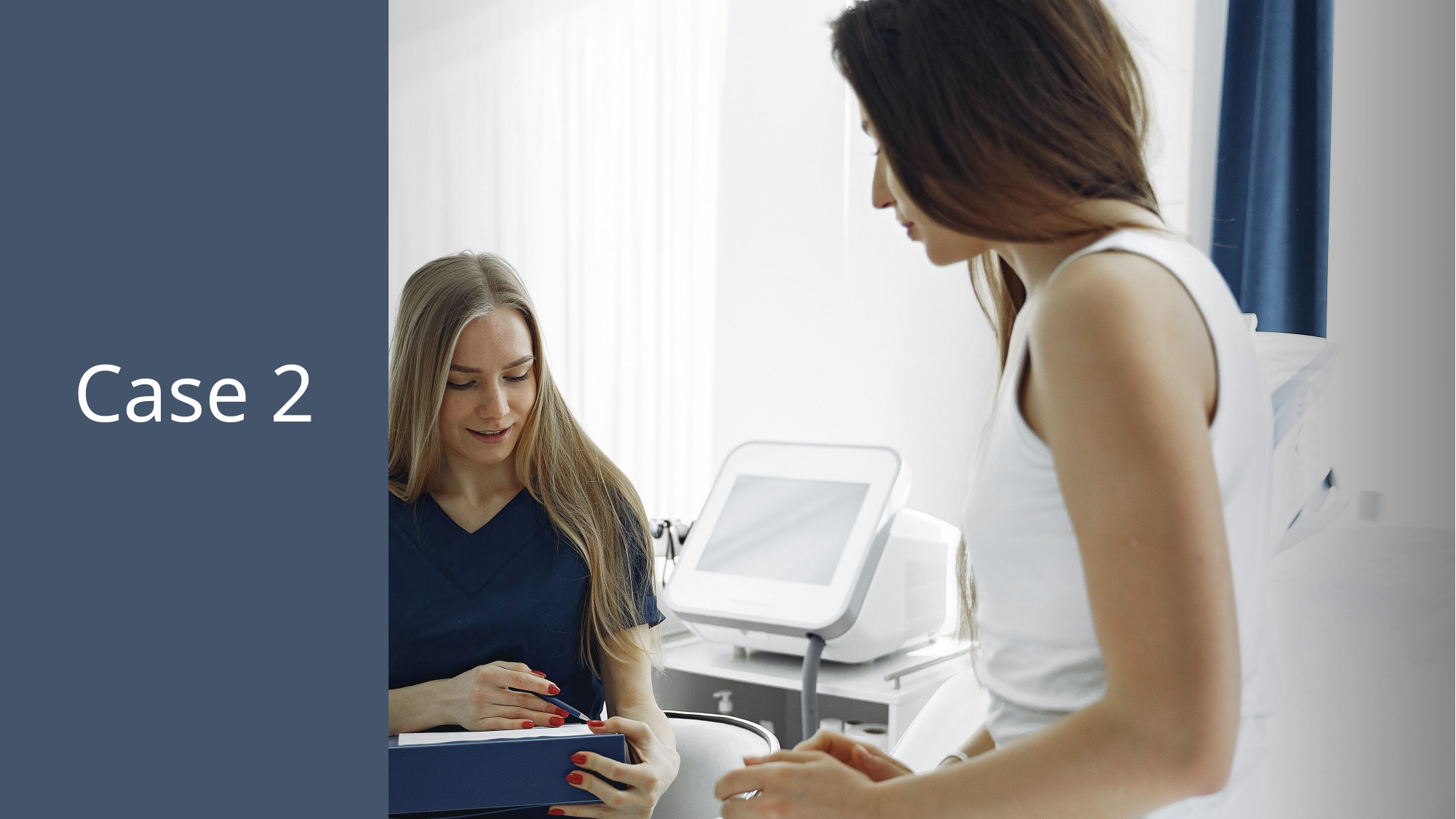

# Case 2

## Slide 12
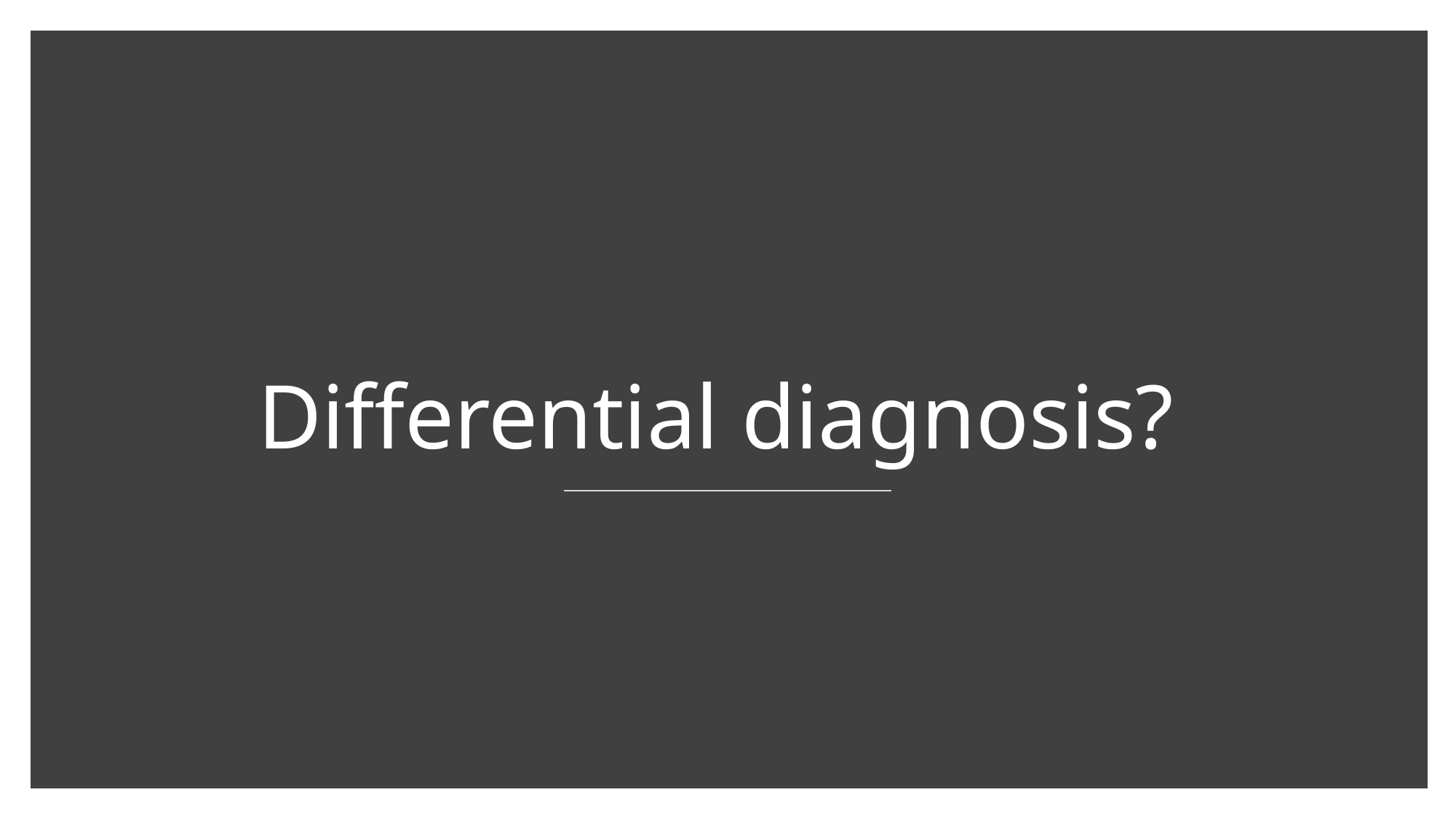

# Differential diagnosis?

## Slide 13
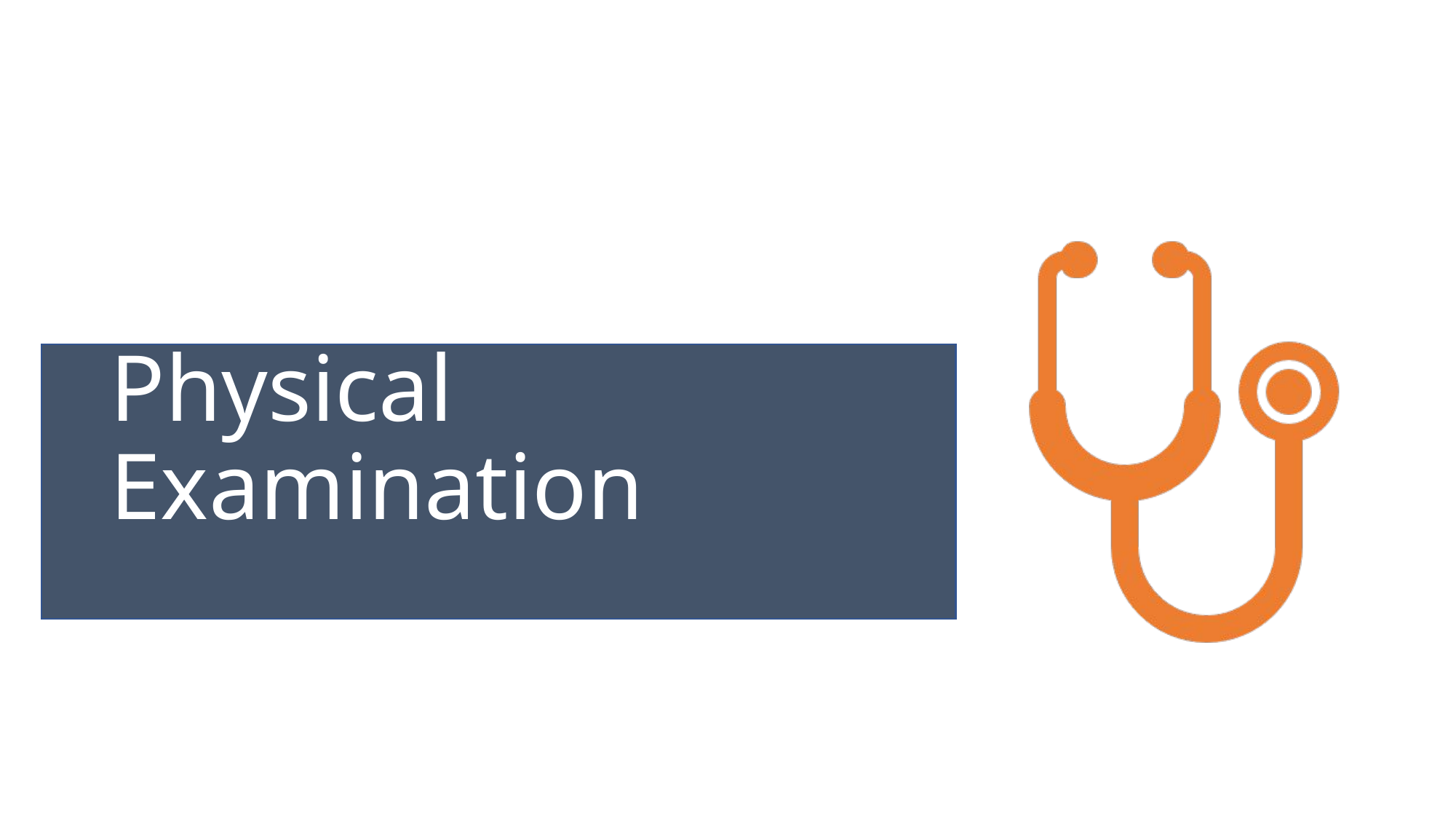

# Physical Examination

## Slide 14
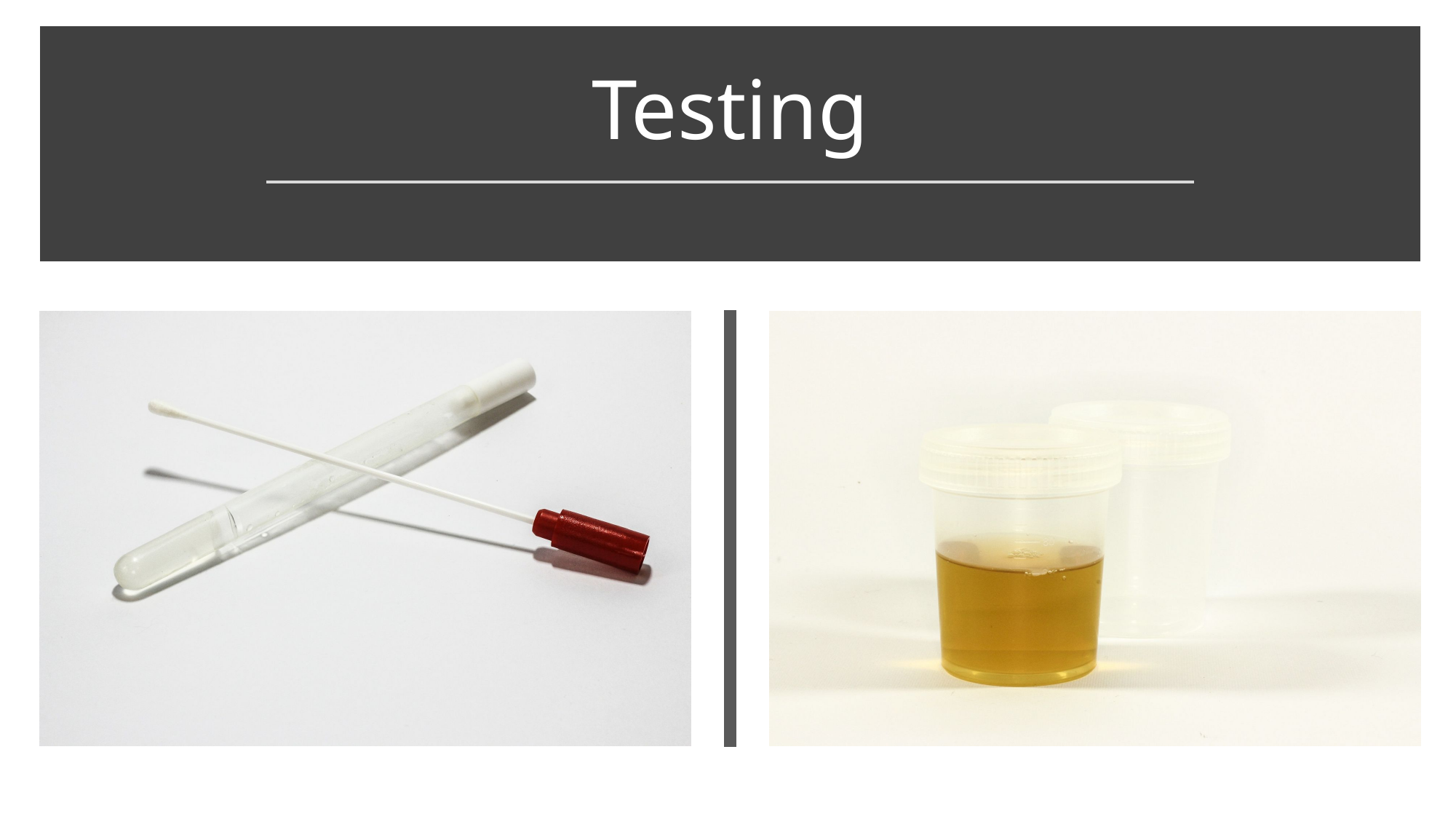

# Testing

## Slide 15
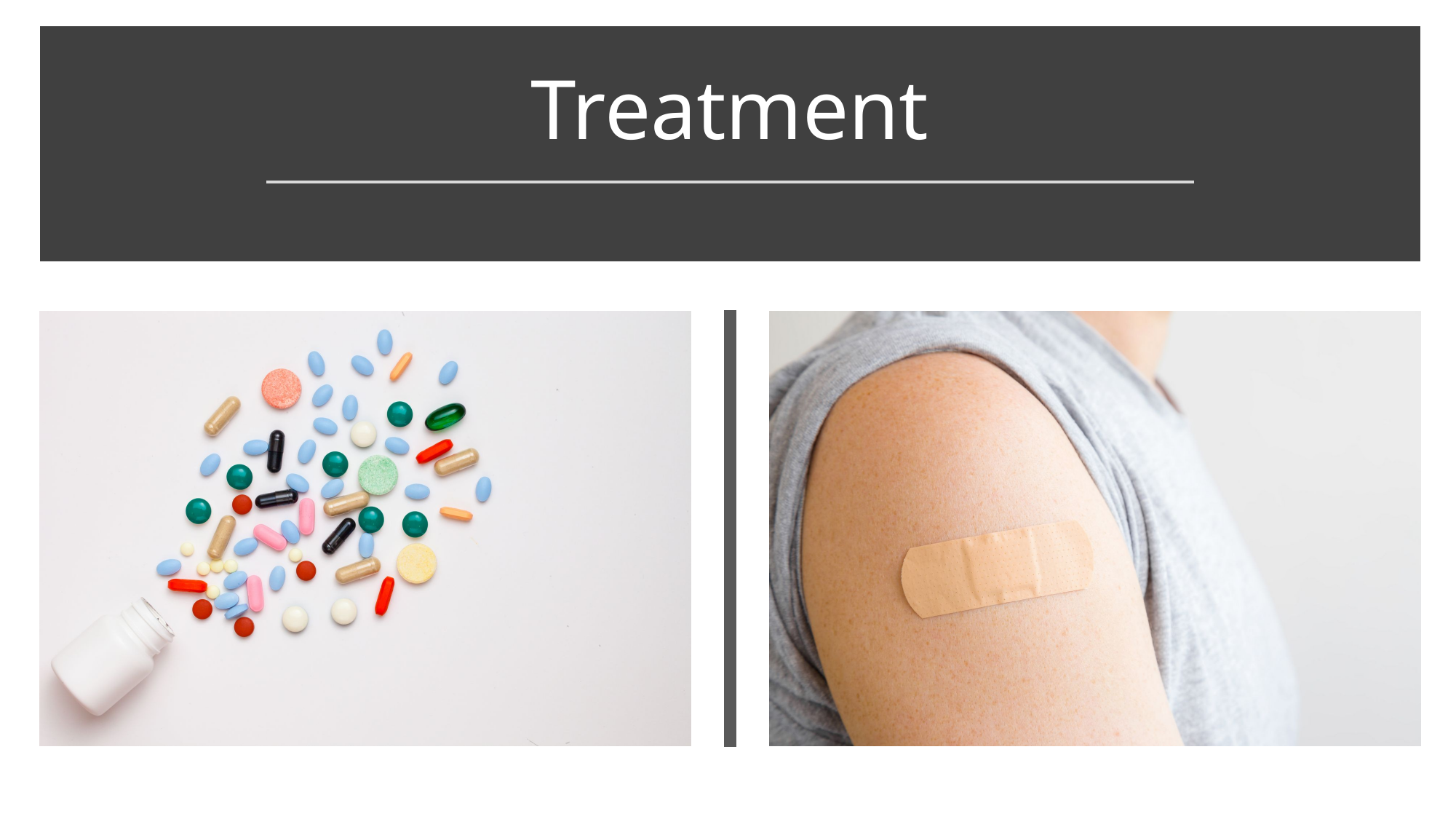

# Treatment

## Slide 16
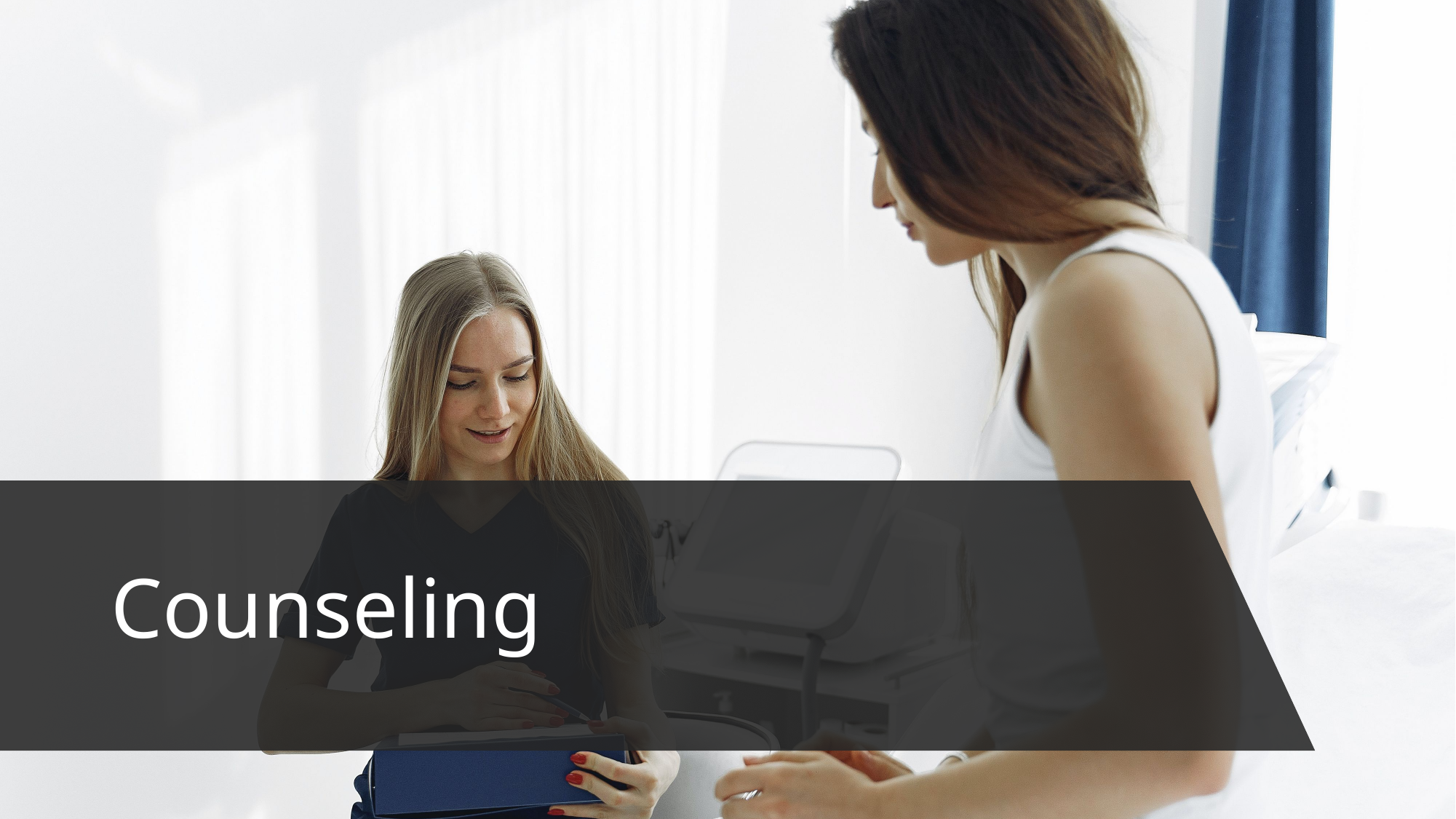

# Counseling

## Slide 17
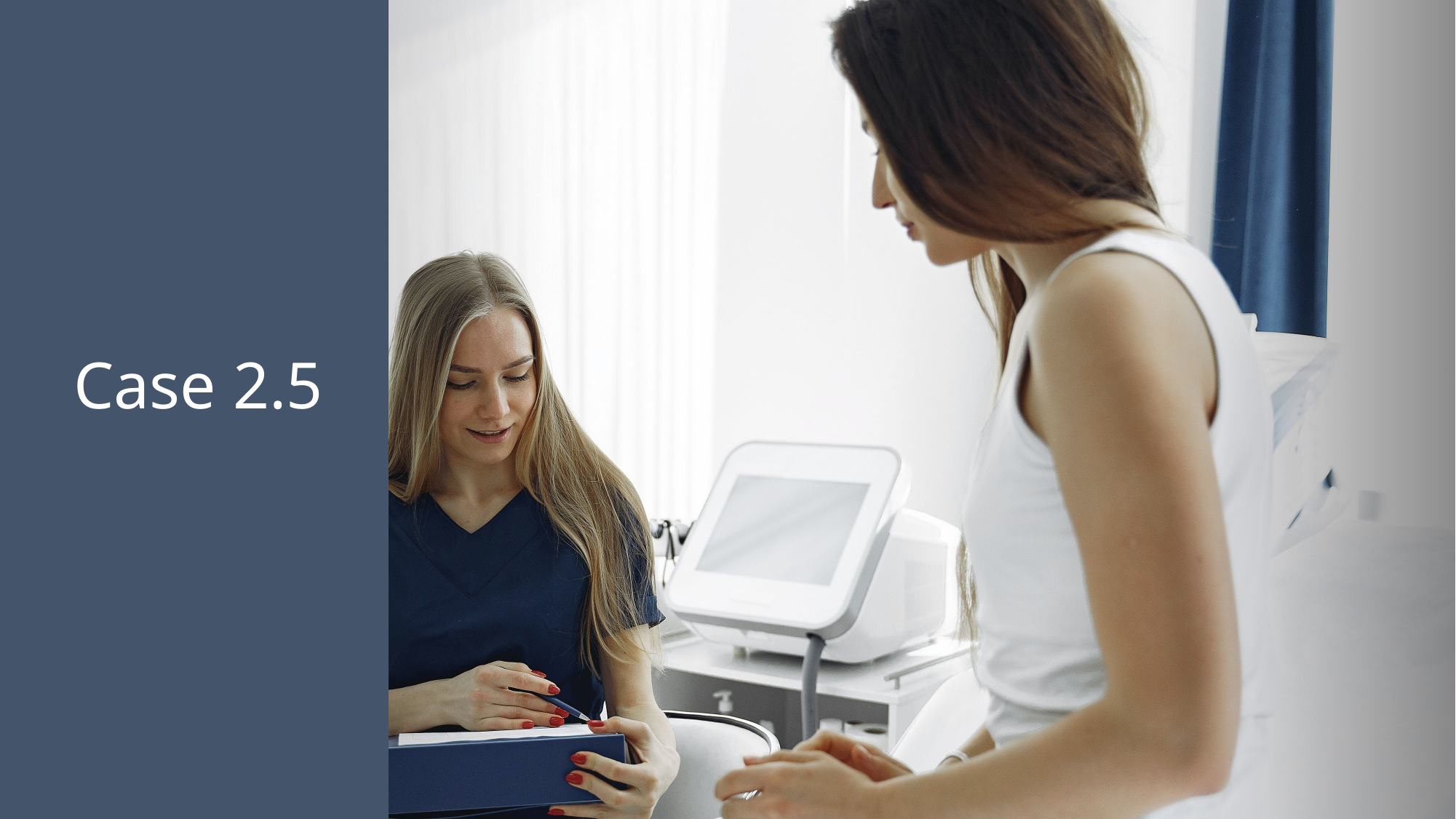

# Case 2.5

## Slide 18
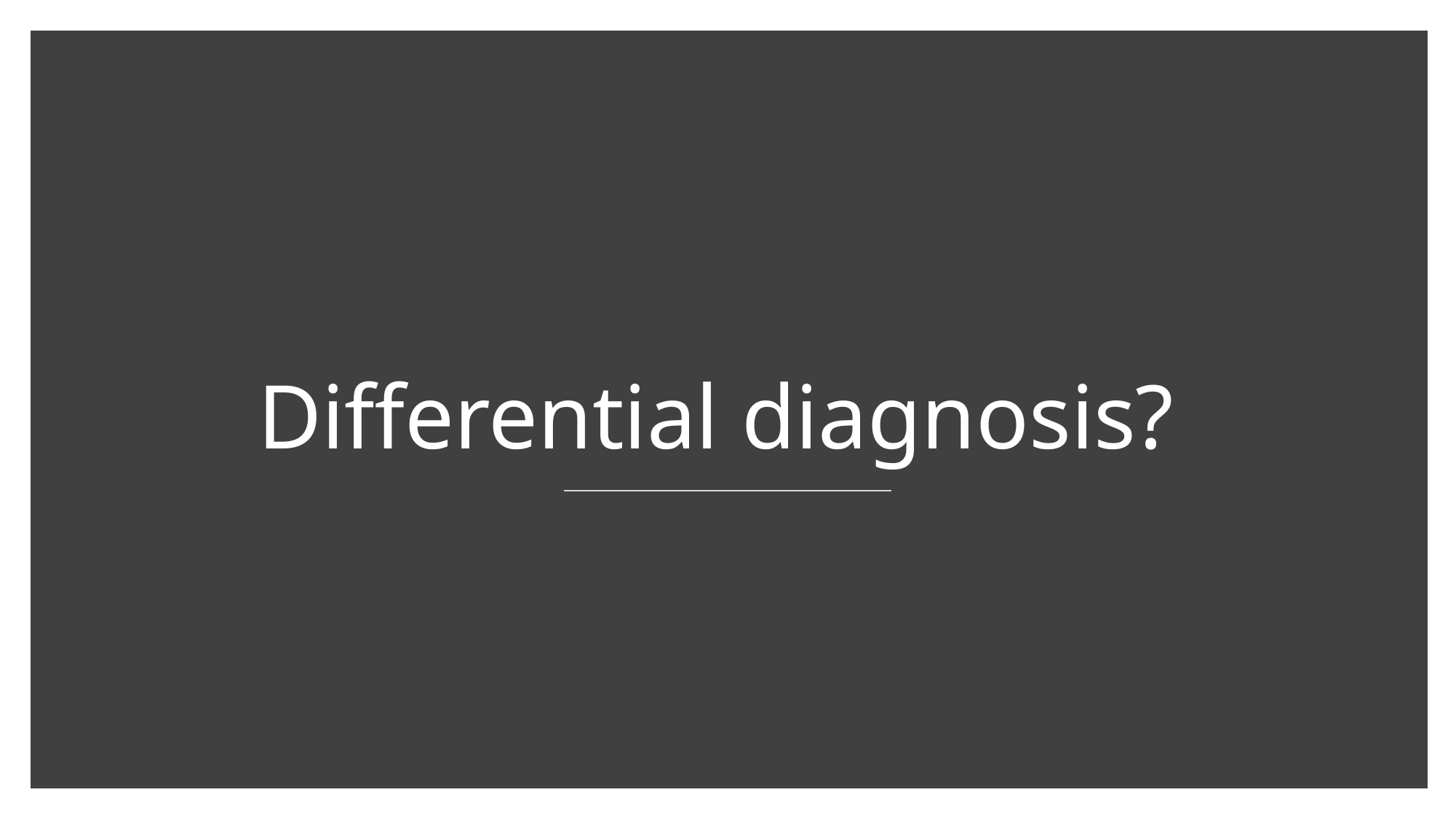

# Differential diagnosis?

## Slide 19
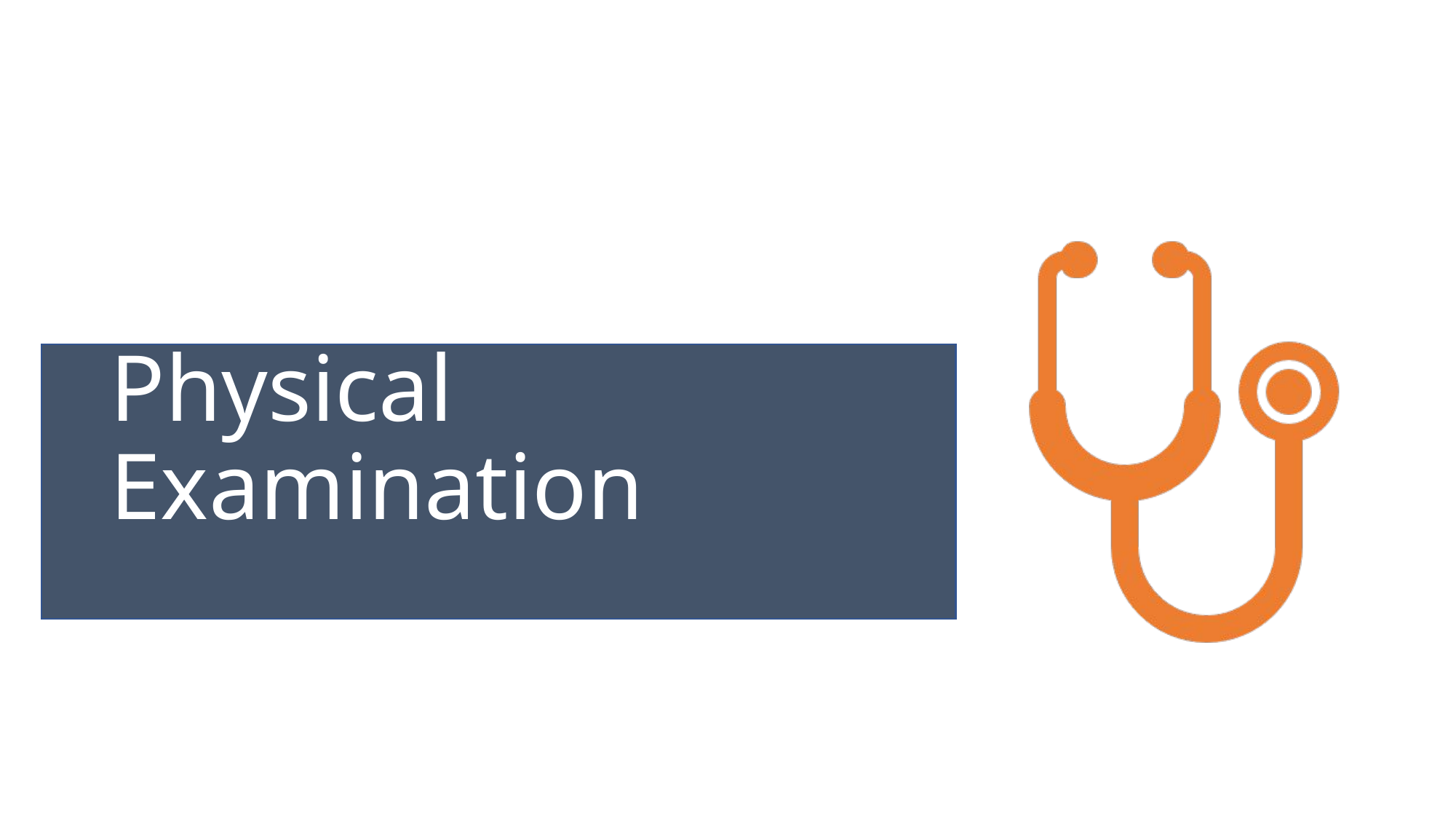

# Physical Examination

## Slide 20
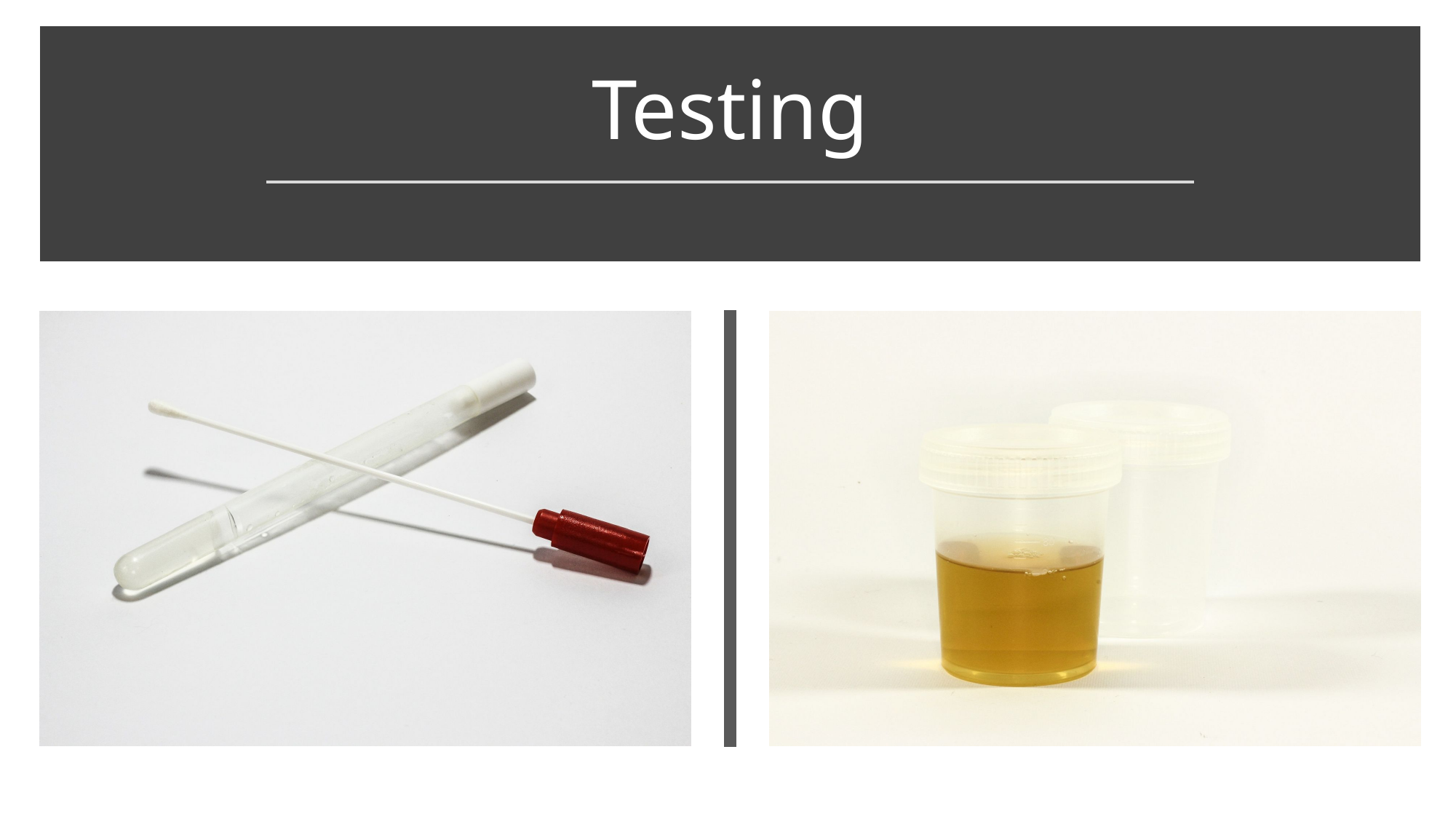

# Testing

## Slide 21
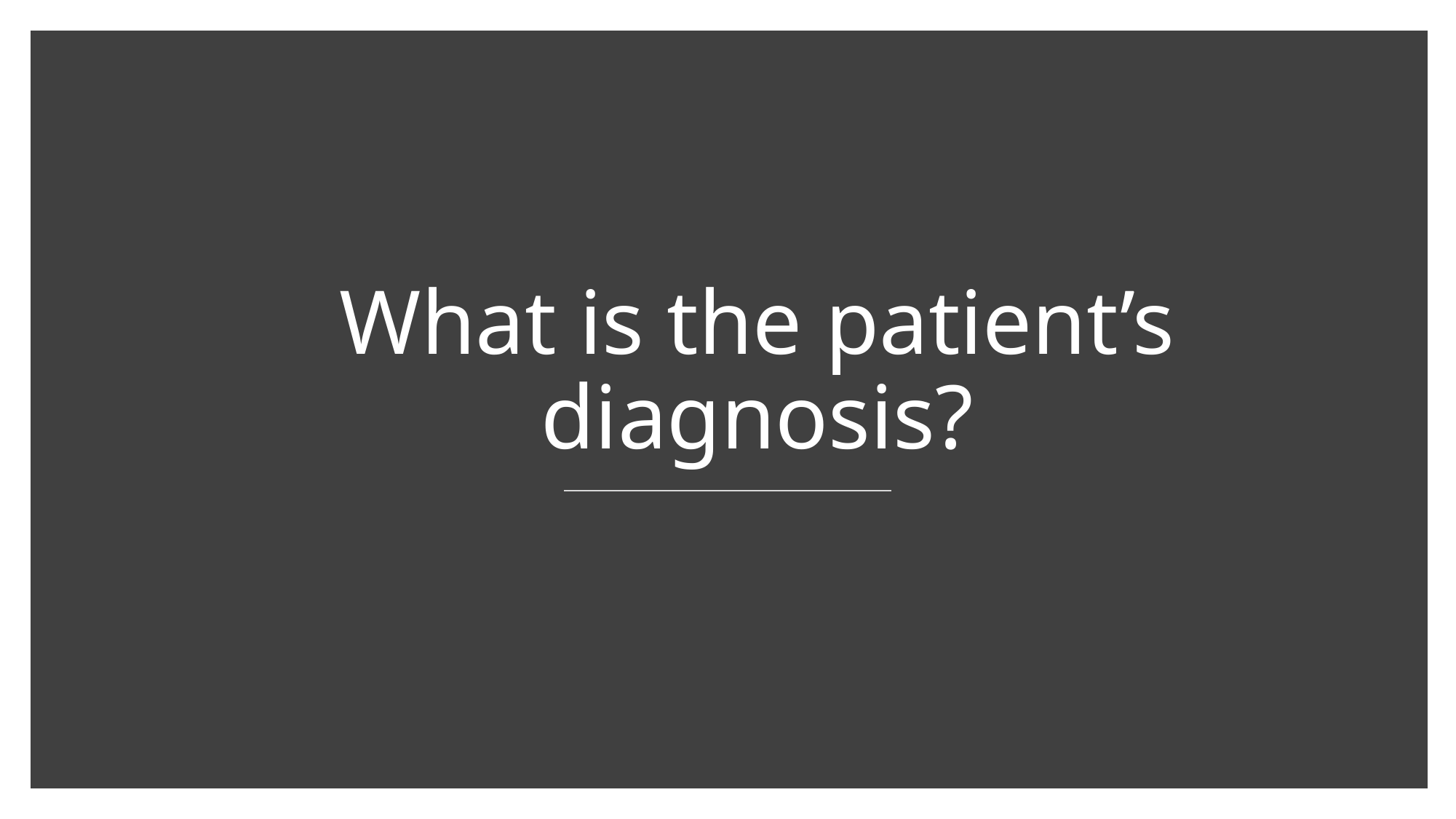

# What is the patient’s diagnosis?

## Slide 22
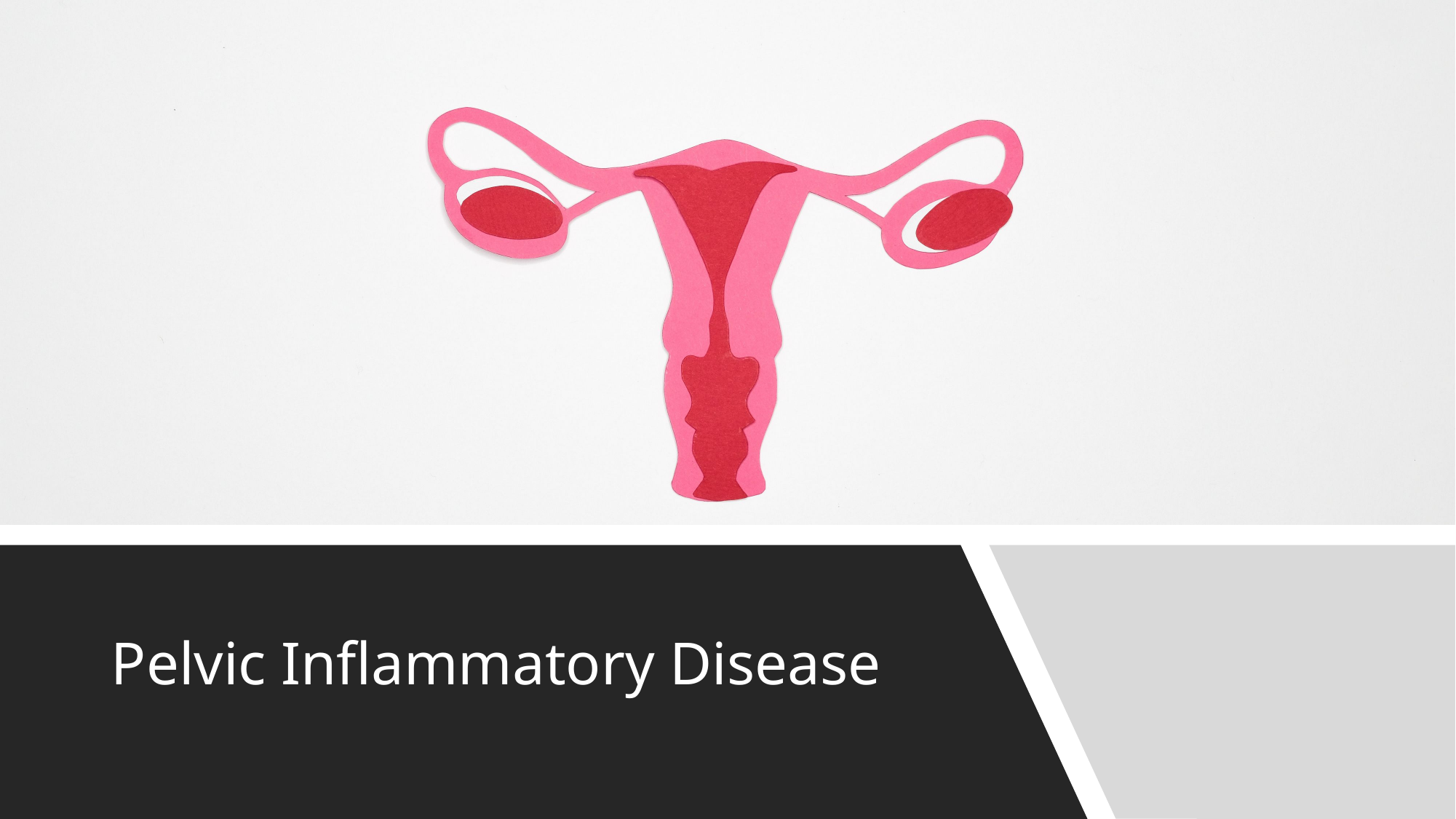

# Pelvic Inflammatory Disease

## Slide 23
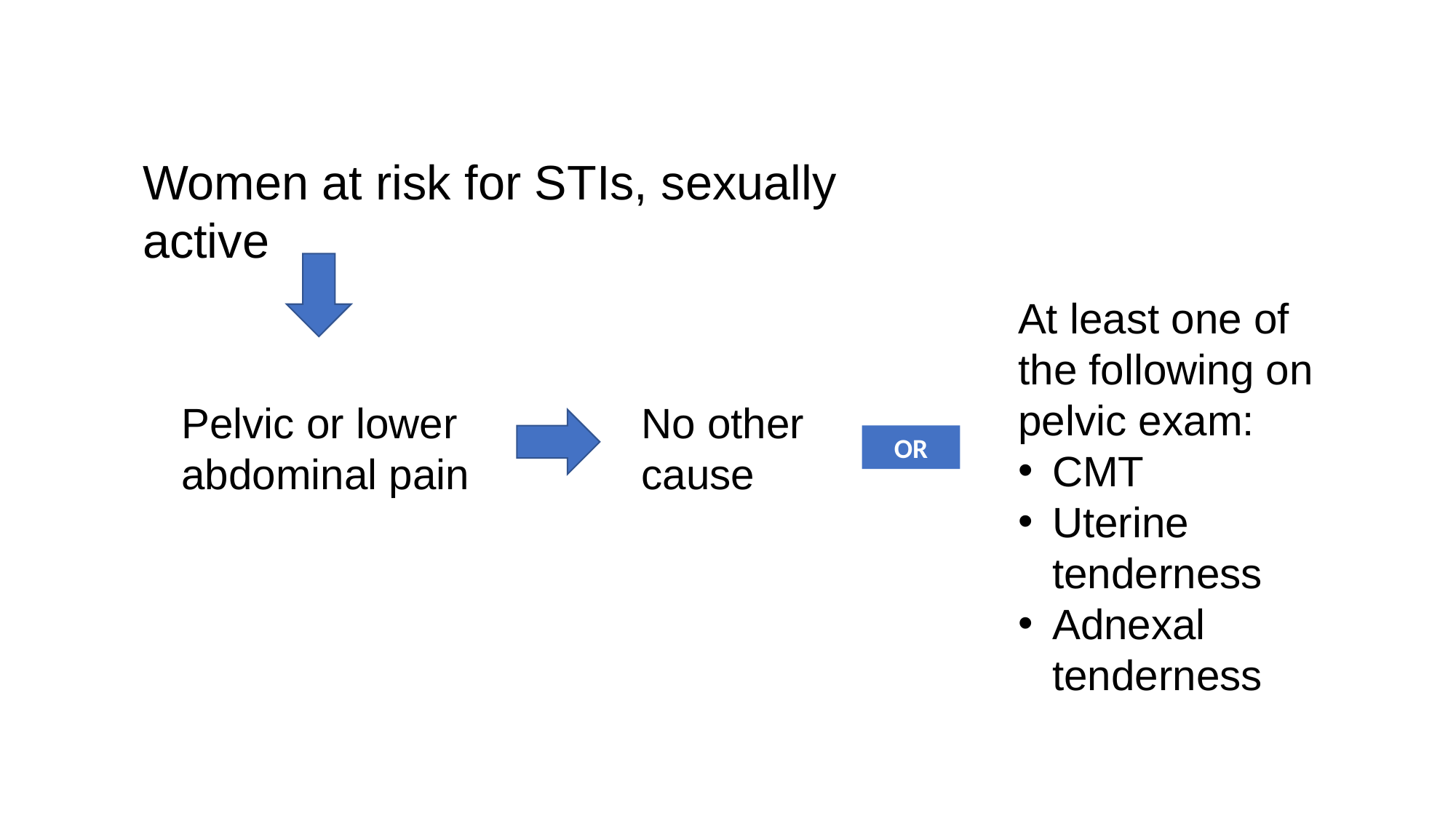

Women at risk for STIs, sexually active
At least one of the following on pelvic exam:
CMT
Uterine tenderness
Adnexal tenderness
Pelvic or lower abdominal pain
No other cause
OR

## Slide 24
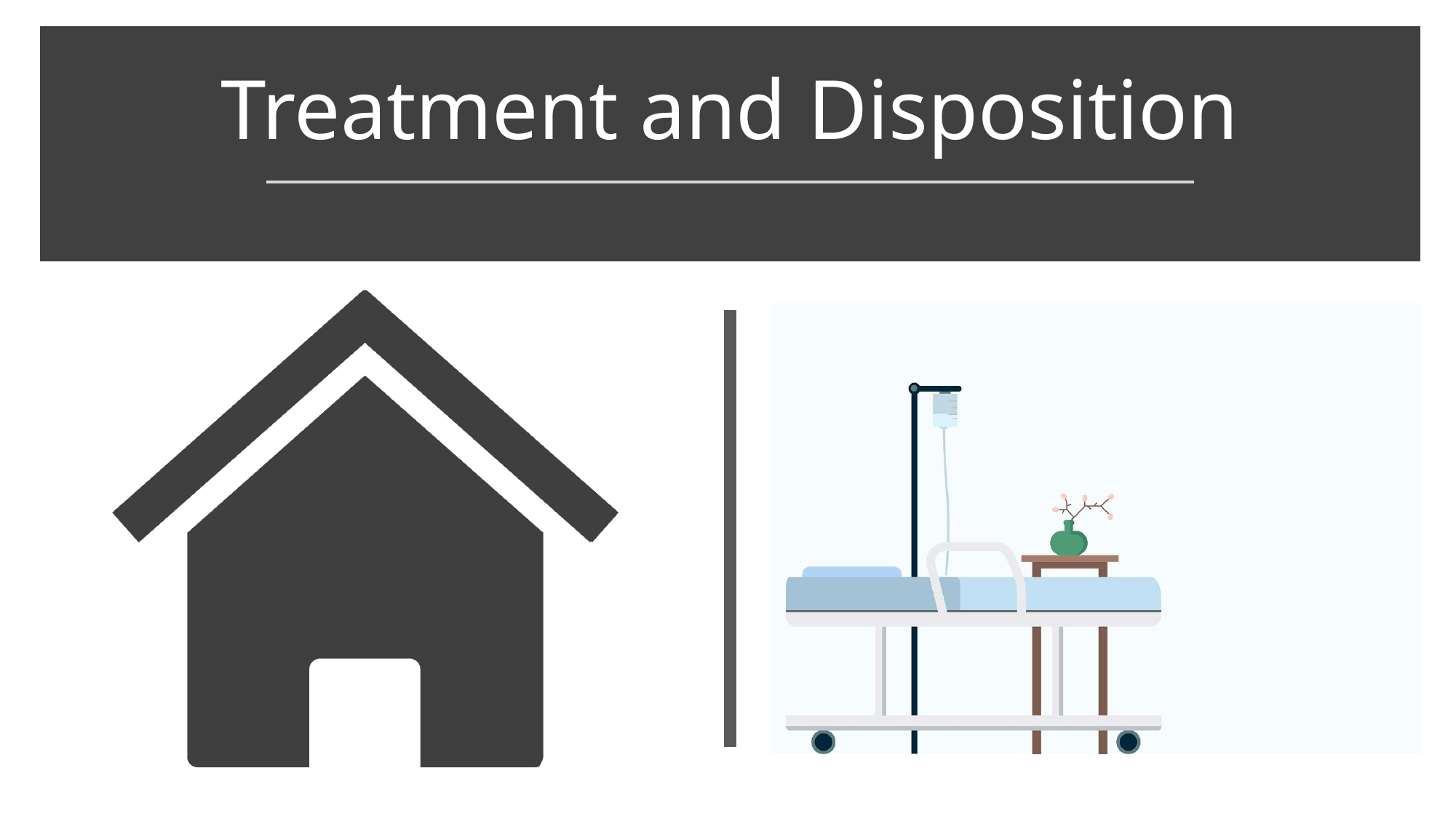

# Treatment and Disposition

## Slide 25
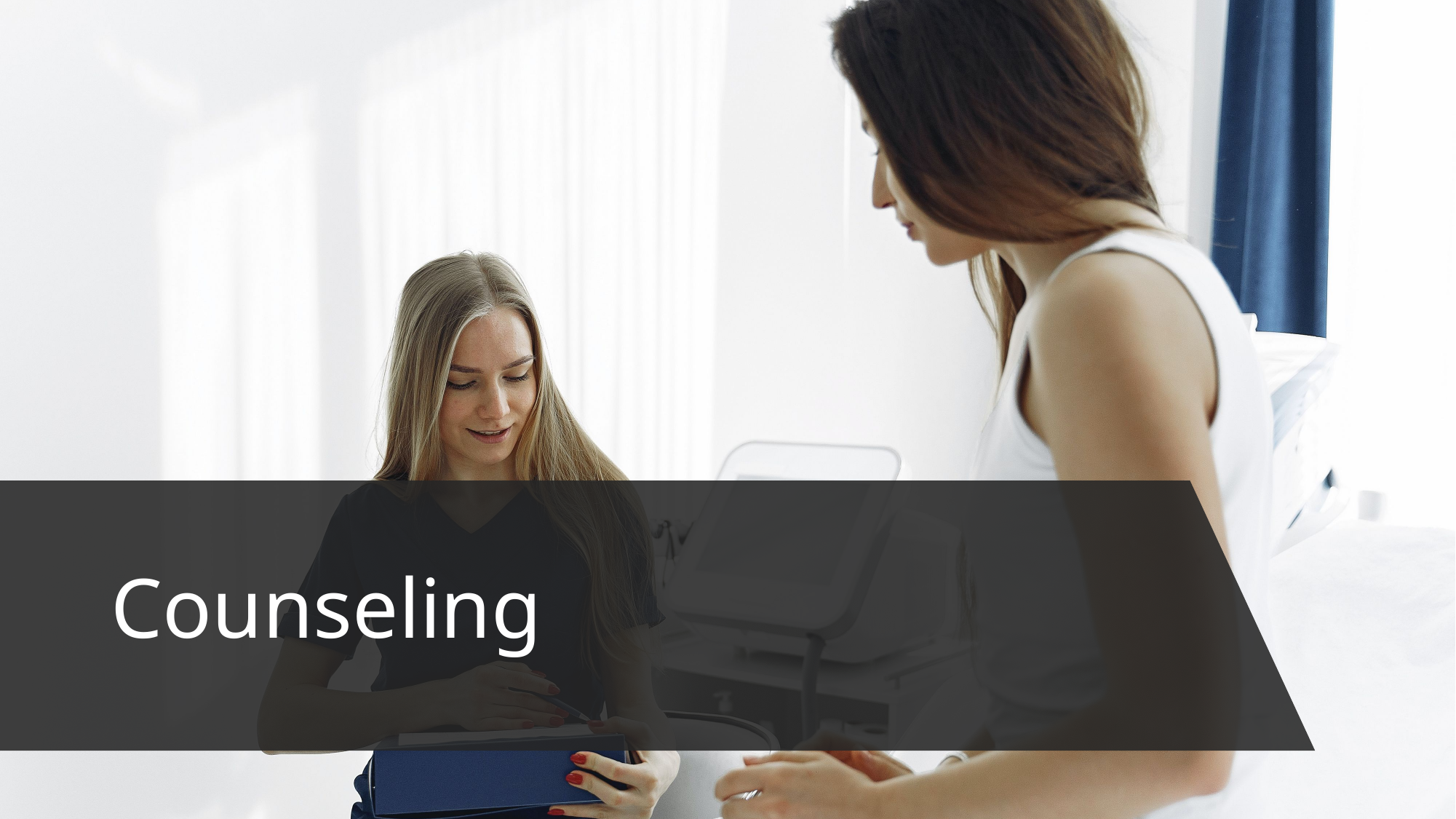

# Counseling

## Slide 26
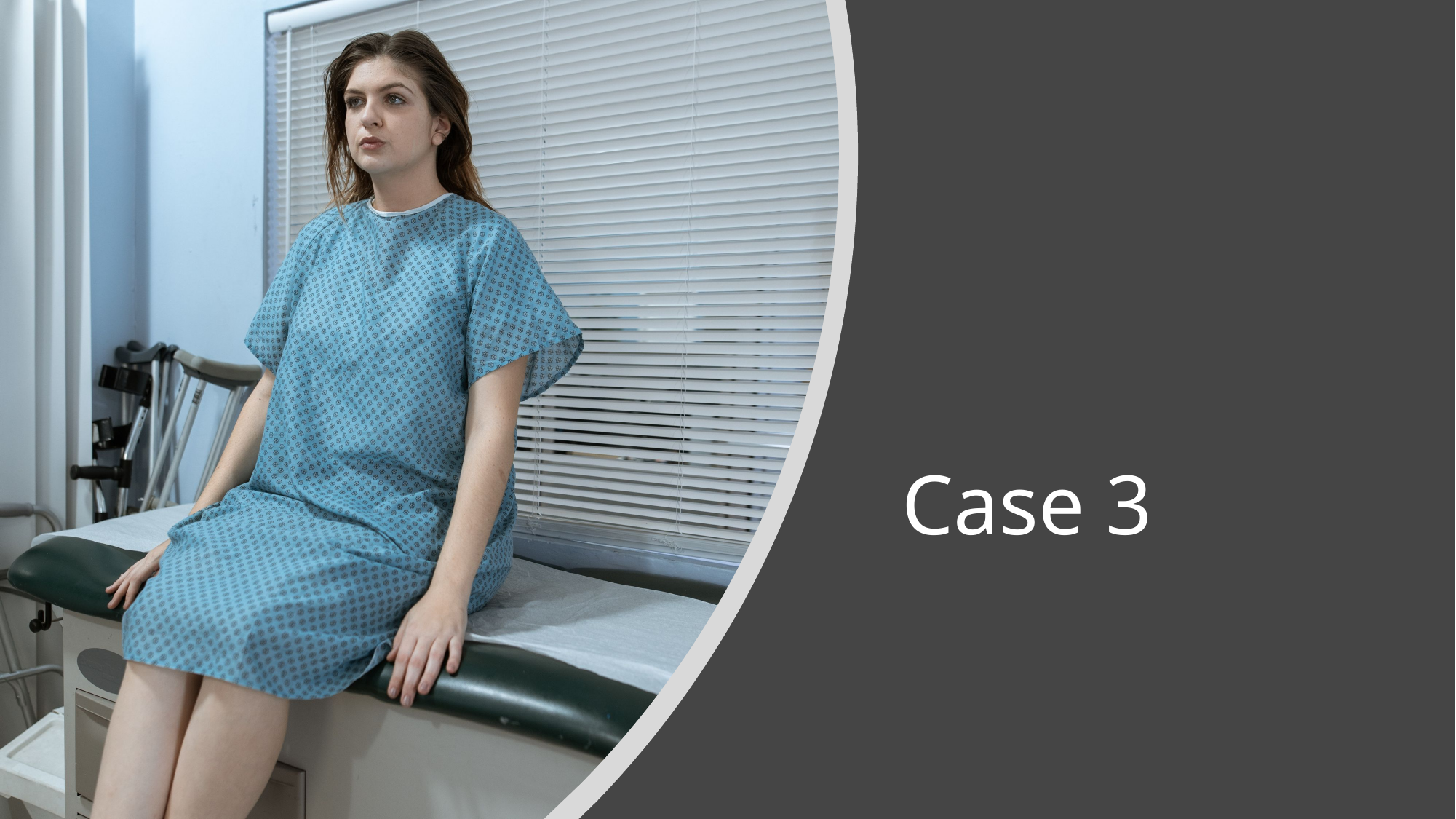

# Case 3

## Slide 27
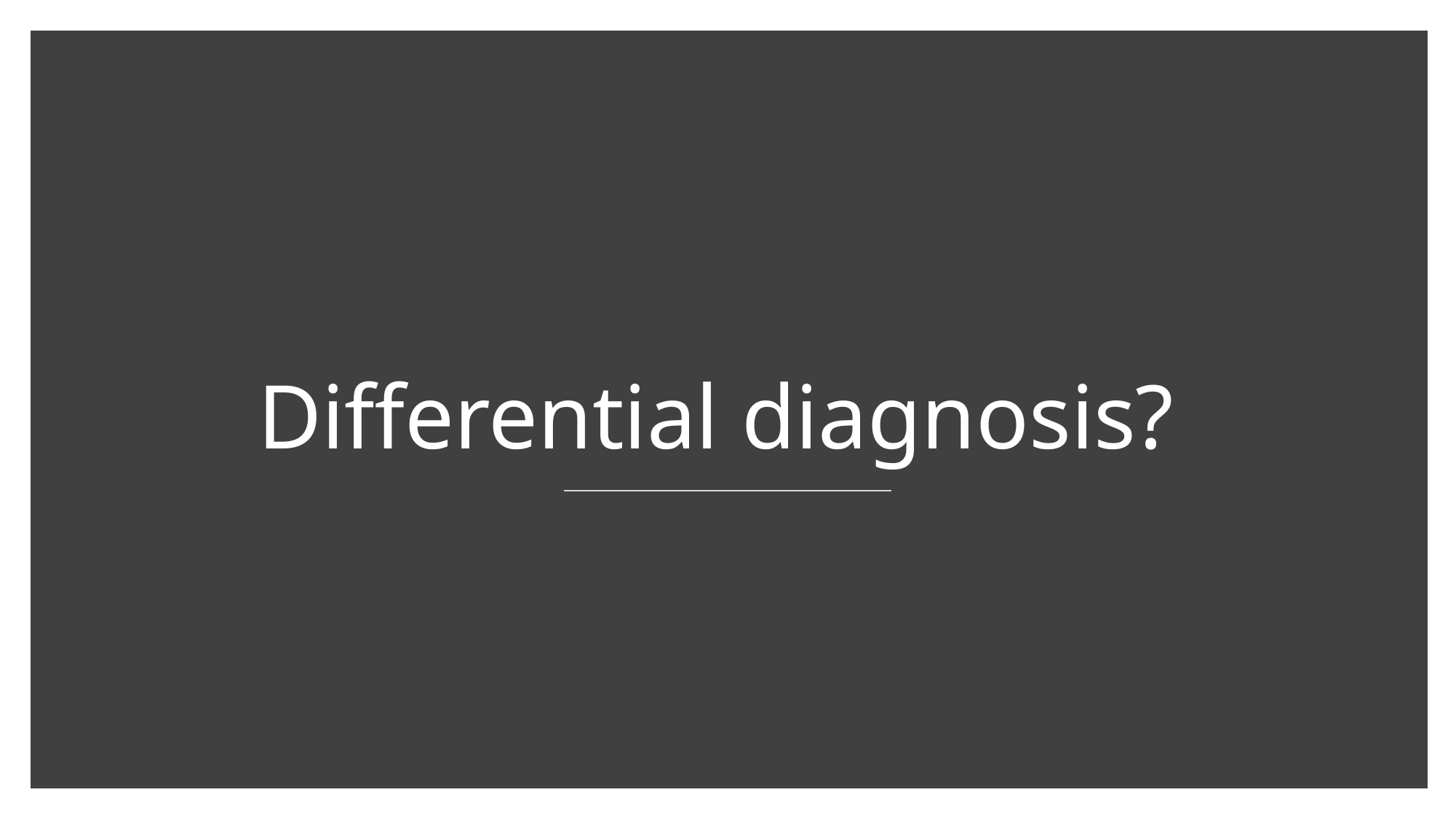

# Differential diagnosis?

## Slide 28
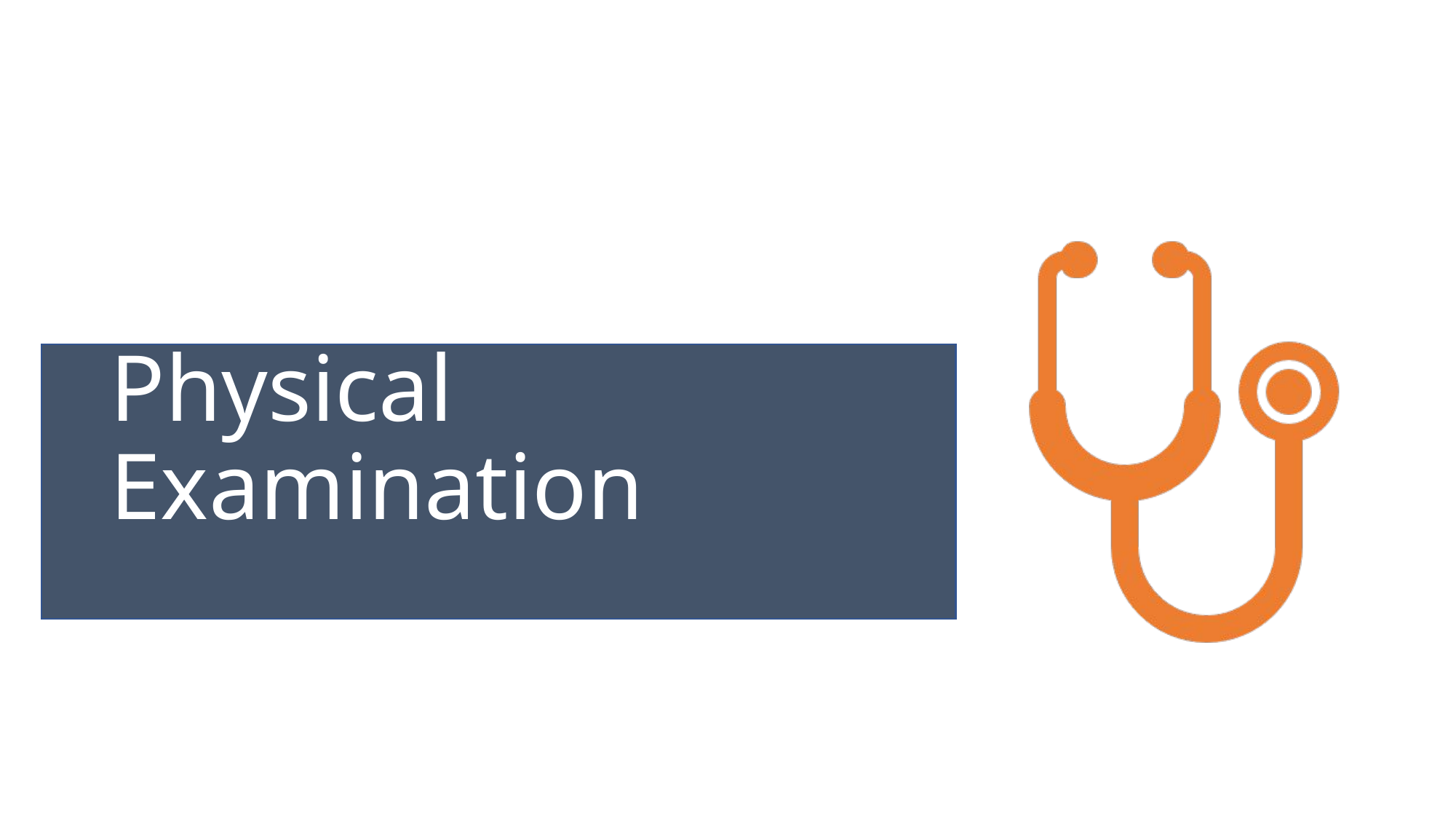

# Physical Examination

## Slide 29
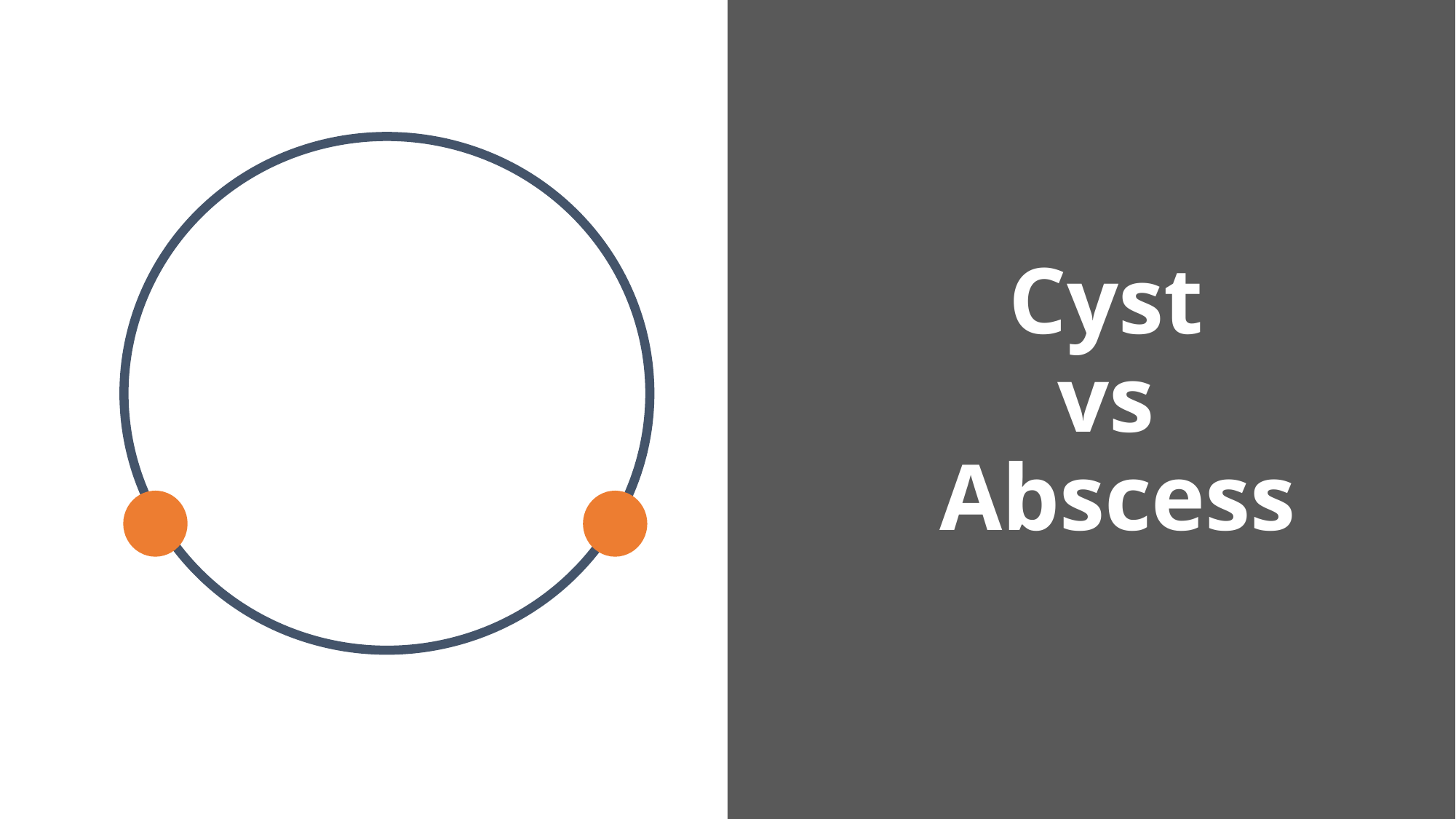

# Cyst vs Abscess

## Slide 30
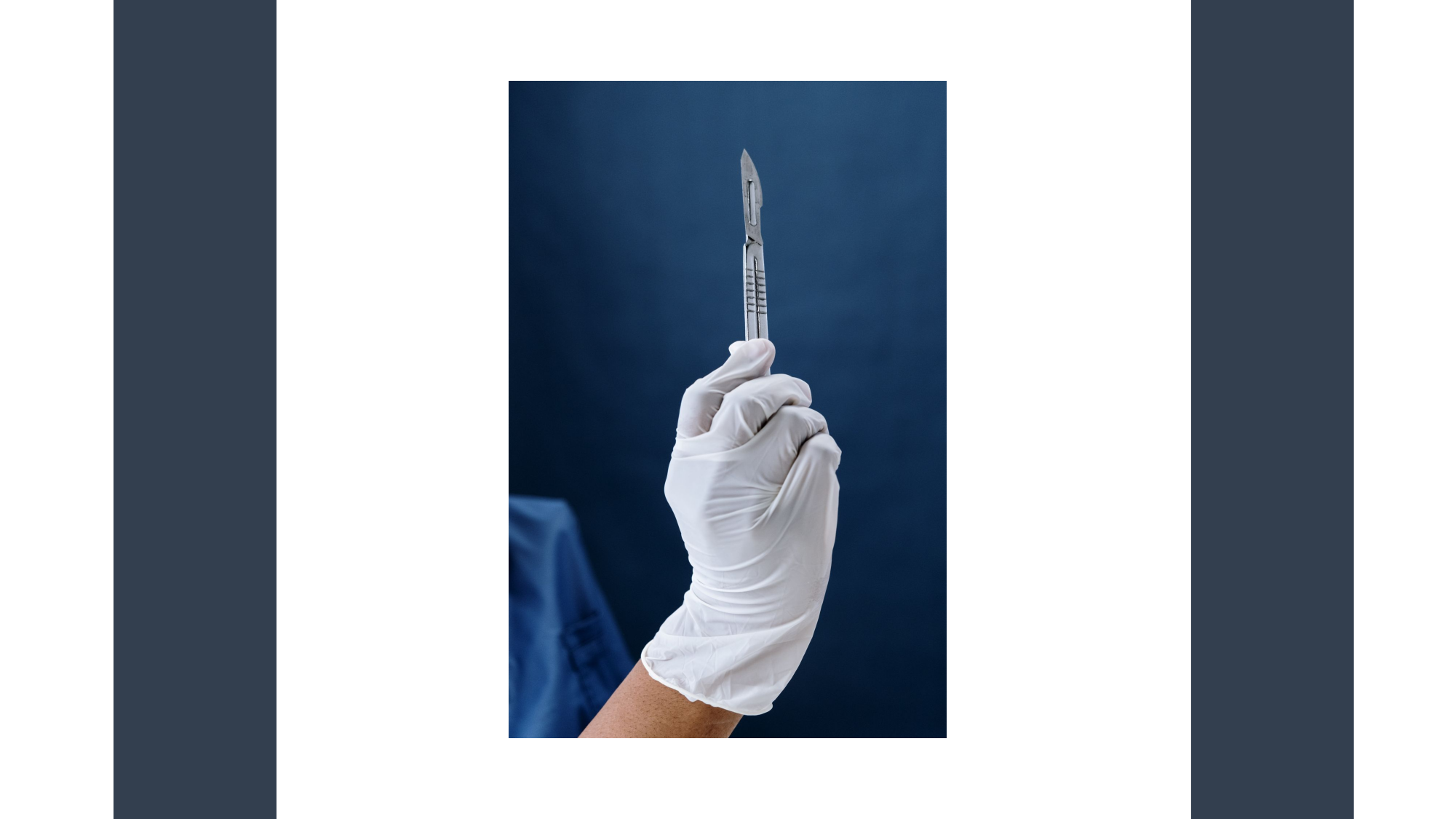

## Slide 31
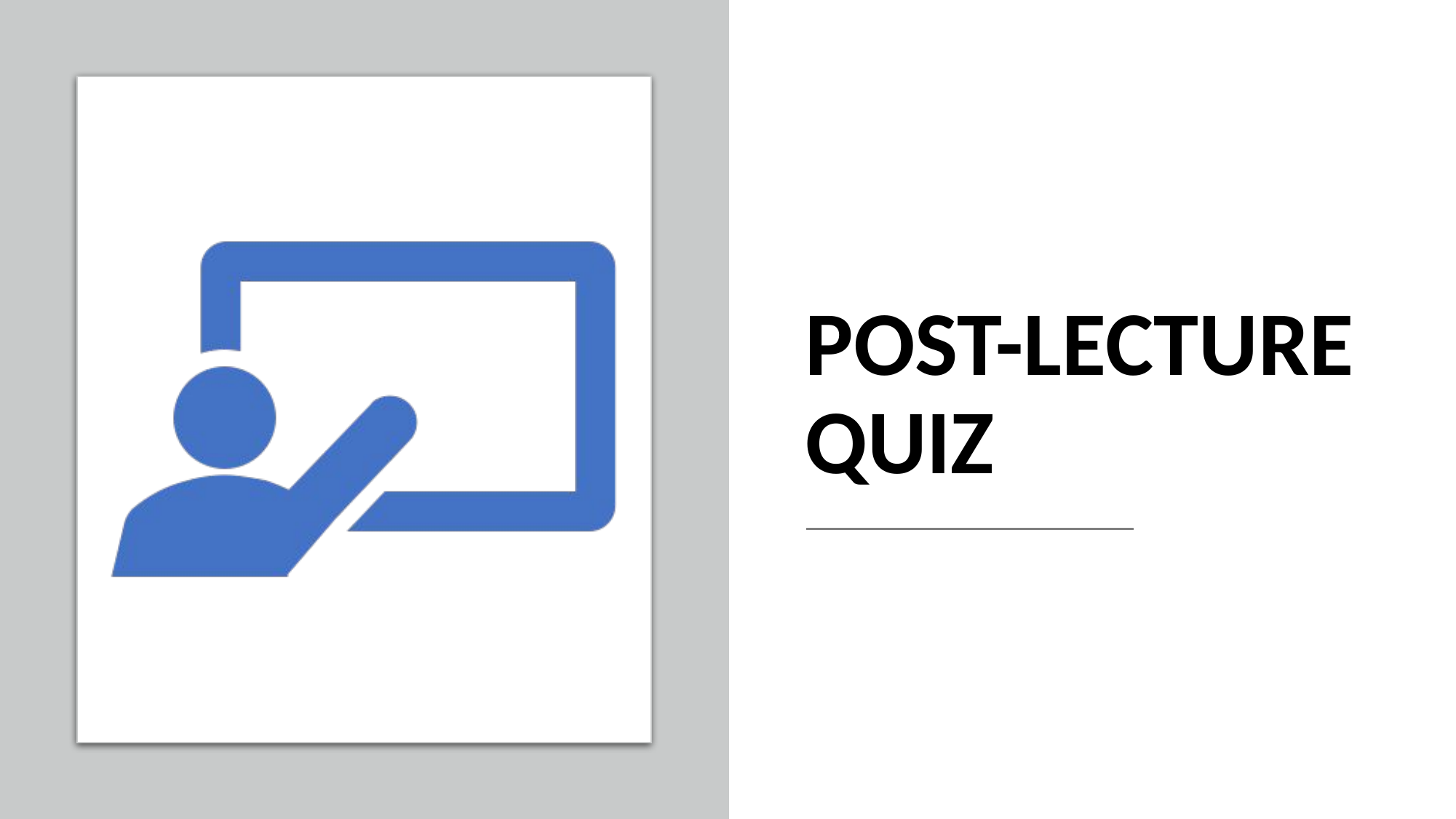

# POST-LECTURE QUIZ

## Slide 32
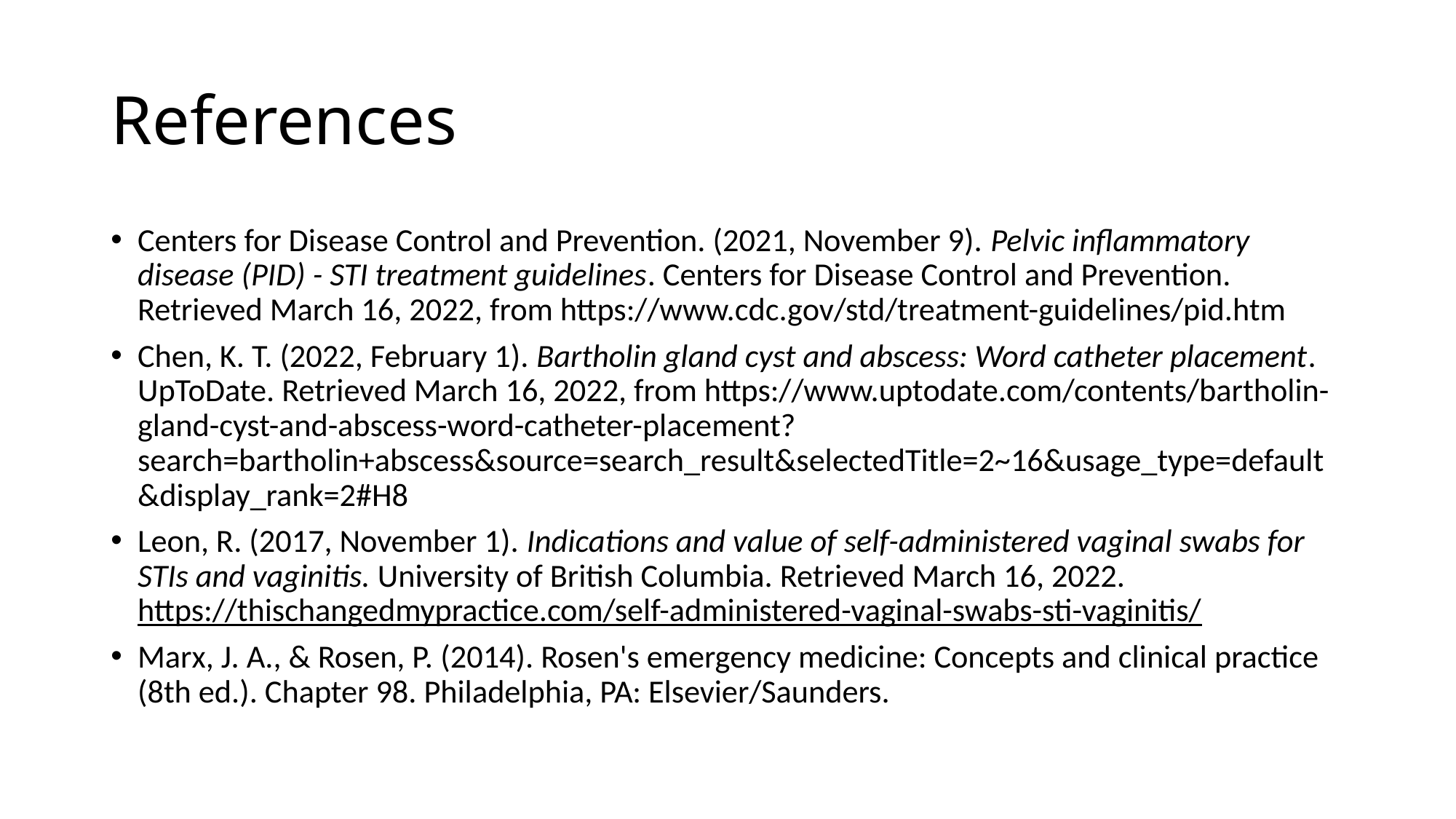

# References
Centers for Disease Control and Prevention. (2021, November 9). Pelvic inflammatory disease (PID) - STI treatment guidelines. Centers for Disease Control and Prevention. Retrieved March 16, 2022, from https://www.cdc.gov/std/treatment-guidelines/pid.htm
Chen, K. T. (2022, February 1). Bartholin gland cyst and abscess: Word catheter placement. UpToDate. Retrieved March 16, 2022, from https://www.uptodate.com/contents/bartholin-gland-cyst-and-abscess-word-catheter-placement?search=bartholin+abscess&source=search_result&selectedTitle=2~16&usage_type=default&display_rank=2#H8
Leon, R. (2017, November 1). Indications and value of self-administered vaginal swabs for STIs and vaginitis. University of British Columbia. Retrieved March 16, 2022. https://thischangedmypractice.com/self-administered-vaginal-swabs-sti-vaginitis/
Marx, J. A., & Rosen, P. (2014). Rosen's emergency medicine: Concepts and clinical practice (8th ed.). Chapter 98. Philadelphia, PA: Elsevier/Saunders.
