# Supplementary material for: Emergency Medicine Obstetrics and Gynecology: A Case-Based Curriculum for Residents
Source: MedEdPORTAL. 2023 Aug 11;19:11330. doi: 10.15766/mep_2374-8265.11330 (PMC10415535; doi:10.15766/mep_2374-8265.11330)
Supplement: Supplementary file 1 — Ectopic Pregnancy and Emergencies in the First 20 Weeks.pptxPregnancy Emergencies After 20 Weeks.pptxDelivery Emergencies.pptxPelvic Pain in the Nonpregnant Patient.pptxVaginitis, Cervicitis, and PID.pptxAbnormal Uterine Bleeding.pptxLabor and Perimortem C-Section.pptxSession Review Questions.docxPrecurriculum Survey.docxPostcurriculum Survey.docx [file mep_2374-8265.11330-s001.zip › G. Labor and Perimortem C-Section.pptx]

## Slide 1
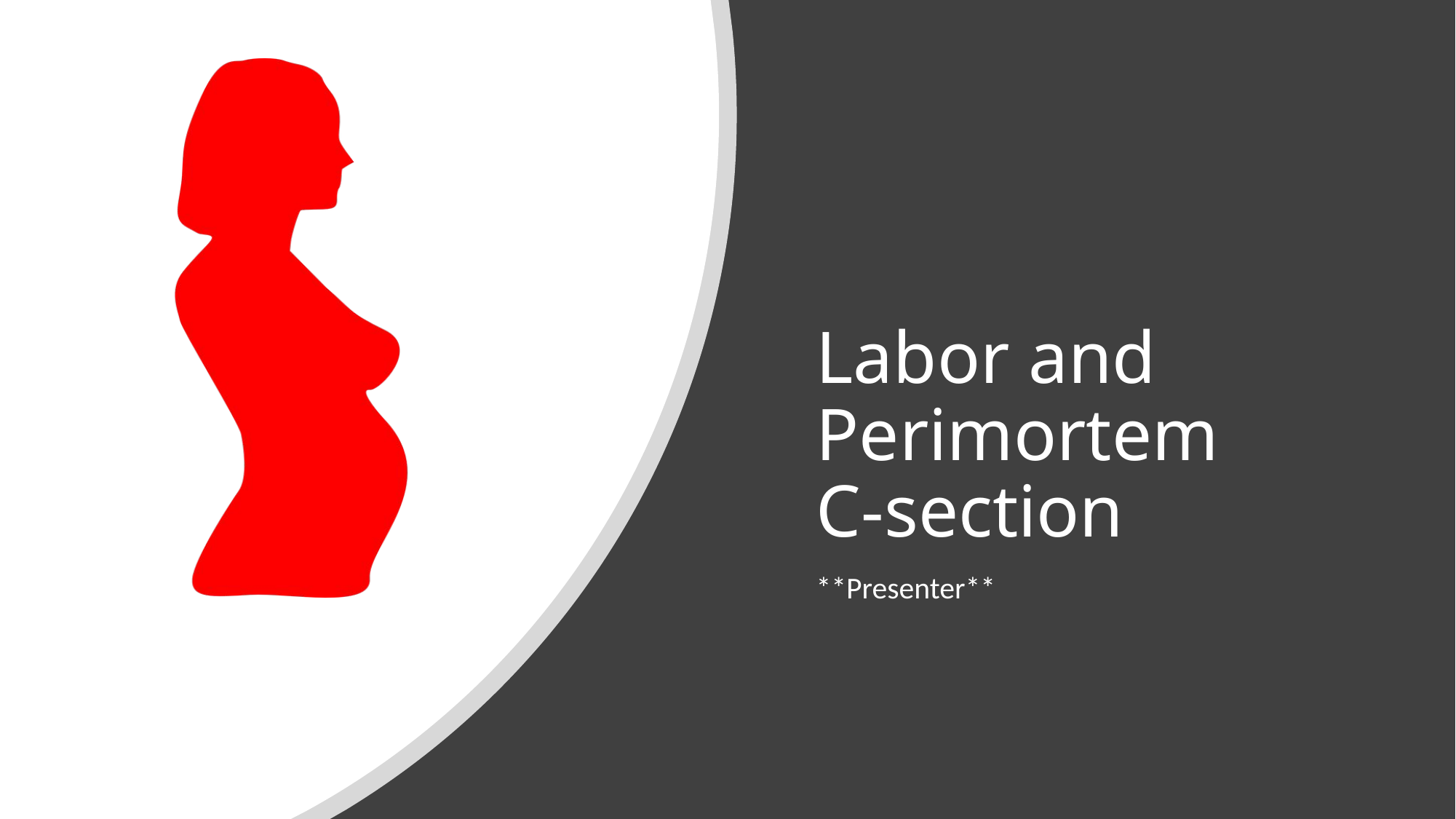

# Labor and Perimortem C-section
**Presenter**

## Slide 2
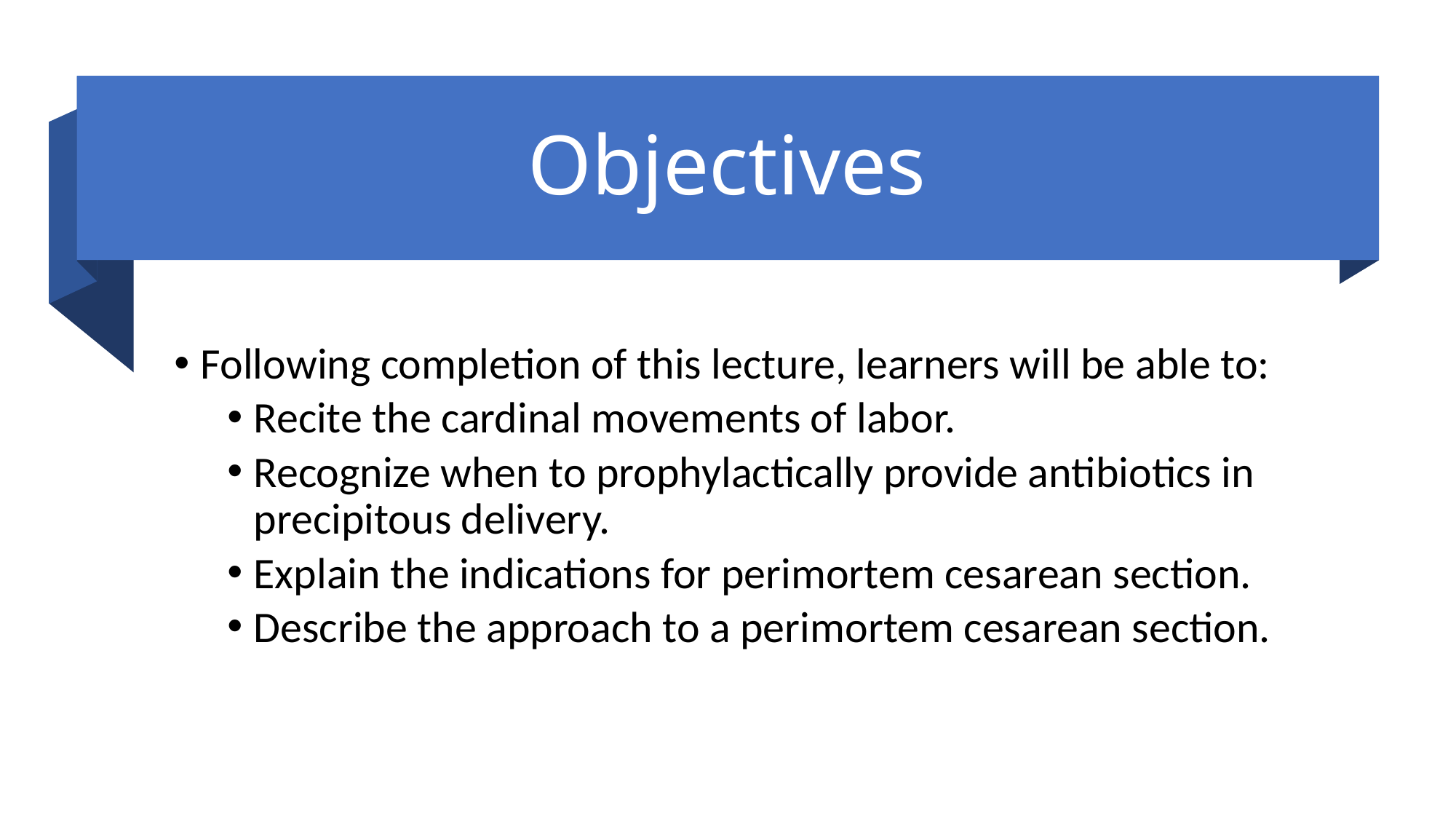

# Objectives
Following completion of this lecture, learners will be able to:
Recite the cardinal movements of labor.
Recognize when to prophylactically provide antibiotics in precipitous delivery.
Explain the indications for perimortem cesarean section.
Describe the approach to a perimortem cesarean section.

## Slide 3
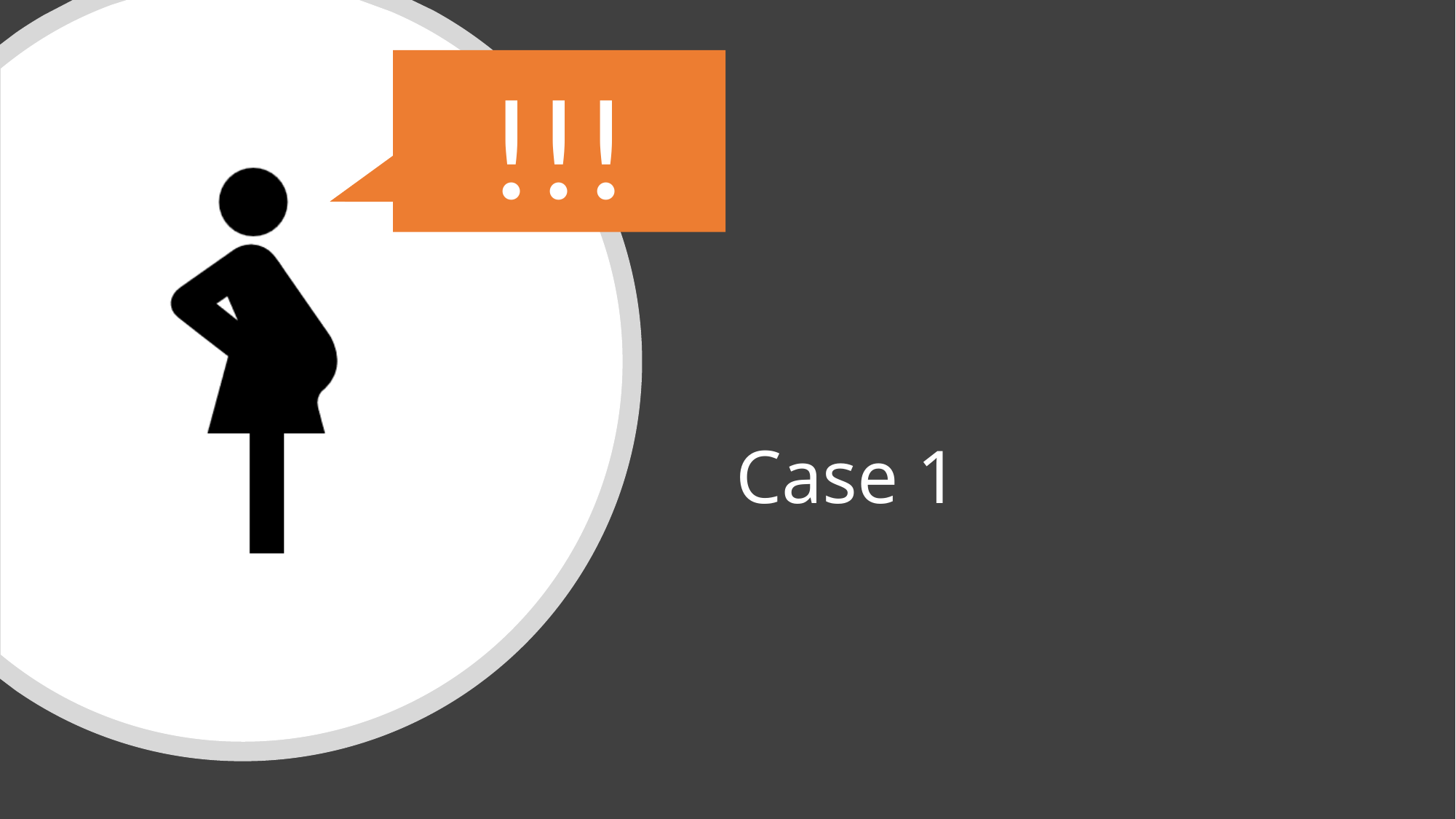

!!!
# Case 1

## Slide 4
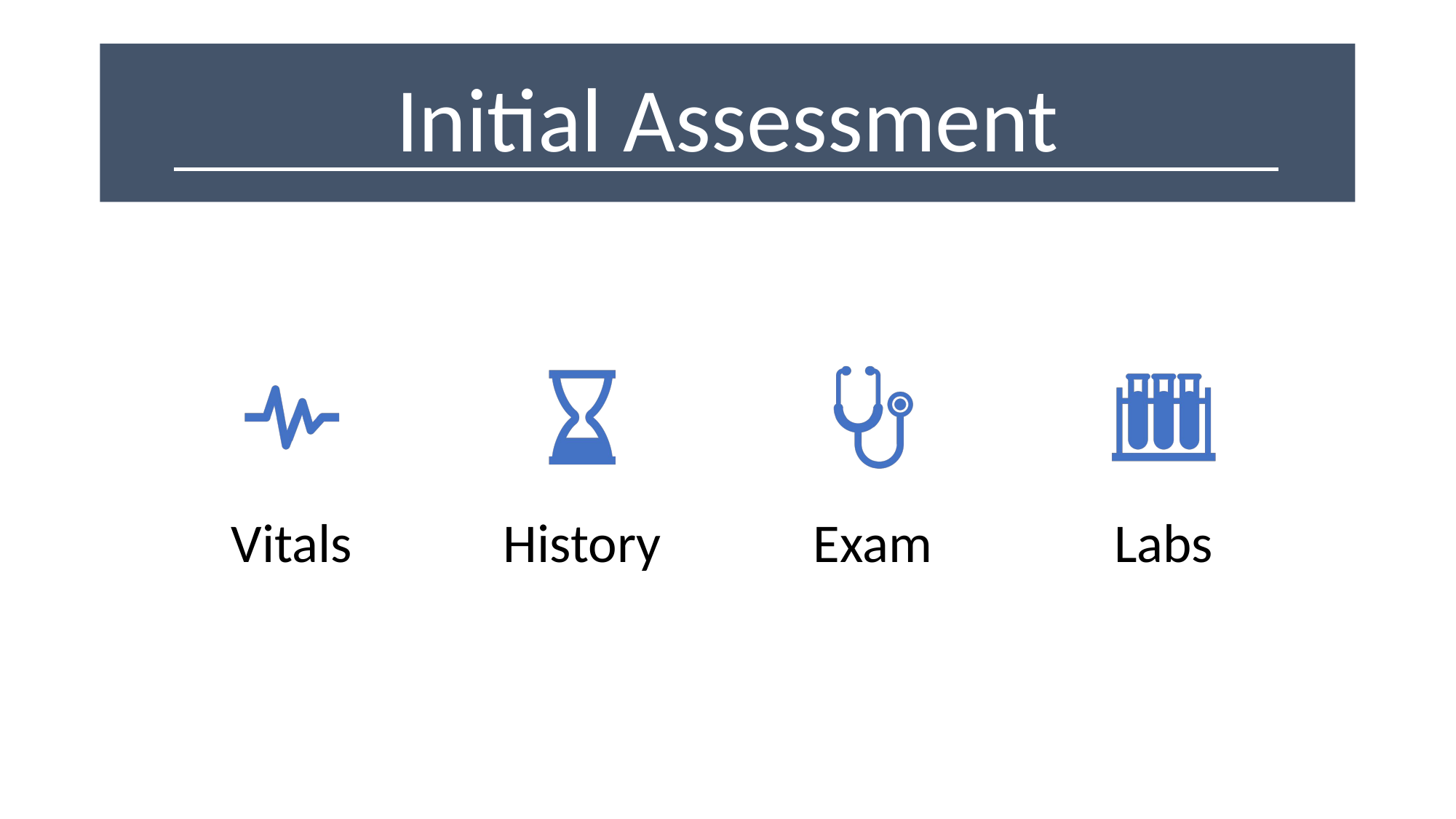

# Initial Assessment
Vitals
History
Exam
Labs

## Slide 5
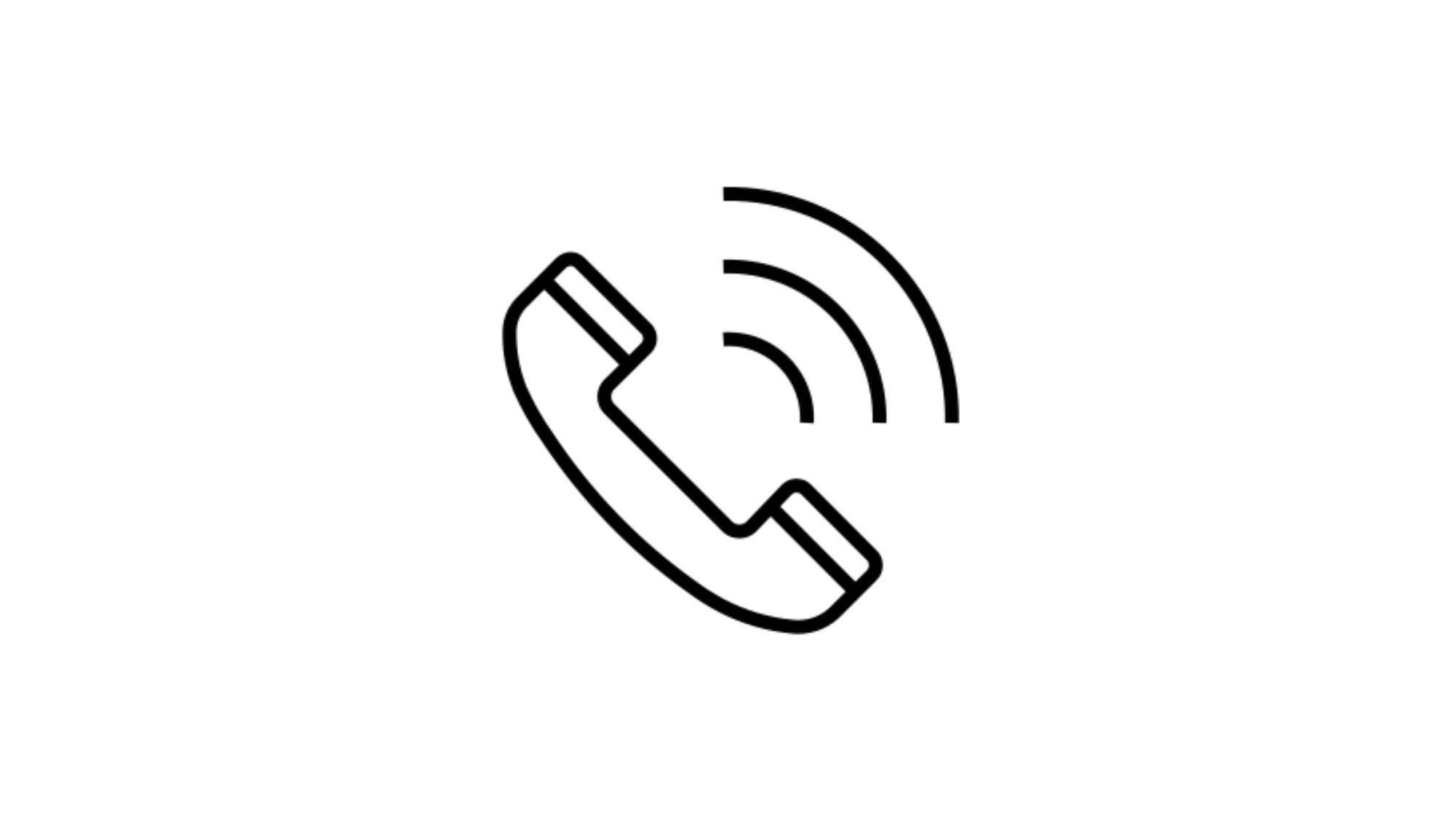

## Slide 6
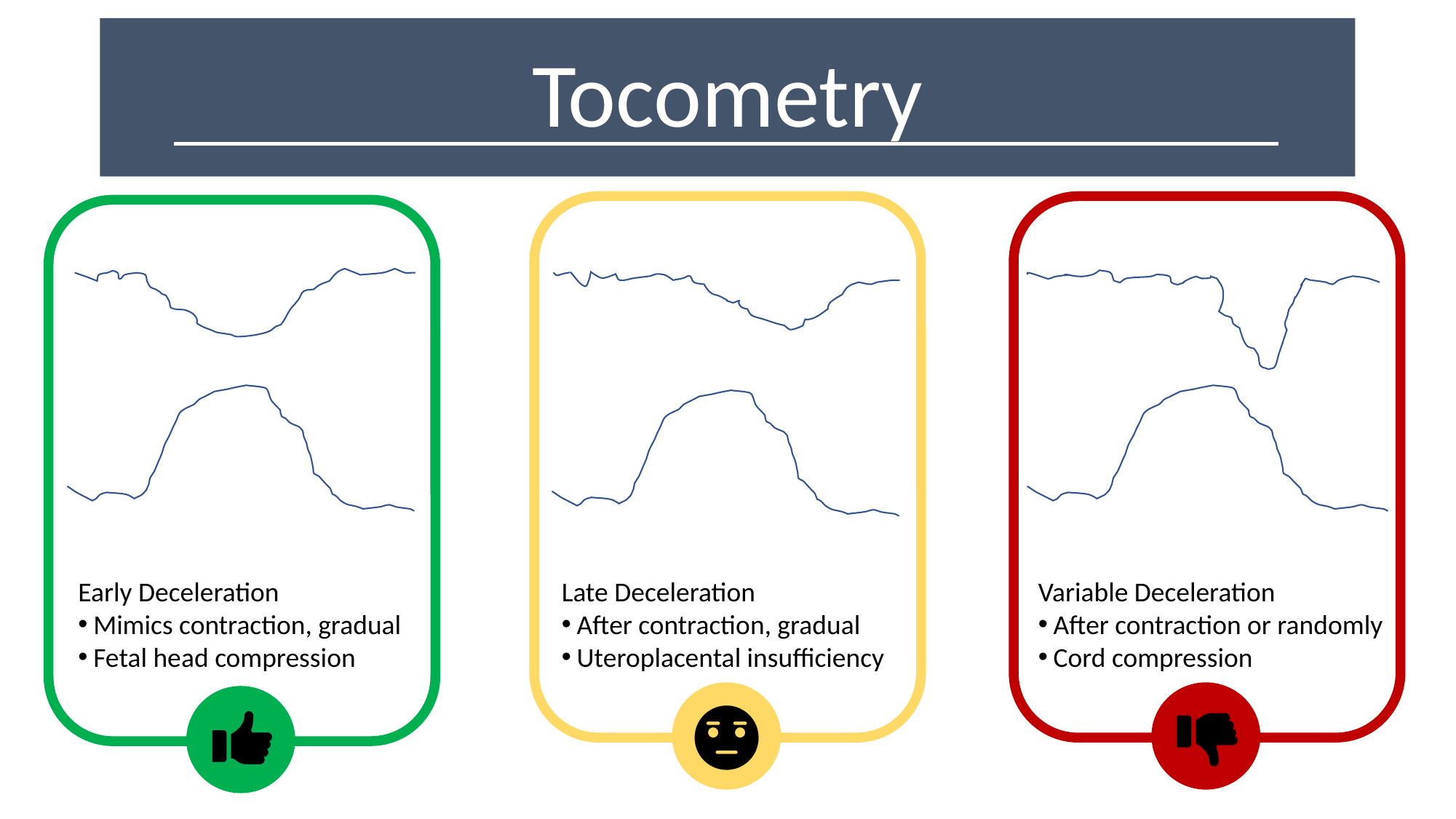

# Tocometry
Late Deceleration
After contraction, gradual
Uteroplacental insufficiency
Variable Deceleration
After contraction or randomly
Cord compression
Early Deceleration
Mimics contraction, gradual
Fetal head compression

## Slide 7
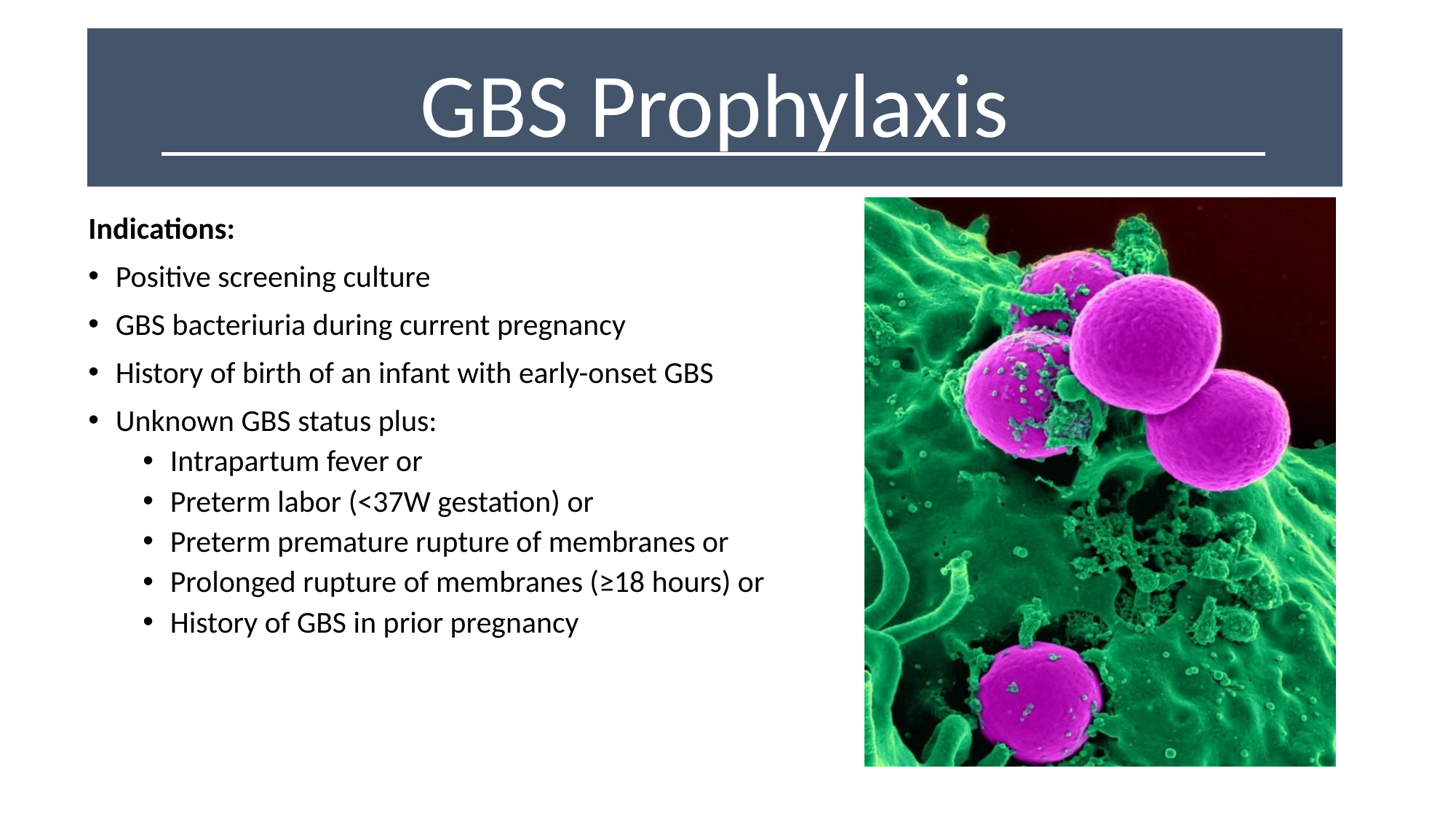

GBS Prophylaxis
Indications:
Positive screening culture
GBS bacteriuria during current pregnancy
History of birth of an infant with early-onset GBS
Unknown GBS status plus:
Intrapartum fever or
Preterm labor (<37W gestation) or
Preterm premature rupture of membranes or
Prolonged rupture of membranes (≥18 hours) or
History of GBS in prior pregnancy

## Slide 8
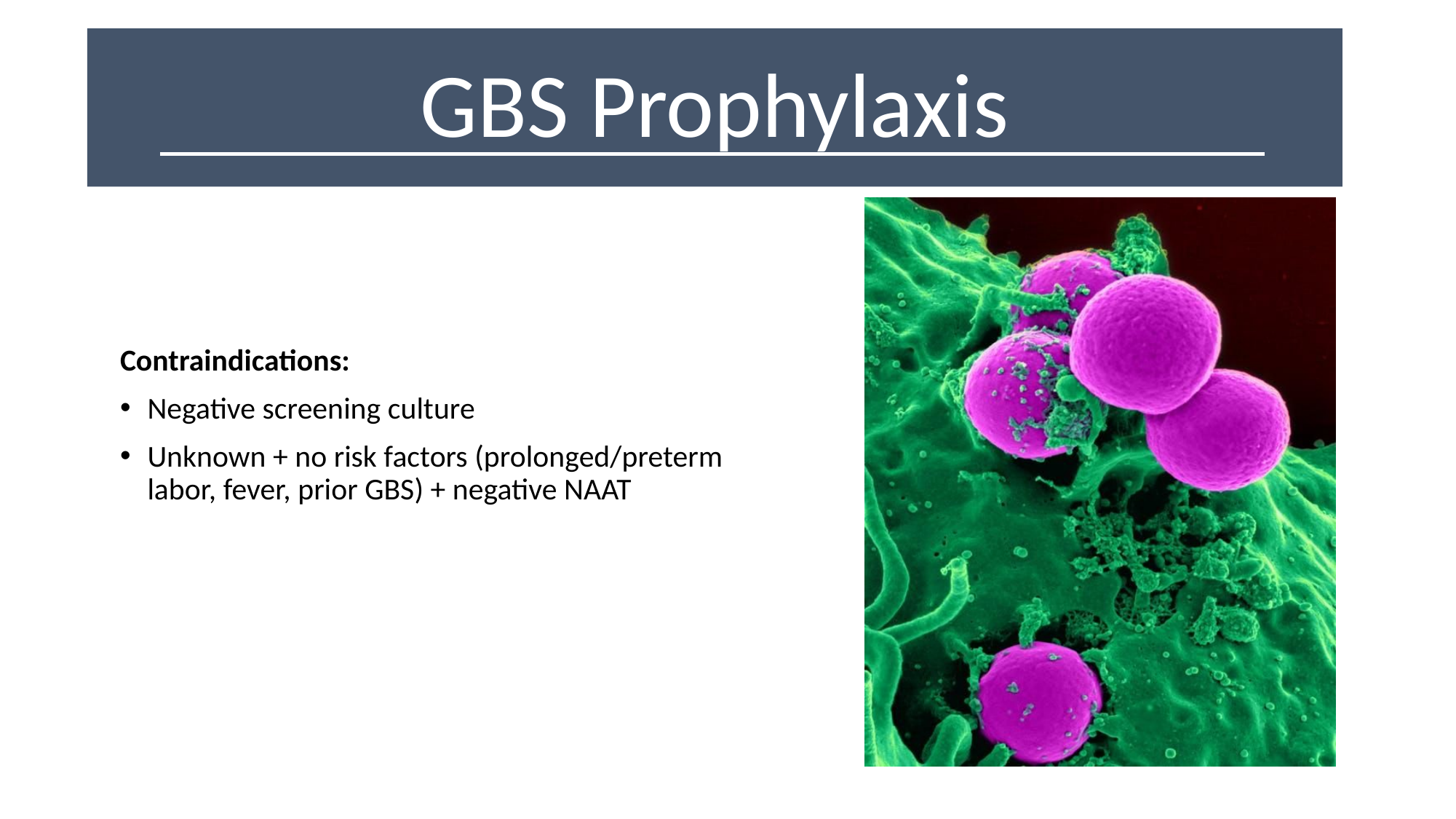

GBS Prophylaxis
Contraindications:
Negative screening culture
Unknown + no risk factors (prolonged/preterm labor, fever, prior GBS) + negative NAAT

## Slide 9
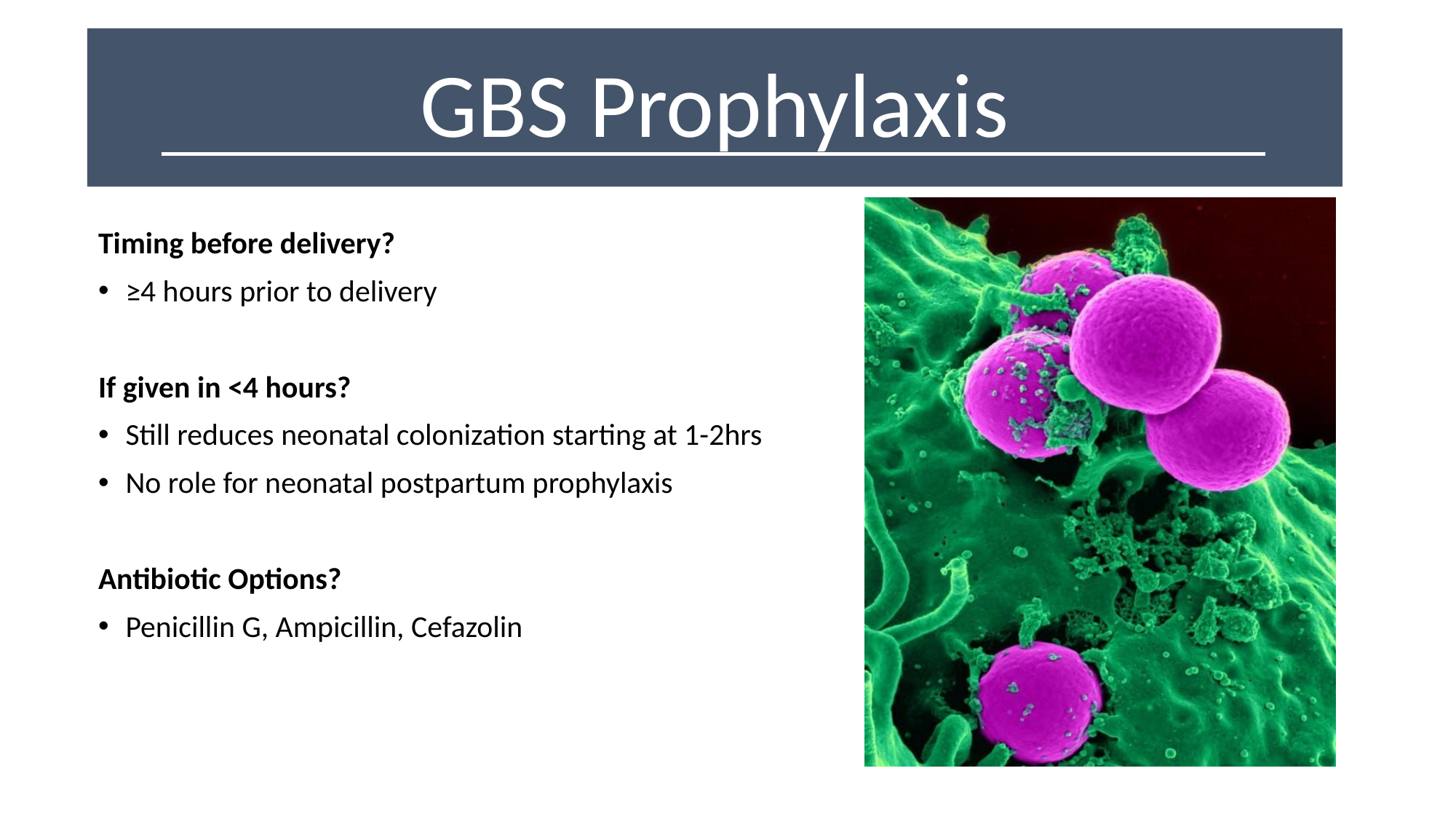

GBS Prophylaxis
Timing before delivery?
≥4 hours prior to delivery
If given in <4 hours?
Still reduces neonatal colonization starting at 1-2hrs
No role for neonatal postpartum prophylaxis
Antibiotic Options?
Penicillin G, Ampicillin, Cefazolin

## Slide 10
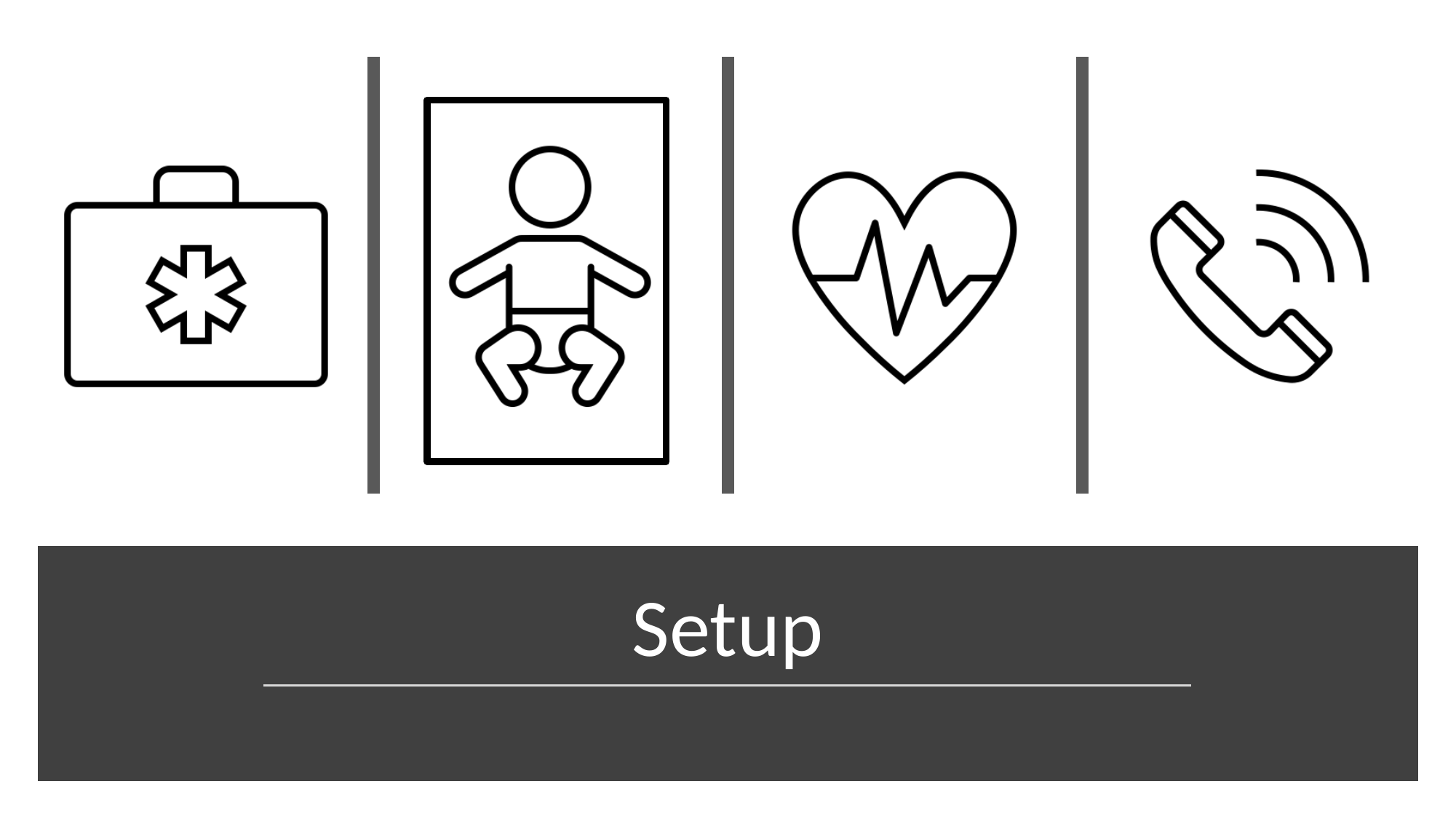

# Setup

## Slide 11
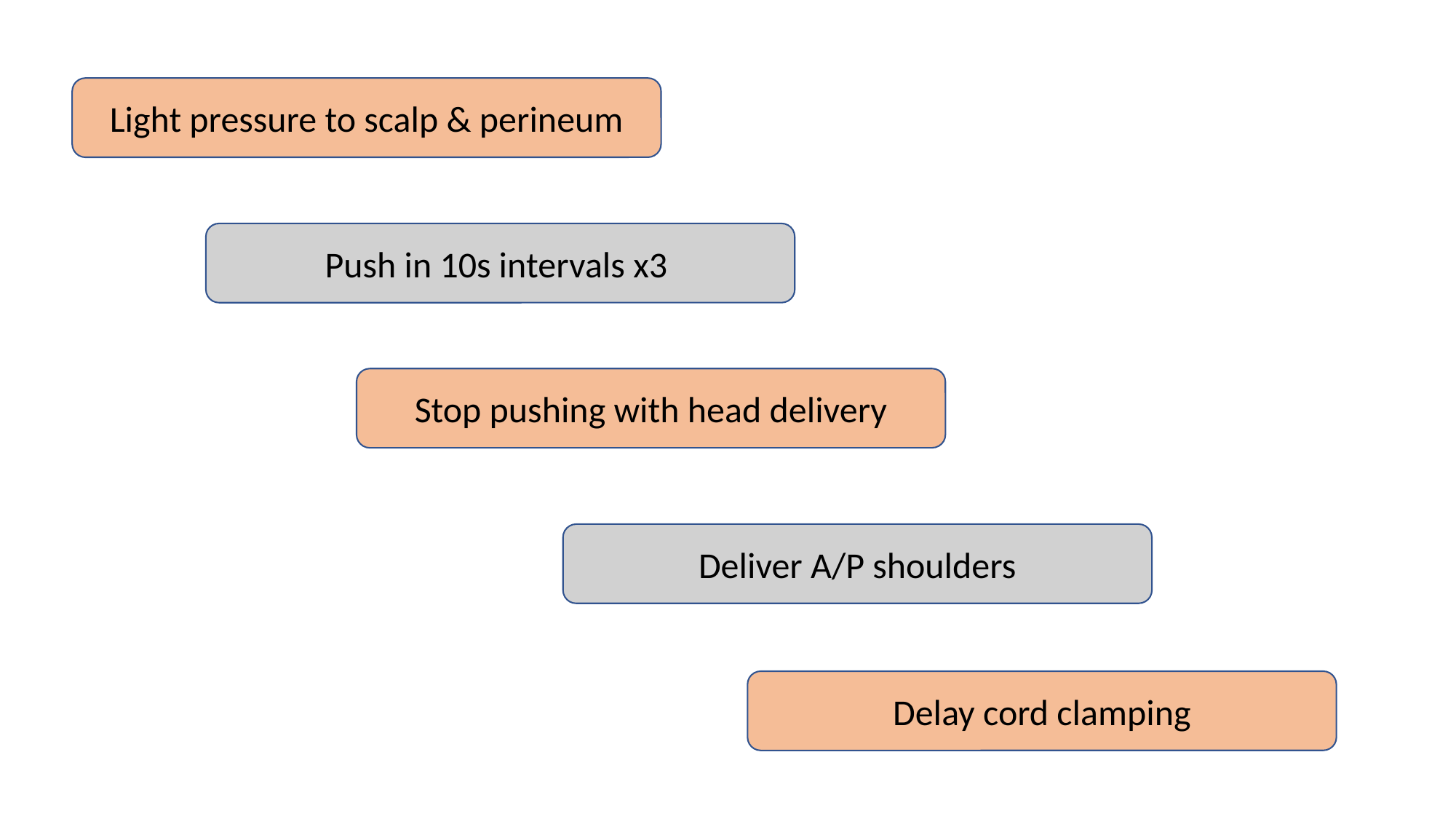

Light pressure to scalp & perineum
Push in 10s intervals x3
Stop pushing with head delivery
Deliver A/P shoulders
Delay cord clamping

## Slide 12
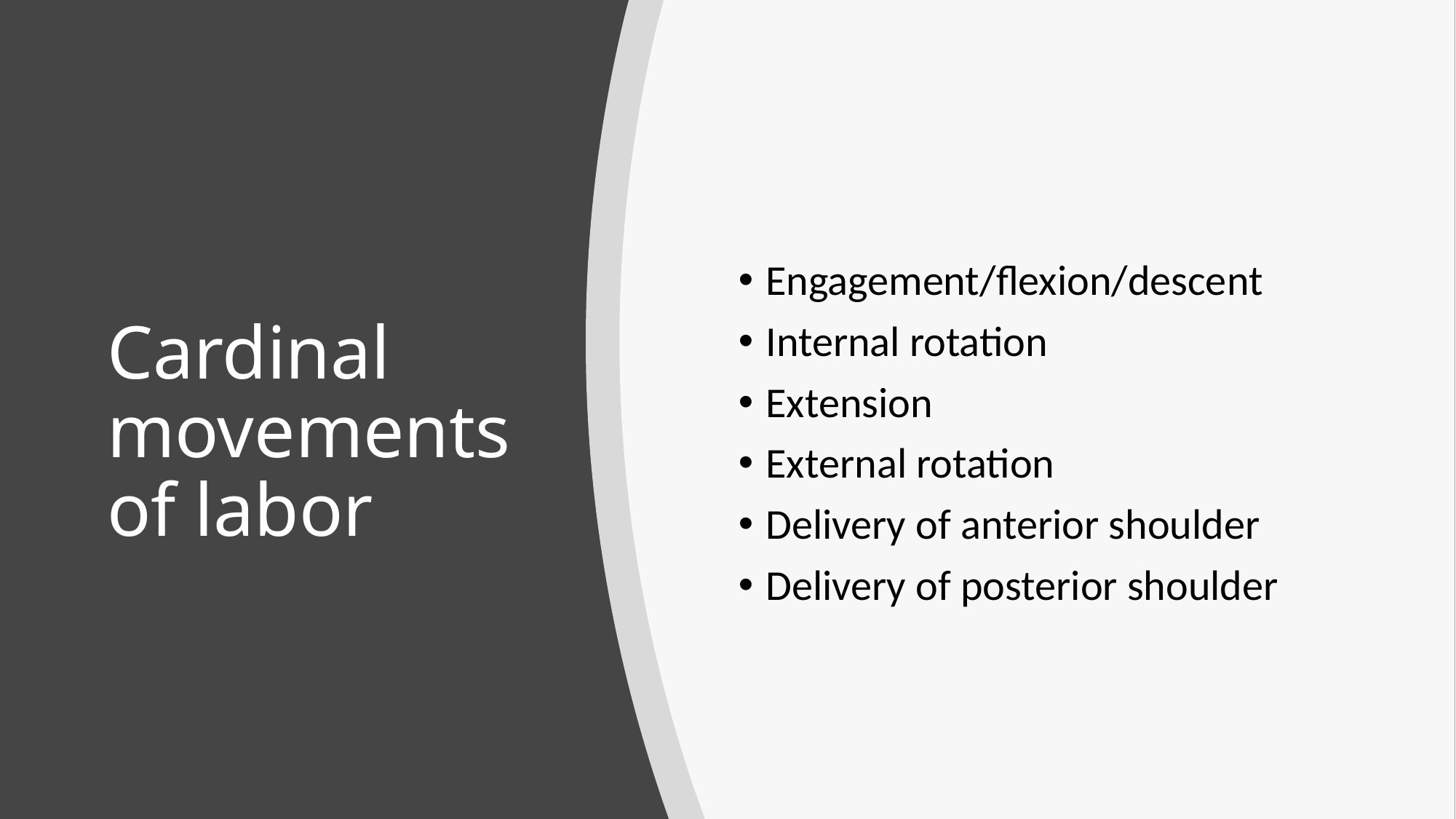

Engagement/flexion/descent
Internal rotation
Extension
External rotation
Delivery of anterior shoulder
Delivery of posterior shoulder
# Cardinal movements of labor

## Slide 13
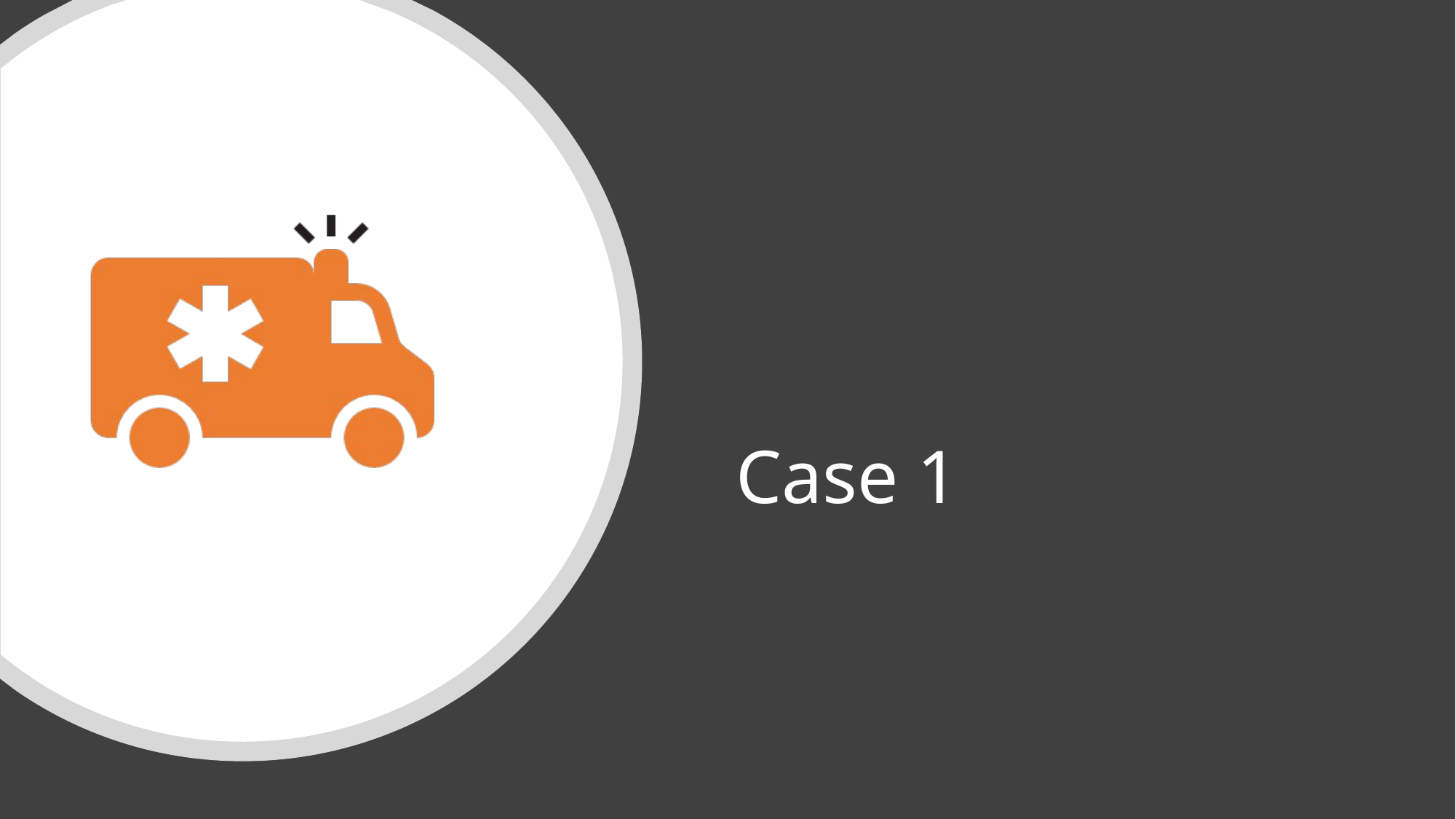

# Case 1

## Slide 14
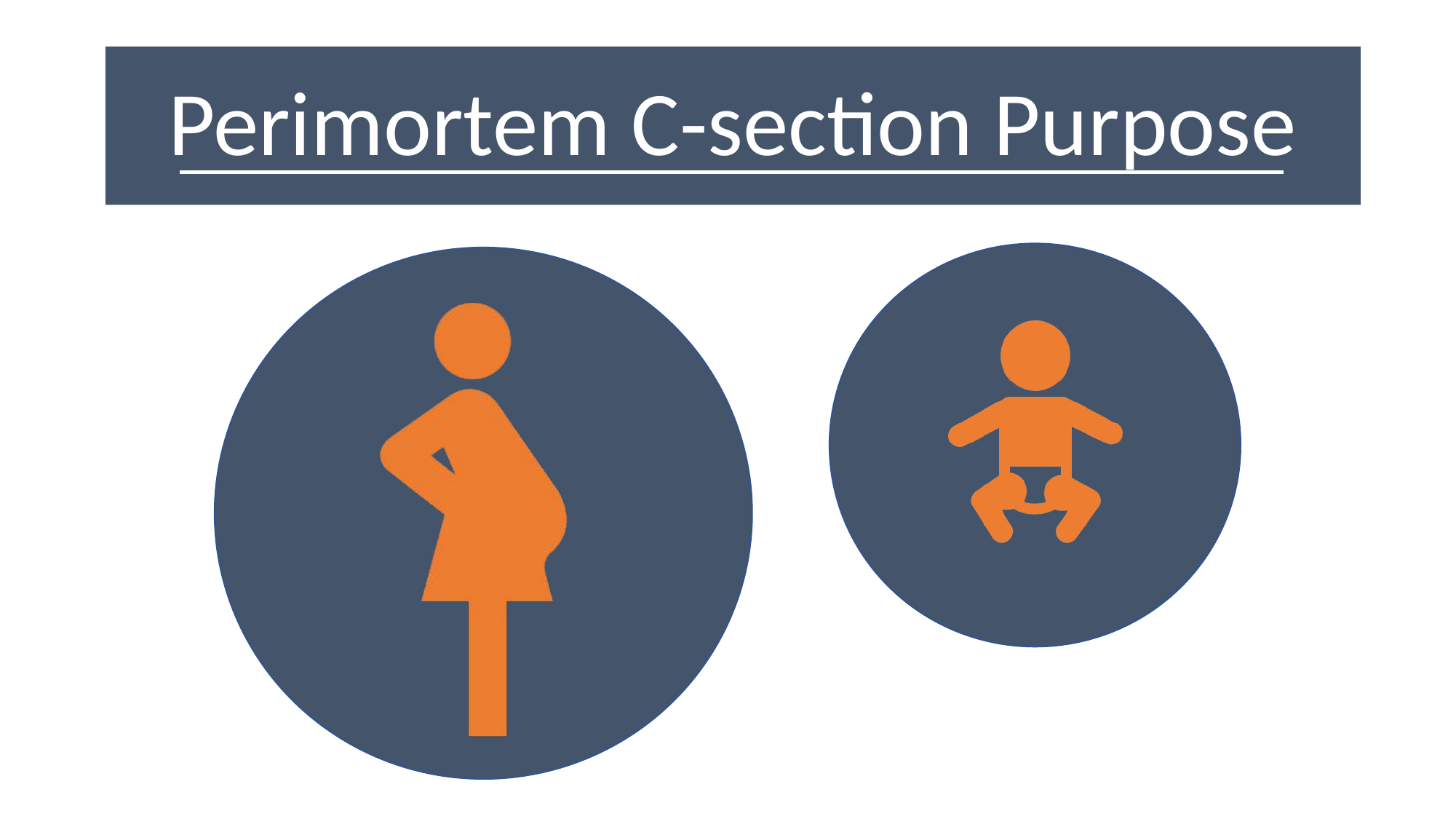

Perimortem C-section Purpose

## Slide 15
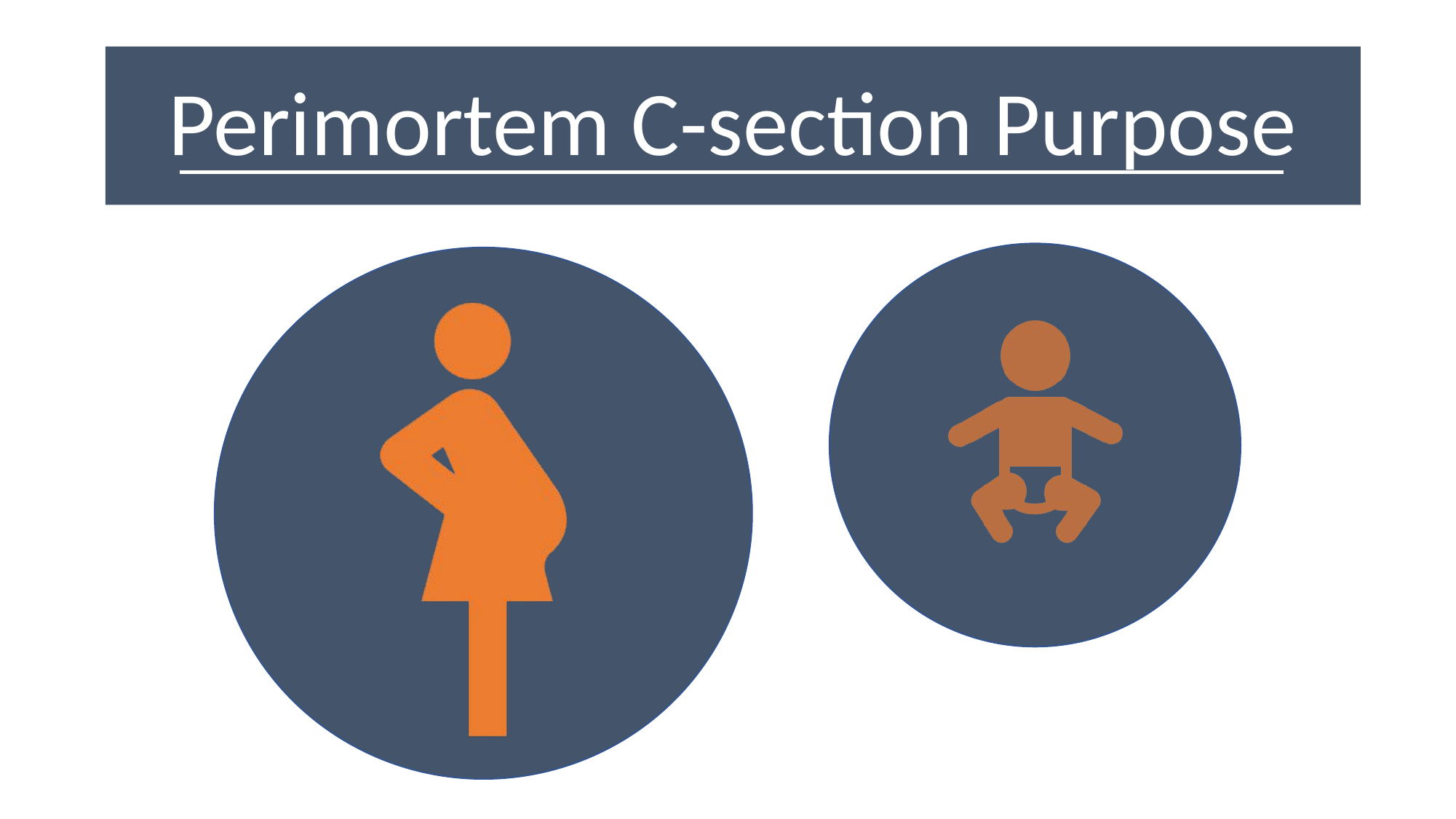

Perimortem C-section Purpose

## Slide 16
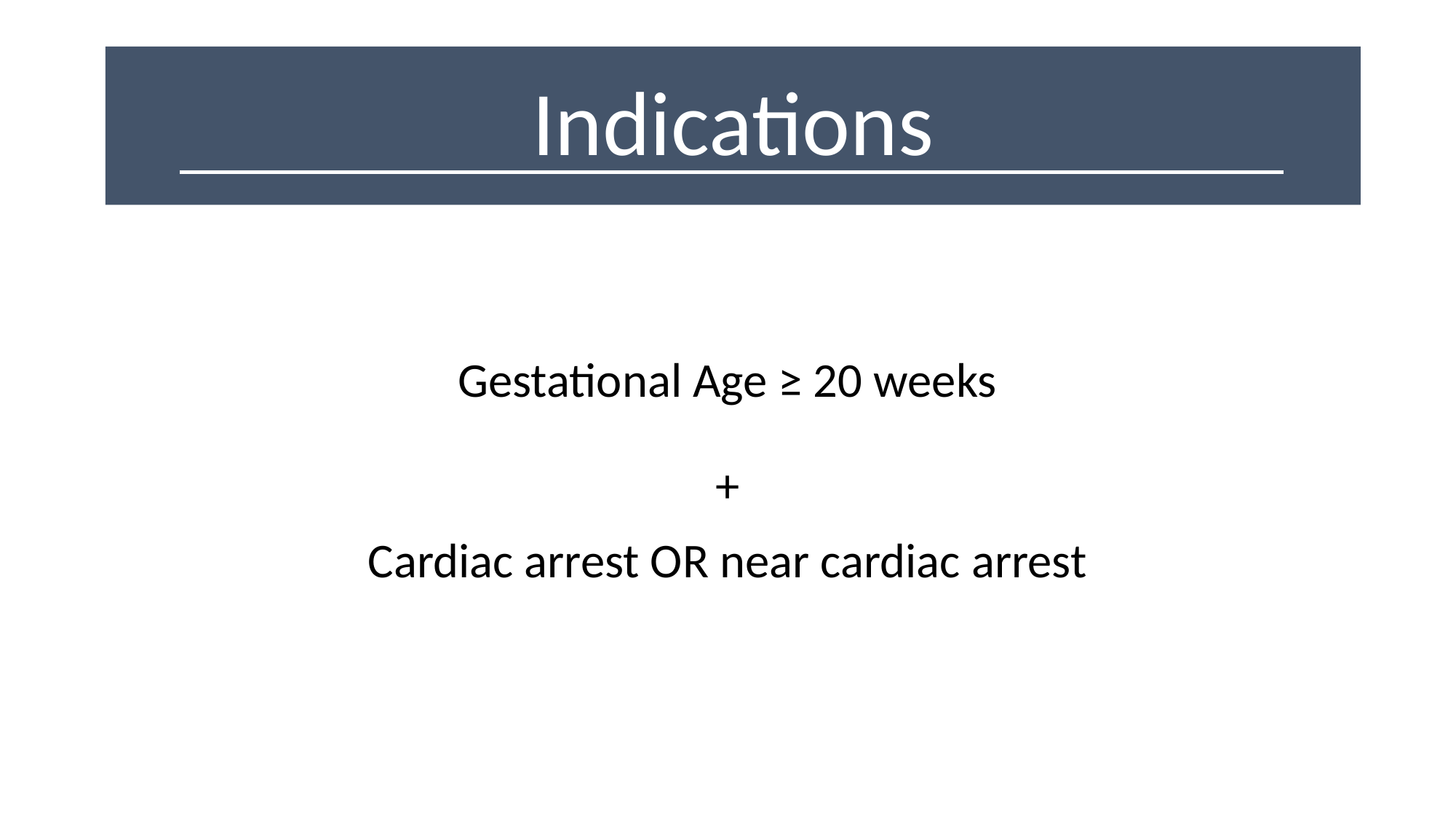

Indications
Gestational Age ≥ 20 weeks
+
Cardiac arrest OR near cardiac arrest

## Slide 17
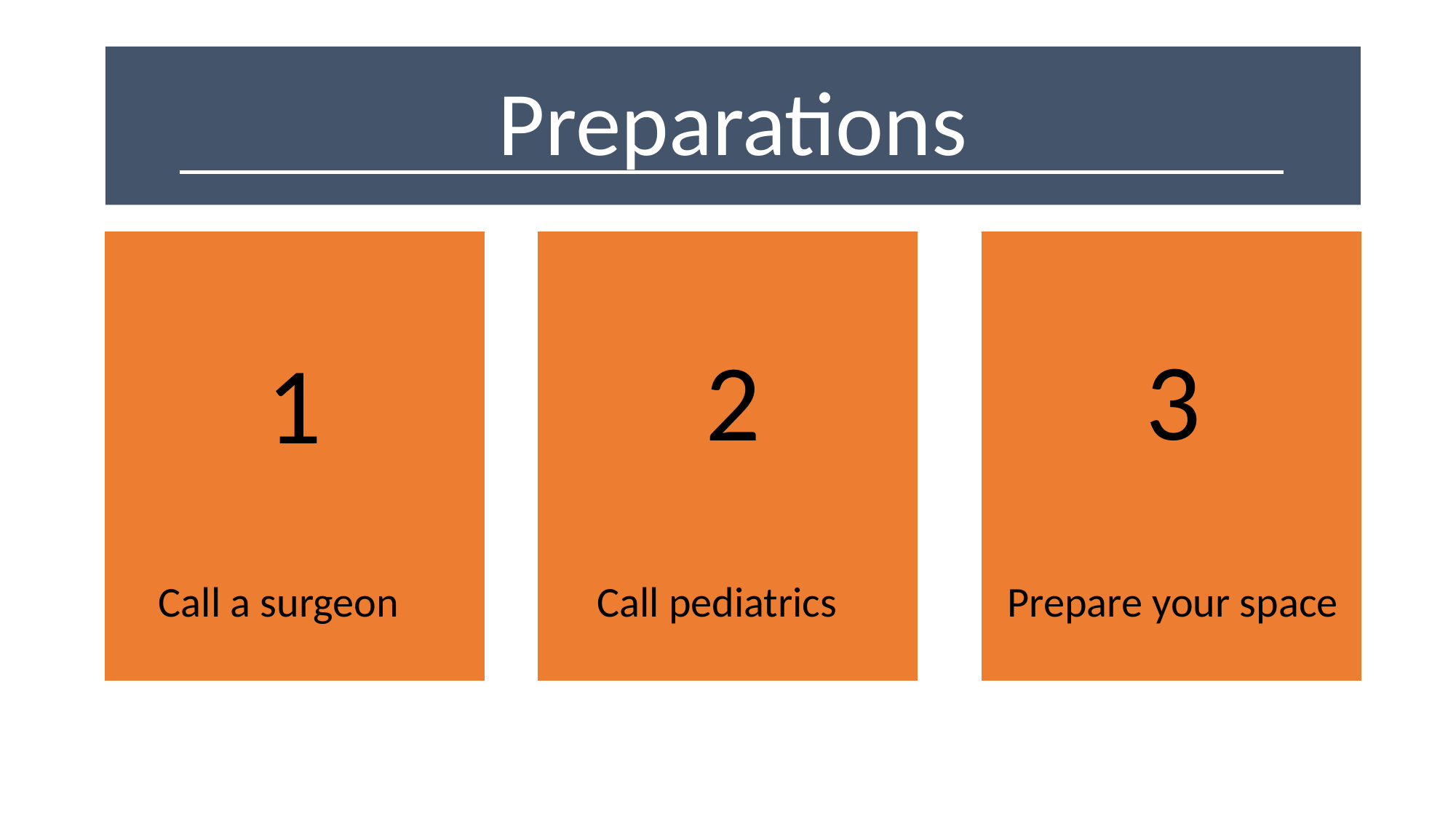

Preparations
3
2
1
Call a surgeon
Call pediatrics
Prepare your space

## Slide 18
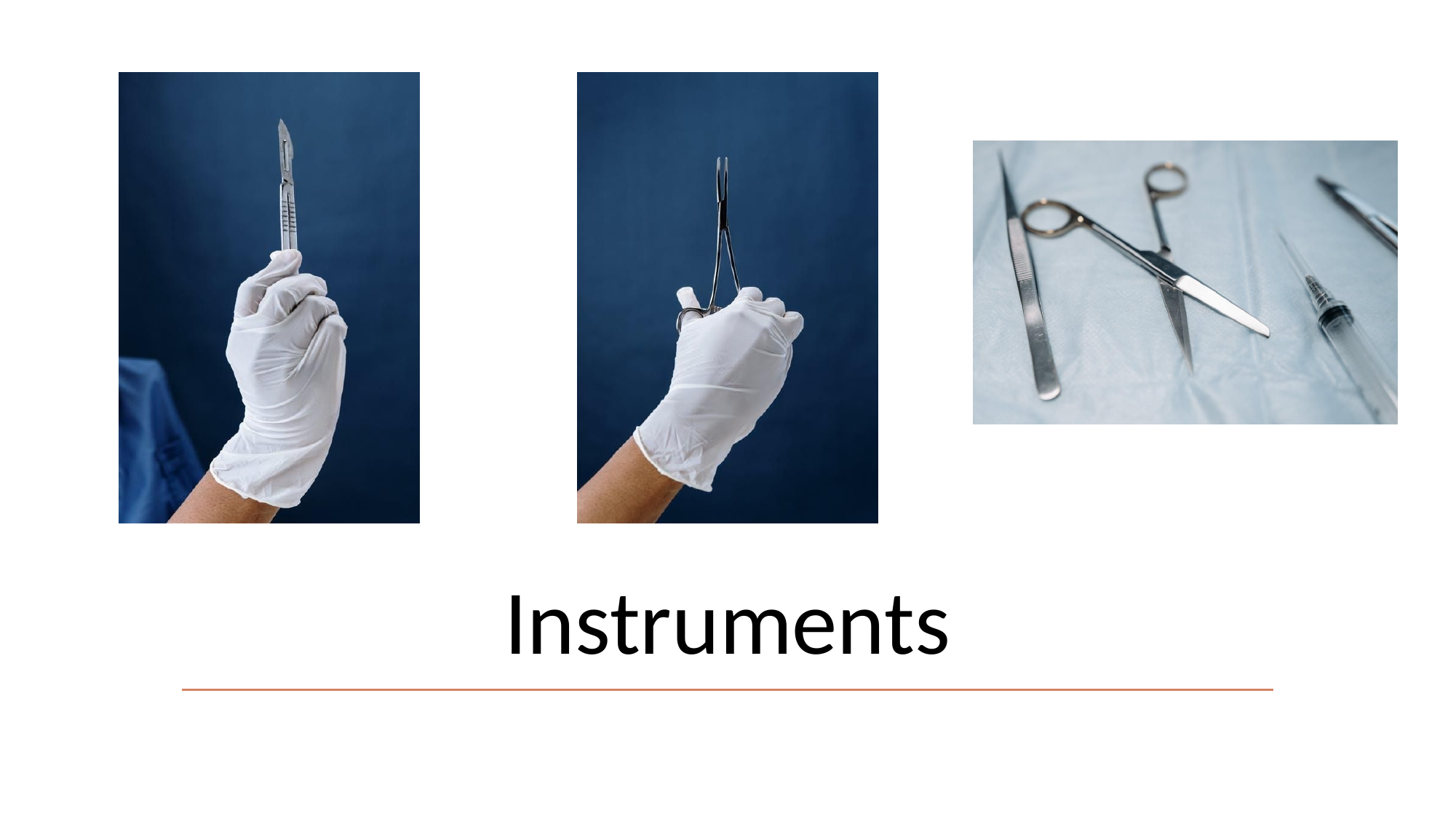

# Instruments

## Slide 19
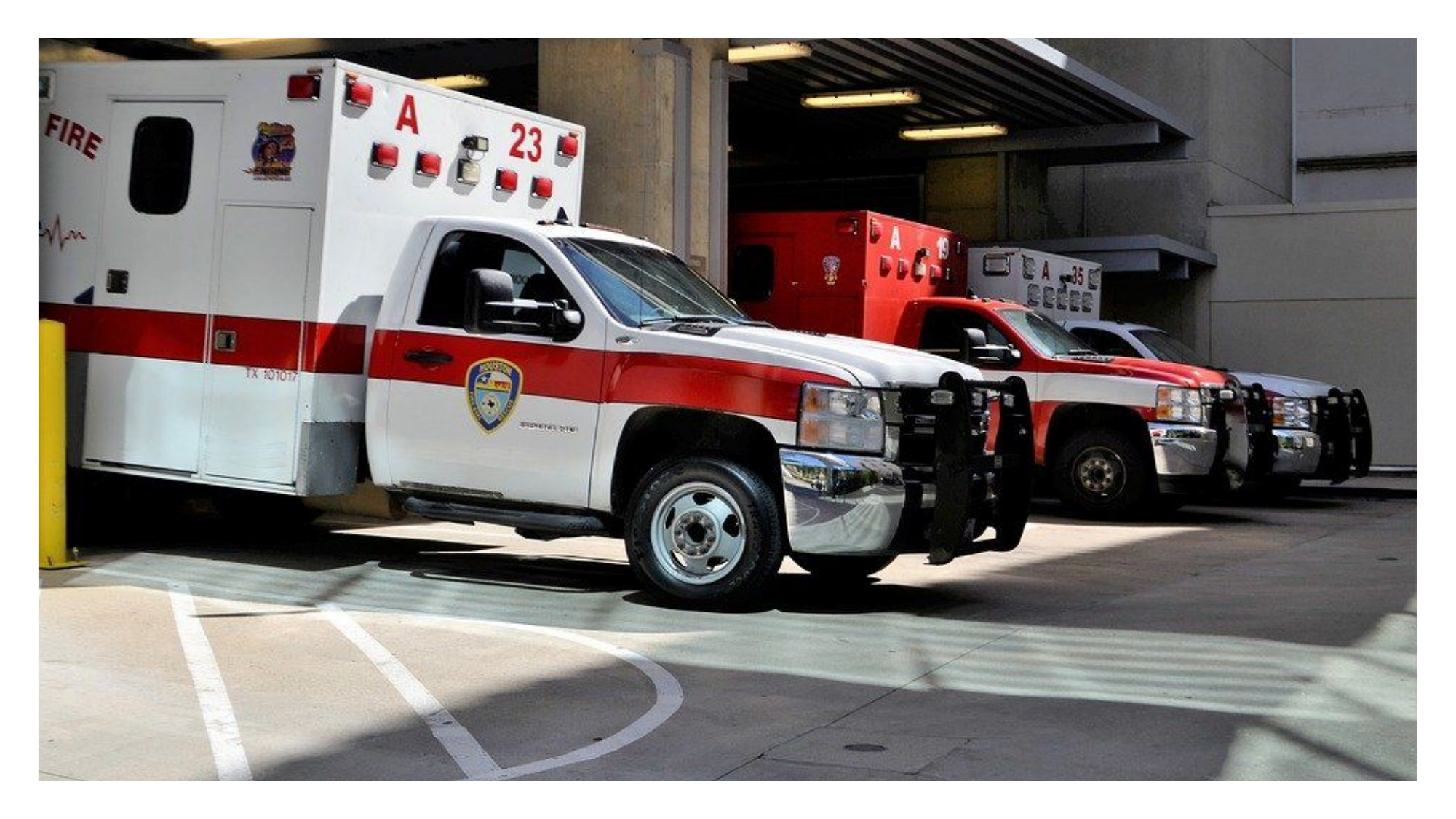

## Slide 20
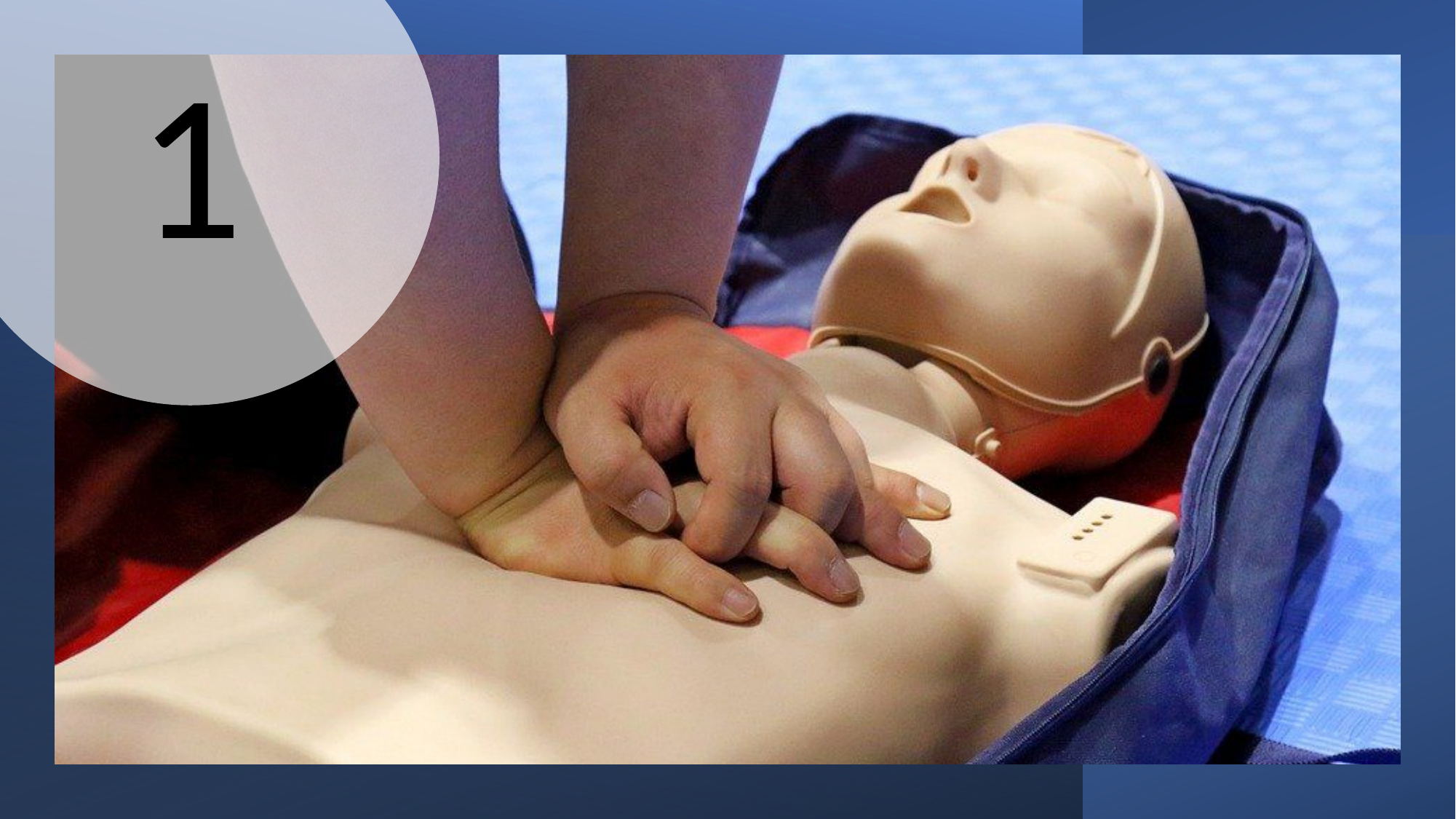

1

## Slide 21
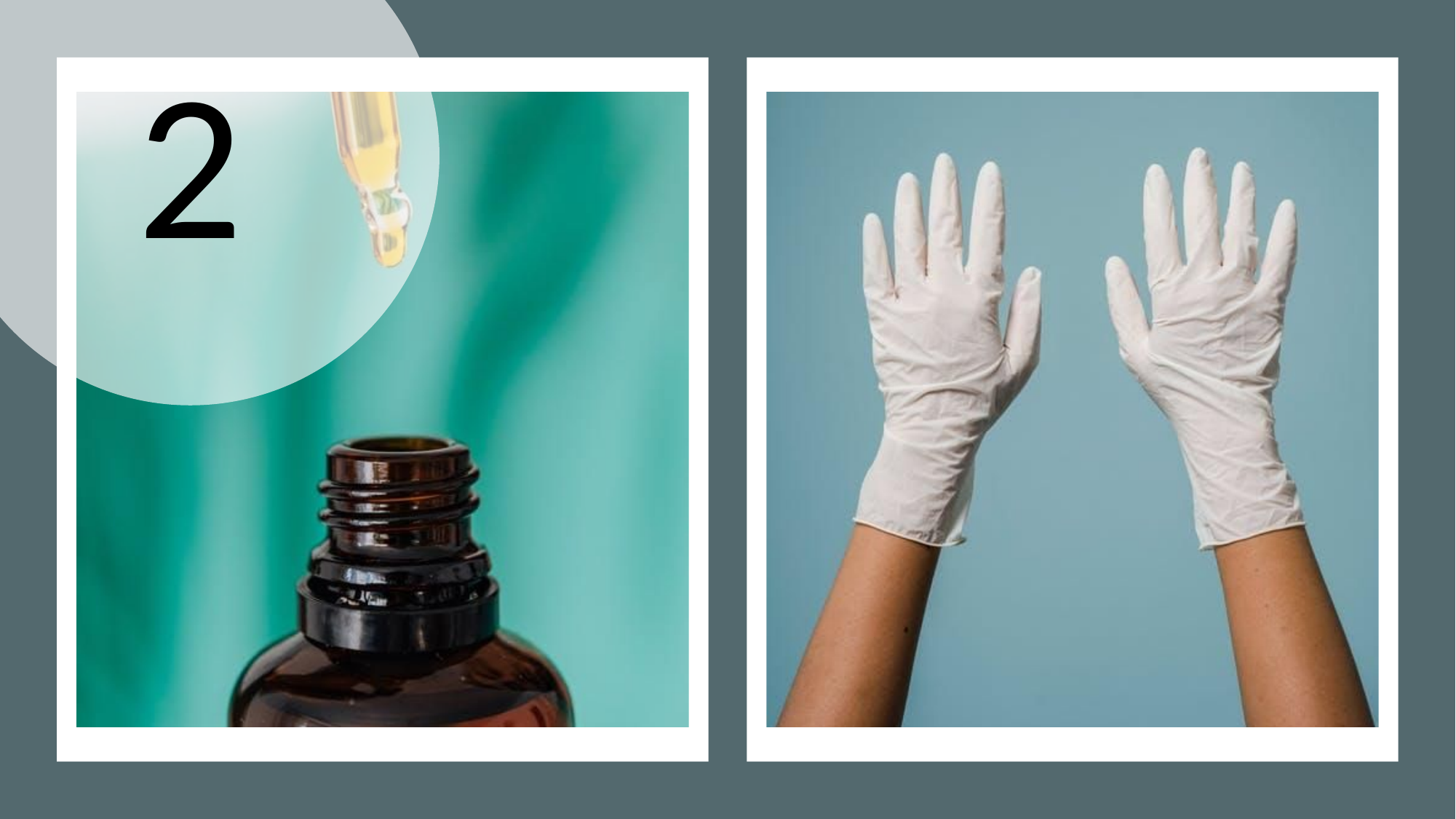

2

## Slide 22
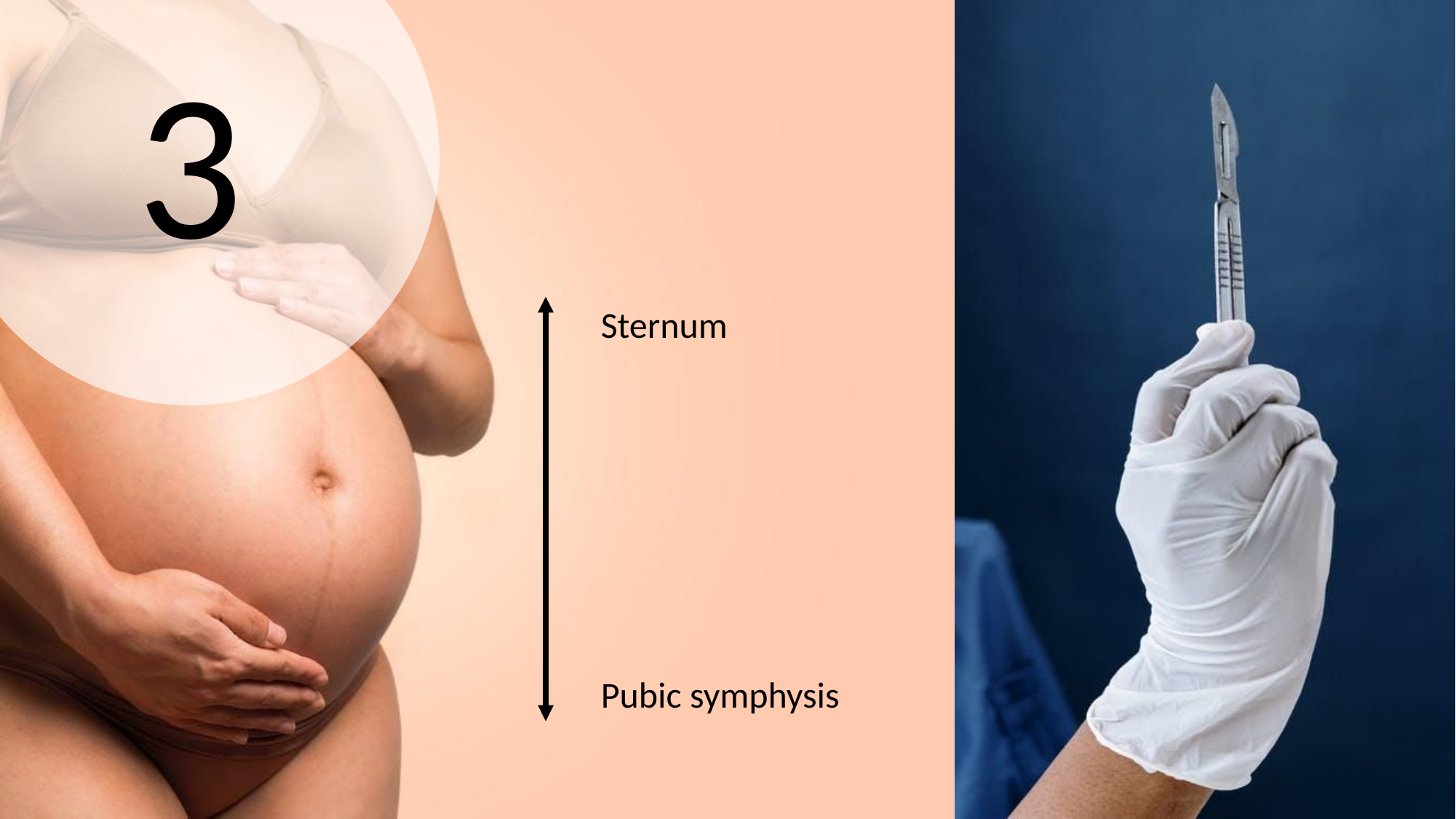

3
Sternum
Pubic symphysis

## Slide 23
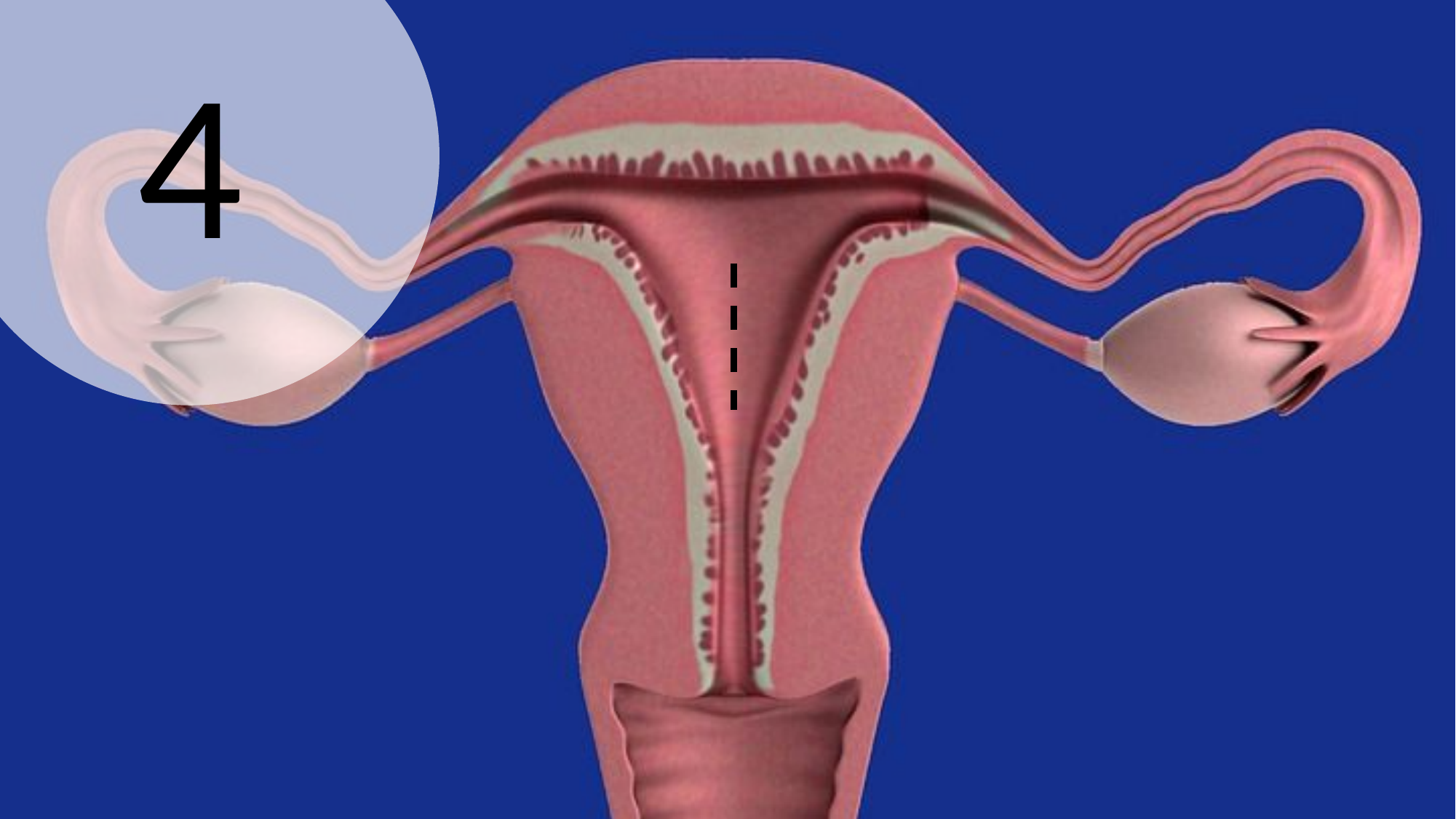

4

## Slide 24
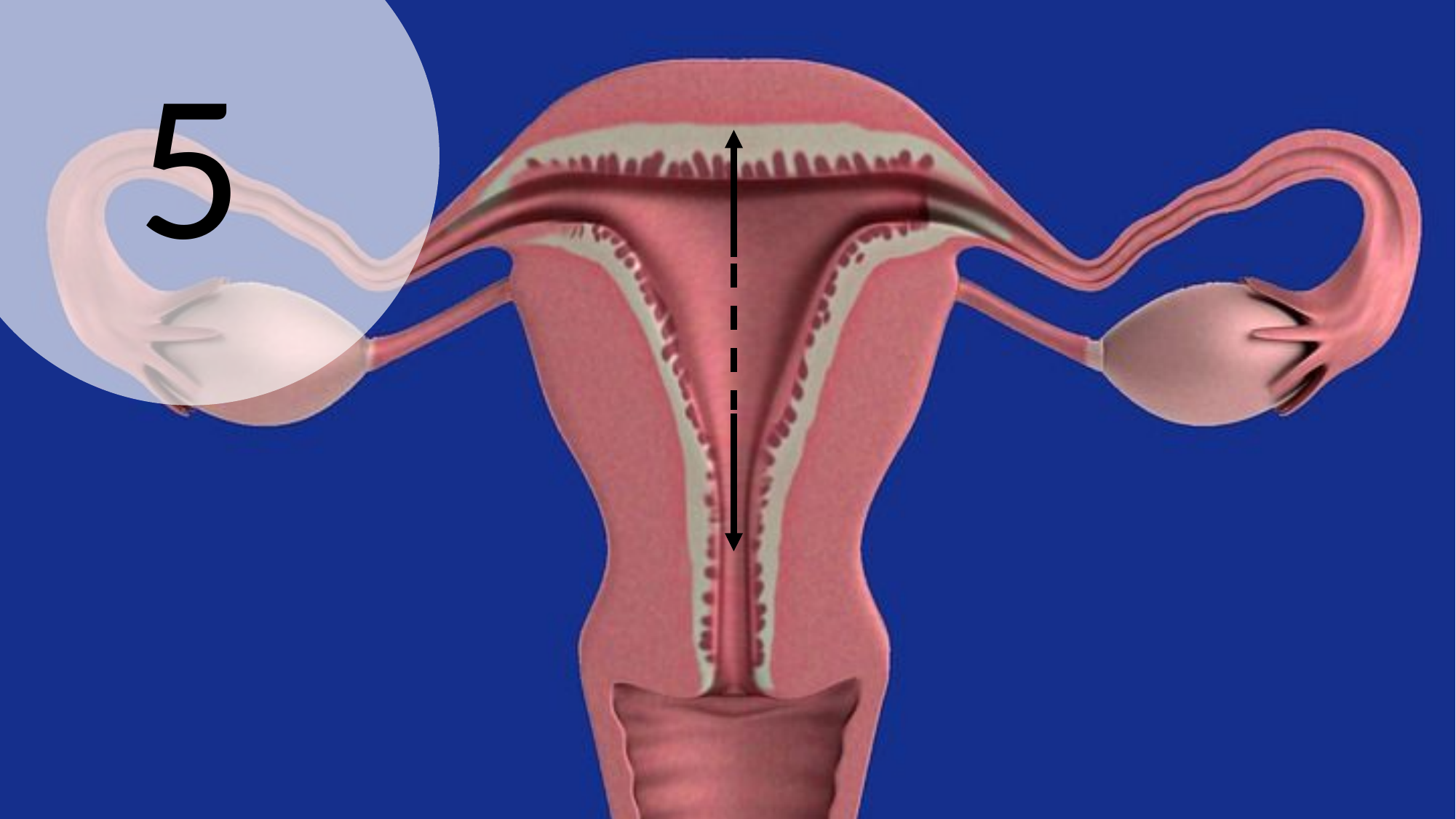

5

## Slide 25
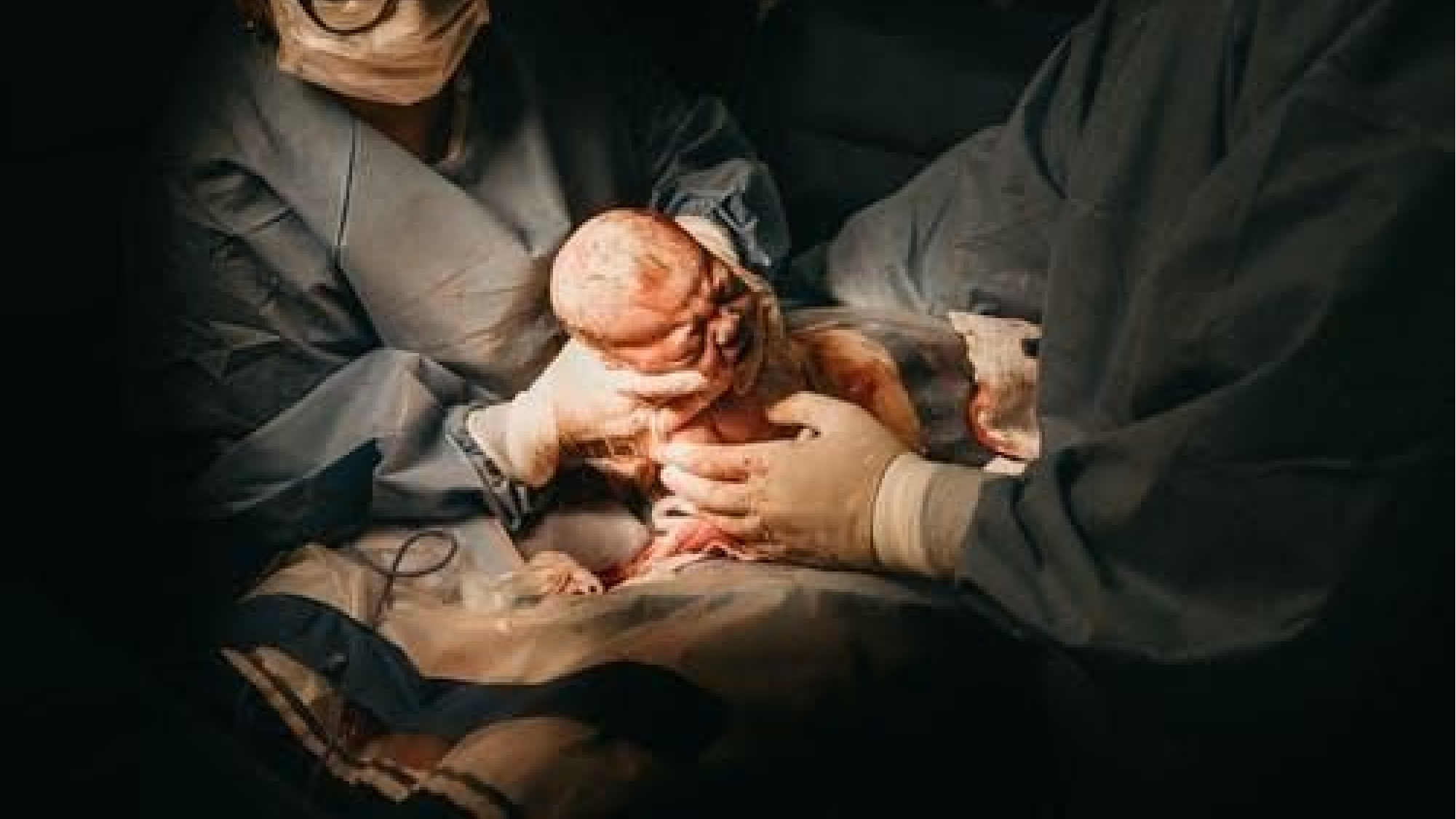

## Slide 26
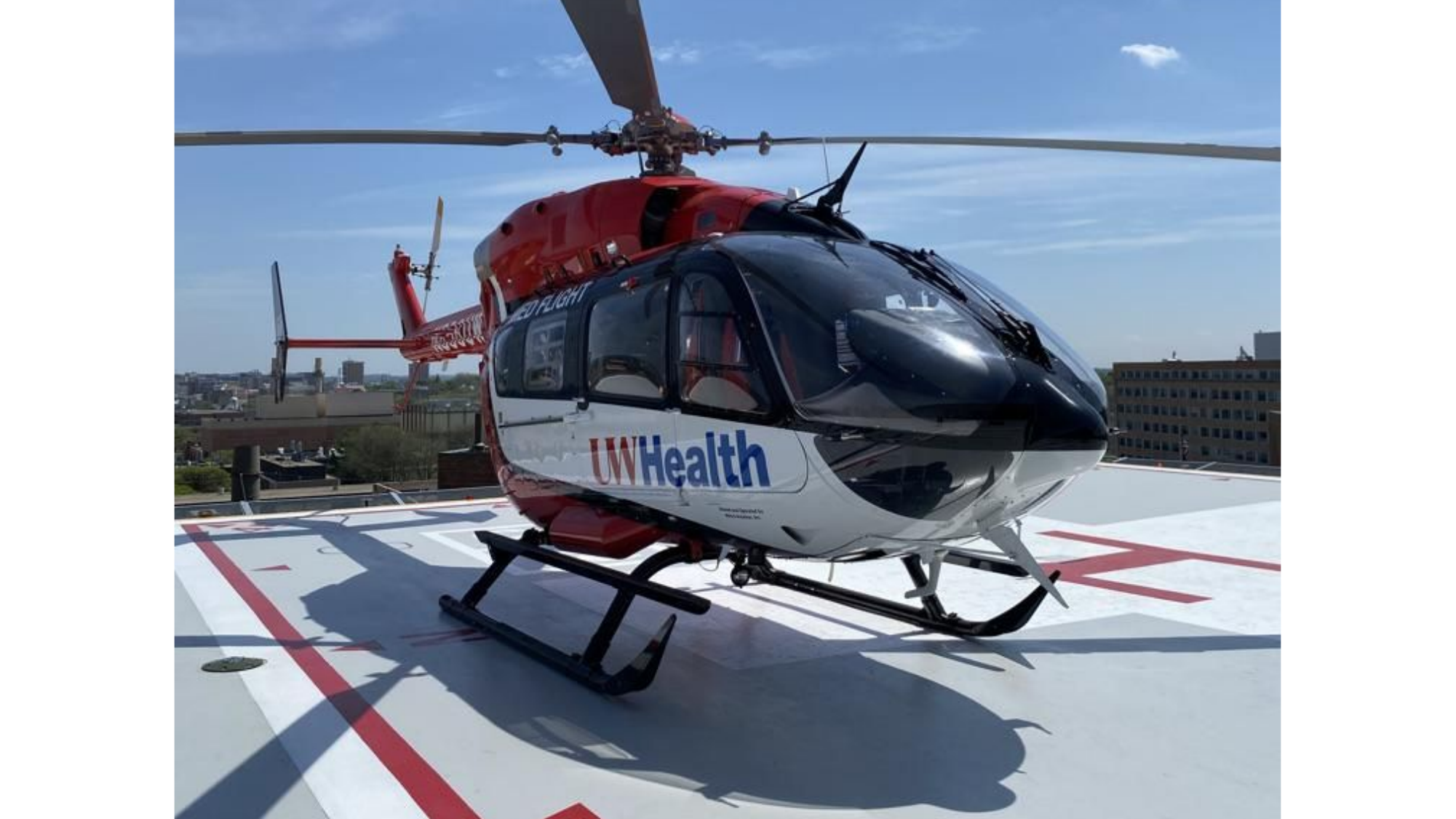

## Slide 27
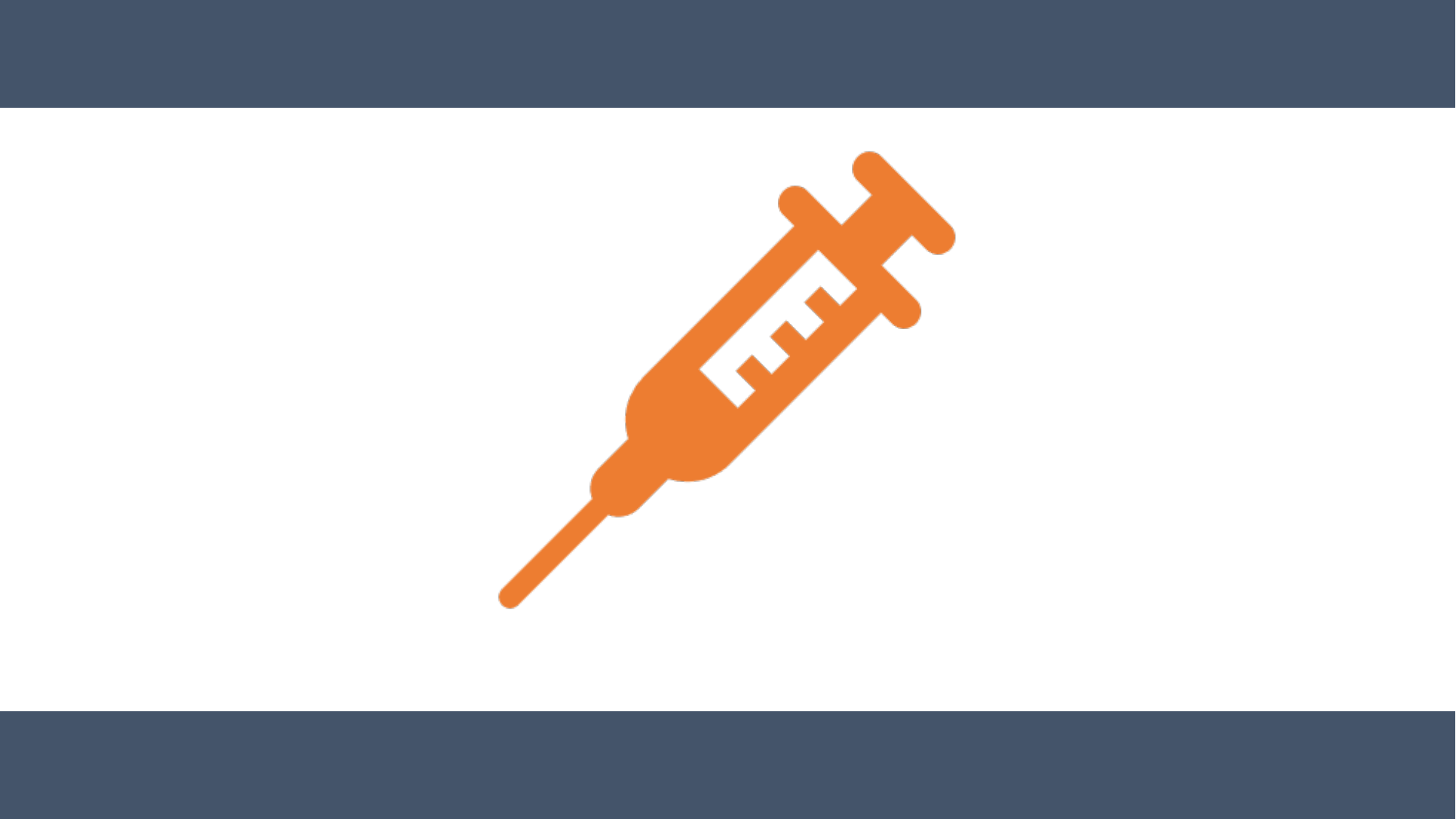

## Slide 28
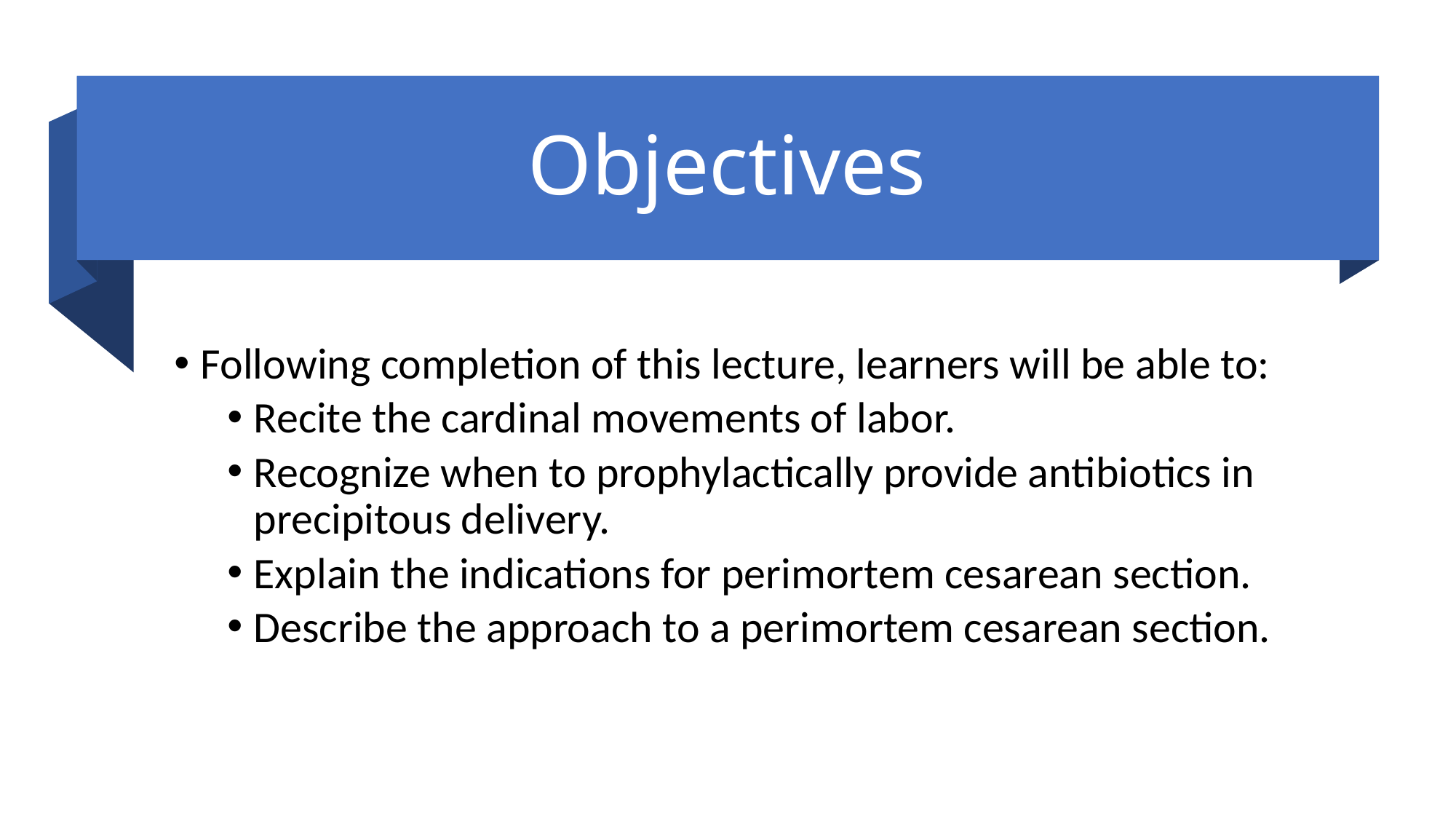

# Objectives
Following completion of this lecture, learners will be able to:
Recite the cardinal movements of labor.
Recognize when to prophylactically provide antibiotics in precipitous delivery.
Explain the indications for perimortem cesarean section.
Describe the approach to a perimortem cesarean section.

## Slide 29
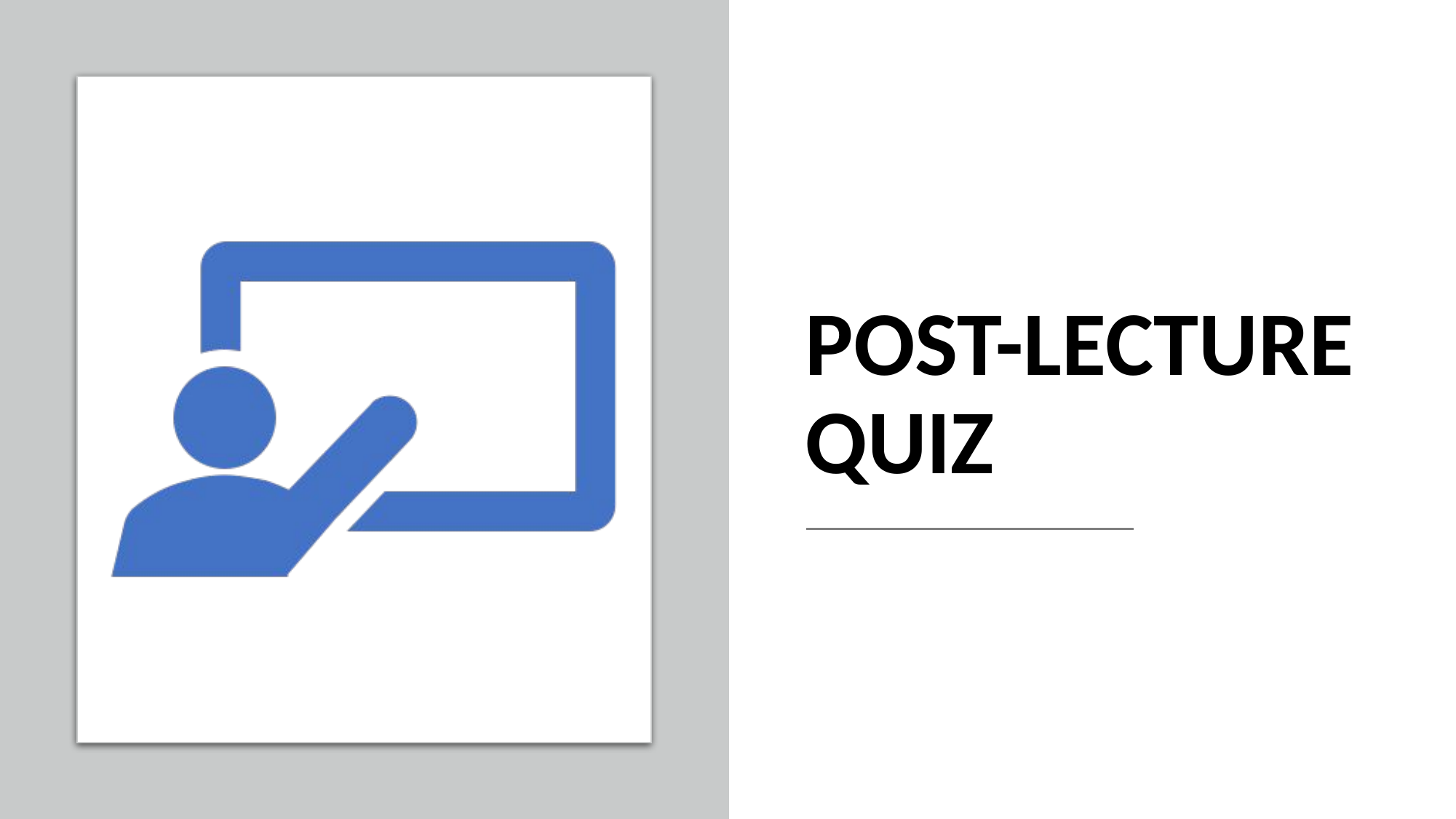

# POST-LECTURE QUIZ

## Slide 30
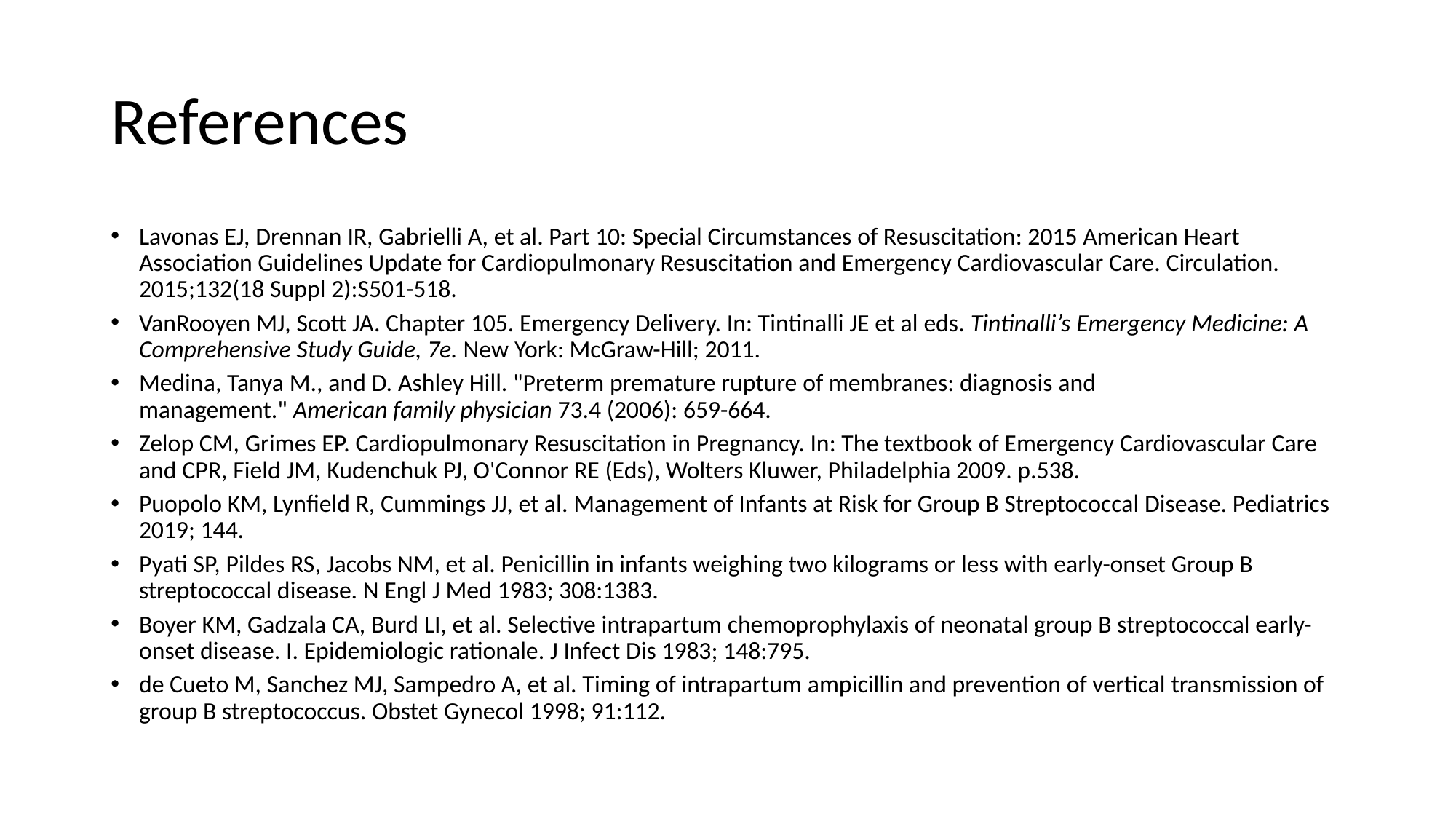

# References
Lavonas EJ, Drennan IR, Gabrielli A, et al. Part 10: Special Circumstances of Resuscitation: 2015 American Heart Association Guidelines Update for Cardiopulmonary Resuscitation and Emergency Cardiovascular Care. Circulation. 2015;132(18 Suppl 2):S501-518.
VanRooyen MJ, Scott JA. Chapter 105. Emergency Delivery. In: Tintinalli JE et al eds. Tintinalli’s Emergency Medicine: A Comprehensive Study Guide, 7e. New York: McGraw-Hill; 2011.
Medina, Tanya M., and D. Ashley Hill. "Preterm premature rupture of membranes: diagnosis and management." American family physician 73.4 (2006): 659-664.
Zelop CM, Grimes EP. Cardiopulmonary Resuscitation in Pregnancy. In: The textbook of Emergency Cardiovascular Care and CPR, Field JM, Kudenchuk PJ, O'Connor RE (Eds), Wolters Kluwer, Philadelphia 2009. p.538.
Puopolo KM, Lynfield R, Cummings JJ, et al. Management of Infants at Risk for Group B Streptococcal Disease. Pediatrics 2019; 144.
Pyati SP, Pildes RS, Jacobs NM, et al. Penicillin in infants weighing two kilograms or less with early-onset Group B streptococcal disease. N Engl J Med 1983; 308:1383.
Boyer KM, Gadzala CA, Burd LI, et al. Selective intrapartum chemoprophylaxis of neonatal group B streptococcal early-onset disease. I. Epidemiologic rationale. J Infect Dis 1983; 148:795.
de Cueto M, Sanchez MJ, Sampedro A, et al. Timing of intrapartum ampicillin and prevention of vertical transmission of group B streptococcus. Obstet Gynecol 1998; 91:112.
