# Supplementary material for: Emergency Medicine Obstetrics and Gynecology: A Case-Based Curriculum for Residents
Source: MedEdPORTAL. 2023 Aug 11;19:11330. doi: 10.15766/mep_2374-8265.11330 (PMC10415535; doi:10.15766/mep_2374-8265.11330)
Supplement: Supplementary file 1 — Ectopic Pregnancy and Emergencies in the First 20 Weeks.pptxPregnancy Emergencies After 20 Weeks.pptxDelivery Emergencies.pptxPelvic Pain in the Nonpregnant Patient.pptxVaginitis, Cervicitis, and PID.pptxAbnormal Uterine Bleeding.pptxLabor and Perimortem C-Section.pptxSession Review Questions.docxPrecurriculum Survey.docxPostcurriculum Survey.docx [file mep_2374-8265.11330-s001.zip › I. Precurriculum Survey.docx]

**Pre-Curriculum Survey**

We wrote Q1 and Q2 to create anonymous identifiers for the residents to help with the research component of the project. These questions were made to match the residents pre- and post-curriculum without giving away their identities. Any type of unique identifying questions could be used if preferred over the following.

Q1: What is your mother’s maiden name?

Q2: If you live in an apartment, please provide your apartment number (not building address number). If you live in a house, please provide your house number.

Q3: Please circle your answer to the following: I feel confident in my ability to manage…

Ectopic pregnancy

Strongly disagree, somewhat disagree, neither agree nor disagree, somewhat agree, strongly agree

Spontaneous abortion

Strongly disagree, somewhat disagree, neither agree nor disagree, somewhat agree, strongly agree

Preeclampsia

Strongly disagree, somewhat disagree, neither agree nor disagree, somewhat agree, strongly agree

Preterm premature rupture of membranes

Strongly disagree, somewhat disagree, neither agree nor disagree, somewhat agree, strongly agree

Postpartum hemorrhage

Strongly disagree, somewhat disagree, neither agree nor disagree, somewhat agree, strongly agree

Shoulder dystocia

Strongly disagree, somewhat disagree, neither agree nor disagree, somewhat agree, strongly agree

Abnormal uterine bleeding in a non-pregnant female

Strongly disagree, somewhat disagree, neither agree nor disagree, somewhat agree, strongly agree

Vaginitis/cervicitis

Strongly disagree, somewhat disagree, neither agree nor disagree, somewhat agree, strongly agree

Pelvic inflammatory disease

Strongly disagree, somewhat disagree, neither agree nor disagree, somewhat agree, strongly agree

Ovarian hyperstimulation syndrome

Strongly disagree, somewhat disagree, neither agree nor disagree, somewhat agree, strongly agree

Ovarian torsion

Strongly disagree, somewhat disagree, neither agree nor disagree, somewhat agree, strongly agree
