# Supplementary material for: Emergency Medicine Obstetrics and Gynecology: A Case-Based Curriculum for Residents
Source: MedEdPORTAL. 2023 Aug 11;19:11330. doi: 10.15766/mep_2374-8265.11330 (PMC10415535; doi:10.15766/mep_2374-8265.11330)
Supplement: Supplementary file 1 — Ectopic Pregnancy and Emergencies in the First 20 Weeks.pptxPregnancy Emergencies After 20 Weeks.pptxDelivery Emergencies.pptxPelvic Pain in the Nonpregnant Patient.pptxVaginitis, Cervicitis, and PID.pptxAbnormal Uterine Bleeding.pptxLabor and Perimortem C-Section.pptxSession Review Questions.docxPrecurriculum Survey.docxPostcurriculum Survey.docx [file mep_2374-8265.11330-s001.zip › _Educational Summary Report_11330.pdf]

MedEdPORTAL® The AAMC Journal of Teaching and Learning Resources

Original Publication Open Access

Emergency Medicine Obstetrics and Gynecology: A Case-Based Curriculum for Residents

Shannon M. Burke, MD*, Thaddeus Schmitt, MD, Polly Kennedy, MD, Brittany Kotek, MD, James N. Wolfe, MD, Corlin Jewell, MD, Kaitlin A. Ray, MD, Benjamin H. Schnapp, MD, MEd

*Corresponding author: smburke@mcw.edu

Abstract

Introduction: Millions of patients present to US emergency departments every year with OB/GYN concerns. Emergency medicine trainees must be adequately prepared to care for this population, regardless of how commonly they appear in the training environment. We used active learning and gamification principles in this curriculum to increase learner engagement and participation in the material. **Methods:** We chose OB/GYN topics based on review of Tintinalli's OB/GYN content and the American Board of Emergency Medicine's Model of Clinical Practice. Each session comprised a case-based lecture and review questions using the game-based Kahoot! online software. Pre- and postcurriculum surveys assessed residents' confidence in caring for emergent OB/GYN pathologies on a 5-point Likert scale. We designed survey questions assessing the first level of Kirkpatrick's levels of training evaluation; these questions were reviewed and revised by the department's Medical Education Scholarship Committee for validity. **Results:** A mean of 18 residents attended each session. Seventy-six percent of residents (26 of 34) completed the precurriculum survey, 67% (23 of 34) completed the postcurriculum survey, and 44% (15 of 34) completed both. For all respondents, mean reported confidence with curriculum topics increased from 3.5 to 4.0 ($p < .05$). For residents completing both surveys, confidence increased from 3.4 to 4.0 ($p < .01$). **Discussion:** Application of this curriculum significantly improved learner confidence in targeted OB/GYN topics. Future directions could include evaluating curricular impact at higher levels in the Kirkpatrick model, extending sessions to include more time for interaction, and adding suggested readings.

Keywords
Active Learning, Spaced Repetition, Cased-Based Learning, Clinical Teaching/Bedside Teaching, Emergency Medicine, OB/GYN

Educational Objectives

Following completion of this curriculum, resident physicians will be able to:

1. Identify common OB/GYN pathologies presenting to the emergency department (ED).
2. Outline appropriate diagnostic evaluations for OB/GYN pathologies in the ED.
3. Describe the appropriate management of OB/GYN pathologies in the ED.

Citation:
Burke SM, Schmitt T, Kennedy P, et al. Emergency medicine obstetrics and gynecology: a case-based curriculum for residents. *MedEdPORTAL*. 2023;19:11330.
https://doi.org/10.15766/mep_2374-8265.11330

Introduction

In 2018, there were approximately 3,276,000 visits to emergency departments (EDs) across the country for obstetric or gynecologic (OB/GYN) conditions.[1] Thus, graduates of emergency medicine (EM) residencies must be suitably trained to care for and manage the wide variety of OB/GYN emergencies. However, depending on their clinical environment, residents may not see a sufficient number of OB/GYN encounters to become fully comfortable providing care for patients with emergent OB/GYN complaints. Therefore, this content must be delivered to residents by other means to attain a necessary level of competence to both pass the American Board of Emergency Medicine Qualifying Examination and successfully practice EM independently.

There have been other attempts at creating a curriculum designed to teach medical trainees OB/GYN topics. Several institutions have designed multiyear women's health fellowships for residency graduates across specialties.[2] However, being

Copyright © 2023 Burke et al. This is an open-access publication distributed under the terms of the Creative Commons Attribution license.

able to manage these complaints is a critical skill for any independently practicing emergency physician and should be addressed during residency. Another *MedEdPORTAL* curriculum addresses many of the same topics but relies solely on asynchronous content.[3] Two additional curricula exist targeting EM residents specifically, but one focuses solely on obstetrical complaints while the other utilizes the flipped classroom model.[4,5]

We developed a series of seven sessions given to an audience of PGY 1-PGY 3 EM residents and incorporating case-based didactics and an online game-based learning platform that address high-yield OB/GYN topics. To our knowledge, this is the first published curriculum combining traditional lecturing with case discussion and competitive question-based gamification in the critical domain of OB/GYN presentations to the ED. We used principles of active learning with the aim of increasing learner engagement and participation as well as retention of material.[6,7] Gamification has become increasingly popular in medical education as a means of encouraging active participation among residents.[8] These combined elements create a focused curriculum that delivers critical content to residents while simultaneously encouraging active engagement.

Methods

The target audience for this curriculum was EM residents at all levels of training. Ideal instructors for this curriculum included attendings and fellows. However, with guidance on delivery of didactics, resident physicians interested in developing their skills as lecturers would also be able to deliver this content. Our curriculum group paired the resident lecturers with more experienced faculty to prepare their lecture content and questions. Prior knowledge of the basics of OB/GYN anatomy and pathophysiology were helpful in understanding the topics of this curriculum. The instructors had to have the ability to deliver case-based lectures.

We outlined the curriculum's content by reviewing the section for OB/GYN in *Tintinalli's Emergency Medicine: A Comprehensive Study Guide*[9-16] and the American Board of Emergency Medicine's Model of Clinical Practice.[17] Expert educators within the University of Wisconsin ED reviewed the content to ensure that it would cover information commonly evaluated in EM board examinations as well as information felt to be necessary to create an appropriate knowledge base for clinical care in the ED. The six topics chosen for these lectures were ectopic pregnancy and emergencies in the first 20 weeks (Appendix A), pregnancy emergencies after 20 weeks (Appendix B), delivery emergencies (Appendix C), pelvic pain in the nonpregnant patient (Appendix D), vaginitis/cervicitis/pelvic inflammatory disease (Appendix E), and abnormal uterine bleeding (Appendix F). We were concerned about having sufficient time in one session to cover delivery emergencies as well as perimortem cesarean section. As the latter was a rare procedure, we anticipated that residents would want the time to ask questions and fully explore the procedure. Therefore, a seventh lecture was created covering the basics of labor and delivery and perimortem C-section (Appendix G).

We presented the lectures throughout the academic year starting in August, with the final presentation at the end of April. The lectures were not presented at regular intervals due to the availability of conference scheduling slots. Each month had at least one session except for September, November, and January. March had two sessions as the delivery and perimortem C-section lecture was added to this month.

The curriculum was delivered during EM conference hours. Residents were expected to attend these lectures unless clinical duties required them to be away. As mentioned above, the goal was to encourage engagement from learners to reap the benefits of active learning. Therefore, questions to the audience progressed the lectures. The lectures were created using Microsoft PowerPoint. The created slide decks included talking points within the notes sections so instructors could present the slides with minimal preparation. Each session was 30 minutes long and included a case-based lecture followed by three to five review questions. Appendix H contains all the postsession review questions organized by lecture. After the initial session, sessions began with three to six review questions covering topics from previous lectures. These review questions were randomly selected from the prior sessions. Each question session lasted 5 minutes, and the lecture lasted 20 minutes. Our team utilized the free version of the game-based online software Kahoot! to deliver the questions in each session.

We presented most lectures in person in the residency conference room utilizing a projection system. However, due to the COVID pandemic, one session was presented over the videoconferencing software Zoom.

Prior to the curriculum, all eligible residents (34 total) completed a survey (Appendix I) assessing their confidence in caring for 11 emergent OB/GYN pathologies presenting to the ED along a 5-point Likert scale (1 = *strongly disagree,* 2 = *somewhat disagree,* 3 = *neither agree nor disagree,* 4 = *somewhat agree,* 5 = *strongly disagree*). Eligibility criteria included any active EM resident at the University of Wisconsin apart from those involved in developing the curriculum. Residents were also

Copyright © 2023 Burke et al. This is an open-access publication distributed under the terms of the Creative Commons Attribution license.

asked anonymous identifying questions to match their pre- and postcurriculum responses. The questions for the survey were created by the curriculum development team and reviewed by the department's Medical Education Scholarship Committee to ensure content validity. This survey assessed the first of Kirkpatrick's levels of training evaluation.[18] Following completion of the curriculum, the survey (Appendix J) was distributed again to evaluate for changes in resident confidence in managing OB/GYN complaints. To aid with the evaluation, residents were also asked which sessions they had been able to attend.

Results

Every resident attended at least one session, with an average of 18 residents per session (Table). In total, 76% of eligible learners (26 of 34) completed the precurriculum survey, 67% (23 of 34) completed the postcurriculum survey, and 44% (15 of 34) completed both. Residents were considered ineligible if they were part of the author group. When examining responses from all residents, mean reported comfort with the material improved significantly from a precurriculum rating of 3.5 to 4.0 ($p < .05$). This effect was even stronger when examining only residents who completed both surveys, with the average confidence score increasing from 3.4 to 4.0 ($p < .01$), with improvement reported for every individual topic (Figure). In an attempt to stratify the effect of individual sessions, we performed a comparison of the average increase in confidence of learners who were able to attend individual sessions against those who missed specific sessions. We did not see significant differences in reported confidence between learners who attended or missed individual sessions regarding those topics, though this result was limited by small numbers in individual subgroups.

We also asked residents to provide feedback on aspects of the curriculum they felt were beneficial or could be improved, with space given for free-text responses. Residents appreciated that the curriculum addressed a patient population less commonly encountered in their clinical environment and that the material was delivered in a case-based style with content specifically designed for EM learners. Residents noted limited time for sessions as an area for potential improvement and suggested adding simulation or small-group components.

Table. Number of Residents in Attendance at Each Session

Session	No. of Residents in Attendance[a]
Ectopic pregnancy and emergencies in the first 20 weeks	20
Pregnancy emergencies after 20 weeks	20
Delivery emergencies	20
Pelvic pain in the nonpregnant patient	18
Vaginitis, cervicitis, and pelvic inflammatory disease	17
Abnormal uterine bleeding	15
Labor and perimortem C-section	18

[a] There were 34 total residents in the program who were not part of the author group.

Discussion

This curriculum led to increased reported confidence in EM residents' ability to manage OB/GYN pathology presenting to the ED. The results were limited by the small number of responses, making us unable to evaluate for changes in confidence between those who were and were not able to attend each specific session.

There were limitations to our curriculum. Generalizability of our results may be limited as the curriculum was implemented at a single site. Kahoot! could have been used more robustly to examine knowledge retention over time with spaced repetition if learners had used persistent personal identifiers between games. However, since this was not initially known, many residents chose different nicknames when signing in to play the content review game. Additionally, the review questions were not always truly review as the audience was mixed between those who had and had not attended the prior session. Therefore, we were unable to reliably match specific residents to their performance over time.

Some feedback from residents mentioned that the sessions were too short. Each session was 30 minutes long, with the lecture portion being about 20 minutes of that time. This may have limited time for questions and learner involvement in the cases. The presenters of the curriculum also reported pressure due to time constraints. Therefore, future iterations of the curriculum could include added time to allow for more learner interaction. Another way to help with the large volume of information would be to provide a list of suggested readings to the learners prior to each session. These readings would introduce topics for the upcoming session. The topics in this curriculum could also be beneficial in other populations, such as family medicine residents, who evaluate similar pathologies in their clinics, sometimes work in EDs, and participate in labor and delivery. We intermittently had medical students present for our sessions, but we did not evaluate their perceptions of the curriculum. Future investigations could evaluate these learners' perceptions.

In the future, curriculum evaluation could be improved by evaluating higher Kirkpatrick levels. Our curriculum was primarily evaluated through residents' confidence in their understanding of the material presented. Previous literature has been mixed regarding correlation of confidence and clinical performance, with some curricula showing positive correlation between confidence

Copyright © 2023 Burke et al. This is an open-access publication distributed under the terms of the Creative Commons Attribution license.

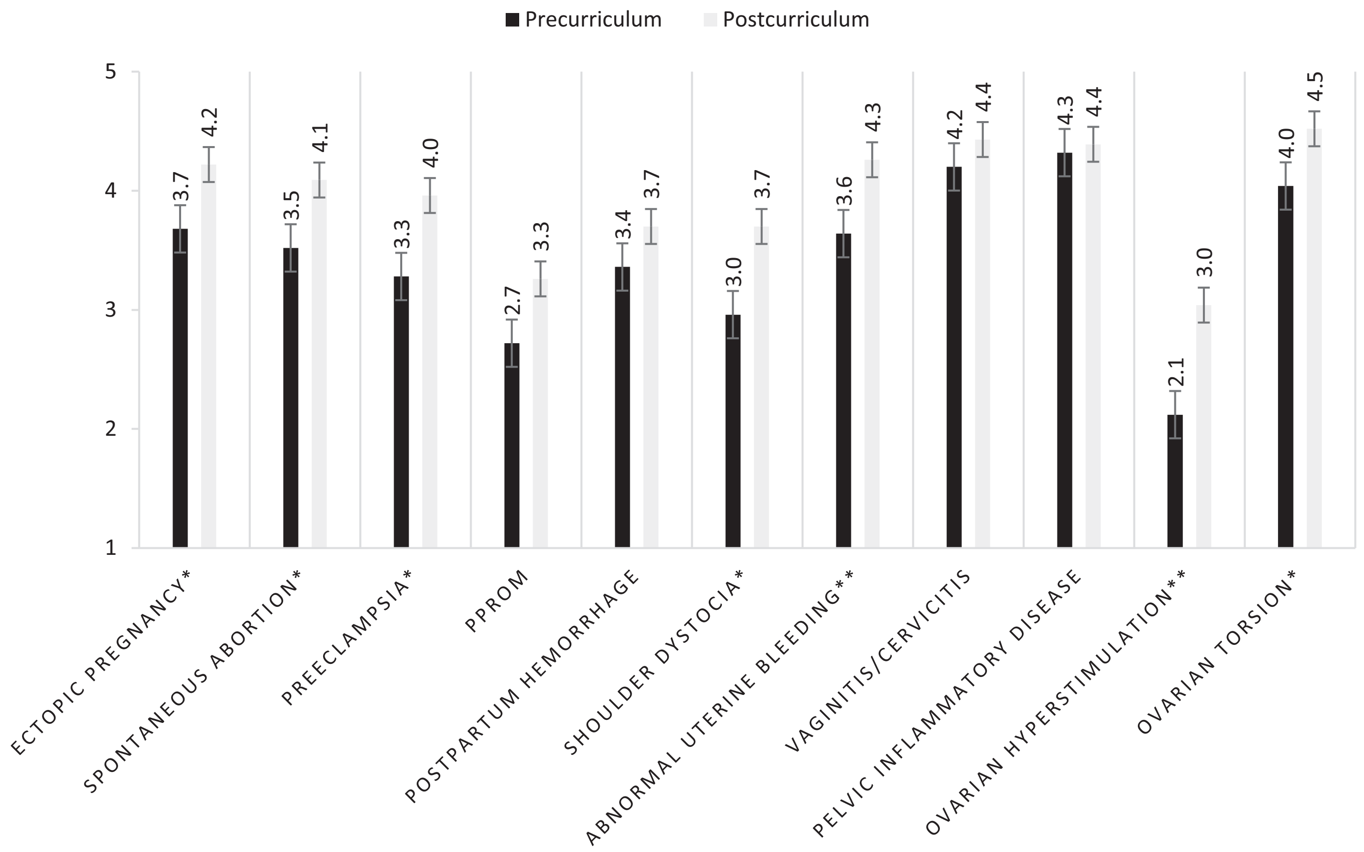

Figure. Average confidence scores and standard deviations (error bars), as rated on a 5-point Likert scale (1 = *strongly disagree*, 5 = *strongly agree*), of residents who responded to both the pre- and postcurriculum surveys. Abbreviation: PPROM, preterm premature rupture of membranes. $^{*}p < .05$. $^{**}p < .01$.

and clinical performance after an educational intervention and others finding no correlation.[19-22] Future iterations of this curriculum could include an evaluative component, such as simulated cases, to assess resident ability and further identify educational deficits.

Overall, our work represents a feasible and useful OB/GYN curriculum for EM residents. This curriculum could benefit other programs where these pathologies are less commonly encountered, as well as EM residents in general, since OB/GYN pathology is a core component of EM training.

Appendices

A. Ectopic Pregnancy and Emergencies in the First 20 Weeks.pptx
B. Pregnancy Emergencies After 20 Weeks.pptx
C. Delivery Emergencies.pptx
D. Pelvic Pain in the Nonpregnant Patient.pptx
E. Vaginitis, Cervicitis, and PID.pptx
F. Abnormal Uterine Bleeding.pptx
G. Labor and Perimortem C-Section.pptx
H. Session Review Questions.docx
I. Precurriculum Survey.docx
J. Postcurriculum Survey.docx

All appendices are peer reviewed as integral parts of the Original Publication.

Shannon M. Burke, MD: Assistant Professor, Department of Emergency Medicine, Medical College of Wisconsin; ORCID: https://orcid.org/0000-0001-7143-7793

Thaddeus Schmitt, MD: Education Scholarship Fellow, BerbeeWalsh Department of Emergency Medicine, University of Wisconsin School of Medicine and Public Health; ORCID: https://orcid.org/0000-0002-3228-8462

Copyright © 2023 Burke et al. This is an open-access publication distributed under the terms of the Creative Commons Attribution license.

Polly Kennedy, MD: Second-Year Resident Physician, BerbeeWalsh Department of Emergency Medicine, University of Wisconsin School of Medicine and Public Health

Brittany Kotek, MD: Second-Year Resident Physician, BerbeeWalsh Department of Emergency Medicine, University of Wisconsin School of Medicine and Public Health

James N. Wolfe, MD: Third-Year Resident Physician, BerbeeWalsh Department of Emergency Medicine, University of Wisconsin School of Medicine and Public Health

Corlin Jewell, MD: Assistant Professor, BerbeeWalsh Department of Emergency Medicine, University of Wisconsin School of Medicine and Public Health; ORCID: https://orcid.org/0000-0002-3551-6918

Kaitlin A. Ray, MD: Assistant Professor, Department of Emergency Medicine, Vanderbilt University Medical Center; ORCID: https://orcid.org/0000-0003-3841-0008

Benjamin H. Schnapp, MD, MEd: Associate Professor, BerbeeWalsh Department of Emergency Medicine, University of Wisconsin School of Medicine and Public Health; ORCID: https://orcid.org/0000-0001-5031-8269

Acknowledgments
The authors would like to acknowledge Kristen Sharp, MD, Clinical Associate Professor of Obstetrics and Gynecology at the University of Wisconsin School of Medicine and Public Health, who took the time to review the PowerPoint slides and session review questions for accuracy.

Disclosures
None to report.

Funding/Support
None to report.

Ethical Approval
Reported as not applicable.

References

1. Cairns C, Kang K, Santo L. *National Hospital Ambulatory Medical Care Survey: 2018 Emergency Department Summary Tables*. National Center for Health Statistics; date unknown. Accessed May 30, 2023. https://www.cdc.gov/nchs/data/nhamcs/web_tables/2018-ed-web-tables-508.pdf
2. Ricanati EHW, Thacker HL. The evolution of women's health education: the Cleveland Clinic's women's health fellowship as a model. *J Womens Health (Larchmt)*. 2007;16(7):1070-1075. https://doi.org/10.1089/jwh.2006.0118
3. Davisson L, Nuss M, Bruno C, et al. Women's health tutorial. *MedEdPORTAL*. 2009;5:1060. https://doi.org/10.15766/mep_2374-8265.1060
4. McGrath J, Mesiwala A, Barrie M, et al. Novel emergency medicine curriculum utilizing self-directed learning and the flipped classroom method: obstetric and gynecologic emergencies small group module. *J Educ Teach Emerg Med*. 2017;2(4):47-82. https://doi.org/10.5070/M524036765
5. Cooper MI, Papanagnou D, Meguerdichian M, Bajaj K. Emergency obstetrics for the emergency medicine provider. *MedEdPORTAL*. 2016;12:10481. https://doi.org/10.15766/mep_2374-8265.10481
6. Torralba KD, Doo L. Active learning strategies to improve progression from knowledge to action. *Rheum Dis Clin North Am*. 2020;46(1):1-19.
7. Freeman S, Eddy SL, McDonough M, et al. Active learning increases student performance in science, engineering, and mathematics. *Proc Natl Acad Sci U S A*. 2014;111(23): 8410-8415. https://doi.org/10.1073/pnas.1319030111
8. Szeto MD, Strock D, Anderson J, et al. Gamification and game-based strategies for dermatology education: narrative review. *JMIR Dermatol*. 2021;4(2):e30325. https://doi.org/10.2196/30325
9. Hang BS. Abnormal uterine bleeding. In: Tintinalli JE, Ma OJ, Yealy DM, et al., eds. *Tintinalli's Emergency Medicine: A Comprehensive Study Guide*. 9th ed. McGraw-Hill; 2020: chap 96.
10. Heniff M, Fleming HRB. Abdominal and pelvic pain in the nonpregnant female. In: Tintinalli JE, Ma OJ, Yealy DM, et al., eds. *Tintinalli's Emergency Medicine: A Comprehensive Study Guide*. 9th ed. McGraw-Hill; 2020:chap 97.
11. Heaton HA. Ectopic pregnancy and emergencies in the first 20 weeks of pregnancy. In: Tintinalli JE, Ma OJ, Yealy DM, et al., eds. *Tintinalli's Emergency Medicine: A Comprehensive Study Guide*. 9th ed. McGraw-Hill; 2020:chap 98.
12. Whelan LJ. Comorbid disorders in pregnancy. In: Tintinalli JE, Ma OJ, Yealy DM, et al., eds. *Tintinalli's Emergency Medicine: A Comprehensive Study Guide*. 9th ed. McGraw-Hill; 2020:chap 99.
13. Young JS. Maternal emergencies after 20 weeks of pregnancy and in the peripartum period. In: Tintinalli JE, Ma OJ, Yealy DM, et al., eds. *Tintinalli's Emergency Medicine: A Comprehensive Study Guide*. 9th ed. McGraw-Hill; 2020:chap 100.
14. Frasure SE, Kerrigan K. Emergency delivery. In: Tintinalli JE, Ma OJ, Yealy DM, et al., eds. *Tintinalli's Emergency Medicine: A Comprehensive Study Guide*. 9th ed. McGraw-Hill; 2020: chap 101.
15. Barclay-Buchanan CJ, Barton MA. Vulvovaginitis. In: Tintinalli JE, Ma OJ, Yealy DM, et al., eds. *Tintinalli's Emergency Medicine: A Comprehensive Study Guide*. 9th ed. McGraw-Hill; 2020: chap 102.
16. Weiss B, Shepherd SM. Pelvic inflammatory disease. In: Tintinalli JE, Ma OJ, Yealy DM, et al., eds. *Tintinalli's Emergency Medicine: A Comprehensive Study Guide*. 9th ed. McGraw-Hill; 2020: chap 103.
17. Beeson MS, Ankel F, Bhat R, et al.; American Board of Emergency Medicine. The 2019 model of the clinical practice of

Copyright © 2023 Burke et al. This is an open-access publication distributed under the terms of the Creative Commons Attribution license.

emergency medicine. *J Emerg Med*. 2020;59(1):96-120. https://doi.org/10.1016/j.jemermed.2020.03.018

18. Kirkpatrick J, Kirkpatrick WK. *An Introduction to the New World Kirkpatrick Model*. Kirkpatrick Partners; 2021. Accessed May 30, 2023. https://www.kirkpatrickpartners.com/wp-content/uploads/2021/11/Introduction-to-the-Kirkpatrick-New-World-Model.pdf
19. Day CS, Yeh AC, Franko O, Ramirez M, Krupat E. Musculoskeletal medicine: an assessment of the attitudes and knowledge of medical students at Harvard Medical School. *Acad Med*. 2007;82(5):452-457. https://doi.org/10.1097/ACM.0b013e31803ea860
20. Morgan PJ, Cleave-Hogg D. Comparison between medical students' experience, confidence and competence. *Med Educ*. 2002;36(6):534-539. https://doi.org/10.1046/j.1365-2923.2002.01228.x
21. O'Donoghue D, Davison G, Hanna LJ, McNaughten B, Stevenson M, Thompson A. Calibration of confidence and assessed clinical skills competence in undergraduate paediatric OSCE scenarios: a mixed methods study. *BMC Med Educ*. 2018;18:211. https://doi.org/10.1186/s12909-018-1318-8
22. Shi D, Liu J, Xu J, Zhu H, Yu X. Evaluation of a new goal-directed training curriculum for point-of-care ultrasound in the emergency department: impact on physician self-confidence and ultrasound skills. *Eur J Trauma Emerg Surg*. 2021;47(2): 435-444. https://doi.org/10.1007/s00068-019-01126-0

Received: September 5, 2022
Accepted: April 17, 2023
Published: August 11, 2023

Copyright © 2023 Burke et al. This is an open-access publication distributed under the terms of the Creative Commons Attribution license.
